# A 100,000-Fold Increase in C–H Bond Acidity Gives Palladium a Key Advantage in C(sp^3^)–H Activation Compared to Nickel

**DOI:** 10.1021/jacs.5c07649

**Published:** 2025-09-09

**Authors:** Lirong Lin, Tim K. Schramm, Pavel Kucheryavy, Roger A. Lalancette, Andreas Hansen, Demyan E. Prokopchuk

**Affiliations:** † Department of Chemistry, 67206 Rutgers University-Newark, Newark, New Jersey 07102, United States; ‡ Mulliken Center for Theoretical Chemistry, Clausius Institute for Physical and Theoretical Chemistry, Rhenish Friedrich Wilhelms University of Bonn, Bonn 53115, Germany

## Abstract

Carbon–hydrogen bond activation is a pillar of synthetic chemistry. While it is generally accepted that Pd is more facile than Ni in C–H activation catalysis, there are no experimental platforms available to directly compare the magnitude of C–H bond weakening between Ni and Pd prior to bond scission. This work presents the first direct measurements of C­(sp^3^)–H bond acidity (p*K*_a_) and bond dissociation free energy (BDFE) for a species containing a ligated alkane–palladium interaction (R_2_CH_2_···Pd), also known as an agostic interaction. Through standard-state equilibrium measurements and advanced computational modeling, we show that Pd acidifies C­(sp^3^)–H bonds 100,000 times more than Ni (5 p*K*_a_ units), indicating that acidification is a key factor making Pd a privileged metal in C­(sp^3^)–H functionalization reactions. Energy decomposition analysis (EDA) calculations show that this is primarily due to a greater electrophilicity of the palladium containing fragment, as forward charge transfer (Δ*E*_CTf_) from the agostic methylene moiety into [Pd] is significantly increased. More broadly, these valuable findings help unravel fundamental performance differences between Earth-abundant and precious metals, potentially guiding future ligand design efforts for catalysis.

## Graphical abstract

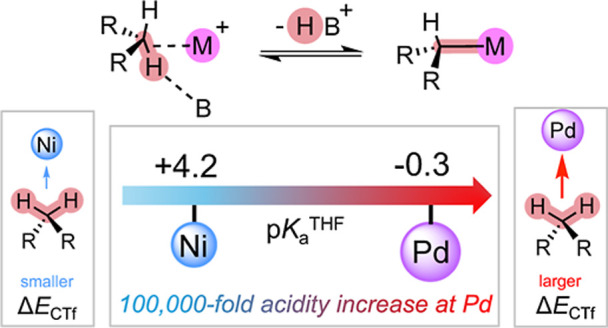

## Introduction

Breaking C–H bonds is an essential feature of synthetic organic chemistry and catalysis. While there is an established precedent for C–H bond functionalization catalyzed by precious metals (PMs) such as palladium,^1^ there is considerable momentum to implement more Earth-abundant metals (EAMs) such as nickel.^2^ This is not a simple task, because 3d metals have attenuated metal–ligand overlap which can lead to open-shell (paramagnetic) ground states, causing them to diverge from reaction pathways encountered by 4d metals.^3^ Moreover, facile 1e^–^ redox chemistry with Ni can promote bond homolysis during C–H activation and C–C coupling catalysis,^4,5^ while Pd and other PMs classically prefer 2e^–^ redox cycling. Therefore, these disparate electronic features between EAMs and PMs make it challenging to extract reactivity trends in C–H activation chemistry, making direct comparisons of catalyst performance under identical reaction conditions (ligand, solvent, base, additives, temperature, etc.) impractical in most cases.

Despite these challenges, researchers have made great strides in using Ni and Pd catalysts for the direct functionalization of unactivated C­(sp^3^)–H bonds through the use of directing ligands.^6–12^ Many catalysts follow a similar sequence of elementary steps, as illustrated at the top of Figure 1, albeit with different supporting ligands, bases, additives, and solvents. In 2005, Daugulis discovered that Pd­(OAc)_2_ was an active precatalyst for the arylation of C­(sp^3^)–H bonds under solvent-free (neat) conditions at 70–130 °C using an 8-aminoquinoline amide (LX) directing group and base (X’ = OAc).^13^ Upon attachment of LX to the metal center, a plausible C–H activation pathway involves the tethered alkane interacting with the metal center through an agostic (i.e., 3-center-2-electron) interaction,^14^ followed by cyclometalation and concomitant release of HX’. With simple inner-sphere bases such as acetate (OAc), the deprotonation event likely follows a concerted metalation-deprotonation (CMD) pathway to release HX’ (HOAc),^15–17^ and follow-up reactions with aryl halide promote C–C bond formation and product release. Mechanistic analysis on the cyclometalation step revealed facile and reversible C­(sp^3^)–H activation at temperatures as low as −35 °C in CD_2_Cl_2_, demonstrating the ease with which Pd can activate C­(sp^3^)–H bonds connected to auxiliary directing groups.^18^ In 2016 and 2017, landmark studies by Yu and Houk showed Pd-catalyzed C–H activation facilitated by the bidentate acetyl-protected aminoquinoline (APAQ) ligand, which assists with intramolecular deprotonation through a putative agostic C­(sp^3^)–H intermediate proposed via density functional theory (DFT).^19–21^ Related work by Rousseaux, Baudoin, and Clot demonstrates the importance of agostic interactions and base selection during Pd-catalyzed C­(sp^3^)–H arylation reactions.^22–24^

**Figure 1:**
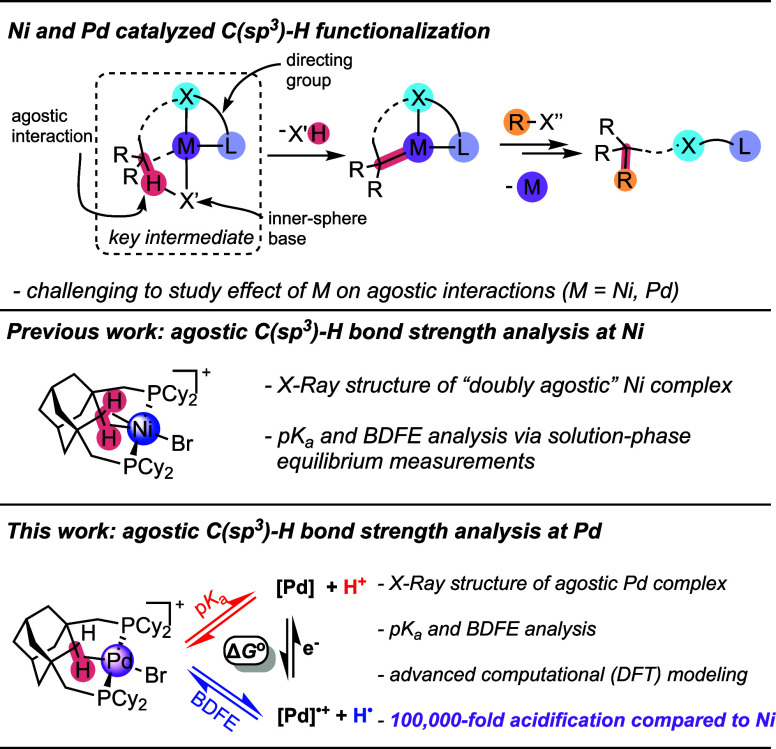
Top: Generic reaction sequence showing chelation-assisted C­(sp^3^)–H functionalization catalysis at Ni and Pd, emphasizing a key proposed intermediate containing an agostic interaction. Middle: previous work with Ni, establishing C­(sp^3^)–H bond strengths for a “doubly agostic” interaction trapped within an adamantyl pincer ligand. Bottom: an isostructural Pd complex, whose agostic C­(sp^3^)–H bond strength is described in this work using a suite of solid state, solution phase, and computational methods.

In 2014, Chatani and Ge independently reported the Ni-catalyzed C­(sp^3^)–H functionalization using 8-aminoquinoline amide as an LX directing group.^25,26^ Both studies used a precatalyst cocktail containing a Ni^II^ source (NiX_2_), ligand (phosphine, ArCOOH), carbonate base (Cs_2_CO_3_, Na_2_CO_3_), polar or nonpolar solvent (toluene, *N*,*N*′-dimethylacetamide, *N*,*N*′-dimethylformamide), and high temperature (130–140 °C). In 2017, a follow-up computational study by Liu suggested that the C­(sp^3^)–H activation step occurs via concerted metalation-deprotonation (CMD) through a network of solvated (Na_2_CO_3_)_*x*_(DMF)_*y*_ intermediates to form a cyclometalated Ni complex.^27^ Later, Baik and Chang proposed a similar route using pivalate (X’ = OPiv) in acetonitrile at 120 °C.^28^ Rigorous mechanistic analysis by Johnson revealed that the open-shell complex Ni^II^(LX)_2_ is capable of activating substrate C­(sp^3^)–H bonds and the use of stronger base (NaO^t^Bu) enables milder temperatures (100 °C) for catalysis.^29^ Nonetheless, these examples underscore the disparate reaction conditions in Ni and Pd catalyst systems, making broad generalizations about the impact of the metal center on C­(sp^3^)–H bond cleavage challenging to ascertain.

Since the above examples involve CMD pathways, simultaneous C–H cleavage and O–H formation makes it challenging to unravel C–H bond strengths, as internal bases will have different M–O bond strengths that might modulate their effective basicity when interacting with the acidified C–H bond. Thus, the absence of quantitative data describing the magnitude of coordination-induced C­(sp^3^)–H bond weakening in Ni- and Pd-catalyzed reactions prompted us to investigate molecular platforms amenable to C–H bond thermochemical analysis. In 2022, we showed that a robust adamantyl (diamondoid) PCP pincer ligand can trap a structurally authenticated “doubly agostic” CH_2_···Ni moiety,^30,31^ enabling us to quantify homolytic and heterolytic C–H bond strengths under standard-state conditions (Figure 1, middle).^32^ Isolation of complex **[NiCH**_**2**_ **]** ^**+**^ involved protonolysis of the alkyl group using the oxonium acid [H­(OEt_2_)_2_]^+^, a reagent^33,34^ which has been used by Brookhart and co-workers to facilitate σ-methane adduct formation under cryogenic conditions.^35^ We described an equilibrium-based approach for measuring the acidity (p*K*_a_) and bond dissociation free energy (BDFE) of the agostic methylene group coordinated to nickel, with a measured p*K*_*a*_^THF^ of 4.2 and bond dissociation free energy (BDFE) of 70 kcal/mol in tetrahydrofuran (THF).^32^ DFT calculations were also in excellent agreement with experiment. These data represented tremendous decreases in heterolytic and homolytic C–H bond strengths relative to free adamantane (p*K*_a_^DMSO^ ≅ 54,^36^ BDE_CH_ ≅ 100 kcal/mol^37^) via coordination-induced bond weakening,^38,39^ underscoring the participation of transition metals in C­(sp^3^)–H activation reactions. Nonetheless, a gap remains in the scientific literature to better understand the fundamental differences in C–H activation performance with group 10 transition metals, particularly between Earth-abundant and precious metals (i.e., Ni vs Pd).

This work presents the first direct measurements of C­(sp^3^)–H bond acidity (p*K*_a_) and bond dissociation free energy (BDFE) for an agostic methylene (R_2_CH_2_) group interacting with Pd in solution, enabling direct C–H bond strength comparisons with its Ni congener (Figure 1). We compare the underlying thermochemical parameters for C­(sp^3^)–H bond weakening between isostructural Ni and Pd pincer complexes via standard-state equilibrium measurements in nonpolar solvent, showing that Pd acidifies C­(sp^3^)–H bonds a 100,000 times more than Ni (5 p*K*_a_ units) while increasing the C–H BDFE and redox potential for the Pd^III/II^ redox couple. The acid–base chemistry with Pd is significantly more complex when compared to Ni,^32^ and variable temperature NMR spectral analysis was undertaken to obtain accurate thermochemical data sets. State-of-the-art DFT calculations integrated into an efficient multilevel workflow provided detailed insights into the spatial arrangement of all species under equilibrium, highlighting their intrinsic conformational flexibility, which was critical for understanding the solution-phase dynamics. Finally, we show that these drastic acidity enhancements at Pd are correlated with its enhanced electrophilicity and help explain reactivity trends in Pd-based C­(sp^3^)–H functionalization catalysis, as substitution for Ni in DFT-computed energy profiles results in more energetically demanding C–H bond scission (i.e., less acidic C­(sp^3^)–H bonds). In a broader context, this experimental and computational work helps unravel fundamental performance differences between Earth-abundant and precious metals in C–H bond activation reactions.

## Results and Discussion

### Structural Analysis of Pd Complexes after Protonation

To begin our C–H activation studies with Pd, the air-stable pincer complex **[PdBr]** is synthesized by mixing the free ligand and PdBr_2_(COD) under basic conditions (COD = 1,5-cyclooctadiene), followed by a modified workup and purification via column chromatography (page S6, Supporting Information).^40^ In line with our previous protonation studies using nickel,^32^ low-temperature dropwise addition of the oxonium acid [H­(OEt_2_)_2_]­[B­(C_6_F_5_)_4_][Bibr ref34] to **[PdBr]** changes the solution color from yellow to orange, and two Pd containing products are generated in solution (Figure 2a). Crystallization via toluene-pentane layering produces X-ray quality crystals of a minor yellow-orange product and a major colorless product, which are the agostic complex **[PdCH**_**2**_ **]** ^**+**^ and bimetallic species **[Pd–Br–Pd]** ^**+**^, respectively—starkly contrasting with the clean protonation chemistry at nickel to quantitatively produce **[NiCH**_**2**_ **]** ^**+**^ under similar conditions.^32^ Single crystal X-ray diffraction reveals that complex **[PdCH**_**2**_ **]** ^**+**^ contains an unsymmetrically bound methylene group interacting with the metal center (Figure 2b, middle). The methylene protons bound to C47 are located in the Fourier difference map, with Pd···H distances of 1.67(4) Å and 2.25(3) Å. The short Pd1···H47B distance and acute C–H···Pd angle (127(4)°) indicate that this C–H···Pd interaction is an incredibly rare example of a structurally authenticated agostic interaction with Pd (i.e., 3-center-2-electron CH···M bond).^41^ The agostic nature of this interaction is also justifiable when considering the observed upfield chemical shift^14^ in solution-phase ^1^H NMR spectra (0.49 ppm, CD_2_Cl_2_; Figure S29). Notably, the reaction in Figure 2a is not mass-balanced, and a rigorous analysis of the equilibrium processes involving **[PdCH**_**2**_ **]** ^**+**^ and **[Pd–Br–Pd]** ^**+**^ are crucial to understanding all subsequent acid–base reactions and determining the agostic C­(sp^3^)–H bond acidity, as described in more detail below.

**Figure 2:**
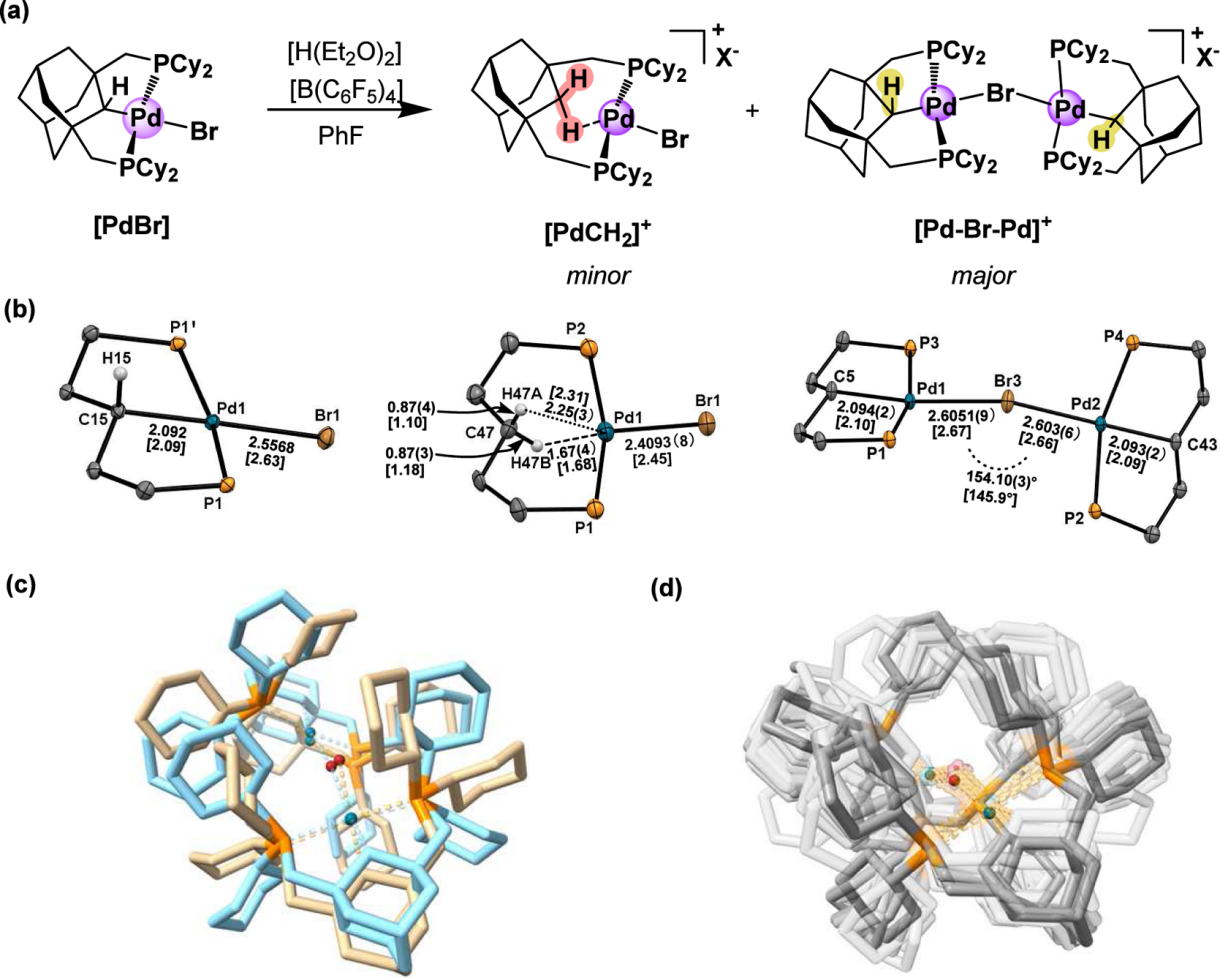
(a) Protonation of **[PdBr]** with [H­(OEt_2_)_2_]­[B­(C_6_F_5_)_4_] to form **[PdCH**_**2**_ **]** ^**+**^ and **[Pd–Br–Pd]** ^+^ which are characterized by single crystal X-ray diffraction (not balanced). (b) Core molecular structures of **[PdBr]** (left), **[PdCH**_**2**_ **]** ^**+**^ (middle), and **[Pd–Br–Pd]** ^**+**^ (right) obtained from single crystal X-ray diffraction (50% probability ellipsoids). Most carbon atoms, hydrogen atoms, cocrystallized solvent, and [B­(C_6_F_5_)_4_]^−^ are omitted for clarity. Additional metrical parameters are discussed in the main text and full structures are shown in Figure S64. X^–^ = B­(C_6_F_5_)_4_^–^. Numbers in brackets correspond to DFT-optimized (r^2^SCAN-3c/CPCM/CH_2_Cl_2_) bond lengths and angles after the multilevel workflow (**[PdBr]**, **[PdCH**_**2**_ **]** ^**+**^) or by relaxing the experimental solid-state geometry (**[Pd–Br–Pd]** ^**+**^). (c) Structural overlay of the DFT-optimized experimental solid-state structure (light brown) and the lowest conformer obtained after the multilevel workflow (blue) for **[Pd–Br–Pd]** ^**+**^ (see main text) with hydrogen atoms omitted for clarity. (d) Structural overlay of all conformers contributing to the calculated energy shift due to thermostatistical (Boltzmann) averaging over the structure ensemble of **[Pd–Br–Pd]** ^**+**^ in solution (see Supporting Information for details). A nonenergy-ranked transparency gradient is applied to all conformers except for the lowest-energy structure, and all hydrogen atoms have again been omitted for clarity.

While the Pd–Br bond length for **[PdBr]** (2.5568(3) Å) is consistent with other *trans*-alkylpalladium bromide complexes,^42–47^ the strongly *trans*-influencing adamantyl moiety enables the preparation of **[Pd–Br–Pd]** ^**+**^ in 80% yield by treating **[PdBr]** with 0.5 equiv. K­[B­(C_6_F_5_)_4_] (Figure 3). X-ray crystallographic analysis of **[Pd–Br–Pd]** ^**+**^ reveals that each Pd­(II) center is in a slightly distorted square planar geometry (τ_4_ = 0.15 and 0.18)^48^ and connected by a canted bromide bridge (∠Pd1–Br3–Pd2 = 154.10(3)°), which tends to be more linear in the solution-phase structure as predicted by DFT (∠Pd1–Br3–Pd2 = 168.7°). The chelating pincer ligands planes are approaching orthogonality, as the dihedral angles P1–Pd1–Pd2–P2 and P3–Pd1–Pd2–P4 are 59.5° and 62.6°, respectively.

**Figure 3:**
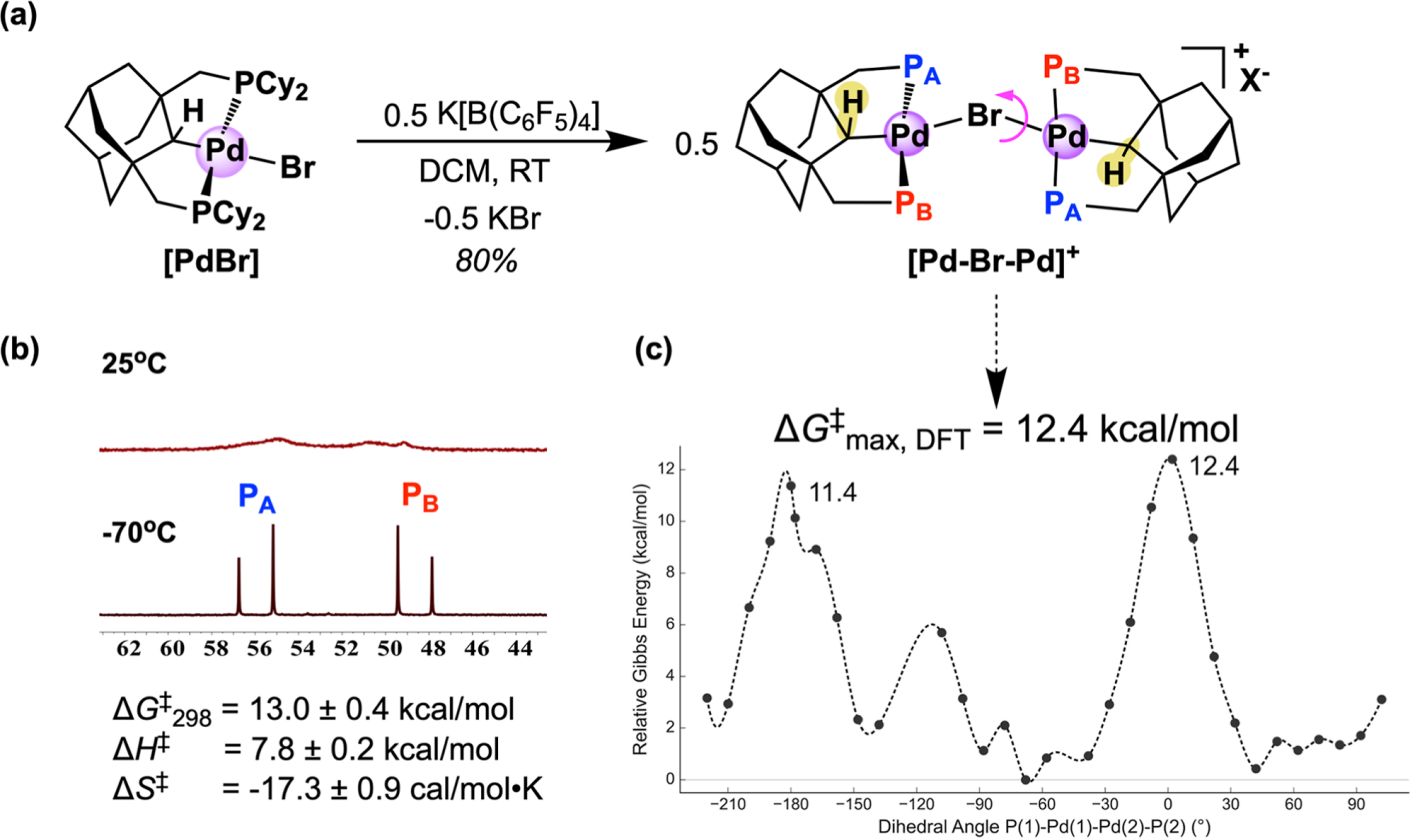
(a) Synthesis of **[Pd–Br–Pd]** ^**+**^ by mixing **[PdBr]** and K­[B­(C_6_F_5_)_4_] in CH_2_Cl_2_ (left), showing rotational motions along the Pd–Br–Pd axis in solution. Labels P_A_ and P_B_ denote the chemically inequivalent phosphines in ^31^P NMR spectra (X^–^ = B­(C_6_F_5_)_4_^–^). (b) Kinetic parameters determined via Eyring analysis, with sample ^31^P NMR spectra of **[Pd–Br–Pd]** ^**+**^ (CD_2_Cl_2_). (c) Gibbs energy profile along the P1–Pd1–Pd2–P2 dihedral angle in **[Pd–Br–Pd]** ^**+**^, with each point representing the lowest-energy conformer at the given constrained dihedral angle. Gibbs energies near local maxima are annotated (kcal/mol, relative to the global minimum), highlighting approximate rotational barriers. Final energies computed at the PBE0-D4/def-QZVPP/COSMO-RS (fine, CH_2_Cl_2_) level of theory.

### Solution-phase Characterization of [PdCH_2_]^+^ and [Pd–Br–Pd]^+^

To characterize **[PdCH**_**2**_ **]** ^**+**^ in solution, ^1^H, ^13^C, and ^31^P NMR spectral data sets were acquired in CD_2_Cl_2_ after the protonation of **[PdBr]** with [H­(OEt_2_)_2_]­[B­(C_6_F_5_)_4_] (Figures S29–S32). In ^1^H NMR spectra, the agostic methylene protons (red, Figure 2a) appear as one broad peak at ca. 0.49 ppm between 25 °C and −70 °C, which are ca. 2.4 ppm more shielded than the α-hydrogen moiety of **[PdBr]** (2.90 ppm). The observation of a single CH_2_···Pd resonance for **[PdCH**_**2**_ **]** ^**+**^ indicates rapid intramolecular exchange of the asymmetric methylene that cannot be resolved on the NMR time scale. This process is consistent with a symmetrically oriented CH_2_···Pd transition state and a low computed barrier height of Δ*G*_298K_^‡^ = 1.7 kcal/mol, corresponding to a half-life time of only τ_1/2_ = 2 ps at room temperature^49^ as predicted at the PBE0-D4 ^50–52^ /def2-QZVPP^53,54^ level of theory with COSMO-RS­(fine)^55–57^ continuum solvation, which was found to be best-performing on a small benchmark study provided in Part B of the Supporting Information. If not stated otherwise, this level of theory will be used throughout to report all DFT-computed thermochemical data. The ^13^C NMR spectrum for **[PdCH**_**2**_ **]** ^**+**^ reveals a triplet at 58.51 ppm (^3^ *J*_CP_ = 11.4 Hz) belonging to the *C*H_2_···Pd moiety and a lowered C–H coupling constant for the agostic hydrogens of **[PdCH**_**2**_ **]** ^**+**^ (^1^ *J*_CH_ = 110 Hz).^58,59^ These data are wholly consistent with the agostic moiety in the analogous complex **[NiCH**_**2**_ **]** ^**+**^ (δ­(^13^C) = 48.5 ppm, ^3^ *J*_CP_ = 17 Hz, ^1^ *J*_CH_ = 106 Hz).^32^ The ^31^P NMR spectrum of **[PdCH**_**2**_ **]** ^**+**^ shows a singlet 66.4 ppm at 298 K and is slightly deshielded relative to **[PdBr]** (51.6 ppm), owing to the increase in overall charge and subsequent loss of the Pd–alkyl bond.

The bromide-bridged complex **[Pd–Br–Pd]** ^**+**^ appears in all acid–base equilibria in this study, therefore it was prudent to conduct a detailed analysis of its solution-phase behavior using experimental and computational approaches. The X-ray crystallographic structure of **[Pd–Br–Pd]** ^**+**^ provides an excellent starting point for our solution-phase DFT optimizations, which faithfully reproduce the metrical data within expected deviations from experiment, using the efficient and robust r^2^SCAN-3c^60^ composite method combined with CPCM^61,62^ implicit solvation.

As expected, the lowest-energy structure obtained from solution-phase conformational sampling^63–65^ of **[Pd–Br–Pd]** ^**+**^ is significantly different from the one obtained after relaxing the experimental solid-state structure, with Figure 2c illustrating a structural overlay of both conformers. Please refer to Section VII of the Supporting Information for a discussion of their relative (conformational) energy and the strong method-dependence thereof.^50–57^ These significant changes in conformation hint at a dynamic behavior of **[Pd–Br–Pd]** ^**+**^ in solution and non-negligible conformational flexibility, which is further visualized by the structural overlay of the whole conformer ensemble (CE) of **[Pd–Br–Pd]** ^**+**^ in Figure 2d. To approximately account for this conformational flexibility, all computed Gibbs energies in this study have been corrected by a thermostatistically averaged conformational shift calculated over the full CE^66^ (eq 28 in the Supporting Information).

An approximate 2-fold rotation axis bisecting the Pd­(PCP) moieties in **[Pd–Br–Pd]** ^**+**^ places the *trans*-phosphines P_A_ and P_B_ within each pincer ligand into unique chemical environments in the solid state, and low-temperature ^31^P NMR spectra in CD_2_Cl_2_ clearly shows two doublets with an AB splitting pattern for the inequivalent *trans*-phosphines (55.9 and 48.6 ppm, −70 °C; ^2^ *J*_PP_ = 318.6 Hz)^67^ that coalesce as temperatures are increased (Figure 3). In a higher boiling solvent such as fluorobenzene, peak coalescence is achieved at ca. 70 °C (Figure S4). We rationalize that this temperature dependence is due to free rotation of the pincer ligand around the Pd–Br–Pd “axle”, making the phosphines chemically indistinguishable on the NMR time scale as the rate of rotation increases (Figure 3b). Line broadening simulation and Eyring analysis (Figures S6 and S7) shows that this dynamic process has a relatively low free energy barrier (Δ*G*_298K_^‡^ = 13.0 ± 0.4 kcal/mol) with a significant entropic penalty (Δ*S* ^‡^ = −17.3 ± 0.9 cal/(mol·K)). Within error, the solution-phase kinetics are nearly identical in fluorobenzene (Figures S4–S7). DFT calculations strongly corroborate this dynamic rotation mechanism as scanning the P1–Pd1–Pd2–P2 dihedral angle reveals a comparable free energy barrier of approximately 12.4 kcal/mol (*k*_rot_ = 5 × 10^3^ s^–1^)^49^ when the pincer ligands adopt a mutually parallel orientation (i.e., ∠P1–Pd1–Pd2–P2 = 0°; Figure 3c). The dihedral energy profile serves as an appropriate model for the molecular dynamics in solution, with each data point representing a fully conformationally optimized structure at its corresponding dihedral angle. Given the numerous degrees of freedom in the system, localizing a singular transition pathway thus seems unlikely. Further, ^1^H–^1^H EXSY NMR experiments of **[Pd–Br–Pd]** ^**+**^ show no signs of chemical exchange processes occurring at room temperature (Figure S28).

### Equilibrium Thermochemistry of [PdCH_2_]^+^ and [Pd–Br–Pd]^+^

A balanced chemical equilibrium is shown in Figure 4a after protonation of **[PdBr]** with [H­(OEt_2_)_2_]­[B­(C_6_F_5_)_4_] in fluorobenzene. Reaction workup and ^1^H NMR spectroscopic analysis in CD_2_Cl_2_ reveals a highly dynamic and selective exchange process involving protons within the ethereal adduct **[PdCH**_**2**_ **·Et**_**2**_ **O]** ^**+**^ (0.45 ppm; red), the conformationally flexible **[Pd–Br–Pd]** ^**+**^ (3.60 ppm; yellow), and the proposed bromonium salt **[HBrH·2OEt**_**2**_ **]** ^**+**^ (16.31 ppm; green, Figures 4 and S41). We emphasize that the removal of exogenous Et_2_O after the protonation of **[PdBr]** is achieved by drying under high vacuum, leaving behind only Et_2_O that is intimately involved in the acid–base chemistry in Figure 4a. Proton exchange behavior is also clearly observed through ^1^H–^1^H EXSY NMR experiments,^68,69^ showing intense in-phase cross peaks at three different protonation sites (Figure 4b). By using the cross-peak to diagonal peak intensity ratio, we find that the proton exchange is slow at room temperature, with *k*_ex_ = 0.32 ± 0.7 s^–1^ (Δ*G*_298_^‡^ = 22.4 ± 1.2 kcal/mol; Table S3).

**Figure 4:**
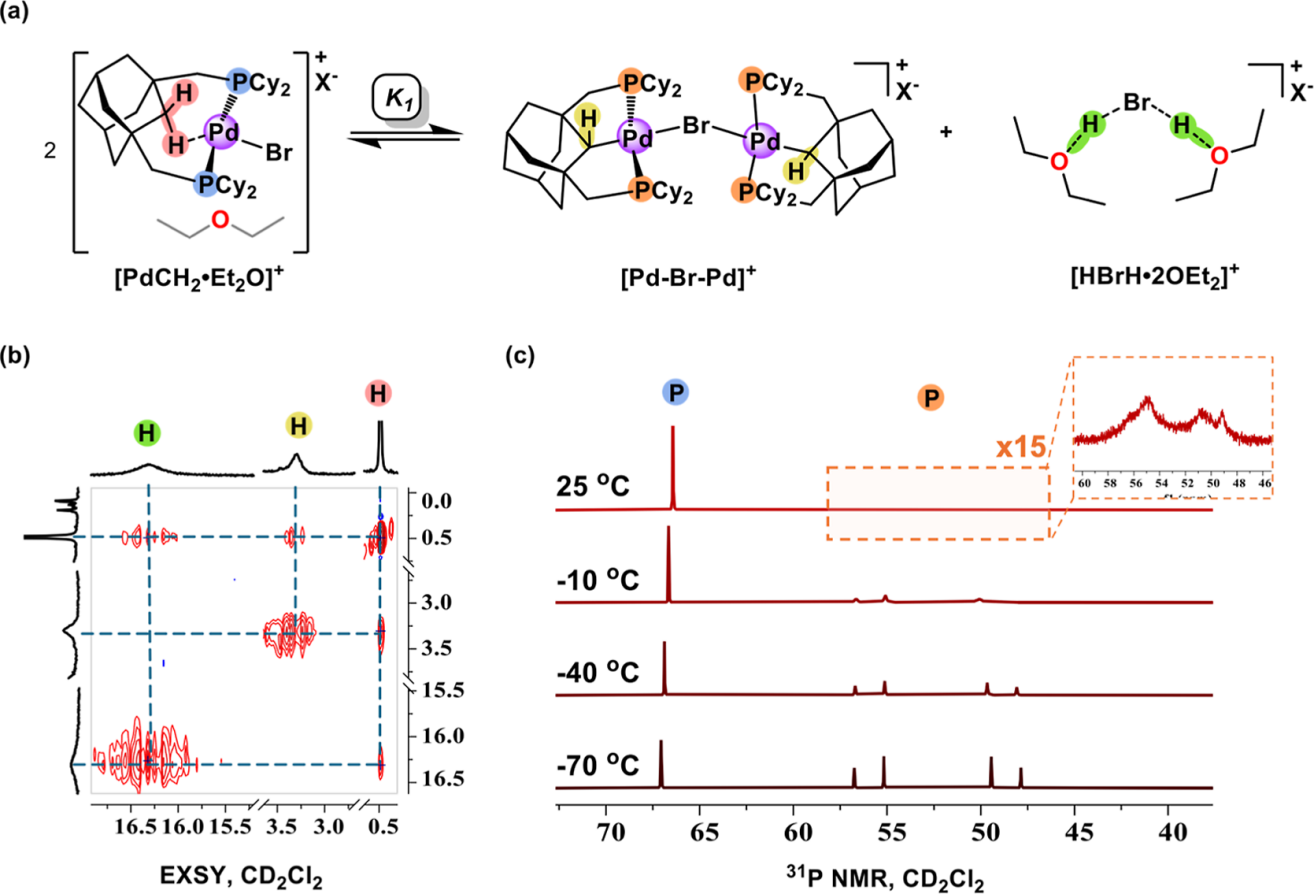
(a) Proton exchange equilibrium between **[PdCH**_**2**_ **·Et**_**2**_ **O]** ^**+**^, **[Pd–Br–Pd]** ^**+**^, and **[HBrH·2OEt**_**2**_ **]** ^**+**^ corresponding to the equilibrium constant ***K***_**1**_. Exchanging protons are colored in red, yellow, and green, respectively. X^–^ = B­(C_6_F_5_)_4_^–^. (b) ^1^H–^1^H EXSY NMR (CD_2_Cl_2_) of the equilibrium mixture at room temperature shows intense in-phase cross peaks indicating proton exchange at three different protonation sites. (c) Variable temperature inverse-gated ^31^P NMR (CD_2_Cl_2_) of the equilibrium mixture, with spectra acquired in 30 °C intervals. The ^31^P NMR spectra show a systematic decrease of **[PdCH**_**2**_ **·Et**_**2**_ **O]** ^**+**^ (blue) at 66.4 ppm, accompanied by an increase of **[Pd–Br–Pd]** ^**+**^ (55.9 (d), 48.7 (d), orange) as the temperature is lowered.

The bromonium adduct **[HBrH·2OEt**_**2**_ **]** ^**+**^ is flanked by two coordinated Et_2_O molecules (δ ≅ 4.0 ppm, br, O­(*CH*_2_CH_3_)_2_), which exhibit a through-space relationship to the strongly deshielded resonance at 16.31 ppm (*H*–Br–*H*; green in Figures 4a, S37). While the interaction of anhydrous HX (X = Br, Cl) with ethereal molecules to generate oxonium ions has been studied to some extent,^70,71^ precedent for the bonding motif shown in Figure 4a has only been structurally authenticated in one instance, where two O = AsPh_3_ moieties are coordinated through their oxygen atoms to an [H···Br···H]^+^ core.^72^ Careful refinement of the employed workflows enabled the computational description of such noncovalently bound adducts (e.g., **[HBrH·2OEt**_**2**_ **]** ^**+**^ or **[PdCH**_**2**_ **·Et**_**2**_ **O]** ^**+**^; see Supporting Information Sections II. and IV. for details), further supporting the proposed bromonium adduct. The [H···Br···H]^+^ motif was also identified as an anilinium variant via diffusion-ordered NMR spectroscopy (DOSY) and computations, as described in the following section.

The concentrations of **[PdCH**_**2**_ **·Et**_**2**_ **O]** ^**+**^, **[HBrH·2OEt**_**2**_ **]** ^**+**^, and **[Pd–Br–Pd]** ^**+**^ can be perturbed by lowering the reaction temperature or by substitution of H^+^ with D^+^ during the protonation of **[PdBr]**. Van’t Hoff analysis via ^31^P NMR spectroscopy provides a complete picture of the thermochemical behavior in solution (Figure 5), showing that the equilibrium process *K*_1_ is enthalpically driven toward **[Pd–Br–Pd]** ^**+**^ formation (Δ*H* = −1.42 ± 0.05 kcal/mol; Table 1). However, this is countered with a negative entropic term (Δ*S* = −5.44 ± 0.20 cal/(mol·K)), yielding a slightly positive standard-state free energy (Δ*G*_298K_ = 0.20 ± 0.08 kcal/mol). While exhaustive deuteration of all exchanging protonation sites is not possible under these experimental conditions, reacting **[PdBr]** with one equivalent of the isotopologue [D­(OEt_2_)_2_]­[B­(C_6_F_5_)_4_] results in partial deuteration and a *normal* equilibrium isotope effect (EIE) of *K*_H_/*K*_D_ = 1.4 ± 0.2 at 283 K, representing a mixture of primary and secondary isotope effects from deuterium scrambling (Figure 5, bottom). at these temperatures yield low signal-to-noise ratios for the Van’t Hoff analysis shown in Figure 5, the raw EIE values measured at these three temperatures also yield normal EIEs.

**Figure 5:**
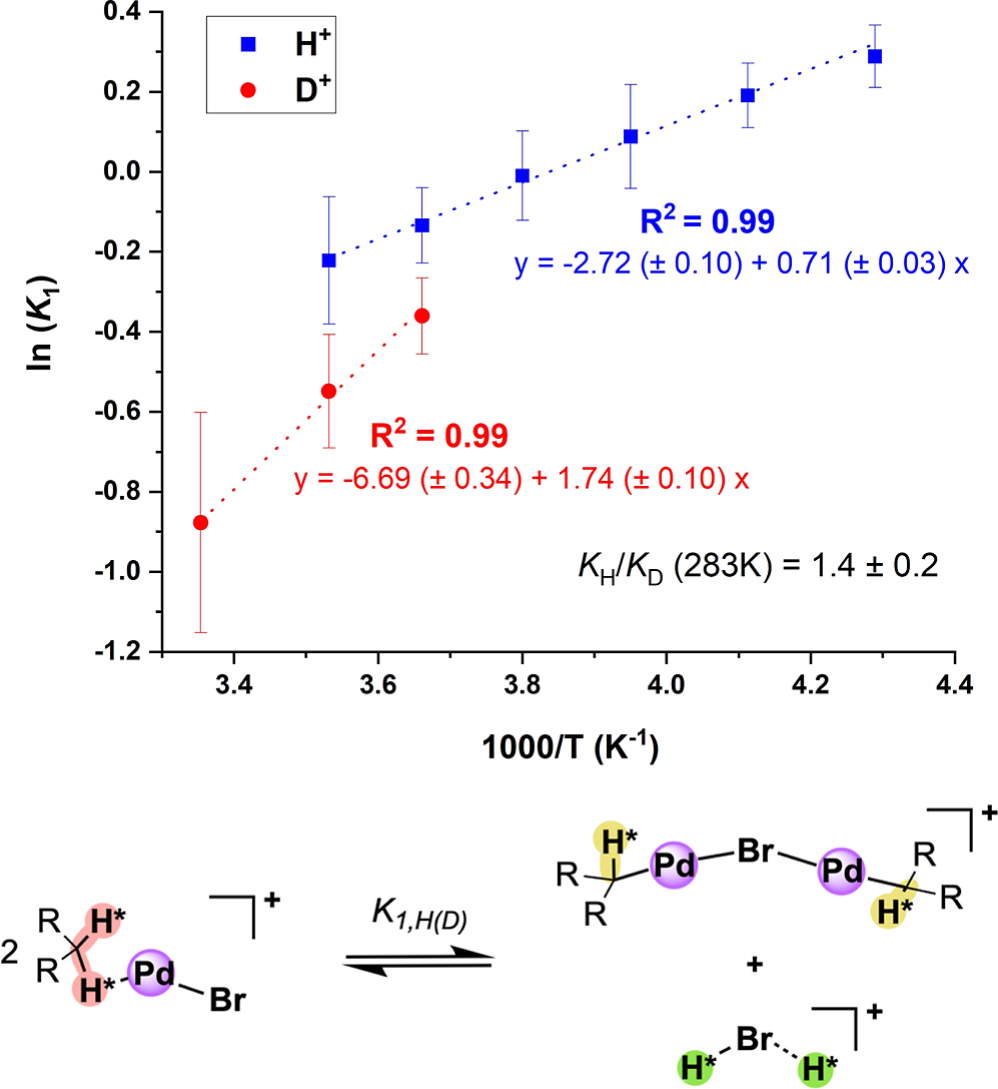
Top: Linear region of the Van’t Hoff plot for the equilibrium constant *K*_1_ after protonation of **[PdBr]** with [H­(OEt_2_)_2_]­[B­(C_6_F_5_)_4_] (blue) and [D­(OEt_2_)_2_]­[B­(C_6_F_5_)_4_] (red), displaying a calculated equilibrium isotope effect of 1.4 ± 0.2 (EIE: *K*_H_/*K*_D_) at 283 K. Bottom: equilibrium process showing possible sites of H/D exchange with asterisks (only one equivalent D^+^ added per equivalent **[PdBr]**). The counterion is B­(C_6_F_5_)_4_^–^ in all structures.

**Table 1:** Experimental and Computed Thermochemical Data for the Equilibrium Processes *K*_1_, *K*_2_, *K*_3_, and *K*_ip_′^a^

		Δ*H* (kcal/mol)	Δ*S* (cal/(mol·K))	Δ*G*_298_ (kcal/mol)	Δ*G*_DFT,298_ ^d^ (kcal/mol)	*K*_H_/*K*_D_ (283 K)
***K***_**1**_ ^b^	H^+^	–1.4 ± 0.1	–5.4 ± 0.2	0.20 ± 0.08	0.9	1.4 ± 0.2
	D^+^	–3.5 ± 0.2	–13.4 ± 0.7	0.52 ± 0.29	1.1	(1.5)
***K***_**2**_ ^b^		–0.86 ± 0.02	–3.7 ± 0.1	0.25 ± 0.04	–0.8	
***K***_**3**_ ^c^		–5.6 ± 1.9	–20.1 ± 6.6	0.3 ± 2.8	–2.9	
***K***_**ip**_ **′** ^c^		–9.0 ± 0.6	–26.0 ± 2.0	–1.3 ± 0.8	–2.1	

a The computational EIE is given in parentheses (see Figures S67 and S68).

b Data collected and calculated in CD_2_Cl_2_.

c Data collected and calculated in 1,2-dichloroethane (DCE).

d Final energies computed at the PBE0-D4/def-QZVPP/COSMO-RS­(fine) level of theory.

Since relative differences in relative zero-point energies (ZPEs) between reactants and products are usually the most dominant upon isotopic substitution,^73^ the normal EIE suggests that the relative differences in ZPE between the ensemble average of protio and deutero adducts is larger for the products (i.e., ΔZPE­(products) > ΔZPE­(reactants)).^74,75^ However, the significantly more negative entropic contribution outcompetes the enthalpic term when using D^+^ as a reagent, driving the standard-state free energy slightly upward under standard-state conditions (Δ*G*_298K_ = 0.52 ± 0.29 kcal/mol). The origins of the more negative entropic term upon isotopic substitution are not clearly understood. Even with full deuteration of all exchanging sites, it would be challenging to predict the position of the EIE, as the ZPE term is driven by enthalpic factors which contribute to inverse EIEs while several other entropic factors contribute to normal EIE behavior.^73^ Deuterium (^2^H) NMR spectra of this equilibrium mixture confirm H/D scrambling, showing CD_2_···Pd (0.87 ppm) and CDH···Pd (0.45 ppm) for the agostic complex, CD-Pd (3.60 ppm) belonging to **[Pd–Br–Pd]** ^**+**^, and D···Br···D^+^ (15.79 ppm) assigned to the bromonium adduct (Figures S38–S40). In ^1^H NMR spectra, isotopic perturbation shifts the proton resonance of the agostic CDH···Pd to 0.06 ppm at 25 °C relative to CH_2_···Pd (Δδ = −0.42 ppm)^76^ and cooling the probe to −70 °C shields the CHD resonance even further (−0.25 ppm; Figure S42).

Computationally modeling *K*_1_ and all related equilibria in Table 1 is highly nontrivial, as the experimental Gibbs energy changes are comparable to typical density functional errors.^77–79^ Additionally, solvation free energy errors for charged species in nonaqueous media can easily reach up to 5 kcal/mol per species,^80^ making favorable error cancellation essential for quantitative results. This is further amplified by the presence of delicate ion-pair effects, which are not captured in our calculations, as the solvent and other solutes are treated as a uniform dielectric. To assess the accuracy of various computational approaches, we used some of the equilibrium data (DCM: ***K***_**1**_, ***K***_**2**_, ***K***_**3**_, DCE: ***K***_**3**_) to set up a small benchmark, employing seven density functionals known for good performance paired with four different implicit solvation models, which finally yielded PBE0-D4 ^50–52^ /def2-QZVPP^53,54^ with COSMO-RS­(fine)^55–57^ continuum solvation as the most reliable method combination for the systems at hand (see Supporting Information part B for detailed discussions). Despite the inherent difficulties in obtaining precise equilibrium data as described above, the calculated reaction energies show good agreement with experiment, albeit the formation of **[Pd–Br–Pd]** ^**+**^ in DCE appears somewhat too exergonic (***K***_**2**_ and ***K***_**3**_, see discussion below; Table 1). Our computations are consistent with the experimentally measured EIE (Section VI of the Supporting Information), with the reaction free energy being most perturbed when deuterium is placed in the nonagostic methylene position of **[PdCH**_**2**_ **·Et**_**2**_ **O]** ^**+**^ (i.e., with higher stretching frequency), consistent with a report by Shapley and co-workers for an agostic osmium complex.^76^

### Equilibrium Thermochemistry Using Anilinium Acids

Complex **[PdCH**_**2**_ **]** ^**+**^ is unstable in common organic solvents such as tetrahydrofuran (THF) and acetonitrile (MeCN), prompting us to use 1,2-dichloroethane (DCE) as the reaction medium for determining the agostic C­(sp^3^)–H bond acidity of **[PdCH**_**2**_ **]** ^**+**^.^81^ Anilinium-derived conjugate acids are uniquely suited for ion-pair equilibrium measurements in DCE because a strong correlation exists between their p*K*_ip_^DCE^ and p*K*_a_^MeCN^, enabling the transposition of acidity data into other solvents such as THF by using experimentally parametrized equations established by Leito and co-workers.^82–84^ Therefore, we opted to react **[PdBr]** with the acids 2,6-dichloroanilinium tetrakis­(pentafluorophenyl)­borate (**[H**_**3**_ **NAr** ^**Cl**^ **]­[B­(C**_**6**_ **F**_**5**_ **)**_**4**_ **]**; p*K*_ip_^DCE^ = 1.1 ± 0.5) and 2,4-difluoroanilinium tetrakis­(pentafluorophenyl)­borate (**[H**_**3**_ **NAr** ^**F**^ **]­[B­(C**_**6**_ **F**_**5**_ **)**_**4**_ **]**; p*K*_ip_^DCE^ = 4.4 ± 0.5). To avoid contamination from exogenous Et_2_O in these acid–base equilibria, these acids were prepared by bubbling anhydrous HCl_(g)_ through a solution containing the conjugate base and K­[B­(C_6_F_5_)_4_] in CH_2_Cl_2_ (Figure S3). The acid **[H**_**3**_ **NAr** ^**Cl**^ **]­[B­(C**_**6**_ **F**_**5**_ **)**_**4**_ **]** produces X-ray quality crystals upon workup.

Mixing one equivalent of the weaker acid **[H**_**3**_ **NAr** ^**F**^ **]** ^**+**^ with **[PdBr]** does not result in formation of the adduct **[PdCH**_**2**_ **]** ^**+**^ (Figure 6, top). Instead, Br^–^ loss generates a mixture of **[Pd–Br–Pd]** ^**+**^ and another putative [HBrH]^+^ adduct **[H**_**2**_ **Br­(H**_**2**_ **NAr** ^**F**^ **)**_**2**_ **]** ^**+**^, proposed such that hydrogen bonding interactions persist between the bromide ion and N–H protons in a similar manner to the ethereal acid **[HBrH·2OEt**_**2**_ **]** ^**+**^ described earlier. Moreover, we argue that the weakly interacting acid–base adduct **[PdBr·H**_**3**_ **NAr** ^**F**^ **]** ^**+**^ is the dominant species on the lefthand side of the equilibrium expression due to the presence of a new singlet at 52.7 ppm in ^31^P NMR spectra (Figure S14), with the Pd–C*H* resonance clearly observed via low temperature ^1^H NMR (Figure S49). The standard-state change in Gibbs free energy for equilibrium *K*_2_ is 0.25 ± 0.04 kcal/mol at 298 K, which is extracted via Van’t Hoff analysis in a similar manner as for *K*_1_ (Table 1 and Figures S15 and S16). Notably, the lack of observable **[PdCH**_**2**_ **]** ^**+**^ hints at the agostic C­(sp^3^)–H bond being significantly more acidic compared to **[NiCH**_**2**_ **]** ^**+**^, as **[H**_**3**_ **NAr** ^**F**^ **]** ^**+**^ cleanly generates an equilibrium mixture of **[NiBr]** and **[NiCH**_**2**_ **]** ^**+**^ in DCE.^32^

**Figure 6:**
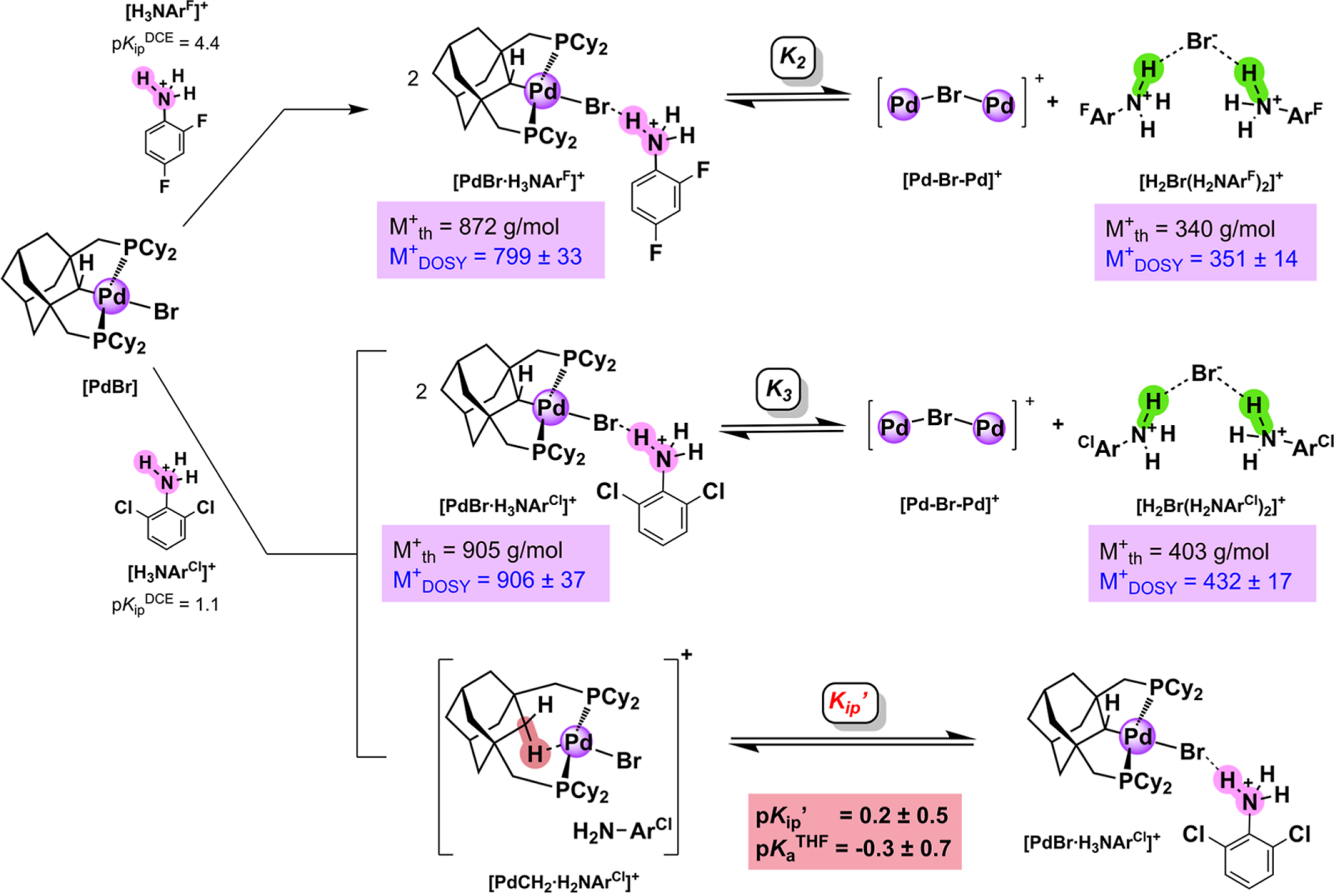
Top: Acid–base equilibrium after protonation of **[PdBr]** with 2,4-difluoroanilinium (**[H**_**3**_ **NAr** ^**F**^ **]** ^**+**^) in CD_2_Cl_2_. Bottom: Acid–base equilibria after protonation of **[PdBr]** with 2,6-dichloroanilinium (**[H**_**3**_ **NAr** ^**Cl**^ **]** ^**+**^) in DCE. Purple boxes: theoretical molecular weights (M_th_^+^) and molecular weights via DOSY NMR spectroscopy (M_DOSY_^+^) using an external calibration curve (see Supporting Information for full details). Red box: experimentally determined p*K*_ip_′ in DCE and calculated p*K*_a_^THF^ using empirical conversion equations.^81,83^ The counteranion is B­(C_6_F_5_)_4_^–^ in all cases. Note that the protonation reactions starting from **[PdBr]** are not balanced to show the stoichiometries used in measuring equilibrium constants.

To experimentally validate the molecular weight and aggregation state of the putative anilinium adducts **[H**_**2**_ **Br­(H**_**2**_ **NAr** ^**F**^ **)**_**2**_ **]** ^**+**^ and **[PdBr·H**_**3**_ **NAr** ^**F**^ **]** ^**+**^ in solution (Figure 6, top), we employed DOSY NMR with an external calibration curve (ECC) methodology, shown to be effective for accurate molecular weight determination of small molecules in common organic solvents.^85–87^ Using existing ECCs led to poor fits with the molecules in this study because the published data do not contain heavy atoms such as Br and Pd, which have a disproportionate effect on the molecular density.^85^ Thus, it was necessary to construct a new “heavy atom” ECC using molecules of comparable molar densities and geometries (Table S13 and Figure S53). Using this new heavy atom ECC, diffusion coefficients (*D*_*x*_) were measured for the aromatic C–H resonance at 7.23 ppm of the **[H**_**3**_ **NAr** ^**F**^ **]** ^**+**^ moiety, providing evidence for the proposed adducts in equilibrium *K*_2_ (Figure 6, purple boxes). Performing an inverse Laplace transform (ILT) on the DOSY NMR spectra^88–90^ enabled us to clearly observe that this aromatic C–H resonance is associated with two solution-phase adducts containing dramatically different diffusion coefficients, and hence, molecular weights (Table S14 and Figure S54). We also note that the assignment of M_DOSY_^+^ = 799 ± 33 for adduct **[PdBr·H**_**3**_ **NAr** ^**F**^ **]** ^**+**^ is lower than expected from theory (M_th_^+^ = 872 g/mol), suggesting that bromide loss may simultaneously generate the dication **[Pd–H**_**3**_ **NAr** ^**F**^ **]** ^**2+**^ in solution (M_th_^+^ = 792 g/mol).

Next, the stronger acid **[H**_**3**_ **NAr** ^**Cl**^ **]** ^**+**^ (p*K*_ip_^DCE^ = 1.1 ± 0.5) was mixed with one equivalent of **[PdBr]** in DCE. Gratifyingly, the four-component mixture now contains the agostic complex **[PdCH**_**2**_ **·H**_**2**_ **NAr** ^**Cl**^ **]** ^**+**^, along with **[PdBr·H**_**3**_ **NAr** ^**Cl**^ **]** ^**+**^, **[Pd–Br–Pd]** ^**+**^, and **[H**_**2**_ **Br­(H**_**2**_ **NAr** ^**Cl**^ **)**_**2**_ **]** ^**+**^ (Figure 6, bottom, and Figure S18). Analogous to reactions with **[H**_**3**_ **NAr** ^**F**^ **]** ^**+**^ described above, the adduct **[PdBr·H**_**3**_ **NAr** ^**Cl**^ **]** ^**+**^ can be identified via three key features: (1) an observable Pd–C*H* resonance in low temperature ^1^H NMR experiments that is correlated to a ^31^P resonance at 52.4 ppm via ^1^H–^31^P HMBC experiments at −70 °C (Figures S50 and S51); (2) through-space coupling between the anilinium and cyclohexylphosphine moieties in ^1^H–^1^H NOESY spectra at −70 °C (Figure S52); (3) a cation with a mass of 906 ± 37 g/mol from ECC-calibrated DOSY NMR, corresponding to **[PdBr·H**_**3**_ **NAr** ^**Cl**^ **]** ^**+**^ (Table S14 and Figure S55). Therefore, the acid does not dissociate from **[PdBr]** in solution. Moreover, the same DOSY spectrum identifies a diffusion coefficient cross-peak at 7.38 ppm, consistent with the mass of bromonium adduct **[H**_**2**_ **Br­(H**_**2**_ **NAr** ^**Cl**^ **)**_**2**_ **]** ^**+**^. Using variable-temperature ^31^P NMR spectroscopy, the relative concentrations can be measured in DCE, enabling the calculation of ***K***_**3**_ (Table 1). Determining product distributions and equilibrium constants by varying the concentration of **[H**_**3**_ **NAr** ^**Cl**^ **]** ^**+**^ proved to be challenging, as ^31^P NMR integral accuracy for all species undergoing chemical exchange became poorer across a wide range of temperatures.

In addition to the above evidence supporting the formation of adduct **[PdBr·H**_**3**_ **NAr** ^**Cl**^ **]** ^**+**^, we do not observe free **H**_**2**_ **NAr** ^**Cl**^ via ^1^H NMR spectroscopy, suggesting that the agostic adduct **[PdCH**_**2**_ **·H**_**2**_ **NAr** ^**Cl**^ **]** ^**+**^ is dominant in solution (Figure S50). Since these anilinium acids and conjugate bases do not freely dissociate in solution, a “true” ion-pair equilibrium (***K***_**ip**_) cannot be directly measured. The lack of solvation with neutral strong acids in DCE is well documented, where strongly acidic molecules are found to directly donate their proton to bases dissolved in solution.^91^ Unfortunately, the range of alternative acid candidates to perform such equilibrium measurements is very limited, as only anilinium-based acids have a strong correlation between p*K*_ip_^DCE^ and p*K*_a_^MeCN^, are soluble enough in DCE to perform these measurements, and are compatible with the complexes used in this study.^81^ Therefore, we resort to define this reaction as an ion-pair adduct equilibrium ***K***_**ip**_ **′**(eq 1),

1 $${[PdCH}_{2}\cdot \cdot \cdot H_{2}{NAr}^{Cl}{]}^{+}⇌{\left[PdBr\cdot \cdot \cdot H_{3}{NAr}^{Cl}\right]}^{+},{K_{ip}}^{\prime }$$

where Δ*G*_298_ = −1.3 ± 0.8 kcal/mol, Δ*H* = −9.0 ± 0.6 kcal/mol, and Δ*S* = −26.0 ± 2.0 cal/mol·K in DCE (Table 1). The origins for a negative entropic term in ***K***_**ip**_ **′** are not clearly understood, and we speculate that non-negligible ion-pairing effects with [B­(C_6_F_5_)_4_]^−^ affect the translational entropy. Nevertheless, the free energy computed via DFT best fits this model (Δ*G*_**DFT**_ = −2.1 kcal/mol), and the next likely candidate deviates from experiment by 2.9 kcal/mol ([PdCH_2_···H_2_NAr^Cl^]^+^ ⇌ [PdBr] + [H_3_NAr^Cl^]^+^; Δ*G*_DFT_ = 1.6 kcal/mol). Further, two other computed acid/base dissociation equilibria are in much poorer agreement with experiment (ΔΔ*G* = 5.7–9.4 kcal/mol, Figure S77).

Using the experimentally determined ***K***_**ip**_ **′** to determine **p** *K*_**ip**_ **′** in DCE and transposing this data into **p** *K*_**a**_^**THF**^ results in **p** *K*_**ip**_ **′** = 0.2 ± 0.5 and **p** *K*_**a**_^**THF**^ = −0.3 ± 0.7 (Figure 6, red box). Therefore, the acidification of this agostic C­(sp^3^)–H interaction at Pd represents a *100,000-fold increase* in C–H bond acidity (ca. 5 p*K*_a_ units) when compared to Ni.^32^ Interestingly, the less acidic agostic C­(sp^3^)–H interaction at Ni helps rationalize why Johnson and co-workers determined that using a stronger base (NaO^*t*^Bu) in place of Na_2_CO_3_ (commonly used in Pd-catalyzed reactions) resulted in significantly higher catalyst turnover under milder conditions for the arylation of *N*-(quionolyn-8-yl)-pivalamide.^29^

### Agostic C–H Bond Thermochemistry

With the acid–base equilibrium chemistry now firmly established, we turned our attention to obtaining a more complete picture of the agostic C–H bond thermochemistry. Attempts were made to measure the redox potential for **[PdBr]** ^**+/0**^ in THF with 0.2 M [^*n*^Bu_4_N]­[B­(C_6_F_5_)_4_] as the supporting electrolyte. At scan rates as high as 50 V/s, an irreversible oxidation feature was observed, with *E*_pa_ = 0.81 V vs Fc^+/0^, preventing us from obtaining an accurate redox potential for the Pd^III/II^ couple (Table 2 and Figures S57–S59). Switching to a noncoordinating solvent such as fluorobenzene does not impart redox reversibility (Figures S61–S63). If this were a reversible redox event, the oxidation peak potential would be even more positive, however rapid consumption of the electrogenerated species cathodically shifts the observed peak potential. Therefore, we use this measured *E*_pa_ as a rough estimate for subsequent thermochemical analyses. The validity of this approach is indirectly corroborated by isodesmic DFT analysis (S76), as the computed difference in potential between Pd^III/II^ and Ni^III/II^ is 0.56 V, only about 100 mV less than the estimate via experiment (0.655 V). Although the fate of Pd is unclear upon oxidation, we determined that the irreversible Pd^II/III^ oxidation and follow-up chemical process likely occurs through a stepwise mechanism under kinetic control of a slow electron transfer step, as ∂*E*_p_/∂logν = 58 mV and (*E*_p/2_ – *E*_p_)_avg_ = 77 mV for CV traces scanned from 100 to 5000 mV/s (Figures S58–S60).^92^ Collectively, these observations are generally consistent with the reticence of Pd^II^ toward generating a stable odd-electron species (along with other precious metals), as isolable mononuclear organometallic Pd^III^ complexes are still quite rare.^93,94^

**Table 2:** C­(sp^3^)–H Bond Thermochemical Data in THF

	p*K*_a_^THF^ (C–H)	*E* (M^III/II^) (V vs Fc^+/0^)	BDFE_CH_ (kcal/mol)
**Pd**	–0.3 ± 0.7^b^	0.810 (*E*_pa_)	79^c^
**Ni** ^32^	4.2 ± 0.7	0.155 (*E*°)	69.8 ± 2.2
**Δ(Pd–Ni)** ^a^	–4.5 ± 1.0^b^(−6.0)	0.655 (0.51)	9 (3.5)

a Computational data at the PBE0-D4/def-QZVPP/COSMO-RS­(fine, THF) level of theory shown in parentheses. Relative differences in p*K*_a_ *E*°, and BDFE are determined using isodesmic schemes as described in Figure S76.

b Estimated using *K*_ip_′^DCE^, see the main text and Supporting Information for details.

c Estimated using the experimental *E*_pa_ and *K*_ip_′^DCE^.

Using a similar isodesmic reaction approach to compute the difference in acidity between **[PdCH**_**2**_ **]** ^**+**^ and **[NiCH**_**2**_ **]** ^**+**^, Δp*K*_a_ = −6.0, about 1.5 orders of magnitude lower than experiment (−4.5 ± 1.0; Table 2). This difference suggests that the “true” p*K*_a_^THF^ of the agostic C–H bond at Pd is probably lower than −0.3, as the computed Δp*K*_a_ does not depend on externally referenced acids or conjugate bases. For the same reason, the estimated (BDFE_CH_) = 79 kcal/mol for Pd is likely overestimated by around 2 kcal/mol since the p*K*_a_^THF^ is expected to be more negative, as governed by the “Bordwell” relationship:^95,96^ BDFE_CH_ = 1.364p*K*_a_ + 23.06*E*° + *C*_G,THF_.^97^ The agostic C–H bond acidity at Pd is modulated by the inverse correlation of *E*° and BDFE_CH_ on the observed p*K*_a_, with the significantly larger Pd^III/II^ oxidation potential outweighing the smaller increase in BDFE_CH_, thus tipping the balance toward generating a more acidic C–H bond. Collectively, this data is graphically presented in Figure 7 and paints a clear picture of agostic C–H bond strength differences between Ni and Pd, with the latter attenuated through a 100,000-fold increase in acidity relative to Ni (Δp*K*_a_ = 4.5 ± 1.0).

**Figure 7:**
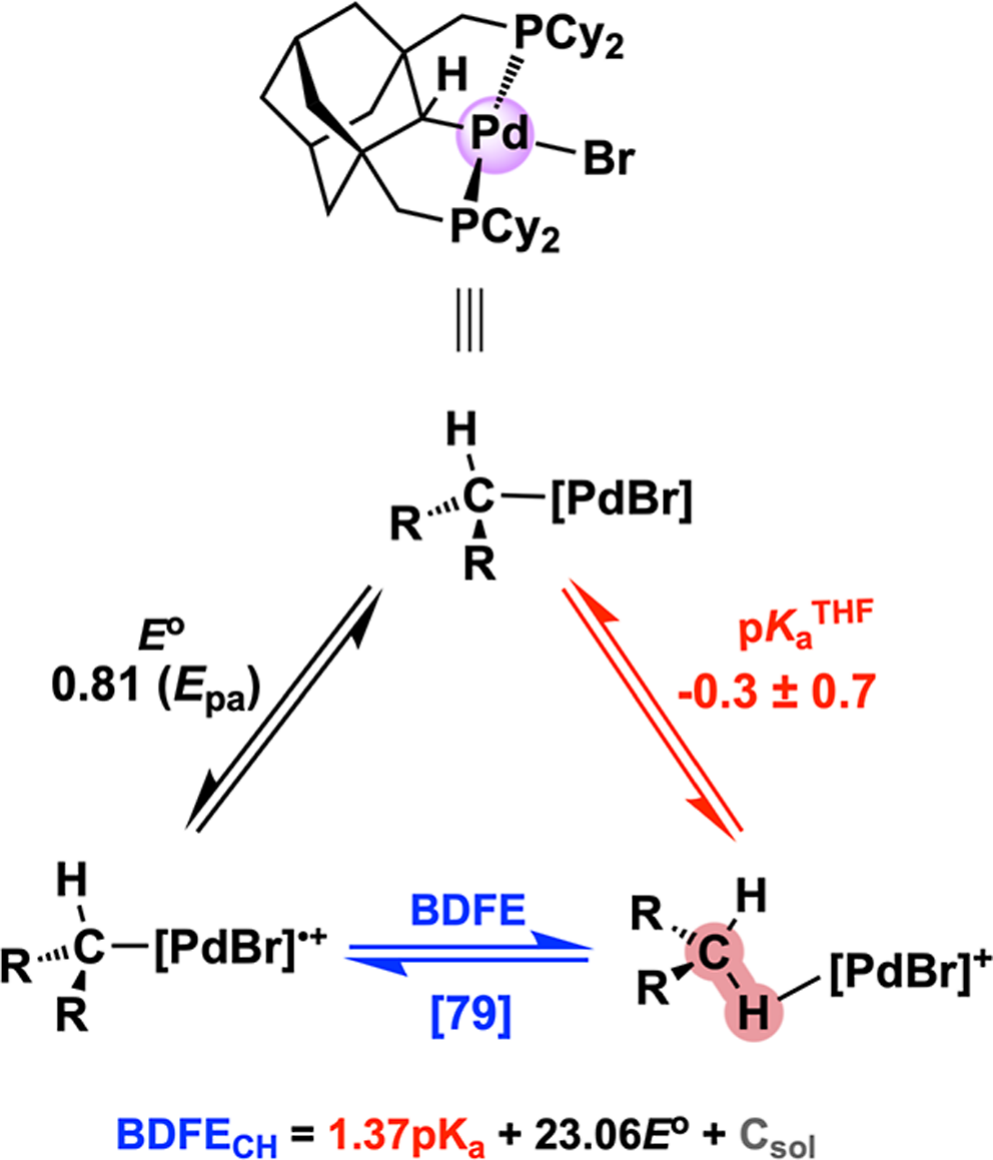
Experimental thermochemical parameters for **[PdBr]**, **[PdBr]** ^**•+**^, and **[PdCH**_**2**_ **]** ^**+**^ in THF, assembled in part by using the transposed ion-pair equilibrium constant (*K*_ip_′) in DCE. The BDFE is shown in brackets as this value was calculated using the experimental *E*_pa_ in THF.

### Energy Decomposition Analysis of the Agostic Interaction

After thoroughly examining the thermochemical data for these isostructural Ni and Pd complexes, we sought to rationalize our findings based on more comprehensive molecular orbital (MO) theory. For this, we carried out a bonding analysis using Head-Gordon’s energy decomposition analysis (EDA),^98–100^ which is based on absolutely localized MOs, called ALMOs.^101^ Within ALMO-EDA, the total interaction energy (Δ*E*_INT_) between two molecular fragments can be separated into physically meaningful energy contributions. These are permanent electrostatics (Δ*E*_ELEC_), Pauli repulsion as the energy penalty associated with occupied orbital overlap (Δ*E*_PAULI_), attractive dispersion (Δ*E*_DISP_), induced electrostatics as polarization (Δ*E*_POL_), and finally, interfragment electron delocalization also known as charge-transfer (Δ*E*_CT_). Notably, all energy contributions can be obtained with consistent inclusion of continuum solvation effects,^102^ which primarily affect the sum of Δ*E*_ELEC_, ΔE_PAULI_ and ΔE_DISP_ that is usually referred to as the frozen energy Δ*E*_FRZ_. It is possible to obtain unidirectional CT contributions from Δ*E*_CT_,^103^ for which we consider charge flow from a ligand to the metal (e.g., the agostic H_2_C → M interaction) as charge flow in the *forward* direction (CTf). Hence, any backbonding from the metal to its ligands can be described as *backward* CT (CTb).

Before applying ALMO-EDA to the agostic interactions in **[NiCH**_**2**_ **]** ^**+**^ and **[PdCH**_**2**_ **]** ^**+**^, we first had to address one major limitation of the EDA scheme, as it is not capable of isolating the agostic interaction of the adamantane (Ad) moiety when it is covalently embedded into the multidentate pincer ligand with further interactions to the metal center. Hence, we constructed a model system with fully isolated adamantane and phosphine units by removing the connecting methylene “arms” of the pincer ligand and replacing those with hydrogen atoms to construct distinct ligands consisting of the adamantane moiety and the remainder of the molecule. Considering that both HPCy_2_ ligands exhibit almost identical EDA components, two fragmentations are possible, with either adamantane ([CH_2_]) or one of the HPCy_2_ units as a fragment and the remainder of [M’CH_2_]^+^ as the second fragment (Figure 8a legend, colored and noncolored regions). When choosing the former, the total interaction energy becomes positive for both metals (hatched bars), which is in stark contrast to the one obtained with the HPCy_2_ fragmentation (solid bars), thus nicely reconfirming the need for the multidentate pincer ligand to structurally constrain the agostic interaction.^32^ Subsequent DFT optimizations only varied the positions of the hydrogen atoms in these structures, while fixing all heavy atom coordinates to yield model systems closely resembling the full [MCH_2_]^+^ complexes (see Figure S69). We refer to these sliced structural representations of [MCH_2_]^+^ as [Ni’CH_2_]^+^ and [Pd’CH_2_]^+^, respectively.

**Figure 8:**
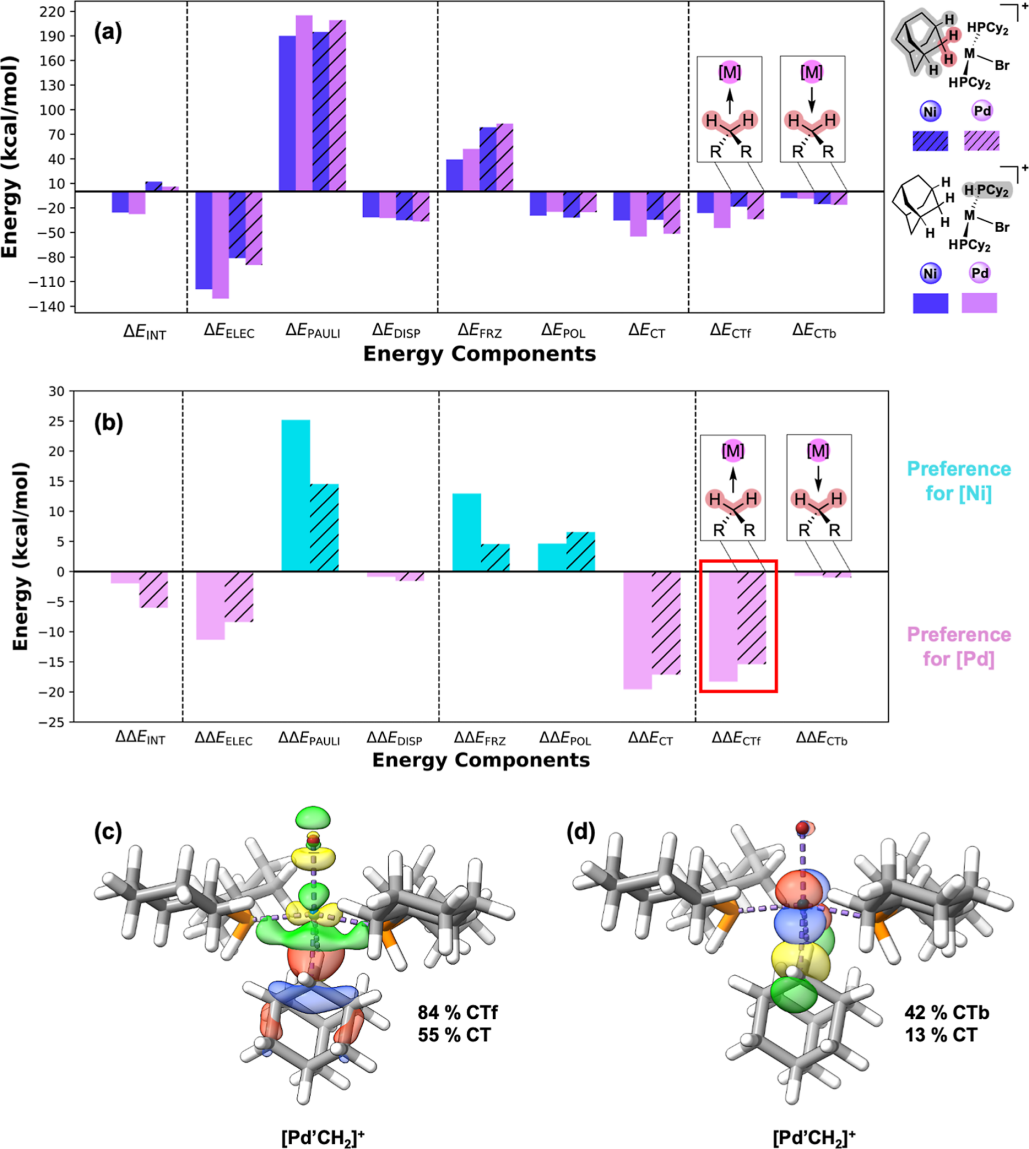
(a) Decomposition of the total interaction energy (Δ*E*) into frozen, polarization and charge transfer contributions at B3LYP-D3­(BJ)/def2-TZVPP/CPCM­(DCM) level of theory as obtained from fragmenting [Ni’CH_2_]^+^ (blue bars) or [Pd’CH_2_]^+^ (violet bars) into adamantane (hatched bars) and its corresponding remainder, including the metal center, or into one of the HPCy_2_ ligands and resulting remainder (nonhatched bars). The frozen energy is further decomposed into permanent electrostatics, Pauli repulsion and dispersion on the left, whereas the charge transfer (CT) energy is separated into forward (CTf) and backward (CTb) energies on the right. For the latter, direction of charge flow is shown at the example of [M’CH_2_]^+^ (b) Decomposition of the *change* in interaction energy (ΔΔ*E*_INT_) when going from [Ni’CH_2_]^+^ to [Pd’CH_2_]^+^. Negative energy values correspond to greater stabilization of that energy component in [Pd’CH_2_]^+^ compared to [Ni’CH_2_]^+^. The most important contribution, Δ*E*_CT_, is highlighted by a red box. (c) Key complementary occupied-virtual pairs (COVPs) representing the bidirectional CT between the adamantane and [Pd] units in [Pd’CH_2_]^+^. Occupied donor orbitals are shown in red and blue color and overlap with empty acceptor orbitals in yellow and green. The significance of these orbital interactions is reflected in the contribution to the associated unidirectional and total CT energies, here given in % of the respective total amount.

Figure 8a shows the ALMO-EDA results for both [Ni’CH_2_]^+^ and [Pd’CH_2_]^+^ at the B3LYP- D4/def2-TZVPP/CPCM­(DCM) level of theory. Although this level of theory was not among the ones tested on our small equilibrium data benchmark, we nevertheless employ it for the bonding analysis herein due to its proven robustness in previous ALMO-EDA studies.^103–106^ In fact, we have re-evaluated all energy components at yet another level of theory to test the transferability of bonding insights and only report consistent trends observed at both levels of DFT (see Tables S24 and S25).

As already reported for coinage metal π-alkyne complexes,^106^ characteristic changes in the EDA energy profile can be observed when moving down the periodic table within one group. Hence, similar to coinage metal π-alkyne complexes, we obtain significantly higher Pauli repulsion in [Pd’CH_2_]^+^ due to increased unfavorable orbital overlap that is not fully compensated by purely permanent electrostatic interactions, requiring further stabilizing contributions. Interestingly, Δ*E*_POL_ is not one of those stabilizing contributions and [Ni’CH_2_]^+^ is actually favored over [Pd’CH_2_]^+^. Further, in accordance with local energy decomposition analysis results by Bistoni and co-workers,^107^ dispersive interactions are significant, albeit both complexes experience similar amounts of stabilization, which leaves only one stabilizing contribution to remedy increased Pauli repulsion: the charge transfer energy (Δ*E*_CT_). While Pauli repulsion is minimized by increasing bond lengths, charge transfer (CT) behaves inversely, resulting in an apparent interplay of Pauli repulsion and charge transfer. This behavior is not unique to Pd and Ni, and is also found to be structurally determining for other molecular examples (see Table S28 for selected bond lengths of the calculated structures).^101,106,108^

To gain more understanding of the specific orbital interactions, variational complementary occupied-virtual (orbital) pairs (COVPs)^109^ were determined for [Ni’CH_2_]^+^ and [Pd’CH_2_]^+^ (see Section VIII of the Supporting Information). COVPs are representations of the key donor-acceptor orbital pairs that contribute to the total CT energy, which can be quantified and thus critically compared between structures. Both [Ni’CH_2_]^+^ and [Pd’CH_2_]^+^ yield key COVPs that are very comparable, featuring a strong σ-type interaction of an occupied σ­(CH) MO with the empty *d*_*z*2_ metal valence orbital in the case of the forward CT interaction (Figure 8c), and a π interaction between the filled metal d_*xz*_ orbital and the empty σ*­(CH) MO in the backward direction (Figure 8d). Hence, the COVPs successfully recover the hyperconjugative frontier MO interactions characteristic for typical agostic structural motifs,^110^ reinforcing the classical Dewar–Chatt–Duncanson model.^111,112^

Figure 8b shows the relative difference in interaction energy contributions (ΔΔ*E*) when going from [Ni’CH_2_]^+^ to [Pd’CH_2_]^+^, thus indicating increased stabilization for Pd in the case of negative energy values. This clearly indicates that most of the increased CT stabilization stems from electron donation from adamantane to the Pd fragment (CTf), whereas only minor changes in metal–ligand backbonding (CTb) occur, which is in line with negligible changes in computed bond lengths going from Ni to Pd (see Table S28). Nevertheless, metal backbonding to one C–H moiety is still relevant as the agostic interaction is not fully symmetric, leading to one M–H bond being significantly longer in both metal complexes. Finally, given that most of the (increased) CTf energy in [Pd’CH_2_]^+^ is determined by a single orbital pair (Figure 8c, 84% of the CTf energy), the difference in bonding can also be mainly traced back to this single interaction, which we hypothesize is drastically different due to a change in orbital energies based on the observation that some metal valence orbital energies tend to lower when moving down the periodic table due to lower *d–d* repulsion.^113^ Further, a significantly larger CTf to CTb ratio places [Pd’CH_2_]^+^ on the strongly electrophilic side of the C–H bond activation reactivity spectrum as proposed by Goddard and co-workers.^17,114,115^ Gratifyingly, this high electrophilicity of the coordinated metal toward the agostic [CH_2_] ligand is highly consistent with the observed increase in C–H bond acidity, analogous to how electron-withdrawing groups in typical organic acids affect C–H bond polarization through inductive effects.

### Possible Implications for Catalysis

After establishing a thorough understanding of the thermochemical data for these isostructural Ni and Pd complexes, we were eager to probe the veracity of C–H bond acidification in molecular systems capable of C­(sp^3^)–H functionalization catalysis. However, despite extensive research, the field still lacks well-documented examples of homogeneous Ni and Pd systems that undergo catalytic C­(sp^3^)–H functionalization under identical reaction conditions (solvent, temperature, additives, base, etc.), hampering our ability to solely utilize experimental data for probing the effect of metal substitution on C–H bond acidifications. Further, as mentioned earlier, it is challenging to disentangle the effects of simultaneous C­(sp^3^)–H bond cleavage and O–H bond formation from catalytic systems that undergo CMD-type pathways, as internal bases will have intrinsically different M–O bond strengths that could modulate their effective basicity when interacting with the acidified C–H bond.

To nevertheless provide some possible implications for catalysis, we chose to investigate a Pd catalyst system whose C–H activation mechanism has been studied in detail and supplement the data sets with DFT calculations on the isostructural Ni complexes. The catalyst used in this case study features a bidentate acetyl-protected aminoquinoline (APAQ) ligand, reported by Yu and Houk,^19,20^ which undergoes an intramolecular deprotonation and O–H bond formation through a putative agostic C­(sp^3^)–H intermediate ([Int3-M], Figure 9a). This allows for a direct comparison of metal-induced changes in reactivity based on computational data obtained from the analogous Ni complex. For this, the previously established reaction coordinate for Pd^19^ was systematically reconstructed by replacing the metal center with Ni in the reported structural data followed by subsequent application of our computational workflows as described in Section II of the Supporting Information, thus, including a thorough conformational analysis. As expected, we observed significant structural differences in the final geometries between Ni and Pd, which would further complicate direct comparison of the two potential energy surfaces. Hence, we opted for consistently adhering to the lowest-energy geometries as obtained by replacing Ni in the conformationally explored Pd geometries, while only allowing for minor bond length relaxation in the corresponding Ni complexes (see Section IX of part B of the Supporting Information for a detailed discussion of all relative energies). We must emphasize here, that a complete neglect of conformational exploration, as was the case with the reported structures by Houk et al.,^19^ introduces a significant energy discrepancy of 5.4 kcal/mol for **[Int3-Pd]** and 6.1 kcal/mol for **[Int3-Ni]**. This difference in relative energy corresponds to an κ^2^-CH_2_ to κ^3^-CH_2_ rearrangement of the agostic interaction (Figure 9b), for which the κ^3^-CH_2_ motif has been previously reported to be lower in energy, thus, further confirming our computational observations.^107,116^

**Figure 9:**
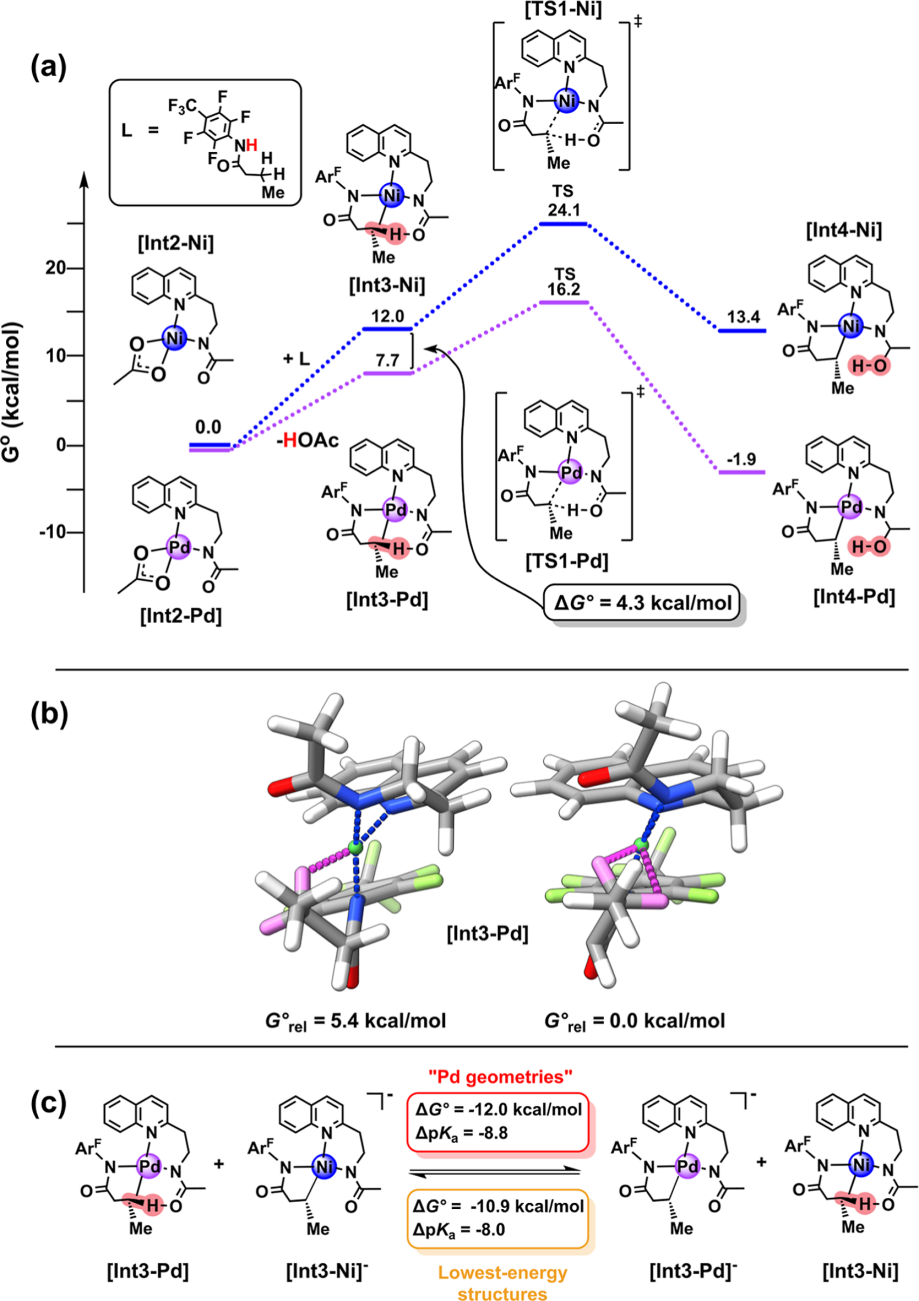
(a) Partial free energy profile comparing C–H activation between the experimentally known Pd­(APAQ) catalyst^19^ and a hypothetical Ni-based analogue in hexafluoroisopropanol (HFIP), showing the difference in ground state free energy (Δ*G*°) at the PBE0-D4/def-QZVPP/COSMO-RS­(fine, HFIP) level of theory for generating a transient C­(sp^3^)–H agostic intermediate. Compound labels used herein are identical to those used in the original manuscript. (b) Comparison of the previously reported structure of **[Int3-Pd]** (left) with the lowest energy structure as obtained in this study (right). Relative energies are given at the same level of theory as in (a) and the agostic interactions as well as the CH_2_ group of interest are colored in violet. (c) Isodesmic reaction equilibrium in HFIP, showing the difference in standard-state free energy (Δ*G*°) and acidity (Δp*K*_a_) between **[Int3-Ni]** and **[Int3-Pd]** at the PBE0-D4/ma-def-QZVPP/COSMO-RS­(fine, HFIP) level of theory for both consistent “Pd geometries” (red, top, see main text for explanations) or using the lowest-energy structures (orange, bottom).

While all relative free energies are lower in the Pd pathway, the 4.3 kcal/mol difference in standard-state free energy for generating the agostic intermediates **[Int3-Ni]** and **[Int3-Pd]** suggests that that ground state destabilization of the agostic Ni complex could result in differences in acidification in the reaction medium (hexafluoroisopropanol). However, the significant electronic reorganizations accompanying intramolecular O–H bond formation along the reaction coordinate in Figure 9a make it challenging to disentangle the differences in C–H bond acidities on going from **[Int3]** to the final products. To obtain more quantitative insights on the acidification of this specific agostic C–H bond, we computed the difference in acidity for **[Int3-Ni]** and **[Int3-Pd]** in the isodesmic reaction shown in Figure 9c, again consistently employing “Pd geometries”. The resulting shift in intrinsic acidification of approximately 8.8 p*K*_a_ units when going from Ni to Pd thus qualitatively aligns with the observed ground state destabilization. Finally, we note that the “Pd geometries” are not the lowest-energy structures for the isodesmic scheme in Figure 9c. When ultimately using the lowest-energy structures, the intrinsic acidification is corrected to a smaller shift of 8.0 p*K*_a_ units, being slightly closer to the experimental acidification regime as reported in this study. Moreover, this observed difference in ground-state thermochemistry (p*K*_a_) may also apply to changes in transition state barrier heights and thus kinetic analyses, and an exemplary system undergoing CMD using an 8-aminoquinoline amide directing group has been similarly analyzed (see Figures S74 and S75 and the discussion thereof in part B of the Supporting Information).

## Conclusions

Understanding the mechanistic minutiae of metal-catalyzed C–H bond activation remains crucial for selective alkane conversion, functionalizing complex organic molecules, and developing new advanced materials. Before this study, a systematic and quantitative comparison of agostic C–H bond thermochemistries as a function of transition metal remained elusive. Consequently, this work provides key experimental insights into coordination-induced C–H bond weakening at Pd by measuring the C–H bond acidity (p*K*_a_) and dissociation free energy (BDFE) under standard-state conditions, enabling direct comparison with an isostructural Ni system. An incredibly rare agostic R_2_CH_2_···PdBr complex was structurally authenticated after acidification of an adamantyl-derived pincer complex, and its complicated acid–base chemistry was probed in solution. Spectral analysis of the solution-phase equilibria uncovered the formation of a bromide-bridged bimetallic Pd complex and base-stabilized bromonium ion, with variable temperature NMR spectroscopy enabling the extraction of thermochemical data via Van’t Hoff analysis. Extensive conformational efforts as part of an efficient density functional theory-based multilevel workflow provided detailed insights into the solution phase structures of the investigated species, also shedding light on the solution phase dynamics of both the agostic R_2_CH_2_···PdBr complex as well as the conformationally flexible bimetallic Pd complex. The most notable outcome is the 100,000-fold acidification of agostic C–H bonds at Pd (p*K*_a_^THF^ = −0.3) when compared to Ni (p*K*_a_^THF^ = 4.2)^32^—results that are wholly consistent with enhanced electrophilic character of [Pd] as determined via ALMO-EDA analysis and theoretical free energy landscapes involving C–H bond activation during catalysis.

To understand if the magnitude of these C–H bond weakening phenomena are broadly true for other Ni and Pd systems, future work will probe the dependence of the ancillary ligand environment on the observed C–H thermochemistry and whether other (strong) acids are chemically compatible for measuring acid–base equilibria. Further investigations, both experimental and computational, are also needed to understand whether these trends extend to the 5d metals (i.e., Pt), other *d*-block triads that participate in C­(sp^3^)–H bond functionalization catalysis (i.e., Groups 8–9), and agostic interactions involving unsaturated C­(sp^2^)–H bonds. We hope that the results of this study assist with the design of new ligand frameworks and reaction conditions that accelerate the development of selective and more sustainable C–H bond functionalization methodologies.

## Supplementary Material





## Notes

Syntheses, spectra, additional figures and schemes, computational details (PDF)
Computed structures and results of the functional benchmark (ZIP)
