# Supplementary material for: A 100,000-Fold Increase in C–H Bond Acidity Gives Palladium a Key Advantage in C(sp3)–H Activation Compared to Nickel
Source: J Am Chem Soc. 2025 Sep 9;147(38):34395–410. doi: 10.1021/jacs.5c07649 (PMC12464984; doi:10.1021/jacs.5c07649)
Supplement: Supplementary file 1 [file ja5c07649_si_001.pdf]

Supporting Information for:

A 100,000-fold Increase in C-H Bond Acidity Gives Palladium a Key Advantage in C(sp^3)-H Activation Compared to Nickel

Lirong Lin,[a] Tim K. Schramm,[b] Pavel Kucheryavy,[a] Roger A. Lalancette,[a] Andreas Hansen,[b] and Demyan E. Prokopchuk[a*]*

[a]Department of Chemistry, Rutgers University-Newark, Newark, New Jersey 07102, United States. *demyan.prokopchuk@rutgers.edu

[b]Mulliken Center for Theoretical Chemistry, Clausius Institute for Physical and Theoretical Chemistry, Rhenish Friedrich Wilhelms University of Bonn, Bonn 53115, Germany. *hansen@thch.uni-bonn.de

Part A

Experimental

Contents

General Comments **4**

Syntheses **6**

[PdBr] 6

[Pd-Br-Pd]$^+$ 7

Protonation of [PdBr] with $H(Et_2O)_2[B(C_6F_5)_4]$ 7

$[H_3NAr^{Cl}][B(C_6F_5)_4]$ 8

$[H_3NAr^{F}][B(C_6F_5)_4]$ 8

VT-NMR and Kinetic Analysis of [Pd-Br-Pd]$^+$ **10**

Acid-Base Reactions **12**

$[H(OEt_2)_2][B(C_6F_5)_4]$ 12

Van't Hoff analysis 12

EXSY Analysis 16

Addition of 2,4-dinitroaniline to $[PdCH_2{\bullet}Et_2O]^+$ 17

$[H_3NAr^{F}][B(C_6F_5)_4]$ 17

Van't Hoff analysis (CD_2Cl_2) 18

EXSY analysis (CD_2Cl_2) 20

$[H_3NAr^{Cl}][B(C_6F_5)_4]$ 21

Van't Hoff analysis (DCE) 21

Van't Hoff analysis (CD2Cl2) 24

EXSY analysis (CD_2Cl_2) 26

NMR Spectra **27**

DOSY NMR and External Calibration Curve Analysis **45**

Electrochemistry **48**

X-Ray Crystallographic Structures **52**

References **52**

General Comments

All reactions were carried out under an atmosphere of nitrogen using standard glove box or high vacuum line (Schlenk) techniques unless stated otherwise. All reagents and solvents were stored under inert gas unless stated otherwise. Inert atmosphere reactions and workup protocols used HPLC-grade, inhibitor-free solvents were dried and degassed over activated alumina using an IT/Inert solvent purification system. Additionally, 1,4-dioxane, *n*-pentane, tetrahydrofuran (THF), fluorobenzene (PhF), diethyl ether (Et_2O), toluene, acetonitrile (MeCN), 1,2-dichloroethane (DCE) and dichloromethane (DCM) were dried over 10% w/v activated 3 Å molecular sieves.[1] Deuterated solvents were subjected to three freeze-pump-thaw cycles and dried over 10% w/v activated 3 Å molecular sieves in a glove box. Glassware was dried overnight at 140 °C and cooled under dynamic vacuum in a glove box antechamber. Celite and glass fiber filter circles (1.6 μm) were dried in an oven at 140 °C overnight prior to use in the glove box. Column chromatography was performed using silica gel (60 Å porosity, 40-63 μm particle size). Elemental analyses were run by CENTC Elemental Analysis Facility (Department of Chemistry, University of Rochester) on a PerkinElmer 2400 Series II Analyzer. Compounds **PCH_2P**[2, 3] and $[H(OEt_2)_2][B(C_6F_5)_4]$[4] (DCl ($Et_2O$) instead of HCl ($Et_2O$) was used for $[D(OEt_2)_2][B(C_6F_5)_4]$) were prepared using known literature procedures while the known complex **[PdBr]**[3] was isolated using a modified workup protocol as described later. $[^nBu_4N][B(C_6F_5)_4]$ was prepared using a known procedure[5] but using $KB(C_6F_5)_4$ as the anion source and recrystallizing via vapor diffusion from fluorobenzene/pentane. To minimize cross-contamination with ethereal solvent vapors inside the glove box for all acid-base reactions, solvents were stored in Teflon-sealed Schlenk vessels when not in use.

NMR Spectroscopy. Experiments were conducted on Bruker Avance III HD 500 MHz NMR and Varian Inova 600 MHz NMR spectrometers. Spectra for ^1H and ^{13}C were referenced to their respective residual protio solvent signal,[6] ^{31}P to external 85% H_3PO_4 (0 ppm), ^{19}F to external $C_6H_5CF_3$ (-63.72 ppm). NMR signal assignments were made by routine one- and two-dimensional experiments provided by Bruker and Varian, including ^1H-^1H COSY, ^1H-^1H NOESY, ^1H-^{13}C HSQC, ^1H-^{13}C HMBC, ^1H-^{31}P HMBC (optimized for ^1J(PH) = 200 Hz, long range J(PH) = 8 Hz). All NMR measurements were carried out at 25 °C unless otherwise stated. For Van't Hoff Analyses from VT-NMR data, S/N (Signal to Noise) ratios were calculated for each signal in Topspin by using the "SINo" function. The relative error for peak integrals is 1/(S/N). For VT-NMR experiments, the probe temperature was calibrated using Sigma-Aldrich methanol-d4 NMR reference standard.

DOSY NMR experiments for constructing external calibration curves (ECCs) were conducted on Bruker Avance III HD 500 MHz spectrometer using the dstebpgp3s pulse program with 5 s relaxation delay (d1), and 0.1 s diffusion delay (d20). Duration of the gradient delay (p30) was optimized for each individual compound in the range between 500 and 900 μs using the dstebpgp3s1d pulse program. For the equilibrium measeuments, p30 was set to 900 μs. All data was processed in Mnova 15.1. For the ECC, each molecule was dissolved on CH_2Cl_2 with a concentration of 1 mg/mL. Low analyte concentrations were required to minimize ion pairing in solution. For equilibria K_2 and K_3, two independently prepared samples contained 1.0 mg **[PdBr]** and 1.1 mg acid (**$[H_3NAr^F][B(C_6F_5)_4]$** for K_2 and **$[H_3NAr^{Cl}][B(C_6F_5)_4]$** for K_3) dissolved in 1.0 mL CD_2Cl_2 were used to measure diffusion coefficients.

Variable temperature ^{31}P NMR experiments with inverse gated decoupling were performed on a 500 MHz Avance III HD NMR spectrometer in the range between -40 and 70 °C in PhF and between -70

and 25 °C in CD_2Cl_2. Spectra were collected in 10 °C intervals. NMR spectra were fitted by trial and error using the DNMR (dynamic NMR)[7] package in Topspin. The exchange rate constant for each modeled line broadening was used to generate an Eyring plot, providing thermodynamic information about the rotation barrier along the Pd-Br-Pd axis in **[Pd-Br-Pd]**$^+$.

Error Propagation for Acid-Base Reactions. For VT-NMR equilibrium experiments, the S/N for all integrals was determined using the "SINo" function as described above. Sample masses were weighed using an analytical balance inside a glove box with a readability of 0.1 mg and error of ± 0.1 mg for each recorded mass. Solvent volumes for NMR samples were measured using standard 1 mL syringes (HSW Norm-Ject) with an estimated error of ± 0.02 mL. For equilibrium measurements and thermochemical calculations, standard error propagation equations were applied to all equations. In many cases, the largest contributor to the error at elevated temperatures in ^{31}P spectra was for **[Pd-Br-Pd]**$^+$, which shows broad signals, resulting in low S/N (Signal to Noise) ratios. For ^{1}H-^{1}H EXSY NMR experiments, noise was integrated to match the size of the peak with the largest dimension. S/N ratios were calculated for each signal for the error calculations.

X-Ray Crystallography. Single crystals of **[PdBr]**, **[Pd-Br-Pd]**$^+$, **[PdCH$_2$]**$^+$ and **[H$_3$NAr^{Cl}][B(C$_6$F$_5$)$_4$]** were selected and mounted using Paratone onto a nylon fiber and cooled to the data collection temperature of 100(2) K with a stream of dry nitrogen gas. X-ray diffraction intensities were collected on a Bruker SMART APEX II CCD Diffractometer using CuKα (1.54178 Å) radiation. For all structures, the CheckCIF routine and structure factor analyses were performed by Platon[8] via https://checkcif.iucr.org/. Graphics were generated using Mercury. All crystallographic data has been deposited with the Cambridge Crystallographic Data Center and is available free of charge through the CCDC online database (https://www.ccdc.cam.ac.uk/).

Electrochemistry. Cyclic voltammetry experiments were conducted under N_2 at 295 ± 3 K using a standard three-electrode setup consisting of a 1 mm PEEK-encased glassy carbon working electrode (eDAQ), graphite rod counter electrode, and Ag wire pseudoreference electrode. The working electrode was polished with 0.25 µm diamond polishing paste, lapping oil, and a rayon microcloth pad (Buehler) inside a glove box and thoroughly rinsed with the solvent used in the corresponding experiment. A Gamry Reference 620 potentiostat, Gamry 1010 potentiostat and Gamry software were used for data collection and analysis. Unless stated otherwise, samples contained 0.2 M [^{n}Bu$_4$N][B(C$_6$F$_5$)$_4$], organic solvent (1.5 mL), and 1 mM analyte. The uncompensated solution resistance (R_u) was measured for each electrochemical solution and post-iR correction (positive feedback) was applied to all CV traces. All CVs are referenced to the $Fc^{+/0}$ redox couple (0 V).

Syntheses

[PdBr]

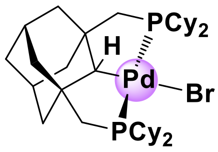

This known complex[3] was purified using a modified protocol. In the glovebox, $PdBr_2$(COD) (374 mg, 1.0 equiv., 1.0 mmol) and K_3PO_4 (254 mg, 1.2 equiv., 1.2 mmol) were suspended in 100 mL dioxane in a thick-walled glass pressure tube followed by the addition of the known 1,3-bis(dicyclohexylmethylphosphino) adamantane ligand **PCH_2P** (612 mg, 1.1 equiv., 1.1 mmol). The tube was sealed and heated at 100 ºC in the fume hood for three hours. Then, the reaction vessel was cooled to room temperature and brought into the glovebox. Next, 100 mL Et_2O was added to the mixture and the solution was filtered through a 60 mL medium-pore glass frit equipped with a pad of Celite. The filtrate was dried and extracted with a mixture of Et_2O:pentane (1:1, 30 mL x 2) to obtain an orange liquid, which was tested by TLC (thin layer chromatography) using 5:1 hexane:Et_2O as the mobile phase under air (Fig. S1, left). Column chromatography was conducted under air with a glass column (⌀ = 2 cm) with a medium-pore glass frit and packed with approximately 17 cm of silica. The column was flushed using a gradient elution of hexane:Et_2O (15:1; 9:1; 5:1; 1:1, 100 mL for each solvent mixture). The product began eluting from the column after collecting approximately 150 mL of yellow liquid, which was indicated by TLC (hexane: Et_2O = 5:1) (Fig. S1, middle). After drying the nearly pure fraction, the yellow solid (180 mg, 24%) was suspended and stirred in 1.0 mL Et_2O for two hours. The analytically pure pale-yellow product (Fig. S1, right) was obtained by isolation on a medium-pore glass frit and the solid was then dried under high vacuum overnight at 80 °C before taking into the glove box (126 mg, 17%). X-ray-quality crystals of **[PdBr]** were obtained by a concentrated pentane or $CHCl_3$ solution at room temperature and NMR matches previously reported spectral data[3]. Attempts to improve the yield by changing the starting Pd source, reaction solvent, or base were unsuccessful.

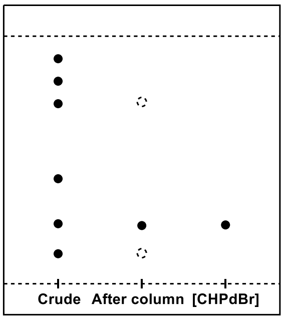

Figure S1- TLC (Hexane: Et_2O = 5:1) of the crude product before column chromatography (left), after column chromatography (middle), and the analytically pure product **[PdBr]** after washing with Et_2O (right).

$[Pd\text{-}Br\text{-}Pd]^+$

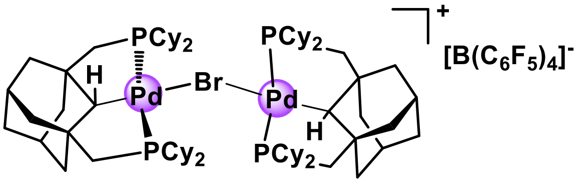

In the glovebox, **[PdBr]** (44.4 mg, 0.060 mmol) and $K[B(C_6F_5)_4]$ (21.6 mg, 0.030 mmol) were added in a 20 ml vial followed by 3.0 mL DCM. The reaction turned light orange immediately. After stirring overnight at room temperature, the reaction mixture was pipetted through a glass fiber filter plug to remove the white precipitate and obtain an orange filtrate. The crude product was evaporated to dryness and rinsed with pentane (2.0 mL x 3). Then, 2.0 mL Et_2O was added and the solution was stirred for 10 min. The pale-yellow solid was isolated on a medium-pore glass frit and washed with Et_2O (2.0 mL), washing away a brown impurity. The resulting pale-yellow product (50 mg, 80%) was obtained after drying under high vacuum. Anal. calcd (%) for $C_{96}H_{122}Pd_2P_4BBrF_{20}$: C 55.34, H 5.90, N 0.00; found: C 54.87, H 5.70, N 0.03.

^{1}H NMR (500 MHz, CD_2Cl_2, 25 ºC): δ 3.28 (s, br, 2H, C*H*Pd), 2.28-0.94 (120H). ^{31}P{^{1}H} NMR (202 MHz, CD_2Cl_2, 25 ºC): δ 55.0 (br), 50.8 (br) ppm. ^{31}P{^{1}H} NMR (202 MHz, CD_2Cl_2, -60 ºC): δ 56.0 (d, $^1J_{PP}$ = 318.59 Hz), 48.8 (d, $^1J_{PP}$ = 318.58 Hz) ppm. ^{13}C NMR (151 MHz, CD_2Cl_2, 25 ºC) δ 149.51 ($B(C_6F_5)_4$), 147.95 ($B(C_6F_5)_4$), 139.63 ($B(C_6F_5)_4$), 137.69 ($B(C_6F_5)_4$), 136.06 ($B(C_6F_5)_4$), 124.67 (br, $B(C_6F_5)_4$), 80.34 (*C*H-Pd), 47.49, 46.10, 44.49, 36.43, 35.77, 31.35, 30.38, 29.62, 28.08, 27.56, 27.36, 26.73, 26.55 ppm.

Protonation of [PdBr] with $H(Et_2O)_2[B(C_6F_5)_4]$

In the glovebox, **[PdBr]** (14.8 mg, 0.020 mmol) was dissolved in 1.0 ml PhF in a Schlenk flask, sealed, then connected to a Schlenk line and cooled to -40 ºC. Next, $[H(OEt_2)_2][B(C_6F_5)_4]$ (16.6 mg, 0.020 mmol) dissolved in 1.0 mL PhF was added to the solution dropwise. After addition, the solution turned yellow and was stirred at -40 ºC for 2 min. The cold bath was removed and the reaction was stirred at room temperature for 20 mins. The color of the solution turned orange while warming to room temperature due to a change in concentration from primarily yellow **[Pd-Br-Pd]**$^+$ at lower temperature and increasing concentrations of orange **[PdCH$_2$]**$^+$ at room temperature (Fig S2). The solvent was removed under a high vacuum for three hours. X-ray-quality crystals containing a mixture of orange **[PdCH$_2$]**$^+$ and pale yellow **[Pd-Br-Pd]**$^+$ were obtained by layering a concentrated toluene solution with pentane at room temperature. Suitable crystals were hand-picked from these batches. From batch to batch, **[Pd-Br-Pd]**$^+$ crystallized as pale yellow needles, pale yellow rods, or pale yellow plates. Crystallization via toluene/Et_2O vapor diffusion at room temperature solely produced colorless crystals of **[Pd-Br-Pd]**$^+$, as excess Et_2O drives the reaction equilibrium towards formation of **[Pd-Br-Pd]**$^+$ in solution.

^{1}H NMR (500 MHz, CD_2Cl_2, 25 ºC): δ 16.31 (s, br, **[HBrH•2OEt$_2$]**$^+$), 4.18 (CH_2, Et_2O), 3.97 (CH_2, Et_2O), 3.30 (br, C*H*-Pd, **[Pd-Br-Pd]**$^+$), 2.27 (CH_{Ad}), 2.24 - 1.10 ppm (mixture of Et_2O, Ad, Cy resonances from **[PdCH$_2$]**$^+$ and **[Pd-Br-Pd]**$^+$. Some Cy and Ad resonances were identified via 2D NMR spectral analysis: 2.17 (CH_{Cy}-P), 2.05 (CH_2-P), 1.69 and 1.93 (CH_{2Ad}) ppm), 0.49 (br, 2H, CH_2-Pd, **[PdCH$_2$]**$^+$) ppm. ^{31}P{^{1}H} NMR (202 MHz, CD_2Cl_2, 25 ºC): δ 66.4 (s, **[PdCH$_2$]**$^+$), 55.2 (br, **[Pd-Br-Pd]**$^+$), 50.8 (br, **[Pd-Br-Pd]**$^+$) ppm. ^{31}P{^{1}H} NMR (202 MHz, CD_2Cl_2, -60 ºC): δ 67.0 (s, **[PdCH$_2$]**$^+$), 55.9 (d, $^2J_{PP}$ = 318.52 Hz, **[Pd-Br-Pd]**$^+$), 48.7 (d, $^2J_{PP}$ = 318.48 Hz, **[Pd-Br-Pd]**$^+$) ppm. ^{13}C NMR (126

MHz, CD_2Cl_2, 25 ºC) δ 163.29 (m, $^1J_{CF}$ = 244.8 Hz, ($B(C_6F_5)_4$), 148.59 (m, $^1J_{CF}$ = 241.6 Hz, $B(C_6F_5)_4$), 137.71 (m, $^1J_{CF}$ = 243.6 Hz, $B(C_6F_5)_4$), 124.52 (br, $B(C_6F_5)_4$), 69.98 (O*C*H_2), 58.51(t, *J* = 11.4 Hz, *C*H_2-Pd of **[PdCH₂]**$^+$), 46.00, 44.81 (t, *J* = 3.0 Hz, quaternary C_{Ad}), 43.24 (s, CH_{2Ad}), 42.36 (t, *J* = 7.5 Hz, P*C*H_2), 36.27, 35.56 (t, *J* = 11.0 Hz, PCH_{Cy}), 34.31, 31.24, 30.12, 29.90 (s, CH_{Ad}), 29.59, 29.45, 28.83, 27.97, 27.43, 27.21, 27.07, 26.93, 26.59, 26.41, 25.94, 14.20 (s, OCH_2CH_3).

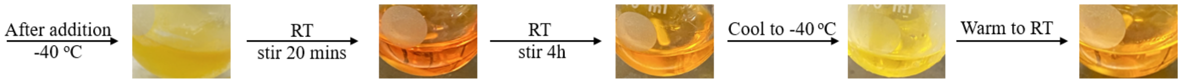

Figure S2- Color change upon addition of $[H(OEt_2)_2][B(C_6F_5)_4]$ to **[PdBr]** and the temperature-dependent color changes after protonation.

$[H_3NAr^{Cl}][B(C_6F_5)_4]$

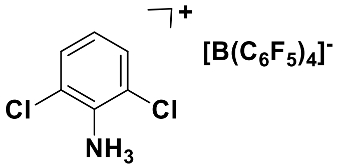

In the glovebox, 2,6-dichloroaniline (32.4 mg, 0.20 mmol) and $K[B(C_6F_5)_4]$ (143.6 mg, 0.20 mmol) were suspended in 10 ml DCM in a Schlenk flask, sealed, then connected to a schlenk line. While stirring under N_2, the reaction was bubbled with $HCl_{(g)}$ for 10 min using a PEEK tube pierced through a rubber septum (Fig. S3). The tube was then removed, and the reaction was stirred under N_2 for 10 min. The solvent was then removed under high vacuum to obtain a white solid and the schlenk flask was taken into a glove box. The analytically pure white product was extracted with DCM (10 mL), filtered through a glass fiber filter plug and dried under high vacuum (122 mg, 73%). X-ray-quality crystals of **$[H_3NAr^{Cl}][B(C_6F_5)_4]$** were obtained by crystallization via Toluene/pentane vapor diffusion at room temperature. Anal. calcd (%) for $C_{30}H_6NCl_2BF_{20}$: C 42.79, H 0.72, N 1.66; found: C 43.02, H 1.01, N 1.79.

^{1}H NMR (500 MHz, CD_2Cl_2): δ 7.58 (d, 2H, *m*-C*H*), 7.51 (t, 1H, *p*-C*H*), 7.09 (3H, N*H*$_3$) ppm. ^{19}F NMR (471 MHz, CD_2Cl_2, 25 °C): δ -133.23, -163.07 (t), -167.08 ppm.

$[H_3NAr^F][B(C_6F_5)_4]$

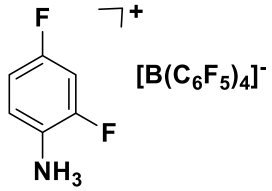

In the glovebox, 2,6-difluoroaniline (20 μL, 0.20 mmol) and $K[B(C_6F_5)_4]$ (144 mg, 0.20 mmol) were suspended in 10 ml DCM in a schlenk flask, sealed, then connected to a schlenk line. While stirring under N_2, the reaction was bubbled with HCl gas for 5 min (Fig. S3). The tube was then removed, and then the reaction was stirred under N_2 for 10 min. The solvent was then removed under high vacuum to obtain a white solid and the schlenk flask was taken into a glove box. The analytically pure white product was extracted with DCM (10 mL), filtered through a medium-pore glass frit and dried under

high vacuum (94 mg, 58%). Anal. calcd (%) for $C_{30}H_6F_{22}BN$: C 44.53, H 0.75, N 1.73; found: C 44.926, H 1.373, N 2.138.

^{1}H NMR (500 MHz, CD_2Cl_2): δ 8.52 (s, 3H, N*H*$_3$), 7.55 (m 1H), 7.16 (m, 1H), 7.10 (m, 1H) ppm. ^{19}F NMR (471 MHz, CD_2Cl_2, 25 °C): δ -102.38, -119.96, -133.24, -162.94, -167.03.

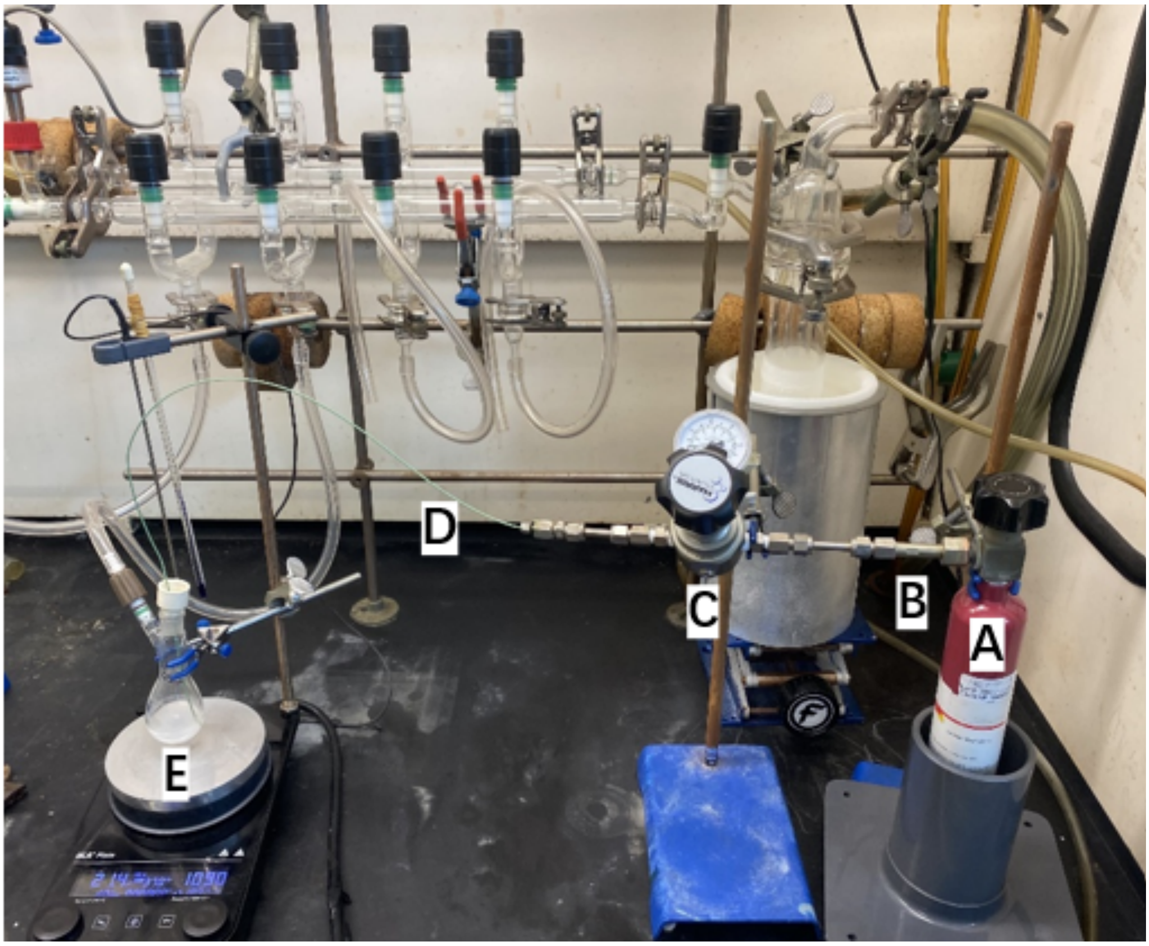

Figure S3- The $HCl_{(g)}$ addition setup. A lecture bottle containing $HCl_{(g)}$ (A) is connected via stainless steel compression fittings (B) to a corrosion resistant single-stage gas regulator (C; Harris HP743 Series, Model 743-050-000-C). The outlet on the low-pressure side contains polyether ether ketone (PEEK) tubing (D; 0.03" ID, 1/16" OD) attached via compression fitting to the stainless-steel outlet tube. The PEEK tubing is pierced directly into a pre-formed hole in a rubber septum connected to a Schlenk flask (E) under N_2.

VT-NMR and Kinetic Analysis of [Pd-Br-Pd]$^+$

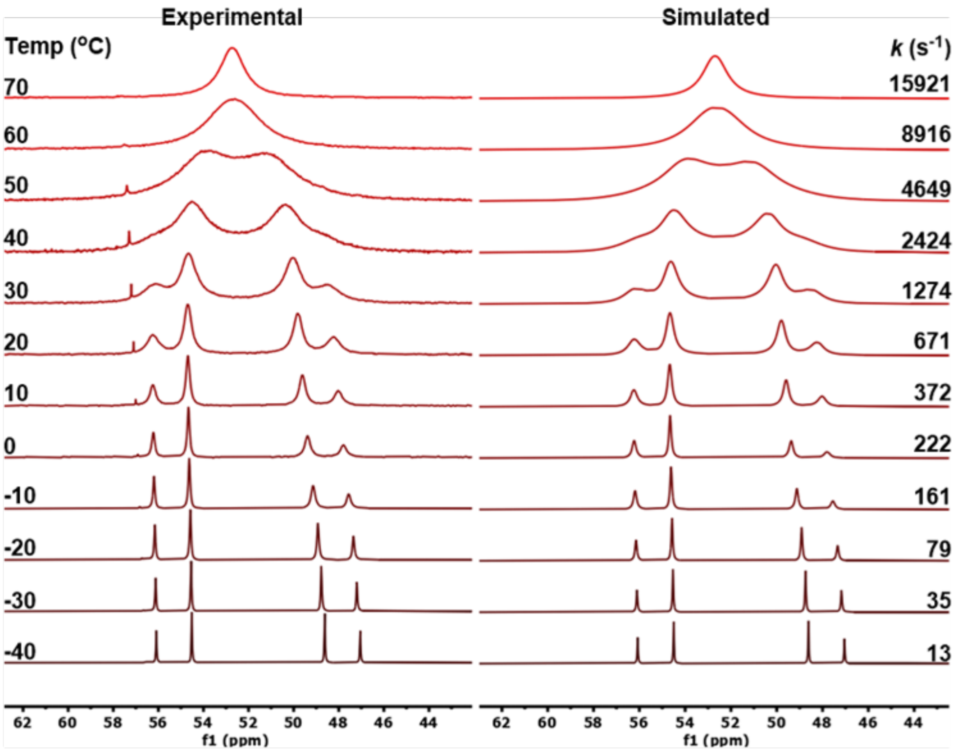

Figure S4- Experimental (left) VT ^{31}P NMR spectrum (202 MHz, PhF) of **[Pd-Br-Pd]**$^+$ and their calculated full lineshape fittings (dNMR Topspin utility) after least-square refinement at different temperature (right) of **[Pd-Br-Pd]**$^+$.

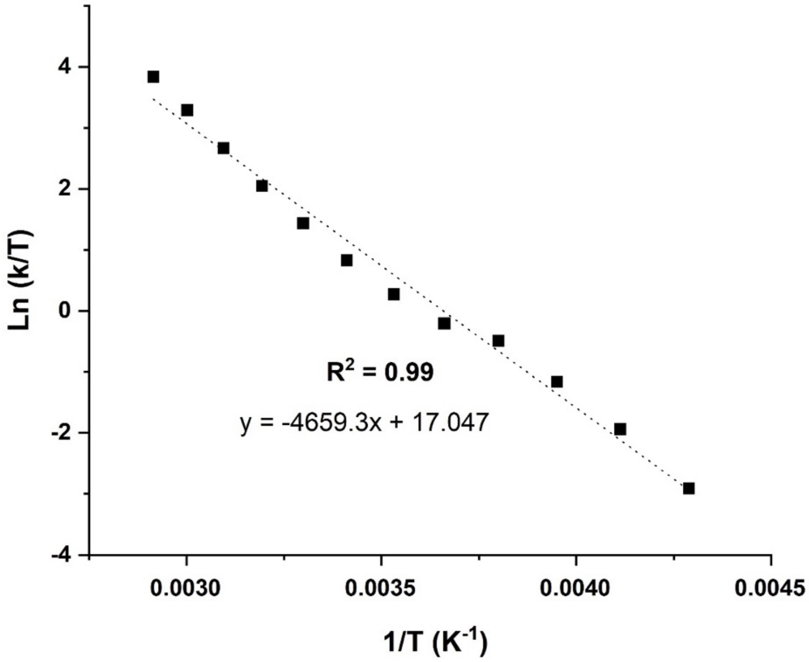

Figure S5- Eyring Plot for **[Pd-Br-Pd]**$^+$ in PhF, where $\Delta H^{\ddagger} = 9.3 \pm 0.3$ kcal/mol, $\Delta S^{\ddagger} = -13.4 \pm 0.5$ cal/(mol·K), and $\Delta G^{\ddagger}_{298K} = 13.3 \pm 0.3$ kcal/mol.

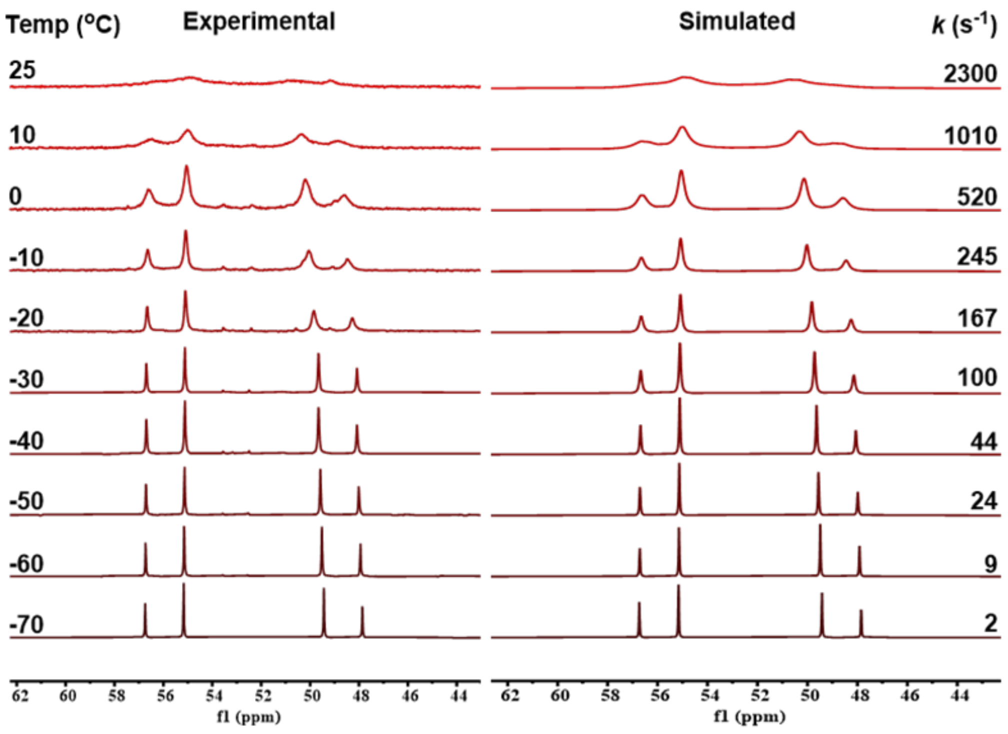

Figure S6- Experimental (left) VT ^{31}P NMR spectrum (202 MHz, CD_2Cl_2) of **[Pd-Br-Pd]**$^+$ and their calculated full lineshape fittings (dNMR Topspin utility) after least-square refinement at different temperature (right) of **[Pd-Br-Pd]**$^+$.

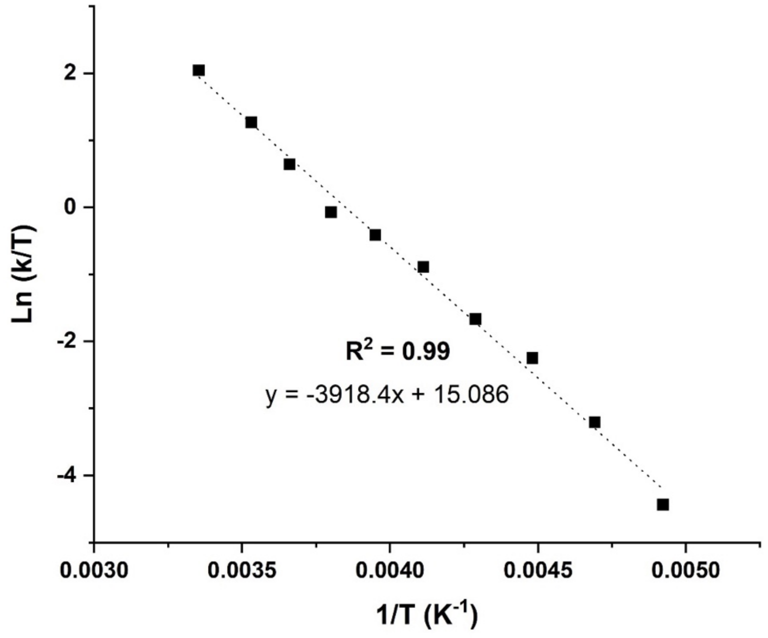

Figure S7- Eyring Plot for **[Pd-Br-Pd]**$^+$ in CD_2Cl_2, where $\Delta H^{\ddagger} = 7.8 \pm 0.2$ kcal/mol, $\Delta S^{\ddagger} = -17.3 \pm 0.9$ cal/(mol·K), and $\Delta G^{\ddagger}_{298K} = 13.0 \pm 0.4$ kcal/mol.

Acid-Base Reactions

$[H(OEt_2)_2][B(C_6F_5)_4]$

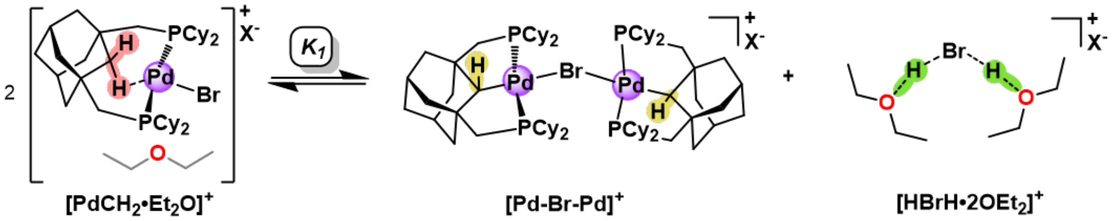

Scheme S1- Proposed equilibrium processed upon addition of $[H(OEt_2)_2][B(C_6F_5)_4]$ to **[PdBr]**. $X^- = B(C_6F_5)_4^-$.

Van't Hoff analysis

In the glovebox, **[PdBr]** (14.8 mg, 0.020 mmol) was dissolved in 1.0 ml PhF in a Schlenk flask, sealed, then connected to a Schlenk line and cooled to -40 ºC. Next, $[H(OEt_2)_2][B(C_6F_5)_4]$ (16.6 mg, 0.020 mmol) in 1.0 mL PhF was added to the solution dropwise. After addition, the solution was stirred at -40 ºC for 2 min. The cold bath was removed, and the reaction was stirred at room temperature for 20 min. The color of the solution turned into orange while warming to room temperature. The solvent was removed under high vacuum for 3 h. The residue was dissolved in CD_2Cl_2 (0.55 mL), which gives the initial concentration c_i as 36 ± 3.7% mmol/L. Then, $^{31}P\ \{^1H\}$ NMR spectra were collected from -70 ºC to 25 ºC in 10 ºC intervals (Fig. S10). The concentration of **$[PdCH_2•Et_2O]^+$** ($c(PdCH_2)$) and **$[Pd\text{-}Br\text{-}Pd]^+$** ($c$(Pd-Br-Pd)) were calculated using equation S1 and S2 with integrals $I(PdCH_2)$ and I(Pd-Br-Pd). The equilibrium constant K_1 was calculated at all temperatures using equation S3, assuming that the concentration of **$[Pd\text{-}Br\text{-}Pd]^+$** is equal to the concentration of **$[HBrH•2OEt_2]^+$**. With the data in Table S1, Van't Hoff plots were generated (Fig. S8-9, blue), allowing the calculation of the thermochemical values for the equilibrium (Table S2). An identical deuteration experiment was conducted with $[D(OEt_2)_2][B(C_6F_5)_4]$ (16.6 mg, 0.020 mmol) (Fig. S11) and the corresponding thermochemical values were calculated (Table S1-2) with Van't Hoff analysis (Fig. S8-9, red).

$$c(\text{PdCH}_2) = \frac{I(\text{PdCH}_2)}{0.5\ I(\text{Pd-Br-Pd}) +\ I(\text{PdCH}_2)} \times c_\text{i} \tag{S1}$$

$$c(\text{Pd-Br-Pd}) = \frac{0.5\ I(\text{Pd-Br-Pd})}{0.5\ I\ (\text{Pd-Br-Pd}) +\ I(\text{PdCH}_2)} \times c_\text{i} \tag{S2}$$

$$K_1 = \frac{c(\text{Pd-Br-Pd})^2}{c(\text{PdCH}_2)^2} \tag{S3}$$

Table S1- VT-NMR peak integration and K_1 data for **[PdCH$_2$•Et$_2$O]$^+$** and **[Pd-Br-Pd]$^+$** reacting with [H(OEt$_2$)$_2$][B(C$_6$F$_5$)$_4$] or [D(OEt$_2$)$_2$][B(C$_6$F$_5$)$_4$]. Integrals are measured relative to the ^{31}P resonance for **[PdCH$_2$•Et$_2$O]$^+$** which is set to exactly 1.

		H^+			D^+		
T(ºC)	*I*(PdCH$_2$)	*I*(Pd-Br-Pd)	K_1	Ln K_1	*I*(Pd-Br-Pd)	K_1	Ln K_1
25	1.00	1.38 (15)	0.48 (13)	-0.74 (28)	1.29 (14)	0.42 (11)	-0.88 (28)
10	1.00	1.79 (10)	0.80 (13)	-0.22 (16)	1.52 (7)	0.58 (8)	-0.55 (14)
0	1.00	1.87 (4)	0.87 (8)	-0.13 (9)	1.67 (4)	0.70 (7)	-0.36 (10)
-10	1.00	1.99 (7)	0.99 (11)	-0.01 (11)	1.78 (3)	0.79 (7)	-0.23 (9)
-20	1.00	2.09 (9)	1.09 (14)	0.09 (13)	1.89 (6)	0.89 (10)	-0.11 (8)
-30	1.00	2.20 (3)	1.21 (10)	0.19 (8)	1.92 (3)	0.92 (8)	-0.08 (8)
-40	1.00	2.31 (2)	1.33 (10)	0.20 (8)	1.97 (3)	0.97 (8)	-0.03 (8)
-50	1.00	2.36 (3)	1.39 (11)	0.33 (8)	1.96 (3)	0.96 (8)	-0.04 (8)
-60	1.00	2.29 (3)	1.31 (10)	0.27 (8)	1.98 (3)	0.98 (8)	-0.02 (8)
-70	1.00	2.26 (2)	1.28 (10)	0.24 (8)	1.95 (3)	0.95 (8)	-0.05 (8)

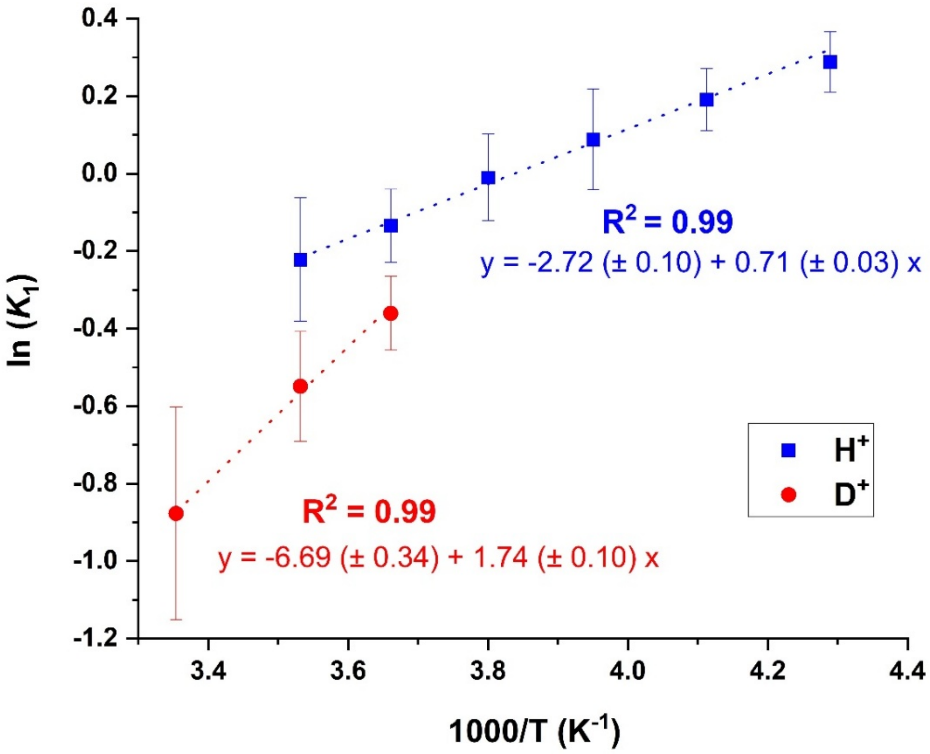

Figure S8- Linear region of Van't Hoff plot for the reaction with [H(OEt$_2$)$_2$][B(C$_6$F$_5$)$_4$].

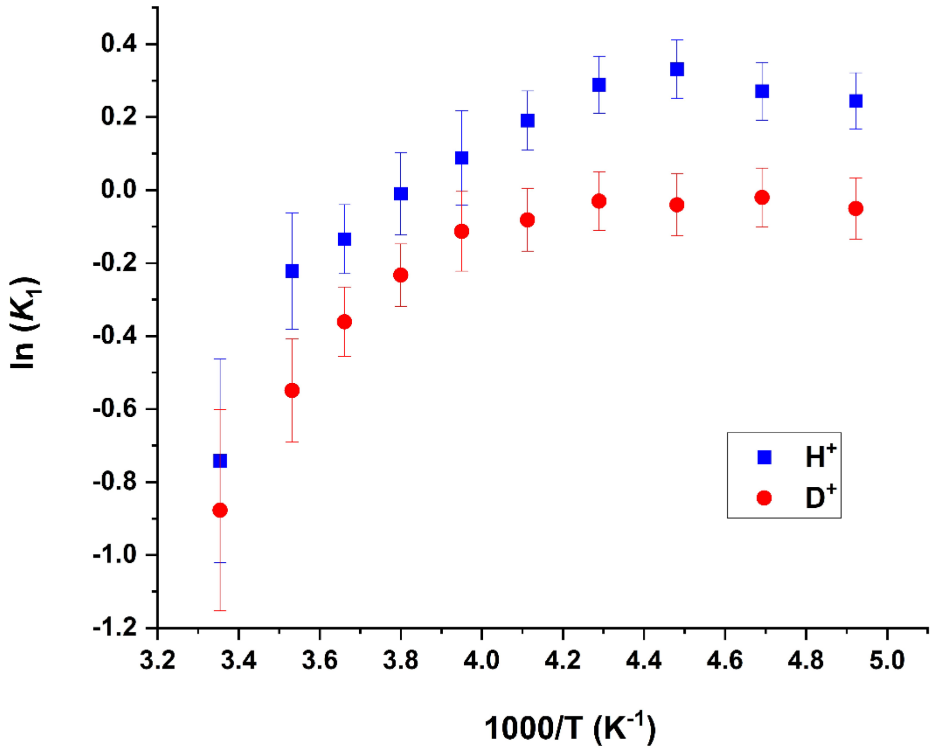

Figure S9- Full temperature profile of Van't Hoff plot for the reaction with $[H(OEt_2)_2][B(C_6F_5)_4]$ (blue) and $[D(OEt_2)_2][B(C_6F_5)_4]$ (red) in CD_2Cl_2. Equilibrium data at lower temperatures are likely unchanged due to slow exchange kinetics and were not included in the Van't Hoff analysis.

Table S2- Thermochemical data values for the equilibrium upon reaction with $[H(OEt_2)_2][B(C_6F_5)_4]$ and $[D(OEt_2)_2][B(C_6F_5)_4]$ in CD_2Cl_2.

	ΔH (kcal/mol)	ΔS (cal/(mol·K))	ΔG_{298K} (kcal/mol)
**H^+**	-1.4 ± 0.1	-5.4 ± 0.2	0.20 ± 0.08
**D^+**	-3.5 ± 0.2	-13.4 ± 0.7	0.52 ± 0.29

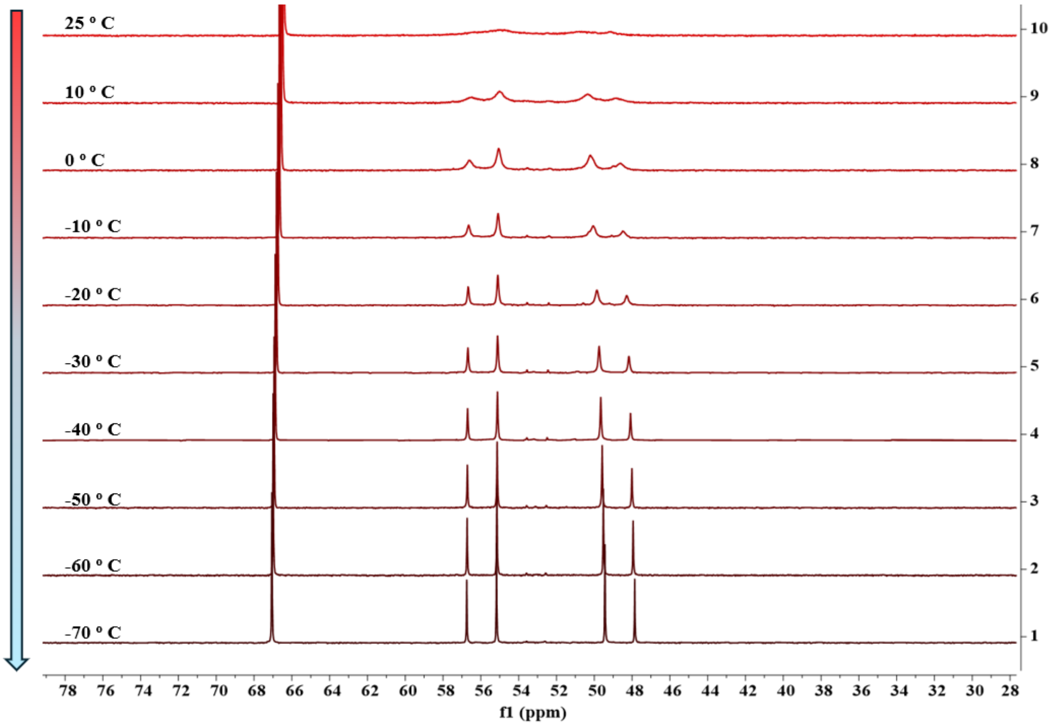

Figure S10- Variable temperature inverse-gated ^{31}P NMR spectra of the equilibrium mixture after reaction of **[PdBr]** with $H(Et_2O)_2[B(C_6F_5)_4]$ (202 MHz, CD_2Cl_2).

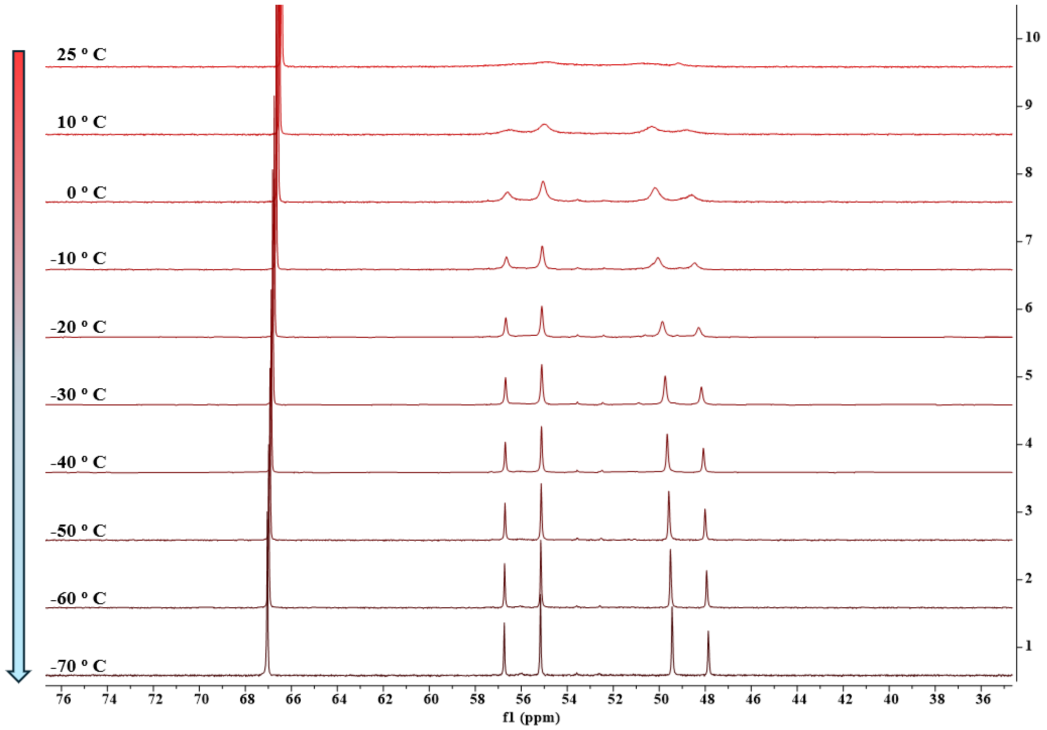

Figure S11- Variable temperature inverse-gated ^{31}P NMR spectra of the equilibrium mixture after reaction of **[PdBr]** with $D(Et_2O)_2[B(C_6F_5)_4]$ (202 MHz, CD_2Cl_2).

EXSY Analysis

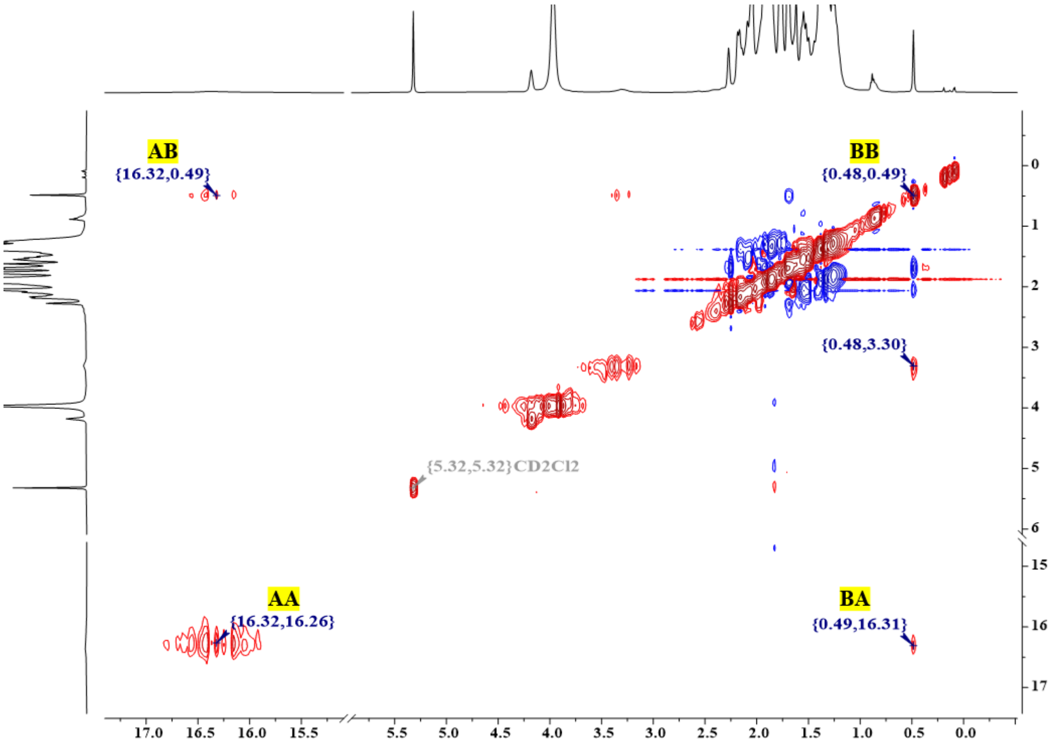

Figure S12- ^{1}H-^{1}H EXSY (red) – NOESY (blue) NMR spectrum of the equilibrium mixture after protonation of **[PdBr]** with $H(Et_2O)_2[B(C_6F_5)_4]$ (500, 500 MHz, CD_2Cl_2, 25 °C, τ_m = 0.5 s, NS = 64).

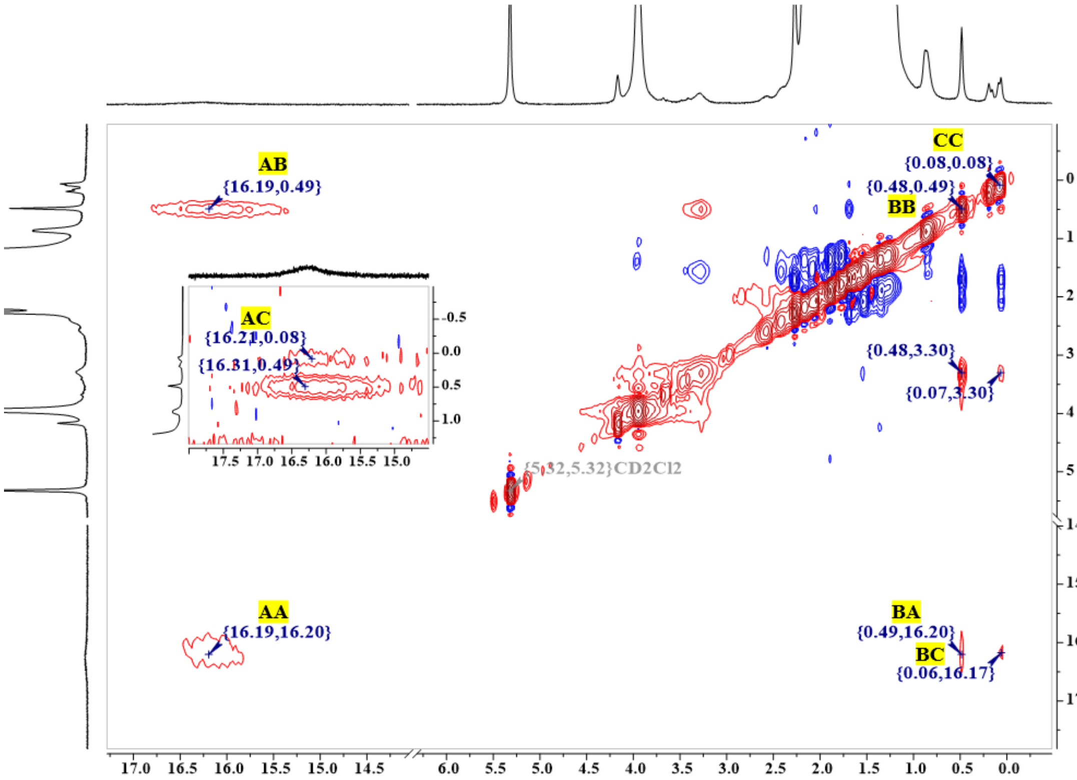

Figure S13- ^{1}H-^{1}H EXSY (red) – NOESY (blue) NMR spectrum of the equilibrium mixture after deuteration of **[PdBr]** with $D(Et_2O)_2[B(C_6F_5)_4]$ (500, 500 MHz, CD_2Cl_2, 25 °C, τ_m = 0.5 s, NS = 64).

Table S3- Calculation of the proton exchange rate constant between **[PdCH$_2$•Et$_2$O]$^+$** and **[HBrH•2OEt$_2$]$^+$** upon protonation from ^{1}H-^{1}H EXSY NMR data: AA (16.32, 16.26), AB (16.32, 0.49), BB (0.48, 0.49), BA (0.49, 16.31) and deuteration from ^{1}H-^{1}H EXSY NMR data: AA (16.19, 16.20), AB (16.19, 0.49), BB (0.48, 0.49), BA (0.49, 16.20)

	Mixing time τ_m (s)	*I*(AA)	*I*(AB)	*I*(BB)	*I*(BA)	Diagonal-peak to cross peak ratio (r)[a]	Rate constant *k* (s^{-1})[b]	$\Delta G^{\ddagger}_{298K}$ (kcal/mol)[c]
H$^+$	0.5	0.4457	0.0613	1.0000	0.0712	10.91 ± 2.83	0.37 ± 0.74	22.27 ± 1.20
	1	0.2171	0.0782	1.0000	0.0888	7.29 ± 0.04	0.28 ± 0.01	22.44 ± 0.02
D$^+$	0.5	0.1823	0.1338	1.0000	0.0374	6.91 ± 0.30	0.58 ± 0.13	21.99 ± 0.13
	0.75	0.2406	0.2402	1.0000	0.0655	4.06 ± 0.09	0.67 ± 0.05	21.91 ± 0.04

[a] $r = \frac{I\,(\mathrm{AA})+I\,(\mathrm{BB})}{I\,(\mathrm{AB})+I\,(\mathrm{BA})}$

[b] $k = \frac{1}{\tau_m}\ \ln\left(\frac{r+1}{r-1}\right)$.

[c] $\Delta G^{\ddagger} = -\mathrm{RT}\ln\left(\frac{hk}{k_B T}\right)$ where R = gas constant, T = temperature (298 K), h = Planck's constant, k_B = Boltzmann's constant.

Addition of 2,4-dinitroaniline to [PdCH$_2$•Et$_2$O]$^+$

Addition of 2,4-dinitroaniline to the protonated **[PdCH$_2$•Et$_2$O]$^+$**: In the glovebox, the complex **[PdCH$_2$•Et$_2$O]$^+$** was prepared as described above by protonation with H(Et$_2$O)$_2$[B(C$_6$F$_5$)$_4$] and was dissolved in 0.6 mL CD$_2$Cl$_2$ in a J-Young tube. Then 2,4-dinitroaniline (2.2 mg, 0.012 mmol) was added to the J-Young tube. ^{1}H and ^{31}P NMR (Fig. S47-48) indicated that no reaction happened after mixing for one day. Then, the solvent was dried by applying high vacuum and the solid was redissolved in DCE. A sealed insert tube with acetone-d_6 was placed into the J.Young tube to improve locking/shimming. The ^{31}P NMR spectrum shows that no reaction was taken place in DCE indicating that 2,4-dinitroaniline is not a strong enough base to deprotonate **[PdCH$_2$•Et$_2$O]$^+$**, therefore, the acidity of the **[PdCH$_2$•Et$_2$O]$^+$** is weaker than the conjugate acid of 2,4-dinitroaniline (pK_{ip}^{DCE} = -3.9; pK_a^{THF} = -4.4 ± 0.5).

[H$_3$NAr^F][B(C$_6$F$_5$)$_4$]

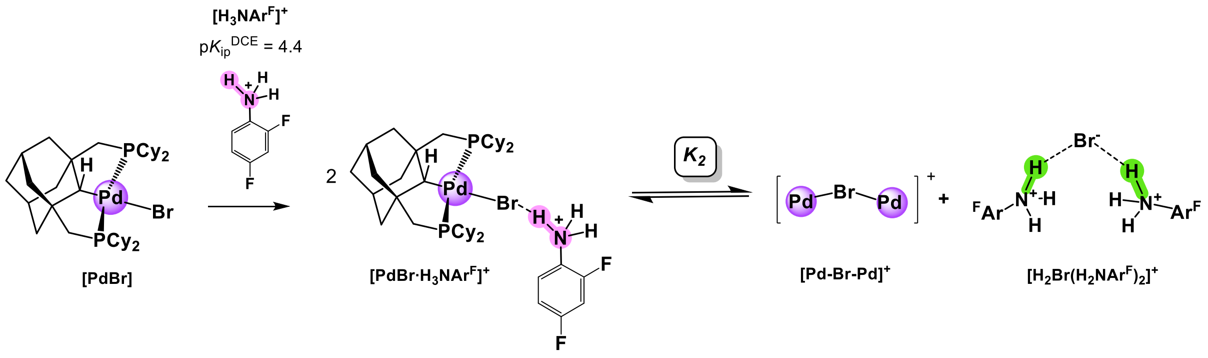

Scheme S2- Proposed equilibrium upon addition of **[H$_3$NAr^F][B(C$_6$F$_5$)$_4$]** to **[PdBr]**.

Van't Hoff analysis (CD_2Cl_2)

In the glovebox, **[PdBr]** (7.4 mg, 0.01 mmol) and **[H_3NAr^F][$B(C_6F_5)_4$]** (8.1 mg, 0.01 mmol) were added in a J-Young tube. Then, 0.550 mL CD_2Cl_2 was added by a 1 mL micro-syringe, which gives the initial **[PdBr]** concentration c_i as 18 (± 3.9%) mmol/L. Then, the J. Young tube was capped. The solution color was very pale yellow after fully mixed. After 3h, $^{31}P\{^1H\}$ NMR was collected from -70 ºC to 25 ºC with 10 ºC intervals between each spectrum. The ^{31}P signal at 52.7 ppm is proposed to be the acid-base adduct **[PdBr•H_3NAr^F]$^+$**. The concentration of **[PdBr•H_3NAr^F]$^+$** (c(PdBr•H_3NAr^F)) and **[Pd-Br-Pd]$^+$** (c(Pd-Br-Pd)) were calculated using equation S5-S6 with their integration I(PdBr•H_3NAr^F) and I(Pd-Br-Pd) relative to the sum of all ^{31}P signals originating from initial **[PdBr]** added to the reaction (c_i). Errors were calculated using standard error propagation. K_2 was calculated at all temperatures based on the ^{31}P NMR by using equation S4. Van't Hoff plot was made with lnK_2 versus 1000/T, which allowed the calculation of experimental thermodynamic values for the equilibrium K_2.

An independent experiment conducted in an identical condition in DCE showed that no **[$PdCH_2$•H_2NAr^F]$^+$** was formed even though spinning the NMR tube on a rotating stirrer for two days. The experiments in both DCM and DCE indicate that **[H_3NAr^F][$B(C_6F_5)_4$]** is not a strong enough acid to protonate **[PdBr]**. Therefore, the acidity of the **[$PdCH_2$]$^+$** is stronger than **[H_3NAr^F][$B(C_6F_5)_4$]** (pK_{ip}^{DCE} = 4.4 ± 0.5; pK_a^{THF} = 3.8 ± 0.5).

$$K_2 = \frac{c(\text{Pd-Br-Pd})^2}{c(\text{PdBr•H}_3\text{NAr}^\text{F})^2} \tag{S4}$$

$$c(\text{PdBr•H}_3\text{NAr}^\text{F}) = \frac{I(\text{PdBr•H}_3\text{NAr}^\text{F})}{0.5\ I(\text{Pd-Br-Pd}) + I(\text{PdBr•H}_3\text{NAr}^\text{F})} \times c_\text{i} \tag{S5}$$

$$c(\text{Pd-Br-Pd}) = \frac{0.5\ I(\text{Pd-Br-Pd})}{0.5\ I(\text{Pd-Br-Pd}) + I(\text{PdBr•H}_3\text{NAr}^\text{F})} \times c_\text{i} \tag{S6}$$

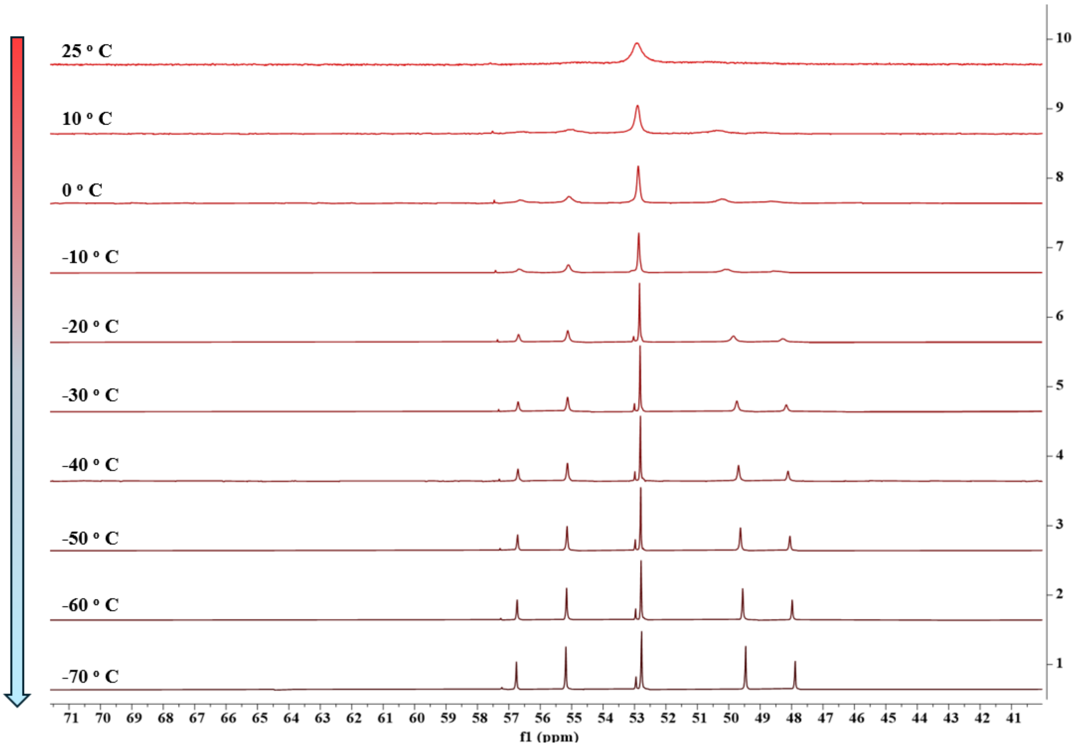

Figure S14- Variable temperature inverse-gated ^{31}P NMR (202 MHz, CD_2Cl_2) spectrum of the equilibrium mixture after addition of **[H_3NAr^F][$B(C_6F_5)_4$]** to **[PdBr]**.

Table S4- Integration of **[PdBr•H$_3$NAr^F]$^+$** (I(PdBr•H$_3$NAr^F)), **[Pd-Br-Pd]$^+$** (I(Pd-Br-Pd)), K_2, and Ln K_2 from -30 ºC to -70 ºC upon addition of **[H$_3$NAr^F][B(C$_6$F$_5$)$_4$]** to **[PdBr]** in CD$_2$Cl$_2$. Integrals are measured relative to the ^{31}P resonance for **[PdBr•H$_3$NAr^F]$^+$** (I(PdBr•H$_3$NAr^F)) which is set to exactly 1.

T(ºC)	*I*(Pd-Br-Pd)	*I*(PdBr•H$_3$NAr^F)	K_2	Ln K_2
-30	1.90 (4)	1.00	0.90 (9)	-0.10 (9)
-40	1.96 (5)	1.00	0.96 (9)	-0.04 (9)
-50	2.05 (1)	1.00	1.05 (8)	0.05 (8)
-60	2.15 (1)	1.00	1.16 (9)	0.14 (8)
-70	2.25 (1)	1.00	1.27 (10)	0.24 (8)

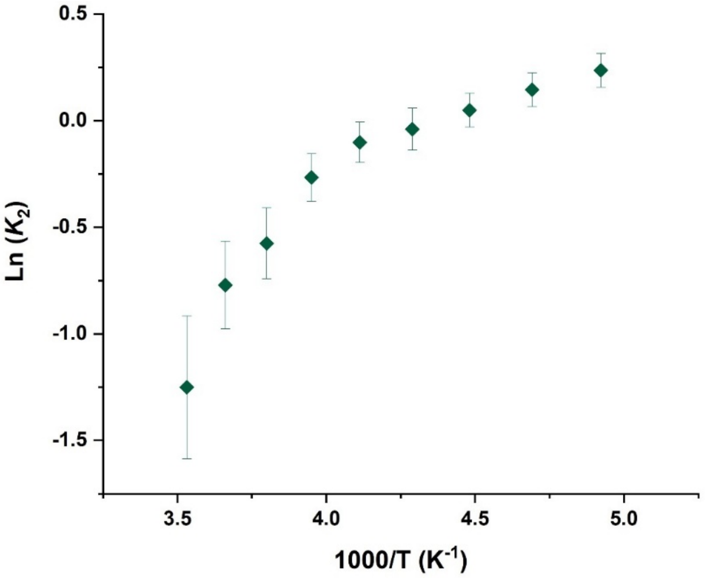

Figure S15- Full region of the Van't Hoff plot for the reaction of **[H$_3$NAr^F][B(C$_6$F$_5$)$_4$]** and **[PdBr]** in CD$_2$Cl$_2$. Data at higher temperatures were not considered due to the large errors associated with broadened ^{31}P resonances.

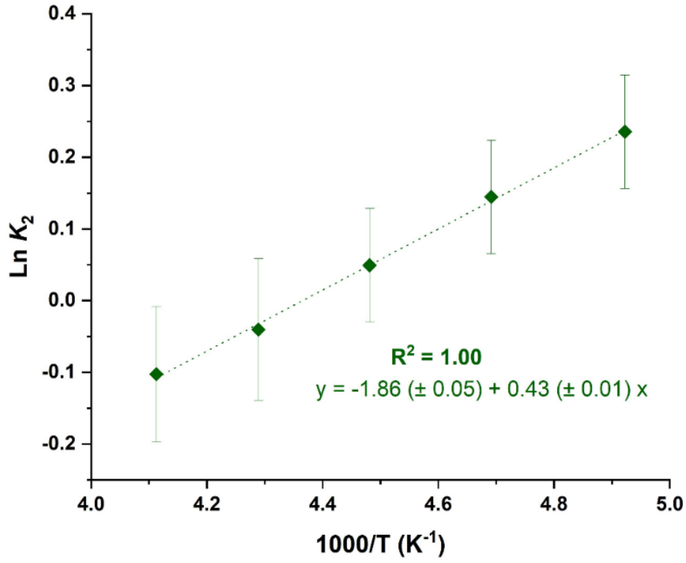

Figure S16- Linear region of the Van't Hoff plot for the reaction of **[H$_3$NAr^F][B(C$_6$F$_5$)$_4$]** and **[PdBr]** in CD$_2$Cl$_2$.

Table S5- Experimental thermodynamic values for the equilibrium upon addition of **[H_3NAr^F][$B(C_6F_5)_4$]** to **[PdBr]** in CD_2Cl_2.

	ΔH (kcal/mol)	ΔS (cal/(mol·K))	ΔG_{298K} (kcal/mol)
K_2	-0.86 ± 0.02	-3.7 ± 0.1	0.25 ± 0.04

EXSY analysis (CD_2Cl_2)

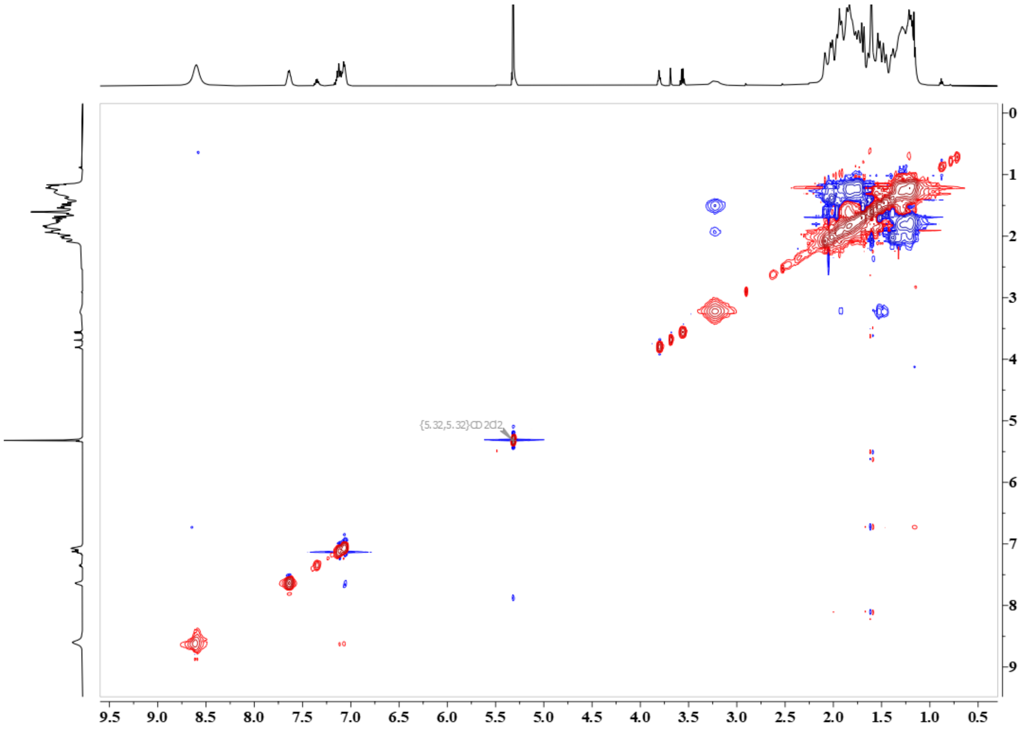

Figure S17- Section of 1H-1H EXSY (red) -NOESY(blue) NMR spectrum of the equilibrium mixture after protonation of **[PdBr]** with **[H_3NAr^F][$B(C_6F_5)_4$]** (500, 500 MHz, CD_2Cl_2, 25 °C, τ_m = 0.5 s, NS = 64).

$[H_3NAr^{Cl}][B(C_6F_5)_4]$

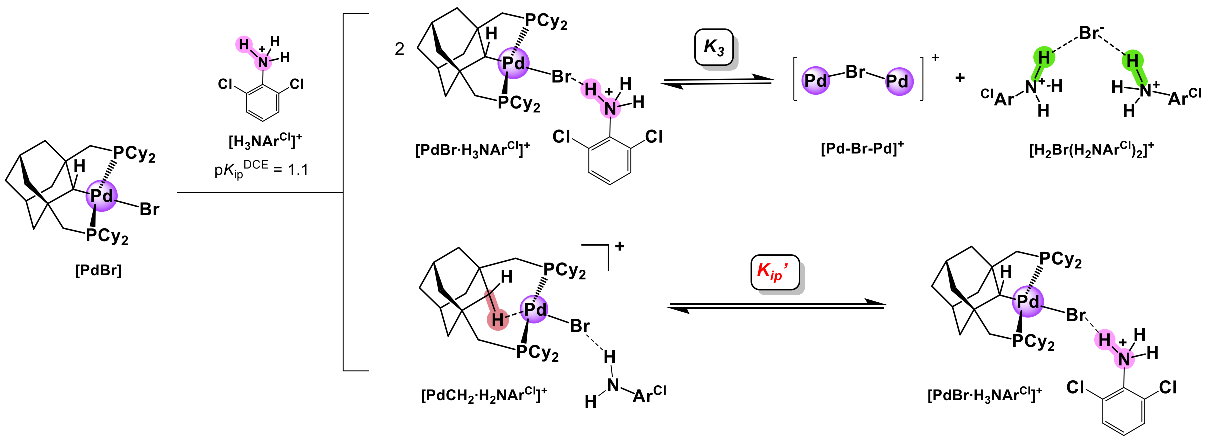

Scheme S3- Proposed equilibria upon addition of $\mathbf{[H_3NAr^{Cl}][B(C_6F_5)_4]}$ to **[PdBr]**.

$$c(PdCH_2{\bullet}H_2NAr^{Cl}) = \frac{I(PdCH_2{\bullet}H_2NAr^{Cl})}{0.5\ I(Pd\text{-}Br\text{-}Pd) + I(PdCH_2{\bullet}H_2NAr^{Cl}) + I(PdBr{\bullet}H_3NAr^{Cl})} \times c_i \quad (S7)$$

$$c(PdBr{\bullet}H_3NAr^{Cl}) = \frac{I(PdBr{\bullet}H_3NAr^{Cl})}{0.5\ I(Pd\text{-}Br\text{-}Pd) + I(PdCH_2{\bullet}H_2NAr^{Cl}) + I(PdBr{\bullet}H_3NAr^{Cl})} \times c_i \quad (S8)$$

$$c(Pd\text{-}Br\text{-}Pd) = \frac{0.5\ I(Pd\text{-}Br\text{-}Pd)}{0.5\ I(Pd\text{-}Br\text{-}Pd) + I(PdCH_2{\bullet}H_2NAr^{Cl}) + I(PdBr{\bullet}H_3NAr^{Cl})} \times c_i \quad (S9)$$

$$K_3 = \frac{c(Pd\text{-}Br\text{-}Pd)^2}{c(PdBr{\bullet}H_3NAr^{Cl})^2} \quad (S10)$$

$$K_{ip}' = \frac{c(PdBr{\bullet}H_3NAr^{Cl})}{c(PdCH_2{\bullet}H_2NAr^{Cl})} \quad (S11)$$

Van't Hoff analysis (DCE)

In the glovebox, **[PdBr]** (8.2 mg, 0.011 mmol) and $\mathbf{[H_3NAr^{Cl}][B(C_6F_5)_4]}$ (9.3 mg, 0.011 mmol) were added in a J-Young tube. Then, 0.55 mL DCE was added by a 1 mL micro-syringe, which gives the initial **[PdBr]** concentration c_i as 20 (± 3.8%) mmol/L. Then, the J. Young tube was capped. The solution color turned brown after fully mixed. After 30 mins, $^{31}P\{^1H\}$ NMR was collected from -15 °C to 25 °C with 5 °C intervals between each spectrum. The ^{31}P signal at 52.7 ppm is proposed to be the acid-base adduct $\mathbf{[PdBr{\bullet}H_3NAr^{Cl}]^+}$. The concentration of $\mathbf{[PdCH_2{\bullet}H_2NAr^{Cl}]^+}$ ($c(PdCH_2{\bullet}H_2NAr^{Cl})$), $\mathbf{[PdBr{\bullet}H_3NAr^{Cl}]^+}$ ($c(PdBr{\bullet}H_3NAr^{Cl})$) and $\mathbf{[Pd\text{-}Br\text{-}Pd]^+}$ (c(Pd-Br-Pd)) were calculated using equation S7-9 with their integration $I(PdCH_2{\bullet}H_2NAr^{Cl})$, $I(PdBr{\bullet}H_3NAr^{Cl})$ and I(Pd-Br-Pd) relative to the sum of all ^{31}P signals originating from initial **[PdBr]** added to the reaction (c_i). Error was calculated using standard error propagation. K_3 and K_{ip}' were calculated at all temperatures based on the ^{31}P NMR by using equation S10-11. Therefore, we estimated that p$K_{ip}'^{DCE}$ of $\mathbf{[PdCH_2]^+}$ is 0.18 ± 0.51 using equation S12 with K_{ip}' = 8.31 ± 2.17 at 25 °C. The pK_a^{MeCN}, and pK_a^{THF} were calculated using equations S13-14[9, 10]. Van't Hoff plots are made for K_3 and K_{ip}' with their corresponding lnK_{eq} versus 1000/T, which allows the calculation of experimental thermodynamic values for the equilibrium processes.

$$pK_{ip}'([PdCH_2]^+, DCE) = pK_{ip}([H_3NAr^{Cl}]^+, DCE) - \log K_{ip}' \quad (S12)$$

$$pK_{ip}'(DCE) = pK_a(MeCN) \cdot 0.99 - 3.93 \quad (S13)$$

$$pK_a\,(THF) = pK_a\,(MeCN) \cdot (0.98 \pm 0.02) - (6.1 \pm 0.4) + 2 \cdot (0.84 \pm 0.16) \quad (S14)$$

Table S6- acidity of anilinium acids and the calculated acidity of **$[PdCH_2]^+$** in MeCN, DCE and THF.

	2,4-dinitroanilinium	**$[H_3NAr^F][B(C_6F_5)_4]$**	**$[H_3NAr^{Cl}][B(C_6F_5)_4]$**	**$[PdCH_2]^+$**
pK_a^{MeCN}	0.03^b	8.39	5.07	4.2 ± 0.5^b
pK_{ip}^{DCE}	-3.9	4.4 ± 0.5^b	1.1 ± 0.5^b	0.18 ± 0.51^a
pK_a^{THF}	-4.4 ± 0.5^c	3.8 ± 0.5^c	0.6 ± 0.5^c	**-0.32 ± 0.71^c**

[a] calculated with equation S12

[b] calculated with equation S13

[c] calculated with equation S14

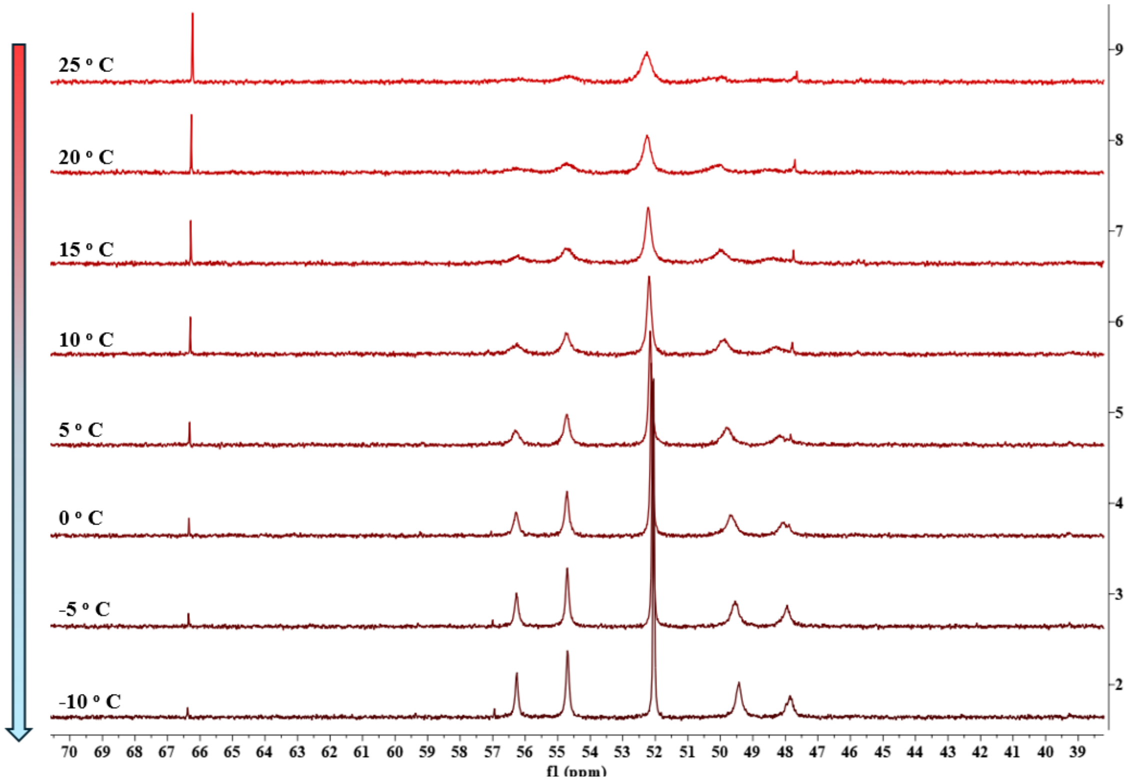

Figure S18- Variable temperature inverse-gated ^{31}P NMR (202 MHz, DCE) spectrum of the equilibrium mixture after addition of **$[H_3NAr^{Cl}][B(C_6F_5)_4]$** to **[PdBr]**.

Table S7- Integration of **[PdCH$_2$•H$_2$NAr^{Cl}]$^+$** (I(PdCH$_2$•H$_2$NAr^{Cl})), **[PdBr•H$_3$NAr^{Cl}]$^+$** (I(PdBr•H$_3$NAr^{Cl})), **[Pd-Br-Pd]$^+$** (I(Pd-Br-Pd)), K_3, K_{ip}', Ln K_3 and Ln K_{ip}' from 25 °C to -10 °C upon addition of **[H$_3$NAr^{Cl}][B(C$_6$F$_5$)$_4$]** to **[PdBr]** in DCE. Integrals are measured relative to the ^{31}P resonance for **[PdCH$_2$•H$_2$NAr^{Cl}]$^+$** (I(PdCH$_2$•H$_2$NAr^{Cl})) which is set to exactly 1.

T(°C)	*I*([PdCH$_2$• H$_2$NAr^{Cl}]$^+$)	*I*(Pd-Br-Pd)	I([PdBr• H$_3$NAr^{Cl}]$^+$)	K_3	Ln K_3	K_{ip}'	Ln K_{ip}'
**25**	1.00 (3)	12.36 (2.82)	8.31	0.55 (27)	-0.59 (49)	8.31 (2.17)	2.12 (26)
**20**	1.00 (3)	20.89 (3.41)	12.38	0.71 (26)	-0.34 (37)	12.38 (2.58)	2.52 (21)
**15**	1.00 (4)	32.49 (4.15)	18.67	0.76 (22)	-0.28 (30)	18.67 (3.22)	2.93 (17)
**10**	1.00 (5)	34.80 (3.20)	20.01	0.76 (16)	-0.28 (22)	20.01 (2.66)	3.00 (13)
**5**	1.00 (8)	43.40 (2.78)	25.31	0.74 (11)	-0.31 (16)	25.31 (3.02)	3.23 (12)
**0**	1.00 (10)	53.90 (2.31)	31.18	0.75 (8)	-0.29 (11)	31.18 (3.78)	3.44 (12)
**-5**	1.00 (13)	77.97 (2.56)	44.97	0.75 (7)	-0.29 (9)	44.97 (6.56)	3.81 (15)
**-10**	1.00 (18)	126.35 (3.52)	75.58	0.70 (6)	-0.36 (8)	75.58 (14.07)	4.33 (19)

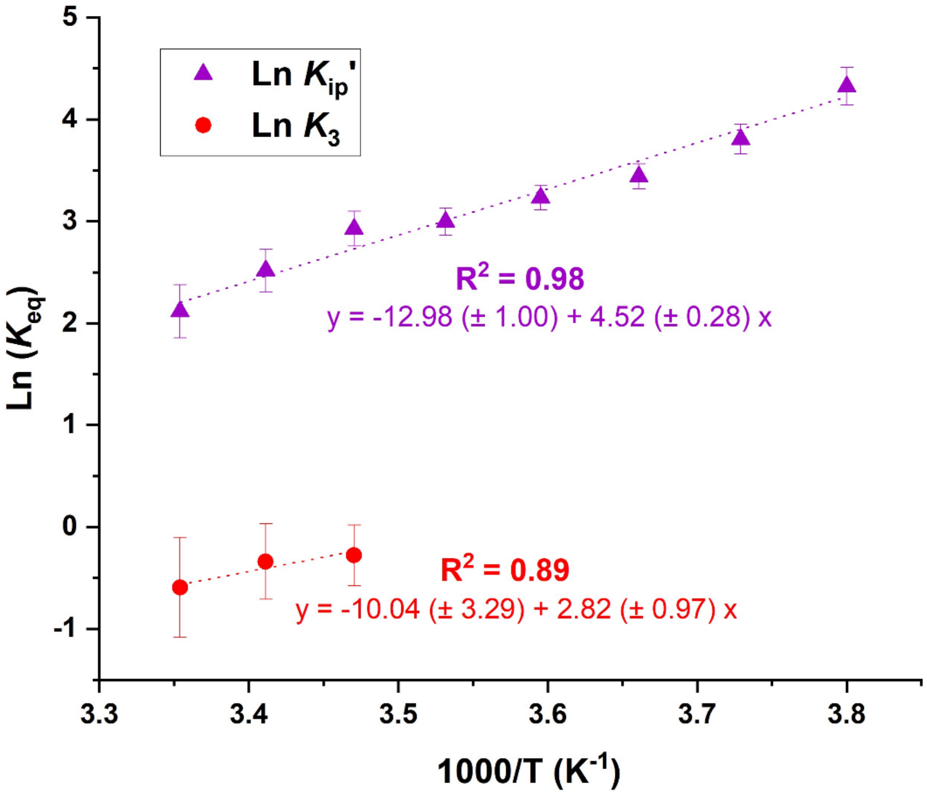

Figure S19- Van't Hoff plot for the reaction of **[H$_3$NAr^{Cl}][B(C$_6$F$_5$)$_4$]** and **[PdBr]** in DCE.

Table S8- Experimental thermodynamic values for the equilibrium upon addition of **[H$_3$NAr^{Cl}][B(C$_6$F$_5$)$_4$]** to **[PdBr]** in DCE.

	ΔH (kcal/mol)	ΔS (cal/(mol·K))	ΔG_{298K} (kcal/mol)
K_3	-5.6 ± 1.9	-20.1 ± 6.6	0.3 ± 2.8
K_{ip}'	-9.0 ± 0.6	-26.0 ± 2.0	-1.3 ± 0.8

Van't Hoff analysis (CD2Cl2)

In the glovebox, **[PdBr]** (7.4 mg, 0.01 mmol) and **[H_3NAr^{Cl}][$B(C_6F_5)_4$]** (8.4 mg, 0.01 mmol) were added in a J-Young tube. Then, 0.550 mL CD_2Cl_2 was added by a 1 mL micro-syringe, which gives the initial **[PdBr]** concentration c_i as 18 (± 3.9%) mmol/L. Then, the J. Young tube was capped. The solution color turned brown after fully mixed. After 1.5h, inverse-gated $^{31}P\{^1H\}$ NMR was collected from -70 ºC to 25 ºC with 10 ºC intervals between each spectrum (Fig. S20). The relative error of the integration of the peaks at 48.7 ppm and 66.4 ppm in ^{31}P NMR spectrum were obtained by the reciprocal for their S/N (signal/noise), which were the main contribution to the spectrum error. The ^{31}P signal at 52.7 ppm was proposed to be the acid-base adduct **[$PdBr•H_3NAr^{Cl}$]$^+$**. The concentration of **[$PdCH_2•H_2NAr^{Cl}$]$^+$** (c($PdCH_2•H_2NAr^{Cl}$)), **[$PdBr•H_3NAr^{Cl}$]$^+$** (c($PdBr•H_3NAr^{Cl}$)) and **[Pd-Br-Pd]$^+$** (c(Pd-Br-Pd)) were calculated using equation S7-9 with their integration I($PdCH_2•H_2NAr^{Cl}$), I($PdBr•H_3NAr^{Cl}$) and I(Pd-Br-Pd) relative to the sum of all ^{31}P signals originating from initial **[PdBr]** added to the reaction (c_i). Error was calculated using standard error propagation. K_3 and K_{ip}' were calculated at all temperatures based on the ^{31}P NMR by using equation S10-11. Van't Hoff plot was made for K_3 and K_{ip}' with their corresponding lnK_{eq} versus 1000/T, which allowed the calculation of experimental thermodynamic values for the equilibrium processes. Van't Hoff plots were made from 10 ºC to -30 ºC, where the intensity of the peak at 66.4 ppm allows accurate integration.

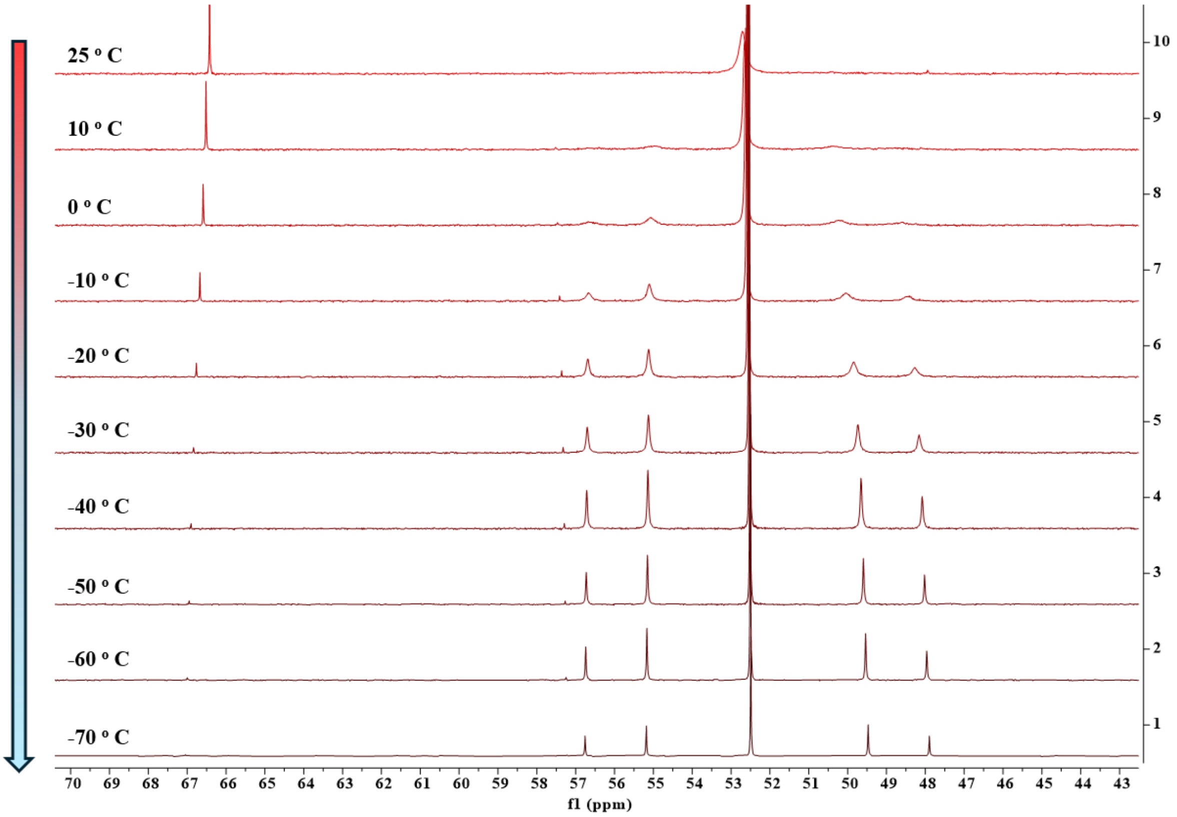

Figure S20- Variable temperature inverse-gated ^{31}P NMR (202 MHz, CD_2Cl_2) spectrum of the equilibrium mixture after addition of **[H_3NAr^{Cl}][$B(C_6F_5)_4$]** to **[PdBr]**.

Table S9- Integration of **[$PdCH_2$•H_2NAr^{Cl}]$^+$** (I($PdCH_2$•H_2NAr^{Cl})), **[PdBr•H_3NAr^{Cl}]$^+$** (I(PdBr•H_3NAr^{Cl})), **[Pd-Br-Pd]$^+$** (I(Pd-Br-Pd)), K_3, K_{ip}', Ln K_3 and Ln K_{ip}' from 10 ºC to -30 ºC upon addition of **[H_3NAr^{Cl}][B(C_6F_5)$_4$]** to **[PdBr]** in CD_2Cl_2. Integrals are measured relative to the ^{31}P resonance for **[$PdCH_2$•H_2NAr^{Cl}]$^+$** (I($PdCH_2$•H_2NAr^{Cl})) which is set to exactly 1.

T(°C)	I([$PdCH_2$•H_2NAr^{Cl}]$^+$)	I(Pd-Br-Pd)	I([PdBr•H_3NAr^{Cl}]$^+$)	K_3 * 10^2	Ln K_3	K_{ip}' * 10^{-1}	Ln K_{ip}'
**10**	1.00 (2)	5.01 (1.12)	10.70	5.48 (1.99)	-2.90 (36)	1.07 (13)	2.37 (13)
**0**	1.00 (2)	9.38 (1.48)	16.85	7.75 (2.10)	-2.56 (27)	1.69 (19)	2.82 (11)
**-10**	1.00 (3)	19.9 (1.96)	31.46	10.00 (1.85)	-2.30 (18)	3.15 (28)	3.45 (9)
**-20**	1.00 (7)	44.53 (2.24)	60.75	13.43 (1.59)	-2.01 (12)	6.08 (59)	4.11 (10)
**-30**	1.00 (17)	121.63 (3.27)	150.15	16.40 (1.50)	-1.81 (9)	15.02 (2.76)	5.01 (18)

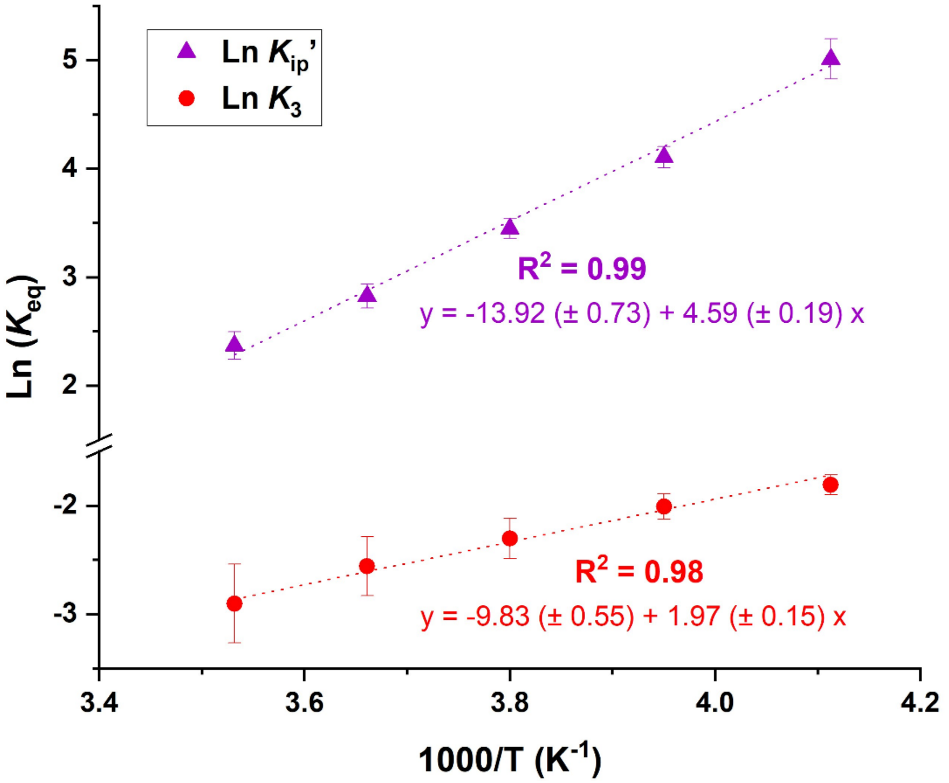

Figure S21- Van't Hoff plot for the reaction of **[H_3NAr^{Cl}][B(C_6F_5)$_4$]** and **[PdBr]** in CD_2Cl_2.

Table S10- Experimental thermodynamic values for the equilibrium upon addition of **[H_3NAr^{Cl}][B(C_6F_5)$_4$]** to **[PdBr]** in CD_2Cl_2.

	ΔH (kcal/mol)	ΔS (cal/(mol·K))	ΔG_{298K} (kcal/mol)
K_3	-3.9 ± 0.3	-19.7 ± 1.1	1.9 ± 0.4
K_{ip}'	-9.2 ± 0.4	-27.8 ± 1.5	-0.9 ± 0.6

EXSY analysis (CD_2Cl_2)

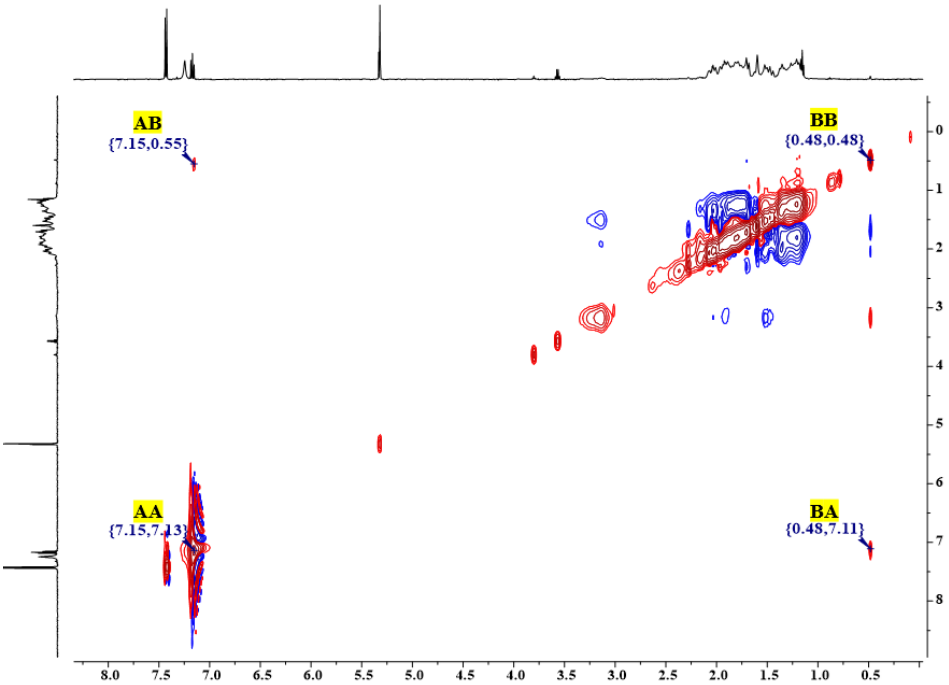

Figure S22- Section of ^{1}H-^{1}H EXSY (red) -NOESY(blue) NMR spectrum of the equilibrium mixture after protonation of **[PdBr]** with **[H$_3$NAr^{Cl}][B(C$_6$F$_5$)$_4$]** (500, 500 MHz, CD_2Cl_2, 25 °C, τ_m = 0.5 s, NS = 64).

Table S11- Calculation of the proton exchange rate constant between **[PdBr•H$_3$NAr^{Cl}]$^+$** and **[PdCH$_2$•H$_2$NAr^{Cl}]$^+$** from ^{1}H-^{1}H EXSY NMR data. AA (7.15, 7.13), AB (7.15, 0.55), BB (0.48, 0.48), BA (0.48, 7.11).

Mixing time τ_m (s)	*I*(AA)	*I*(AB)	*I*(BB)	*I*(BA)	Diagonal-peak to cross peak ratio (r)[a]	Rate constant *k* (s^{-1})[b]	$\Delta G^{\ddagger}_{298}$ (kcal/mol)[c]
0.5	1	0.0069	0.083	0.0143	51.08 (9.54)	0.078 (0.529)	23.2 (4.0)

[a] $r = \frac{I\,(AA)+I\,(BB)}{I\,(AB)+I\,(BA)}$

[b] $= \frac{1}{\tau_m}\ \ln\left(\frac{r+1}{r-1}\right)$.

[c] $\Delta G^{\ddagger} = -RT\ln\left(\frac{hk}{k_B T}\right)$ where R = gas constant, T = temperature (298K), h = Planck's constant, k_B = Boltzmann's constant.

Table S12- Summary of experimental thermodynamic values for the equilibrium processes K_1, K_2, K_3 and K_{ip}'.

		ΔH (kcal/mol)	ΔS (cal/(mol·K))	ΔG_{298K} (kcal/mol)
K_1	H^+	-1.4 ± 0.1	-5.4 ± 0.2	0.20 ± 0.08
	D^+	-3.5 ± 0.2	-13.4 ± 0.7	0.52 ± 0.29
K_2	CD_2Cl_2	-0.86 ± 0.02	-3.7 ± 0.1	0.25 ± 0.04
K_3	CD_2Cl_2	-3.9 ± 0.3	-19.7 ± 1.1	1.9 ± 0.4
	DCE	-5.6 ± 1.9	-20.1 ± 6.6	0.3 ± 2.8
K_{ip}'	CD_2Cl_2	-9.2 ± 0.4	-27.8 ± 1.5	-0.88 ± 0.58
	DCE	-9.0 ± 0.6	-26.0 ± 2.0	-1.3 ± 0.8

NMR Spectra

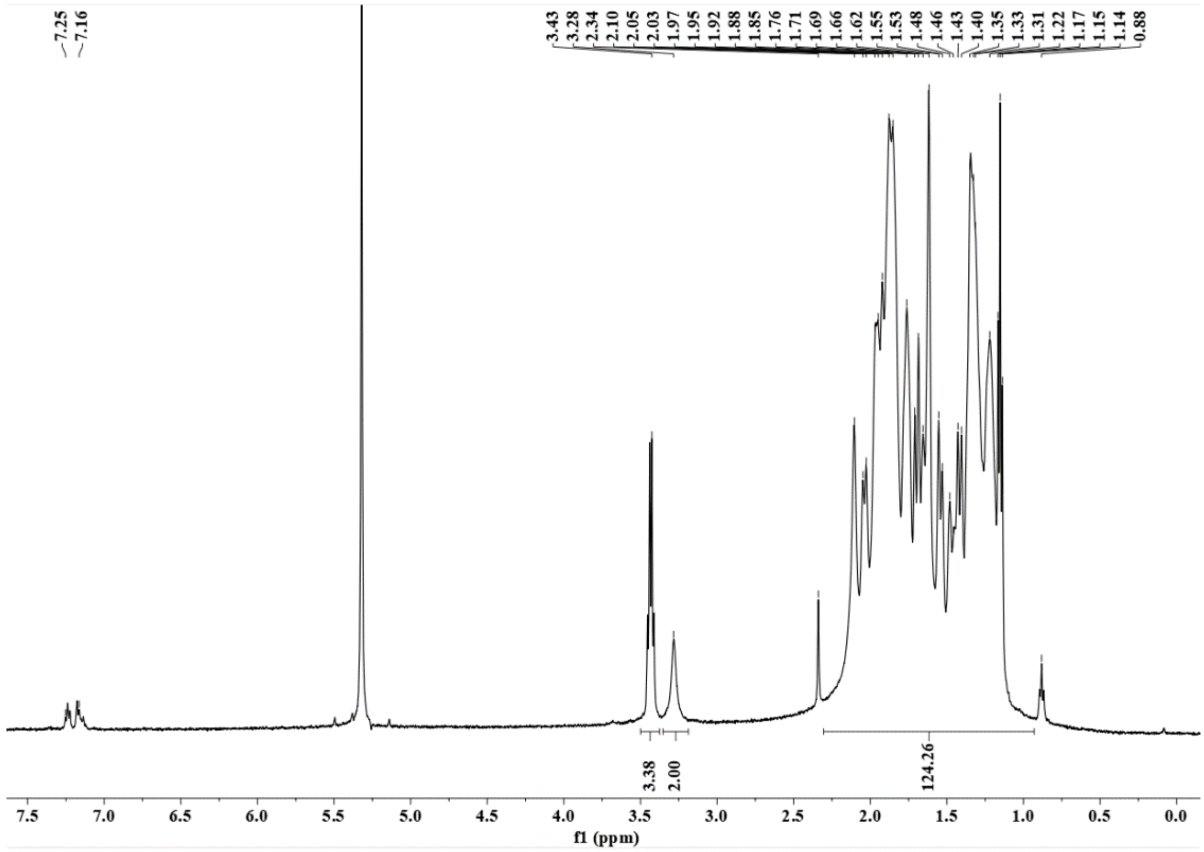

Figure S23- ^{1}H NMR spectrum of **[Pd-Br-Pd]**$^+$ (500 MHz, CD_2Cl_2, 25 °C). Signals at 7.25, 7.16 and 2.34 ppm are residual toluene, 3.43 and 1.15 ppm are residual Et_2O, and 0.88 ppm is pentane.

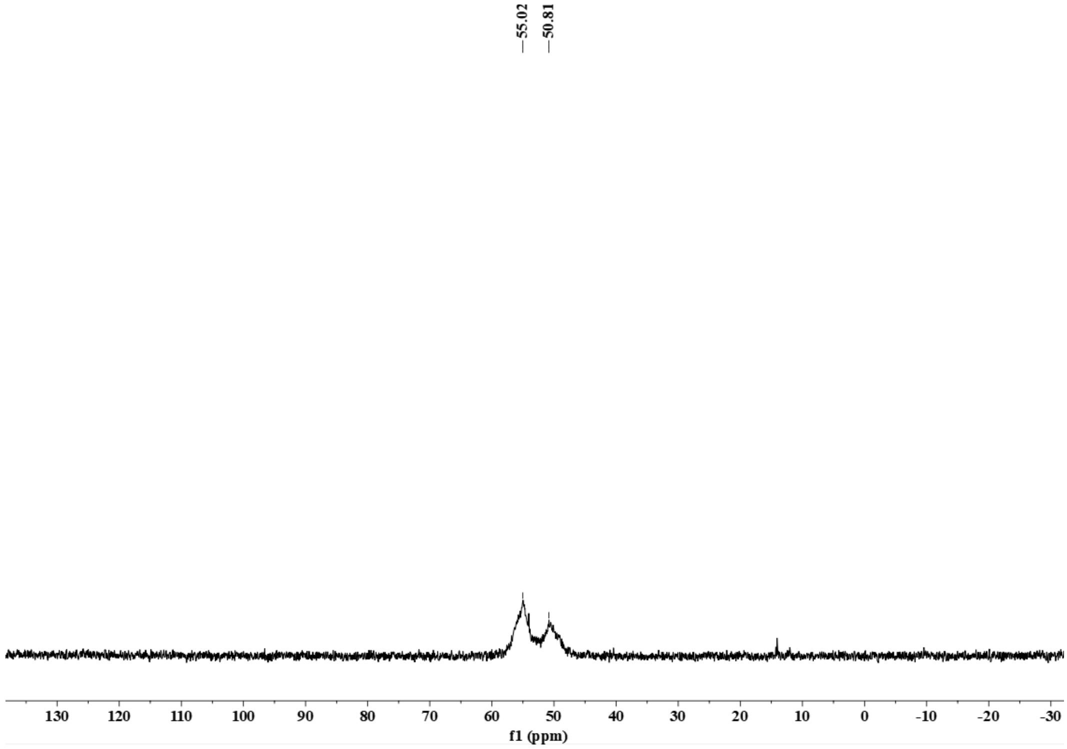

Figure S24- ^{31}P NMR spectrum of **[Pd-Br-Pd]**$^+$ (202 MHz, CD_2Cl_2, 25 °C).

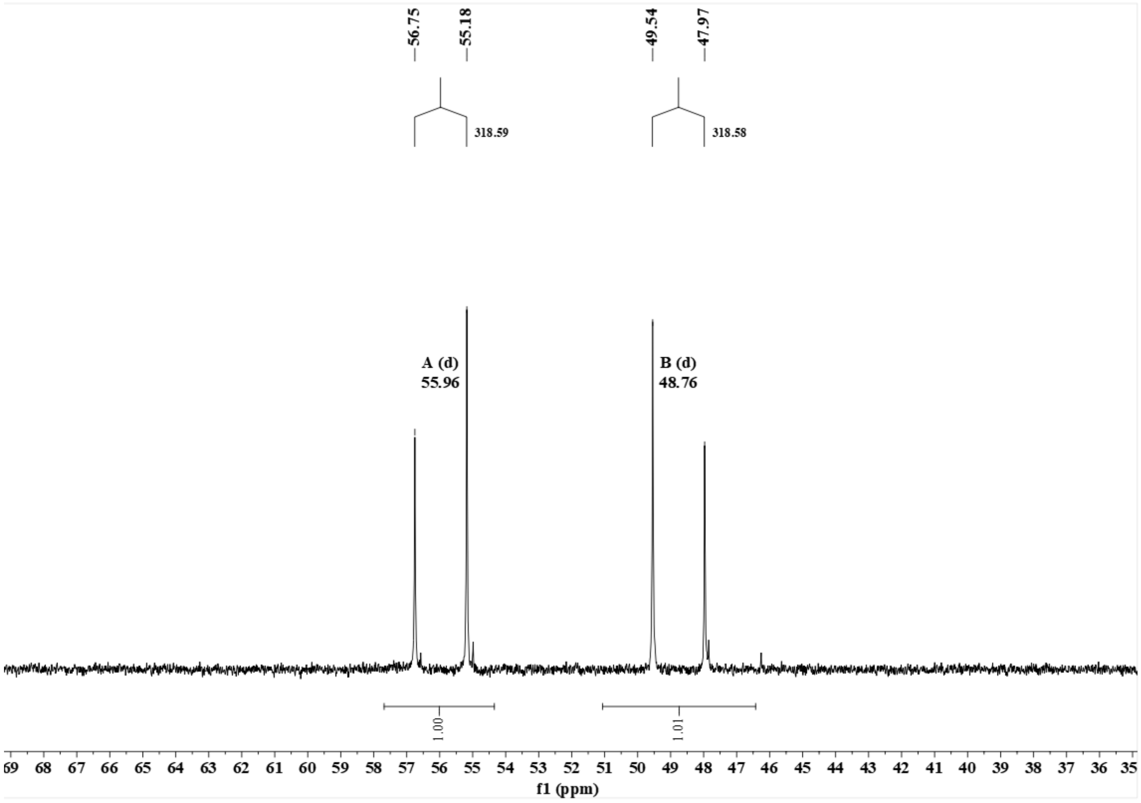

Figure S25- ^{31}P NMR spectrum of **[Pd-Br-Pd]**$^+$ (202 MHz, CD_2Cl_2, -60 °C).

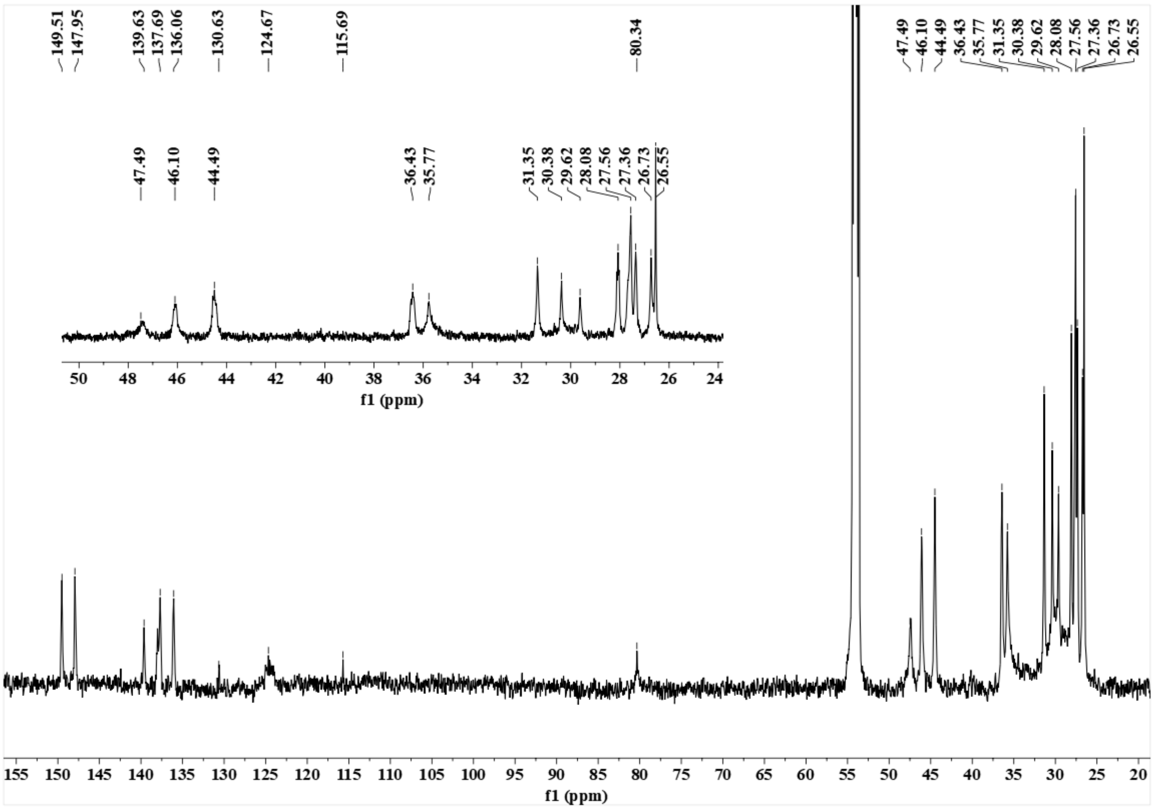

Figure S26- ^{13}C NMR spectrum of **[Pd-Br-Pd]**$^+$ (151 MHz, CD_2Cl_2, 25 °C). Signals at 130.63, 124.67 and 115.69 ppm are residual PhF.

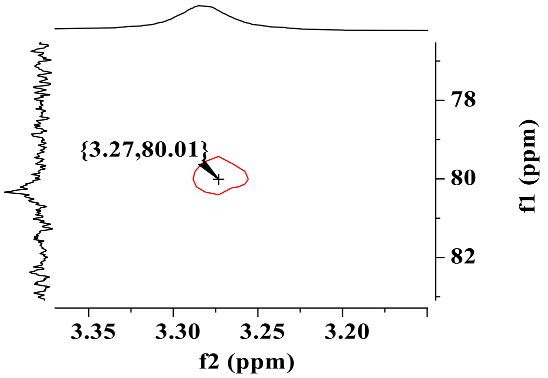

Figure S27- Section of ^{1}H-^{13}C HSQC NMR spectrum of **[Pd-Br-Pd]**$^{+}$ showing the Pd-CH cross peak (600, 151 MHz, CD_2Cl_2, 25 °C).

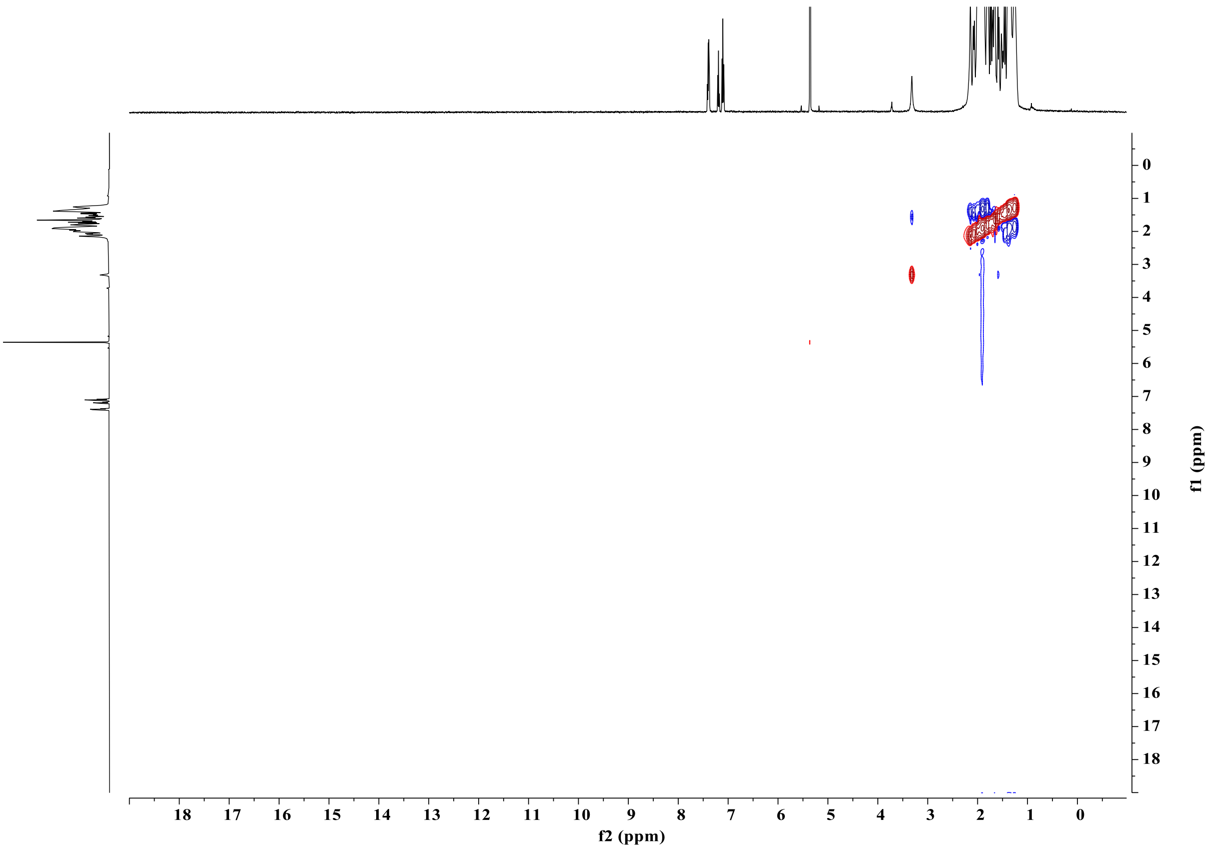

Figure S28- ^{1}H-^{1}H EXSY NMR spectrum of **[Pd-Br-Pd]**$^{+}$, showing no off-diagonal chemical exchange cross-peaks. (500 MHz, CD_2Cl_2, 25 °C, τ_m = 0.5 s, NS = 64).

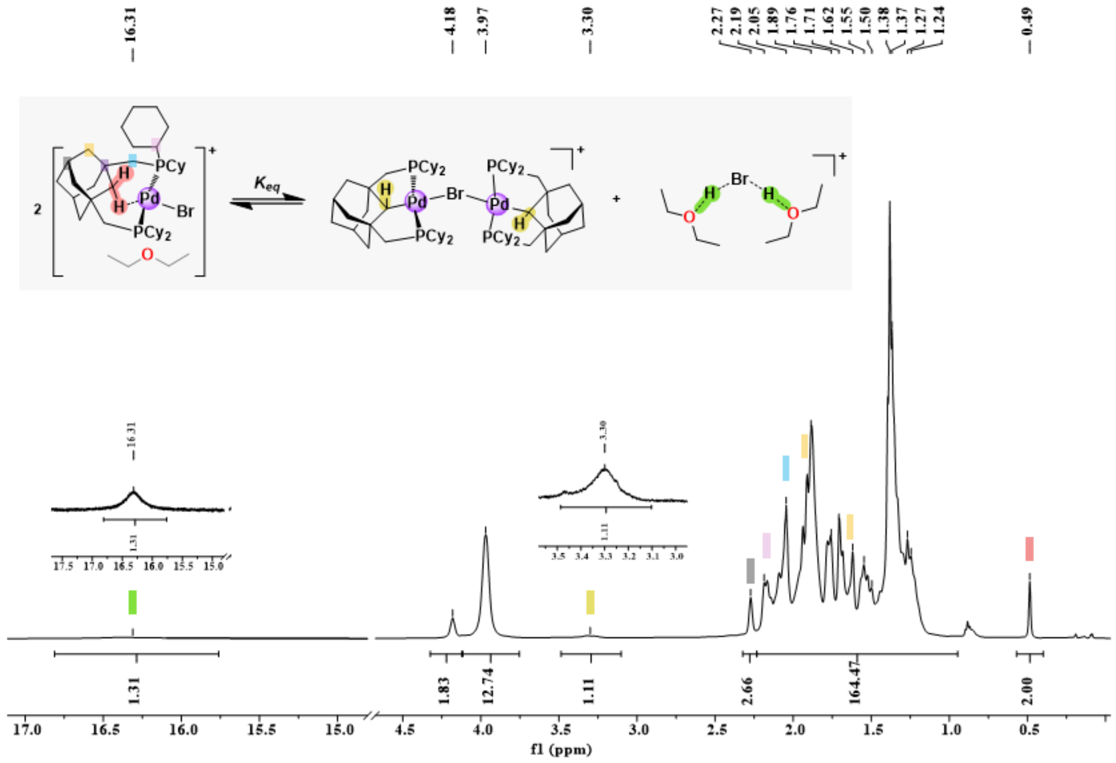

Figure S29- ^{1}H NMR spectrum of the equilibrium mixture after protonation of **[PdBr]** with $H(Et_2O)_2[B(C_6F_5)_4]$, showing a mixture of **[PdCH₂•Et₂O]**$^+$, **[Pd-Br-Pd]**$^+$, and **[HBrH•2OEt₂]**$^+$ (500 MHz, CD_2Cl_2, 25 °C).

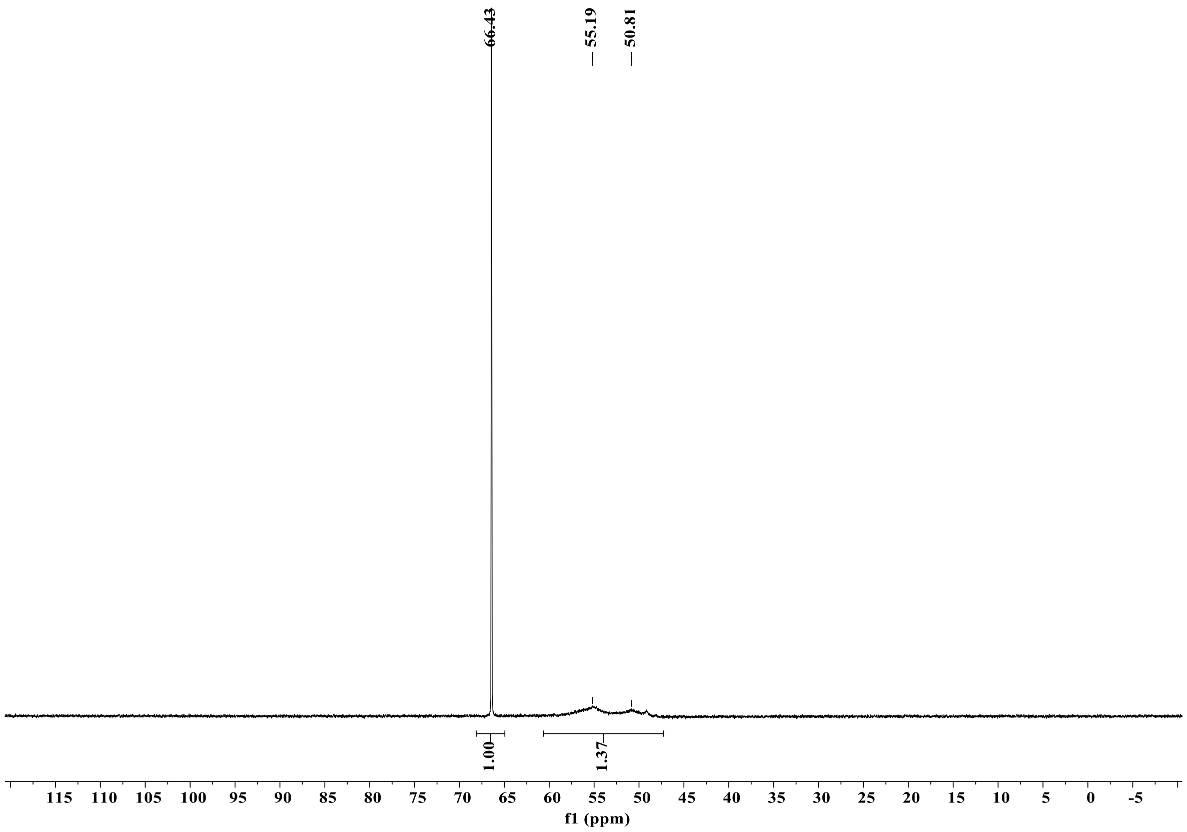

Figure S30- ^{31}P NMR spectrum of the equilibrium mixture after protonation of **[PdBr]** with $H(Et_2O)_2[B(C_6F_5)_4]$, showing a mixture of **[PdCH₂•Et₂O]**$^+$ and **[Pd-Br-Pd]**$^+$ (202 MHz, CD_2Cl_2, 25 °C).

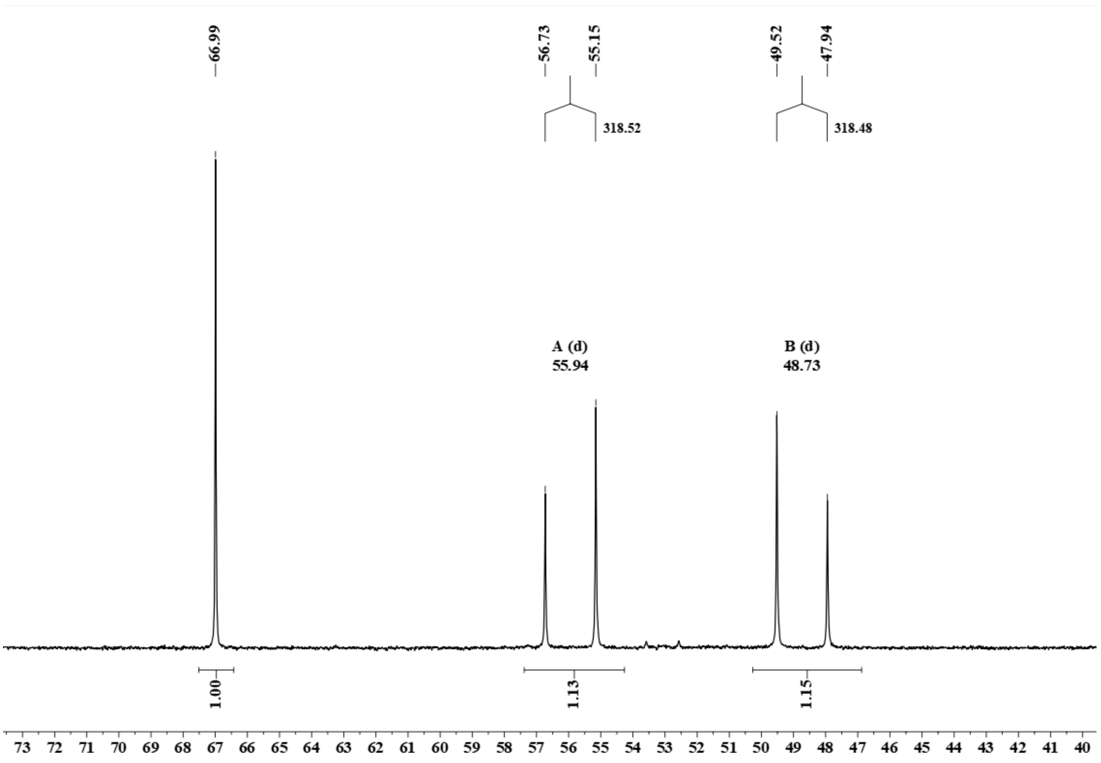

Figure S31- ^{31}P NMR spectrum of the equilibrium mixture after protonation of **[PdBr]** with $H(Et_2O)_2[B(C_6F_5)_4]$, showing a mixture of **[PdCH₂•Et₂O]**$^+$ and **[Pd-Br-Pd]**$^+$ (202 MHz, CD_2Cl_2, -60 °C).

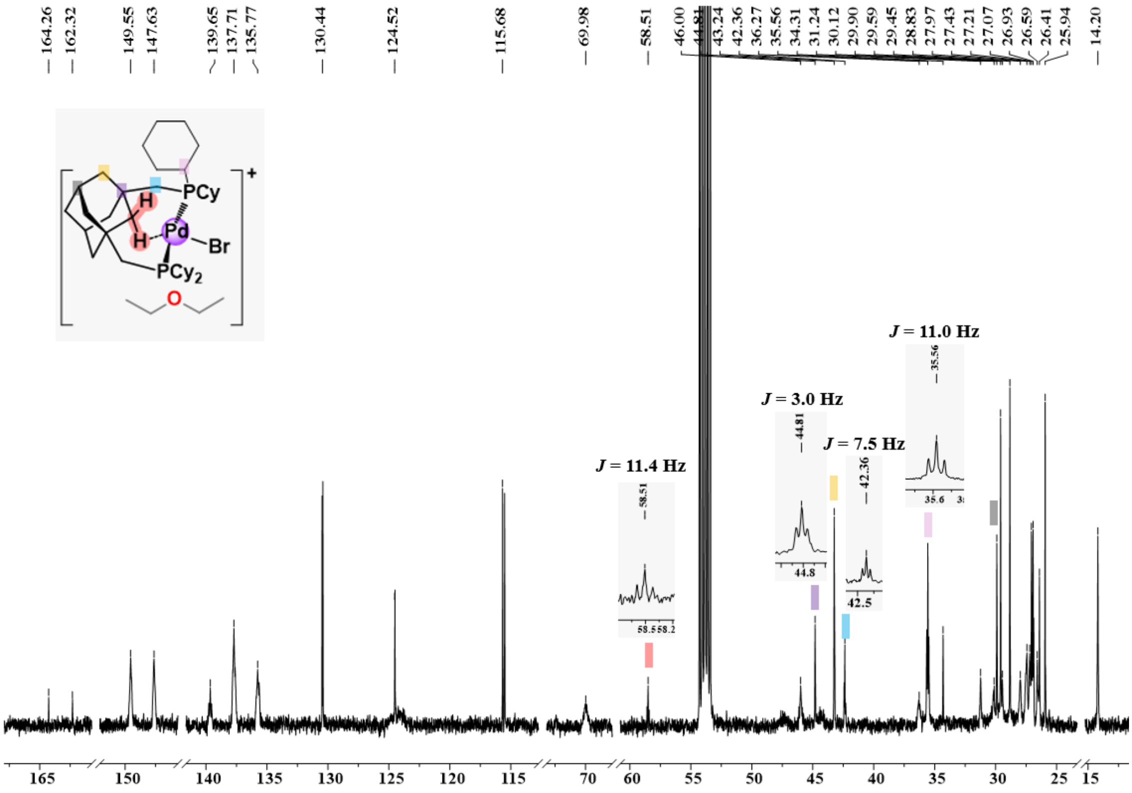

Figure S32- ^{13}C NMR spectrum of the equilibrium mixture after protonation of **[PdBr]** with $H(Et_2O)_2[B(C_6F_5)_4]$ (126 MHz, CD_2Cl_2, 25 °C). Signals at 130.44, 124.52 and 115.68 ppm are residual PhF.

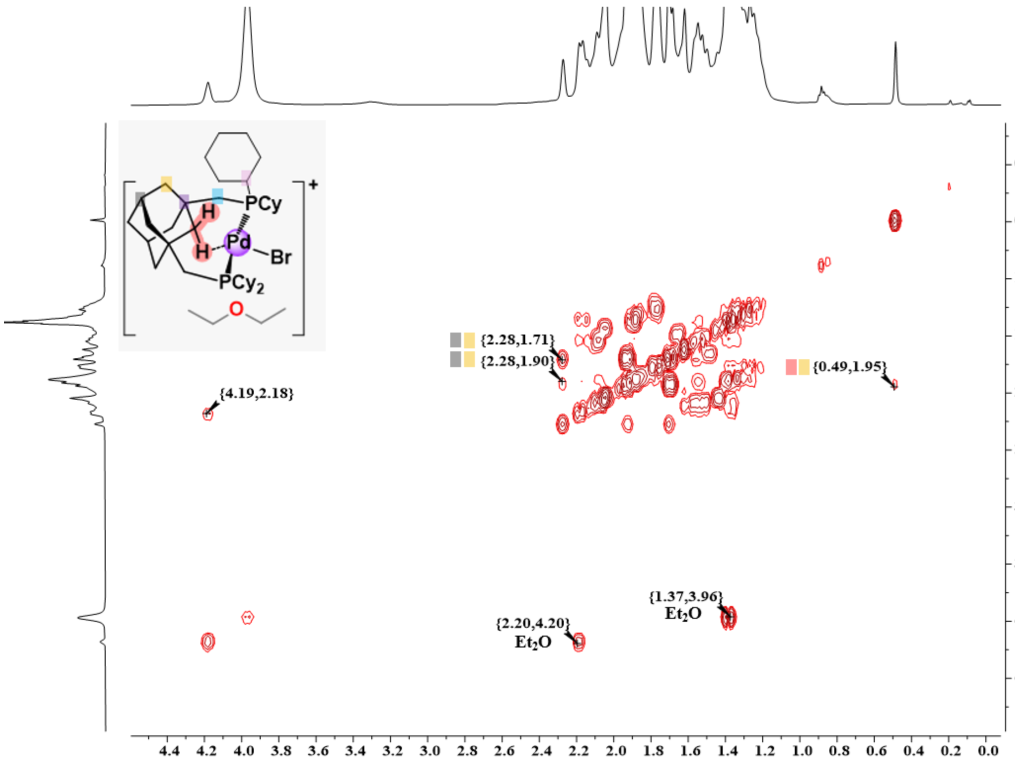

Figure S33- ${}^{1}H$-${}^{1}H$ COSY NMR spectrum of the equilibrium mixture after protonation of **[PdBr]** with $H(Et_2O)_2[B(C_6F_5)_4]$ (500, 500 MHz, CD_2Cl_2, 25 °C).

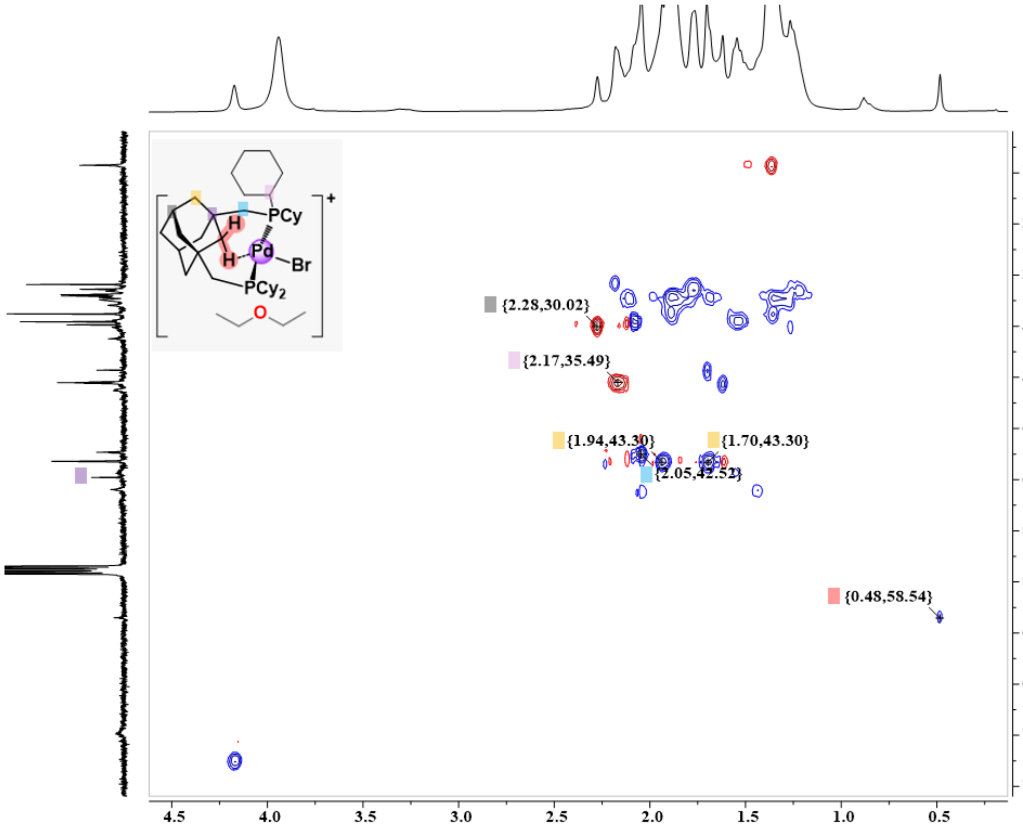

Figure S34- ${}^{1}H$-${}^{13}C$ HSQC NMR spectrum of the equilibrium mixture after protonation of **[PdBr]** with $H(Et_2O)_2[B(C_6F_5)_4]$ (600, 151 MHz, CD_2Cl_2, 25 °C).

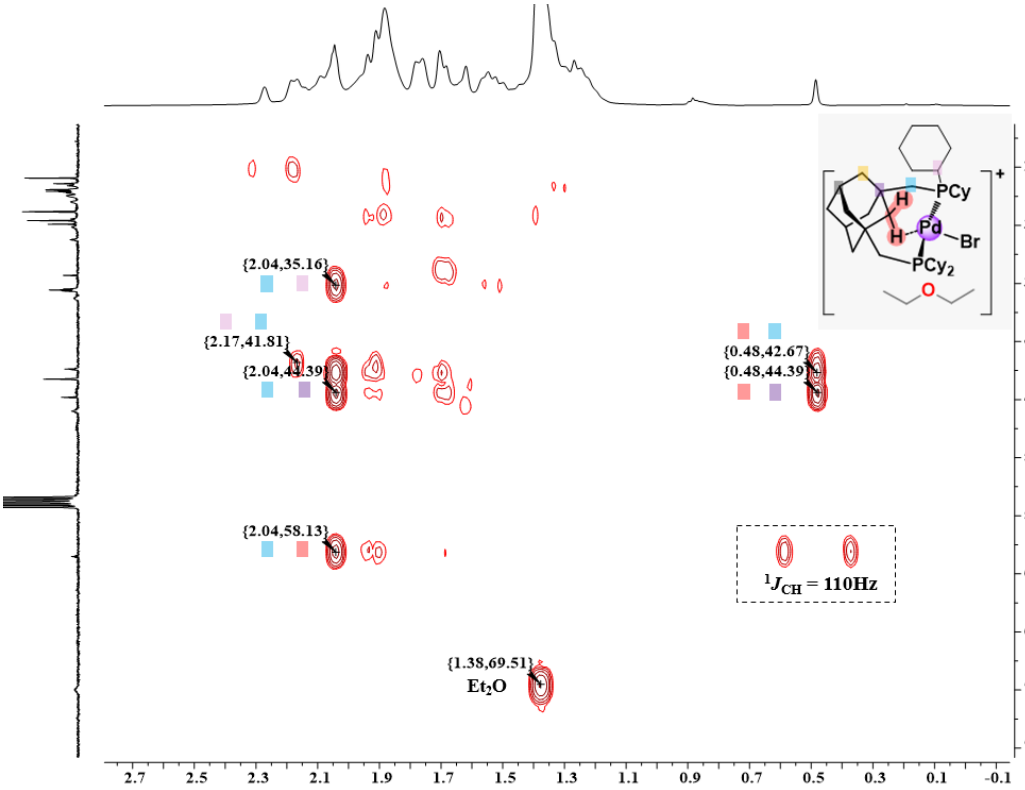

Figure S35- ${}^{1}H$-${}^{13}C$ HMBC NMR spectrum of the equilibrium mixture after protonation of **[PdBr]** with $H(Et_2O)_2[B(C_6F_5)_4]$ (500, 126 MHz, CD_2Cl_2, 25 °C). Peaks in the dashed box are residual coupling from ${}^{1}H$-${}^{13}C$ HSQC with ${}^{1}J_{CH}$ = 110 Hz

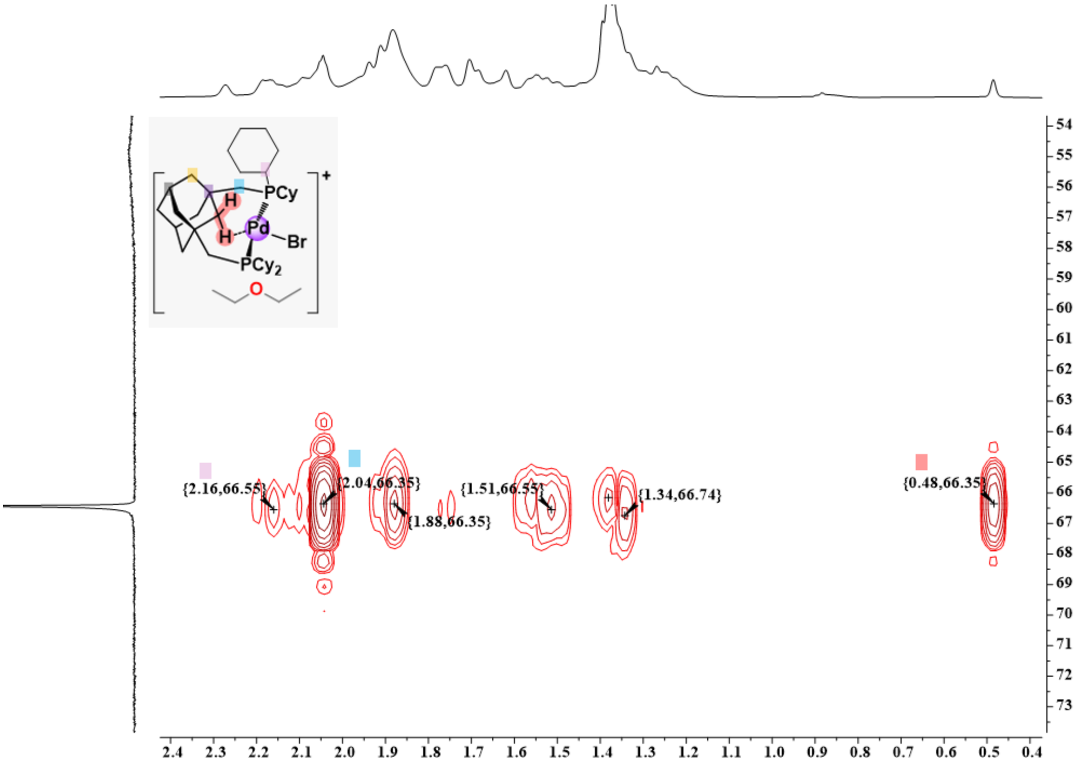

Figure S36- ${}^{1}H$-${}^{31}P$ HMBC NMR spectrum of the equilibrium mixture after protonation of **[PdBr]** with $H(Et_2O)_2[B(C_6F_5)_4]$ (500, 202 MHz, CD_2Cl_2, 25 °C).

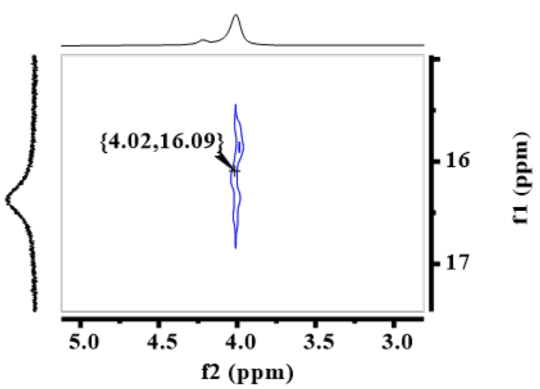

Figure S37- Section of ^{1}H-^{1}H NOESY (blue) NMR spectrum of the equilibrium mixture after protonation of **[PdBr]** with $H(Et_2O)_2[B(C_6F_5)_4]$ (500, 500 MHz, CD_2Cl_2, 25 °C, τ_m = 1.0 s, NS = 64), showing the Br*H*···OC*H*$_2$CH$_3$ cross peak from **[HBrH•2OEt$_2$]$^+$**.

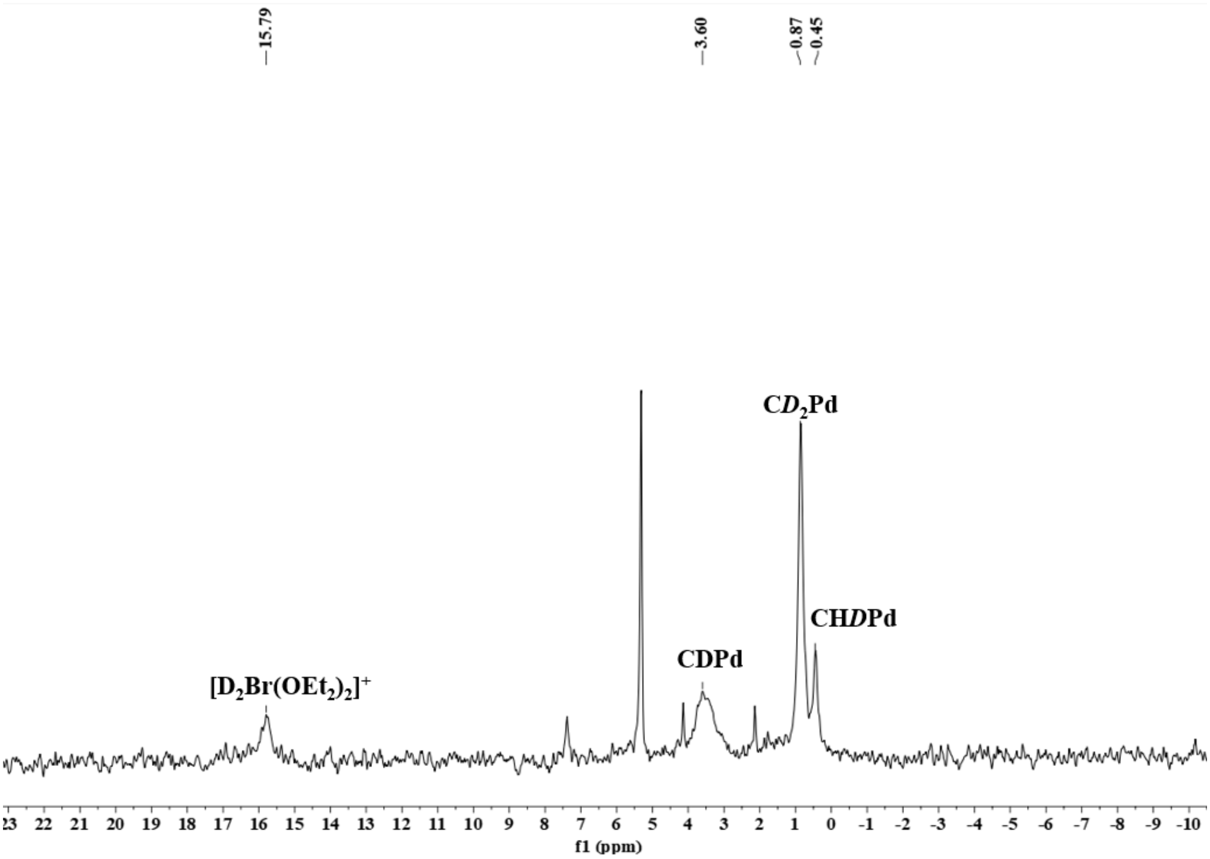

Figure S38- ^{2}H NMR spectrum of the equilibrium mixture after deuteration of **[PdBr]** with $D(Et_2O)_2[B(C_6F_5)_4]$ (77 MHz, CH_2Cl_2, 25 °C).

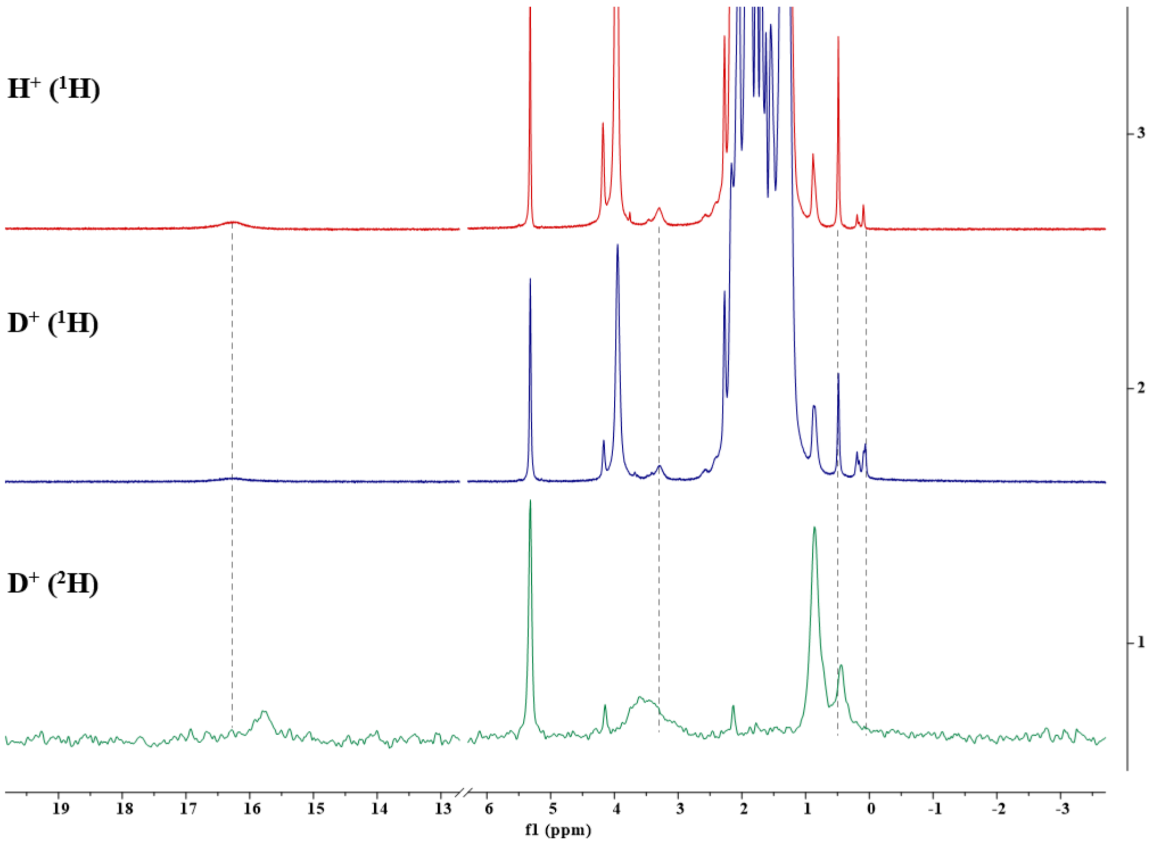

Figure S39- Comparison of ^{1}H NMR (top, red) spectrum of the equilibrium mixture after protonation of **[PdBr]** with $H(Et_2O)_2[B(C_6F_5)_4]$ (500MHz, CD_2Cl_2, 25 °C), ^{1}H NMR (middle, blue) spectrum of the equilibrium mixture after deuteration of **[PdBr]** with $D(Et_2O)_2[B(C_6F_5)_4]$ (500MHz, CD_2Cl_2, 25 °C) and ^{2}H NMR (bottom, green) spectrum of the equilibrium mixture after deuteration of **[PdBr]** with $D(Et_2O)_2[B(C_6F_5)_4]$ (500MHz, CH_2Cl_2, 25 °C).

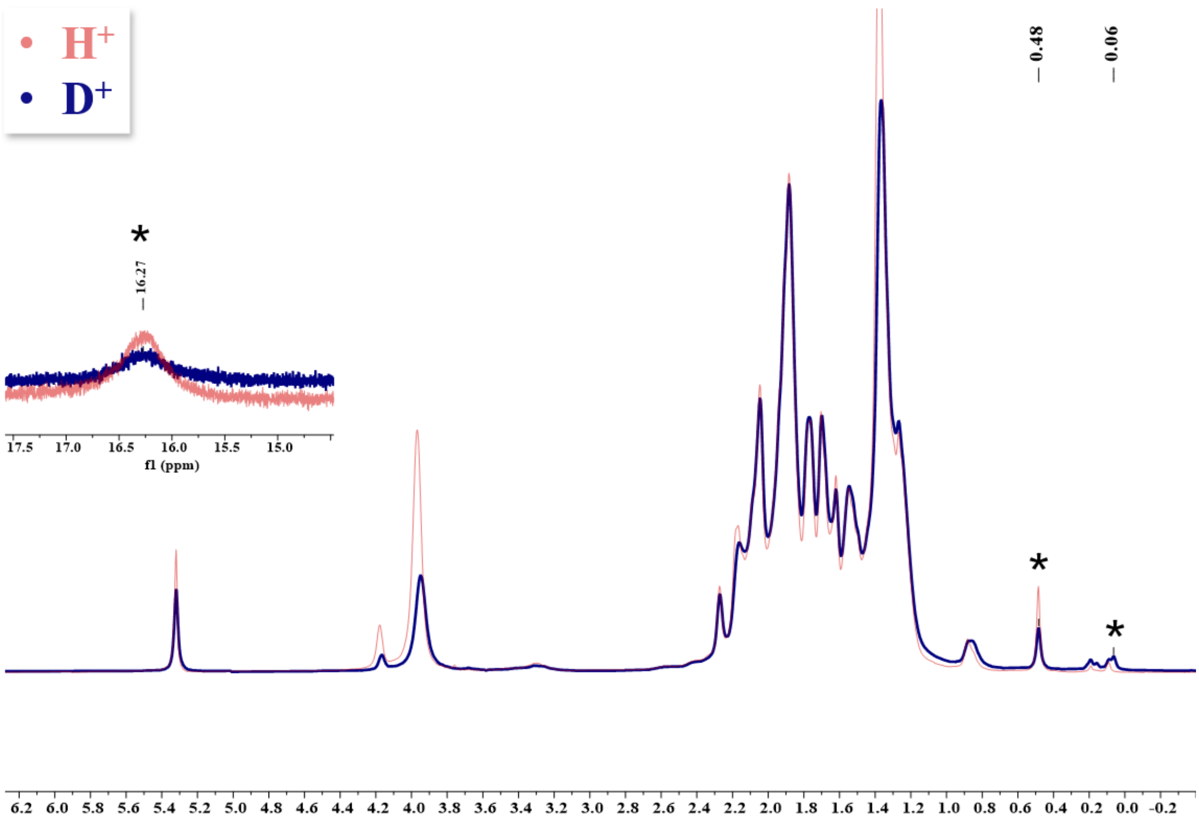

Figure S40- Comparison of ^{1}H NMR (red) spectrum of the equilibrium mixture after protonation of **[PdBr]** with $H(Et_2O)_2[B(C_6F_5)_4]$ (500MHz, CD_2Cl_2, 25 °C) and ^{1}H NMR (blue) spectrum of the equilibrium mixture after deuteration of **[PdBr]** with $D(Et_2O)_2[B(C_6F_5)_4]$ (500MHz, CD_2Cl_2, 25 °C). Peaks at 3.97 and 1.38 ppm are interacting Et_2O. The main differences between two spectrums are highlighted with asterisk.

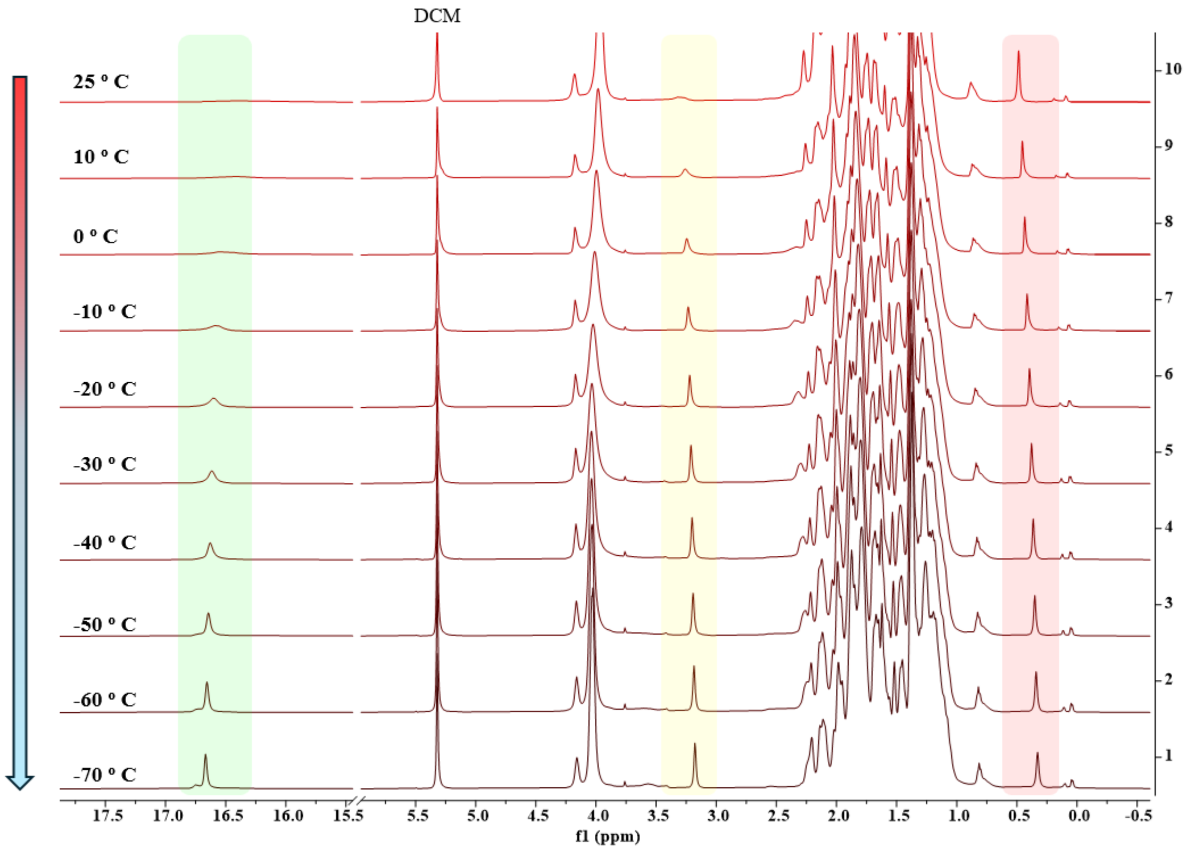

Figure S41- Variable temperature ^{1}H NMR spectrum of the equilibrium mixture after protonation of **[PdBr]** with $H(Et_2O)_2[B(C_6F_5)_4]$ (500 MHz, CD_2Cl_2). The chemical shifts of the exchanging protons from **[PdCH$_2$•Et$_2$O]**$^+$ (red), **[Pd-Br-Pd]**$^+$ (yellow), and the proposed bromonium salt **[HBrH•2OEt$_2$]**$^+$ (green) are labeled with shaded boxes corresponding to the colors in Figure 4a in main text.

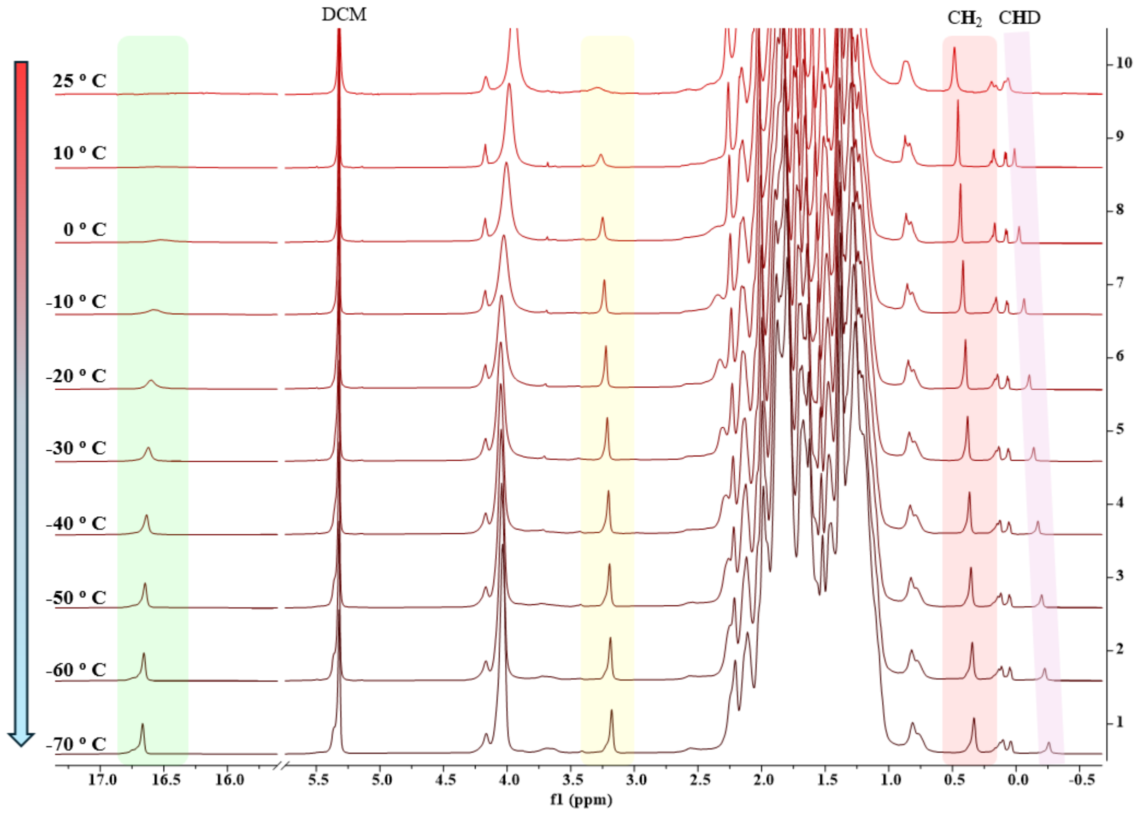

Figure S42- Variable temperature ^{1}H NMR spectrum of the equilibrium mixture after protonation of **[PdBr]** with $D(Et_2O)_2[B(C_6F_5)_4]$ (500 MHz, CD_2Cl_2). The chemical shifts of the exchanging protons from **$[PdCH_2 \bullet Et_2O]^+$** (red: Pd*CH2*; purple: Pd*CHD*), **$[Pd\text{-}Br\text{-}Pd]^+$** (yellow), and the proposed bromonium salt **$[HBrH \bullet 2OEt_2]^+$** (green) are labeled with shaded boxes corresponding to the colors in Figure 5 bottom in main text.

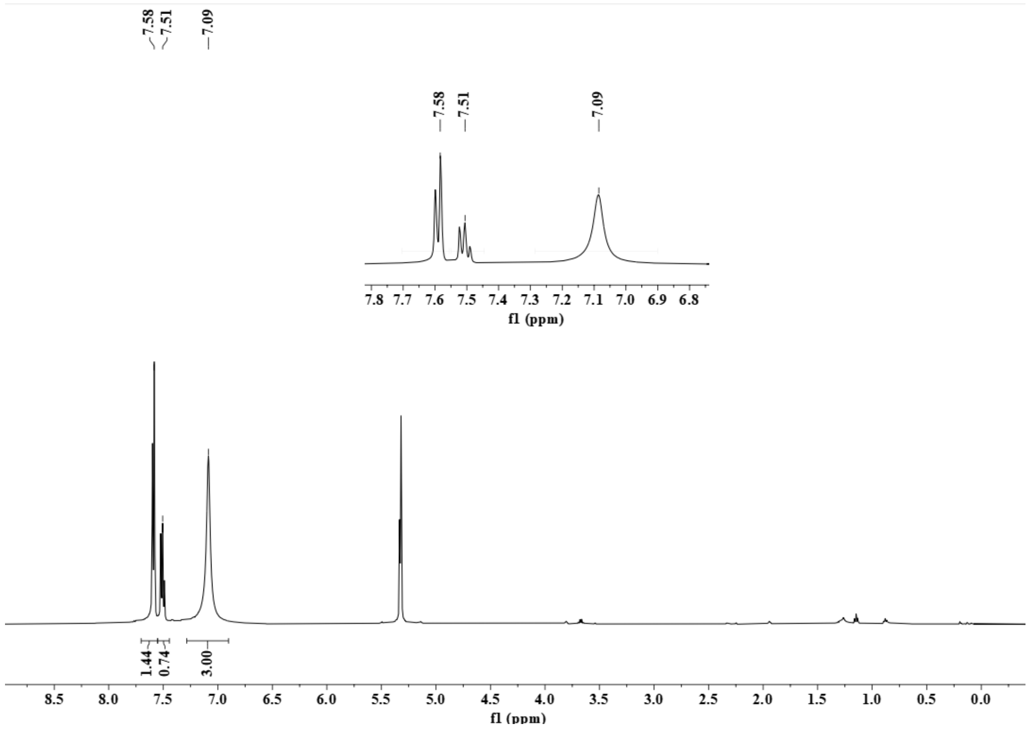

Figure S43- ^{1}H NMR spectrum of **$[H_3NAr^{Cl}][B(C_6F_5)_4]$** (500MHz, CD_2Cl_2, 25 °C). Signals at and 3.66, 1.26, 1.16 and 0.88 ppm are residual Et_2O and pentane in the NMR solvent.

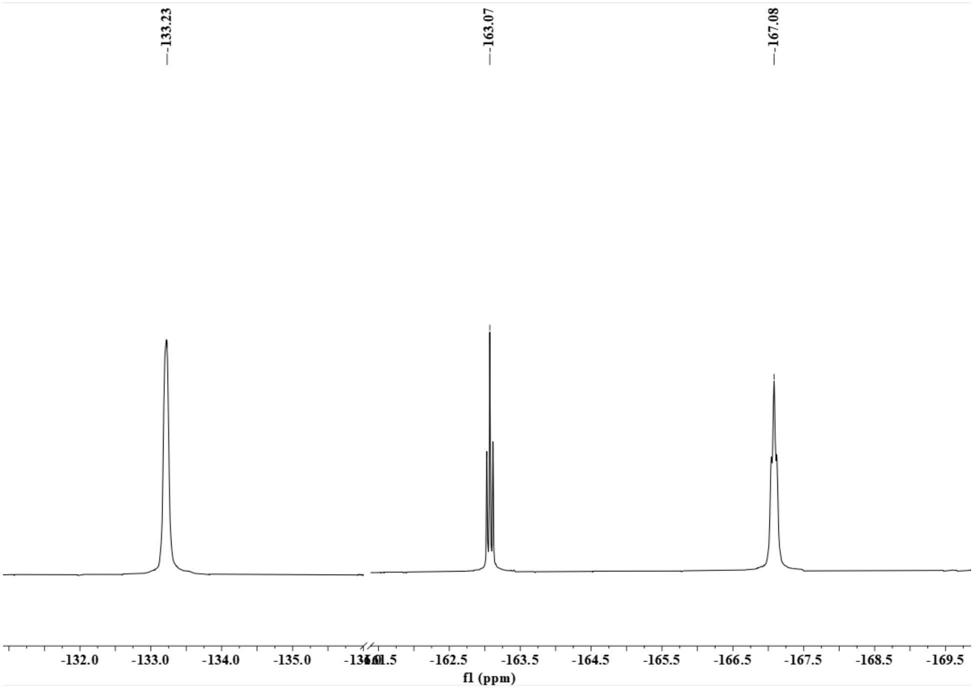

Figure S44- ^{19}F NMR spectrum of **[H_3NAr^{Cl}][$B(C_6F_5)_4$]** (471 MHz, CD_2Cl_2, 25 °C).

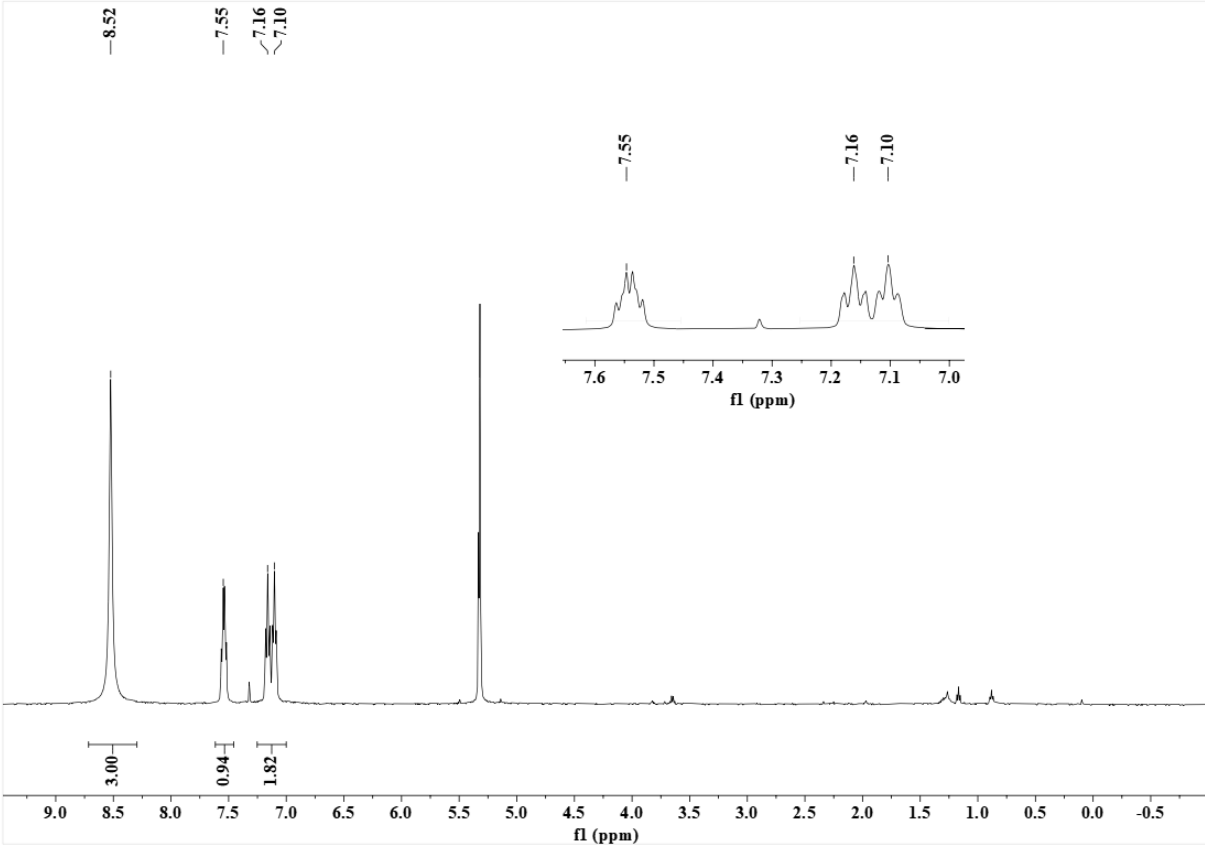

Figure S45- ^{1}H NMR spectrum of **[H_3NAr^{F}][$B(C_6F_5)_4$]** (500 MHz, CD_2Cl_2, 25 °C). Signal at 7.32ppm is impurity from NMR solvent. Signals at 3.66, 1.26, 1.16 and 0.88 ppm are Et_2O and pentane in the NMR solvent.

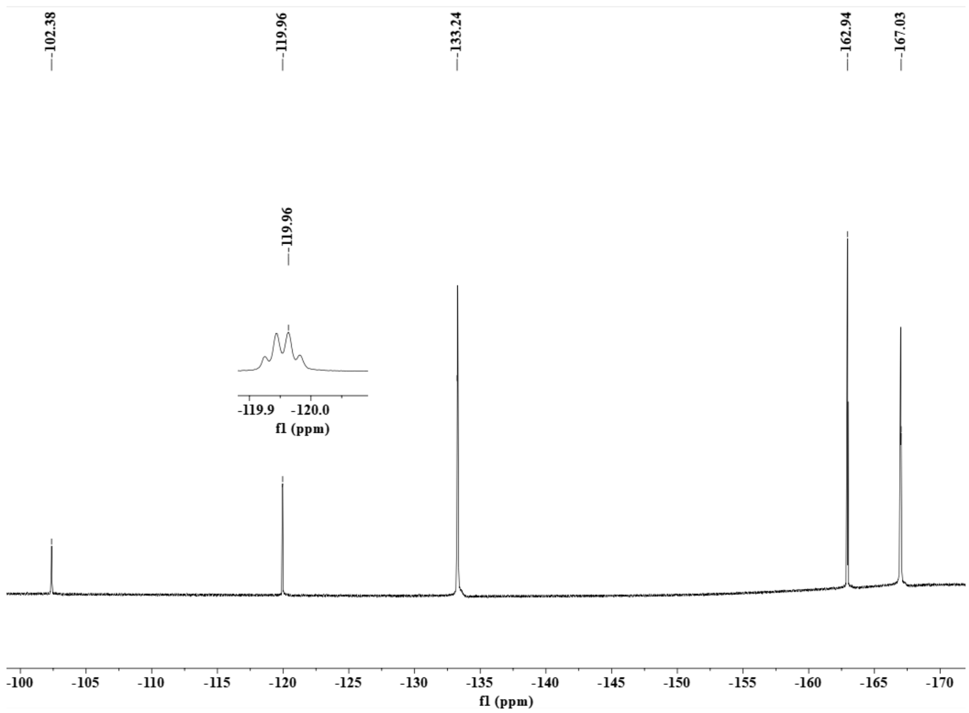

Figure S46- ^{19}F NMR spectrum of **[H_3NAr^F][$B(C_6F_5)_4$]** (471 MHz, CD_2Cl_2, 25 °C).

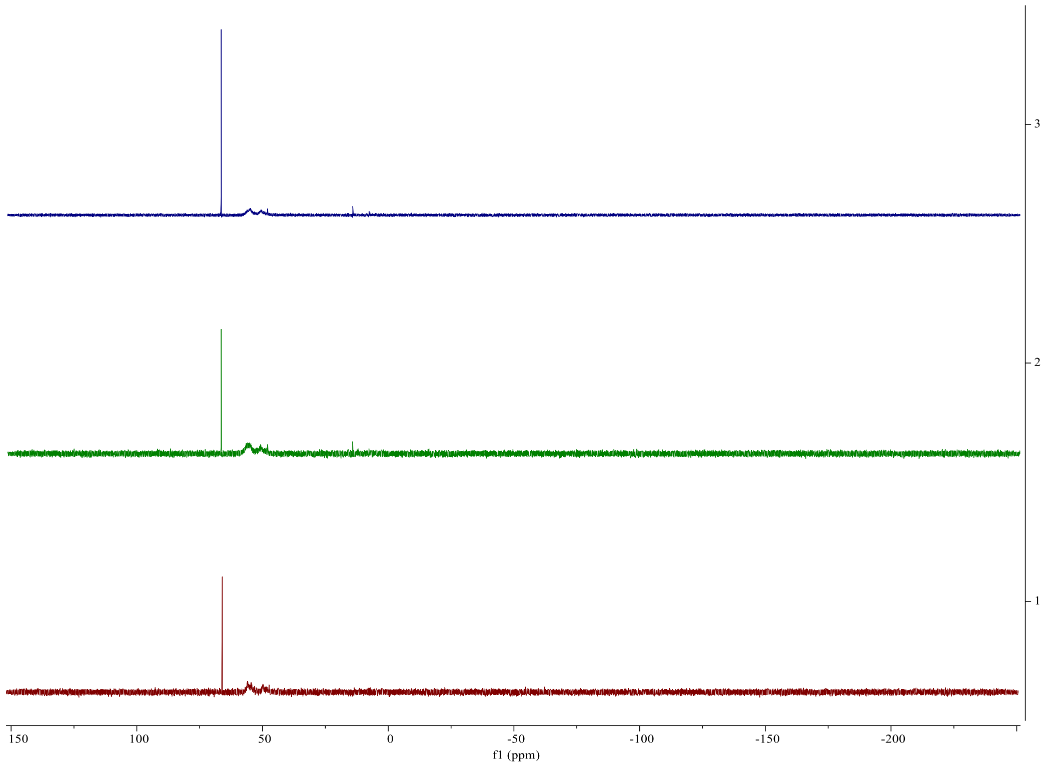

Figure S47- ^{31}P NMR spectrum of the reaction before (top, blue)) and after (middle, green) adding 2,4-dinitroaniline to the equilibrium mixture from protonation of **[PdBr]** with $H(Et_2O)_2[B(C_6F_5)_4]$ (202 MHz, CD_2Cl_2, 25 °C), and the ^{31}P NMR spectrum after drying and redissolving in DCE (bottom, red) (202 MHz, DCE with acetone-d_6 insert, 25 °C). No notable changes were observed in any spectra.

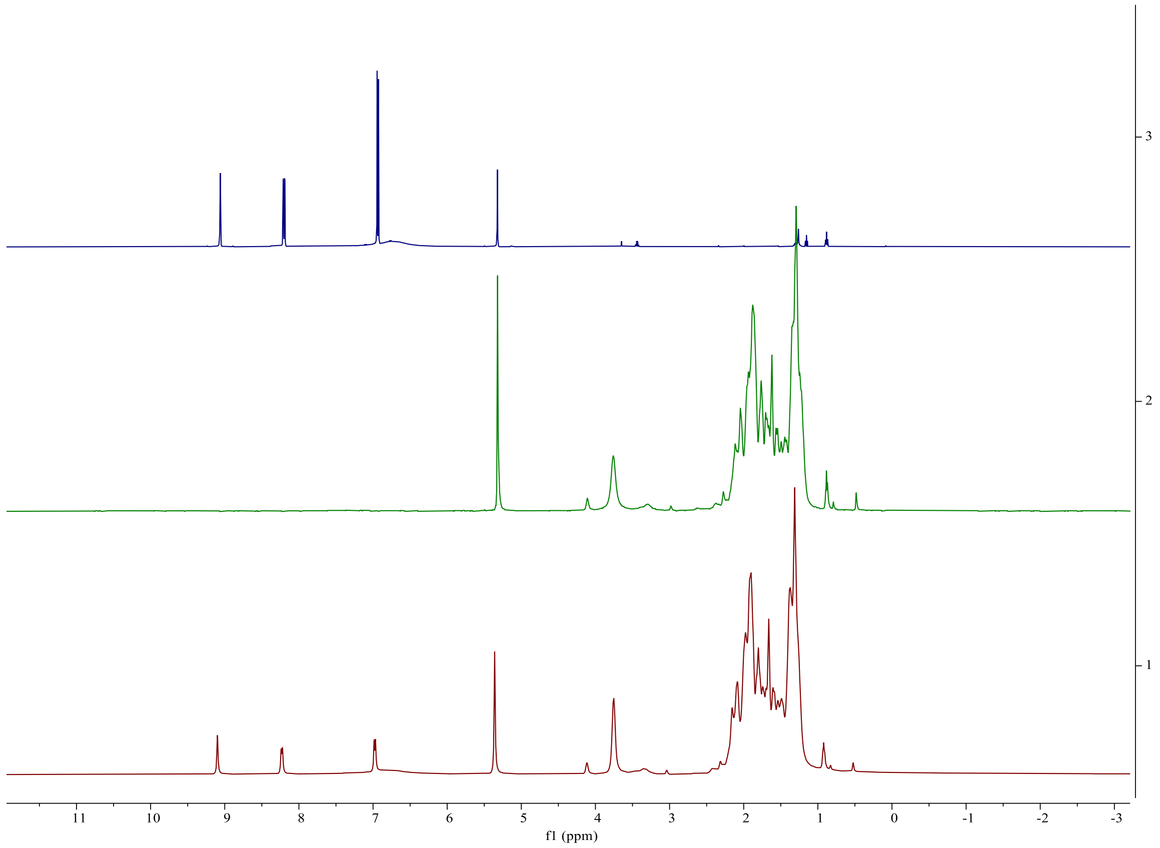

Figure S48- ^{1}H NMR spectrum of 2,4-dinitroaniline (top, blue), the reaction before (middle, green) and after (bottom, red) adding 2,4-dinitroaniline to the equilibrium mixture from protonation of **[PdBr]** with $H(Et_2O)_2[B(C_6F_5)_4]$ (500 MHz, CD_2Cl_2, 25 °C). No notable changes were observed in any spectra.

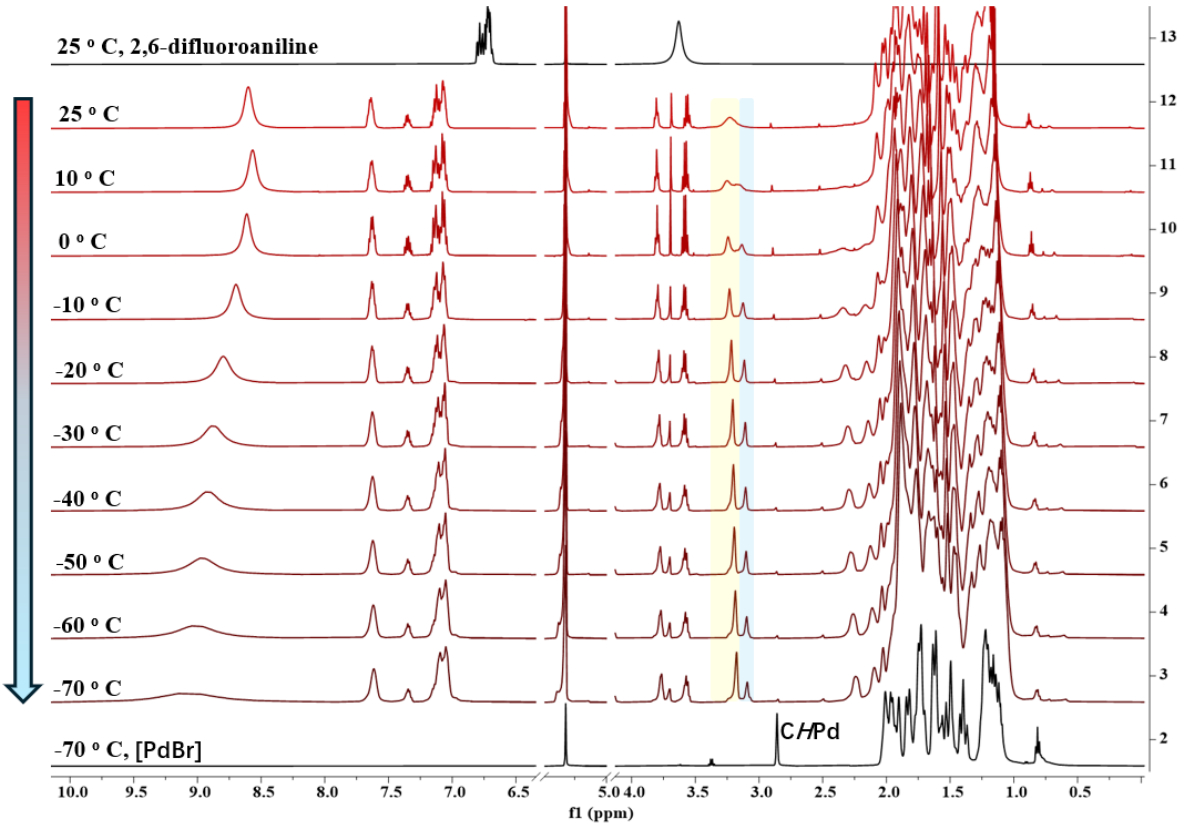

Figure S49- Variable temperature ^{1}H NMR (500 MHz, CD_2Cl_2) spectrum of the equilibrium mixture after protonation of **[PdBr]** with **[H$_3$NAr^F][B(C$_6$F$_5$)$_4$]**, compared to the ^{1}H NMR (500 MHz, CD_2Cl_2) spectrum of 2,6-difluoroaniline at 25 °C (top) and **[PdBr]** at -70 °C (bottom). The peak colored in yellow is the C*H*Pd peak in **[Pd-Br-Pd]**$^+$ and the peak colored in blue is the C*H*Pd peak in **[PdBr·H$_3$NAr^F]**$^+$. Signals at 7.35 ppm are residual PhF. Signals at 3.57 (q) and 3.80 (m) are residual free Et_2O and THF.

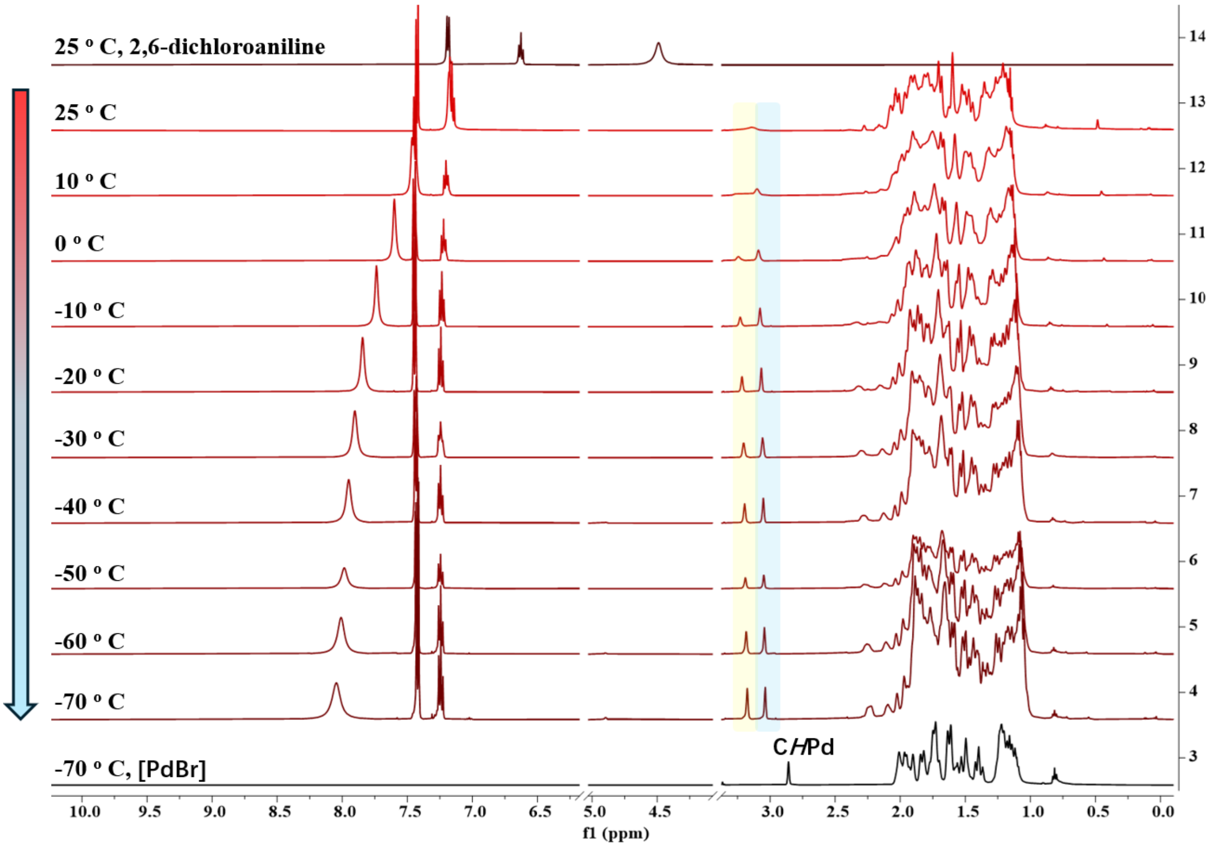

Figure S50- Variable temperature ^{1}H NMR (500 MHz, CD_2Cl_2) spectrum of the equilibrium mixture after protonation of **[PdBr]** with **$[H_3NAr^{Cl}][B(C_6F_5)_4]$**, compared to the ^{1}H NMR (500 MHz, CD_2Cl_2) spectrum of 2,6-dichloroaniline at 25 °C (top) and **[PdBr]** at -70 °C (bottom). The peak colored in yellow is the C*H*Pd peak in **$[Pd\text{-}Br\text{-}Pd]^+$** and the peak colored in blue is the C*H*Pd peak in **$[PdBr{\cdot}H_3NAr^{Cl}]^+$**.

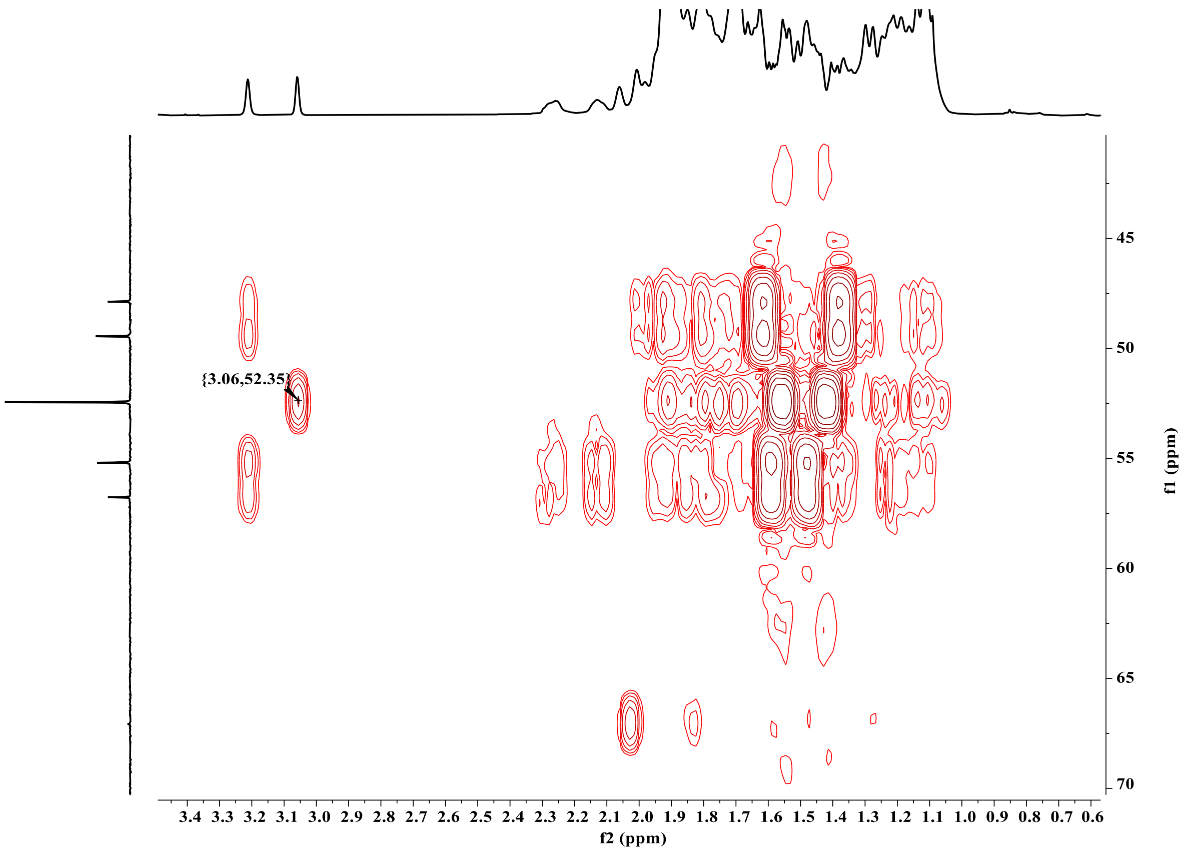

Figure S51- ^{1}H-^{31}P HMBC NMR spectrum of the equilibrium mixture after protonation of **[PdBr]** with **[H$_3$NAr^{Cl}][B(C$_6$F$_5$)$_4$]** (500, 202 MHz, CD_2Cl_2, -70 °C), showing the cross peak of C*H*Pd*P* from **[PdBr·H$_3$NAr^{Cl}]$^+$**.

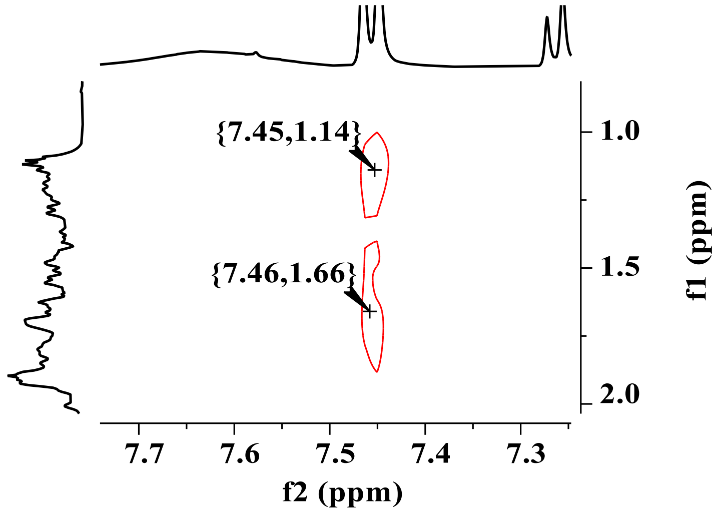

Figure S52- Section of ^{1}H-^{1}H NOESY NMR spectrum of the equilibrium mixture after protonation of **[PdBr]** with **[H$_3$NAr^{Cl}][B(C$_6$F$_5$)$_4$]** (500, 500 MHz, CD_2Cl_2, -70 °C, τ_m = 0.5 s, NS = 32), showing the cross peak from **[PdBr·H$_3$NAr^{Cl}]$^+$**.

DOSY NMR and External Calibration Curve Analysis

We opted to use a DOSY NMR calibration curve method developed by Stalke.[11-13] The correlation between diffusion coefficient and molecular weight can be described by the following equation:

$$D = K \cdot MW_{det}^{\alpha} \tag{S15}$$

where D is diffusion coefficient, MW_{det} is the determined molecular weight, K and α are external calibration curve (ECC) fit parameters. Using the existing ECCs led to poor fits with the molecules in this study because the published ECCs do not contain heavy atoms such as Br and Pd, which have a disproportionate effect on molecular density.[12] Thus, it was necessary to construct a new heavy atom ECC using molecules of comparable molar densities and geometries. This was achieved by measuring D in CD_2Cl_2 and using $\log D_{\mathrm{ref,fix}} = -8.5190$ with the merged curve data (ECC_{Merge}^{CD2Cl2}) where $\log K = -7.55$ and $\alpha = -0.535$.[13] The following equations were used to create the calibration curves:[13]

$$logD_{x,norm} = logD_{ref,fix} - logD_{ref} + logD_x \tag{S16}$$

$$MW_{det} = 10^{\left(\frac{log_{Dx,norm} - logK}{\alpha}\right)} \tag{S17}$$

In equation S16, D_{ref} is the diffusion coefficient for solvent (CH_2Cl_2), D_{x} is the diffusion coefficient for the analyte, and $D_{\mathrm{x,norm}}$ is the normalized diffusion coefficient. The following six molecules were used to construct the ECC: 1,3-bis(bromomethyl)adamantane (**AdBr**), 1,3-bis(dicyclohexylphosphino)adamantane (**PCH$_2$P**), **[H$_3$NAr^F][B(C$_6$F$_5$)$_4$]**, **[H$_3$NAr^{Cl}][B(C$_6$F$_5$)$_4$]**, **[PdBr]**, and **[Pd-Br-Pd]$^+$**. The determined molecular weights (MW_{det}) were plotted against the actual molecular weight (MW_{act}) and used to construct a new ECC (Table S13 and Figure S53). See the Experimental section for spectrometer information, DOSY NMR experimental parameters, and sample preparation details.

Table S13- Analyte diffusion coefficients (D_{x}) and determined molecular weight (MW_{det}) for the molecules used in the heavy atom ECC.

	D_{ref} ($m^2/s\times10^{-9}$)	D_{x} ($m^2/s\times10^{-9}$)	log(D_{ref})	log(D_{x})	Log ($D_{\mathrm{x,norm}}$)	MW_{det} (g/mol)	MW_{act} (g/mol)
**[H$_3$NAr^F]$^+$**	3.26 ± 0.16	1.87 ± 0.21	-8.48	-8.73	-8.76	183 ± 21	130
**[H$_3$NAr^{Cl}]$^+$**	3.03 ± 0.03	1.55 ± 0.08	-8.52	-8.81	-8.81	226 ± 12	163
**AdBr**	3.68 ± 0.25	1.33 ± 0.05	-8.43	-8.88	-8.96	435 ± 16	322
**PCH$_2$P**	3.56 ± 0.34	1.09 ± 0.08	-8.45	-8.96	-9.03	590 ± 43	557
**[PdBr]**	3.77 ± 0.42	0.81 ± 0.13	-8.42	-9.09	-9.19	1143 ± 183	741
**[Pd-Br-Pd]$^+$**	3.98 ± 0.14	0.69 ± 0.05	-8.40	-9.16	-9.28	1691 ± 123	1403

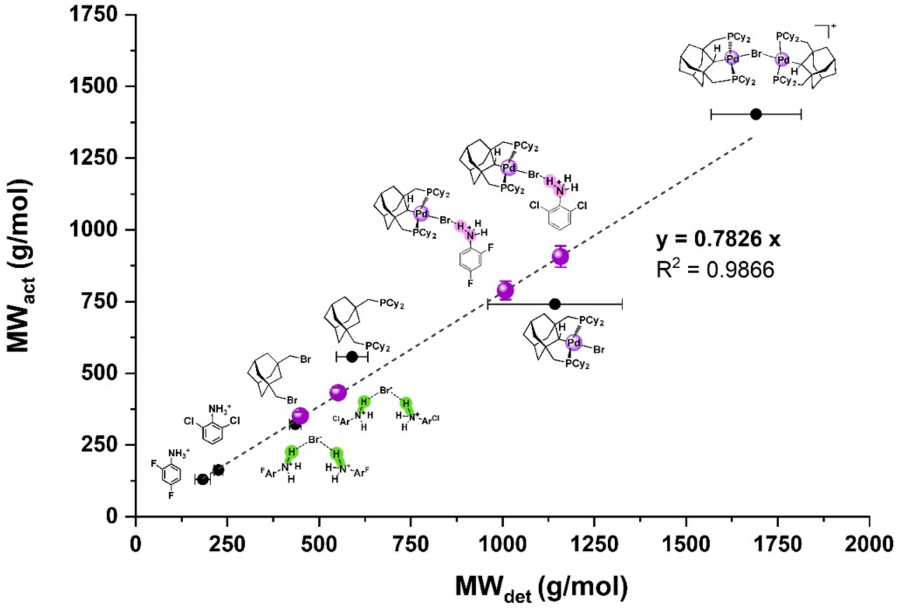

Figure S53- Heavy atom DOSY calibration curve using ECC data from Table S13 (black dots). The corrected molecular weights (MW_{corr}) of the putative **[H$_2$Br(H$_2$NAr^F)$_2$]$^+$**, **[PdBr·H$_3$NAr^F]$^+$**, **[H$_2$Br(H$_2$NAr^{Cl})$_2$]$^+$** and **[PdBr·H$_3$NAr^{Cl}]$^+$** are shown on the calibration curve in purple.

From the above calibration curve, it is seen that $MW_{act} = 0.7826 \cdot MW_{det}$ which is then used to determine the calculated solution-phase molecular weight (MW_{cal}) of the K_2 adducts **[H$_2$Br(H$_2$NAr^F)$_2$]$^+$** and **[PdBr·H$_3$NAr^F]$^+$**, and K_3 adducts **[H$_2$Br(H$_2$NAr^{Cl})$_2$]$^+$** and **[PdBr·H$_3$NAr^{Cl}]$^+$**. Two independently prepared equilibrium mixtures were prepared in CD_2Cl_2 and diffusion coefficients D_x were measured for the aromatic C-H resonances at 7.23 ppm and 7.38 ppm (Table S14). Using the slope from the external calibration curve, the calculated molecular weights are in good agreement with the proposed solution phase molecules.

Table S14- Analyte diffusion coefficient (D_x) and molecular weight data for the aromatic resonances in equilibrium K_2 and K_3 in CD_2Cl_2.

δ (ppm)		D_{ref} ($m^2/s\times10^{-9}$)	D_x ($m^2/s\times10^{-9}$)	log(D_{ref})	log(D_x)	log($D_{x,norm}$)	MW_{det} (g/mol)	MW_{cal} (g/mol)
***K*₂**	**7.23**	3.21	1.14	-8.49	-8.94	-8.97	449	**351±14**
	**7.23**	3.21	0.739	-8.49	-9.13	-9.16	1020	**799±33**
***K*₃**	**7.38**	2.93	0.931	-8.53	-9.03	-9.02	552	**432±17**
	**7.38**	2.93	0.626	-8.53	-9.20	-9.19	1158	**906±37**

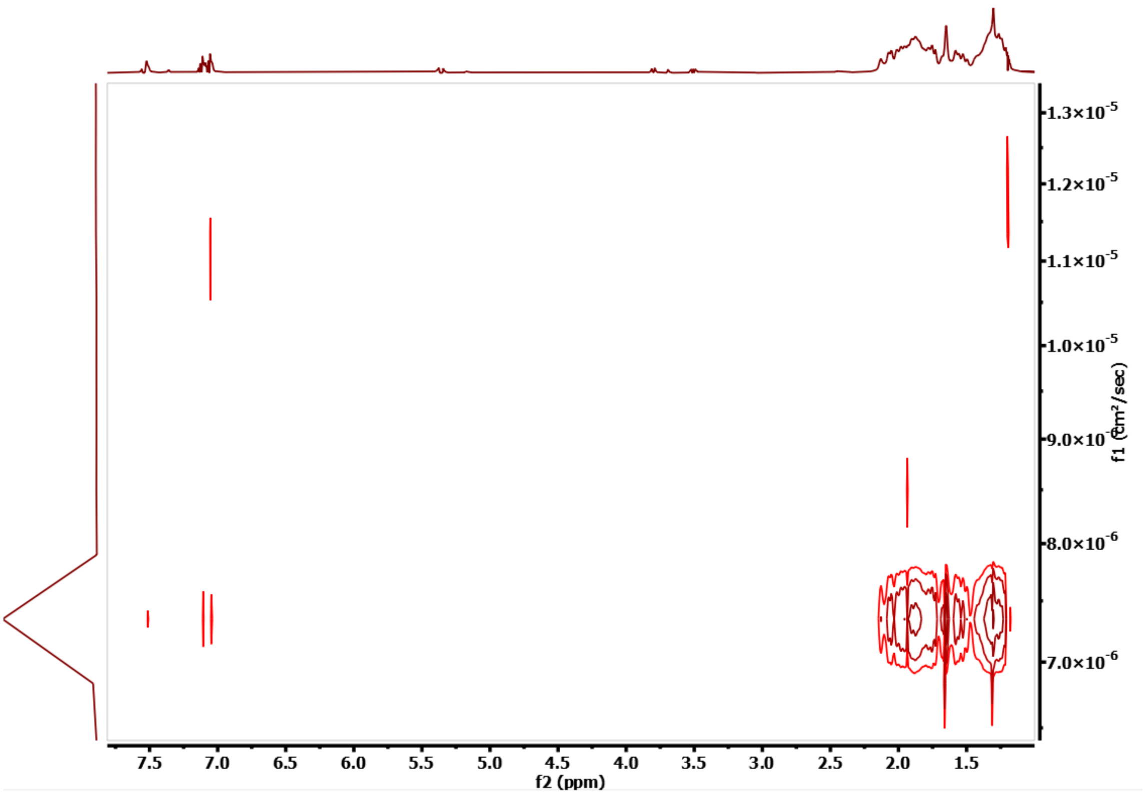

Figure S54- ILT-DOSY plots of the equilibrium mixture after addition of **[PdBr]** with **[H_3NAr^F][$B(C_6F_5)_4$]**.

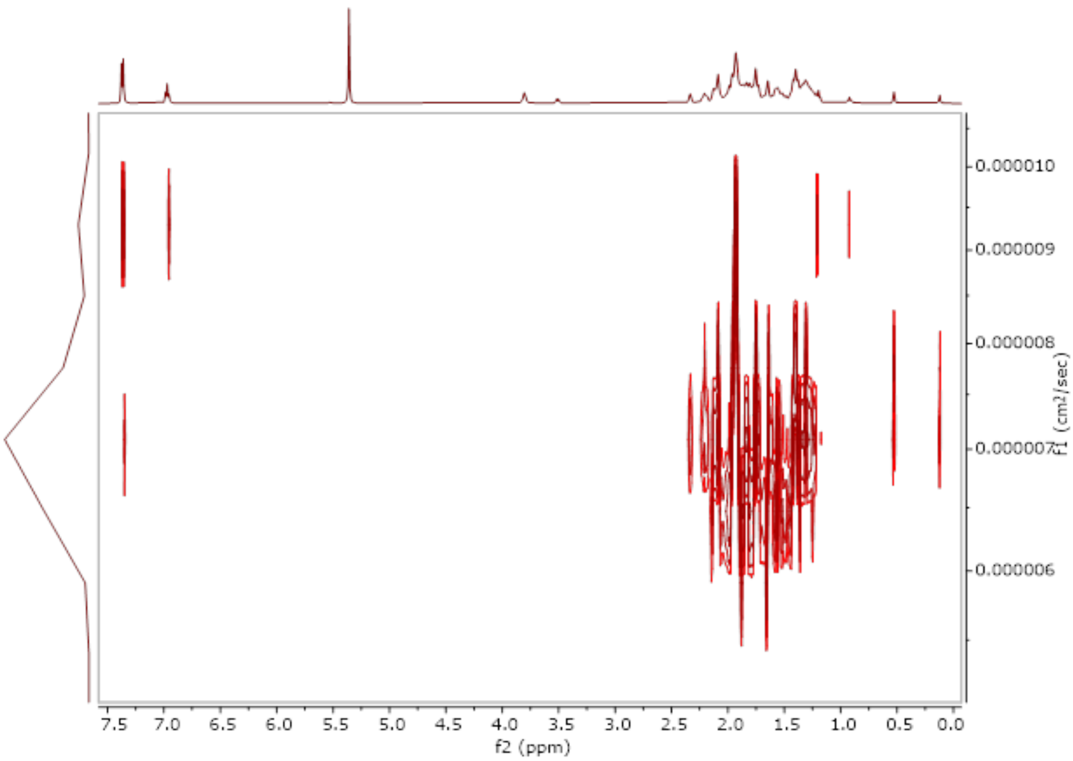

Figure S55- ILT-DOSY plots of the equilibrium mixture after addition of **[PdBr]** with **[H_3NAr^{Cl}][$B(C_6F_5)_4$]**.

Electrochemistry

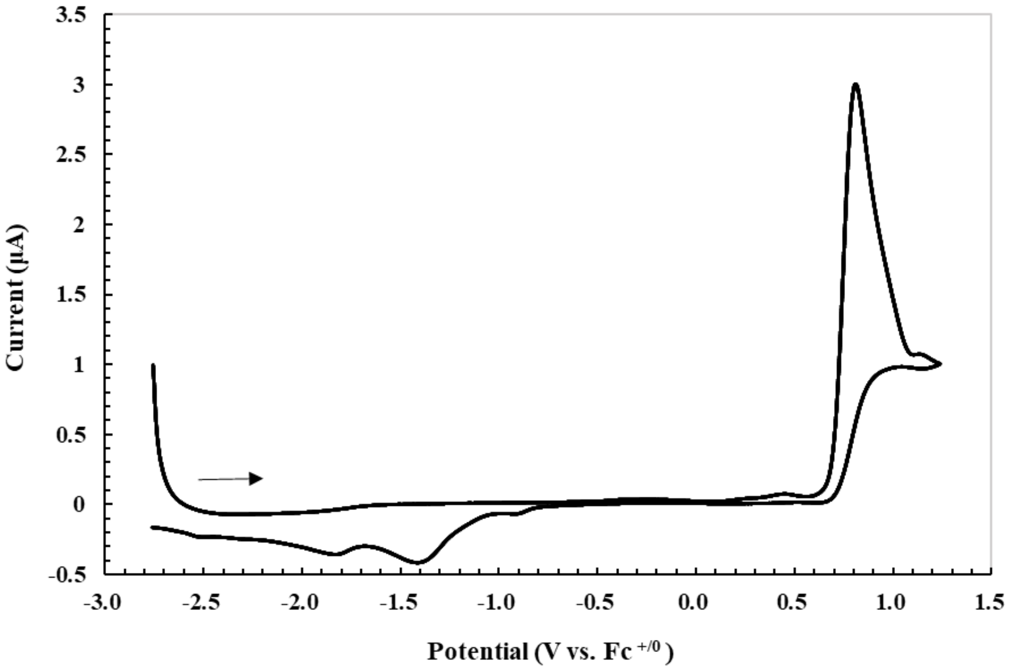

Figure S56- Cyclic voltammograms of **[PdBr]** in THF after background subtraction. Conditions: 1 mM analyte, 0.2 M [$^{n}Bu_4N$][$B(C_6F_5)_4$], scan rate 100 mV/s.

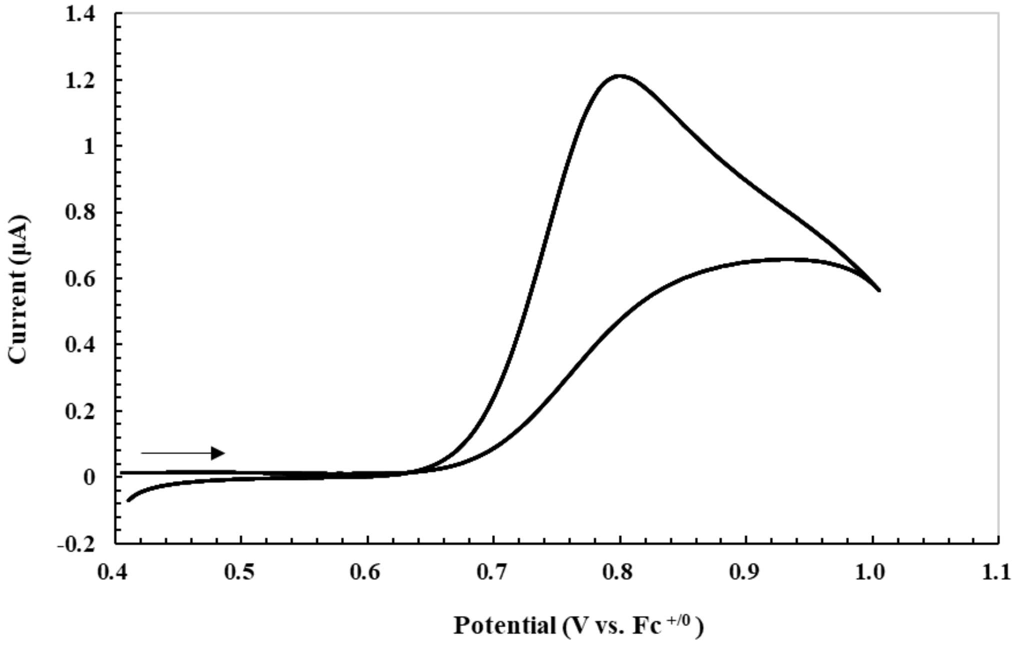

Figure S57- Cyclic voltammograms of **[PdBr]** in THF after background subtraction. Conditions: 1 mM analyte, 0.2 M [$^{n}Bu_4N$][$B(C_6F_5)_4$], scan rate 100 mV/s. E_{pa} = 0.810 V vs. $Fc^{+/0}$.

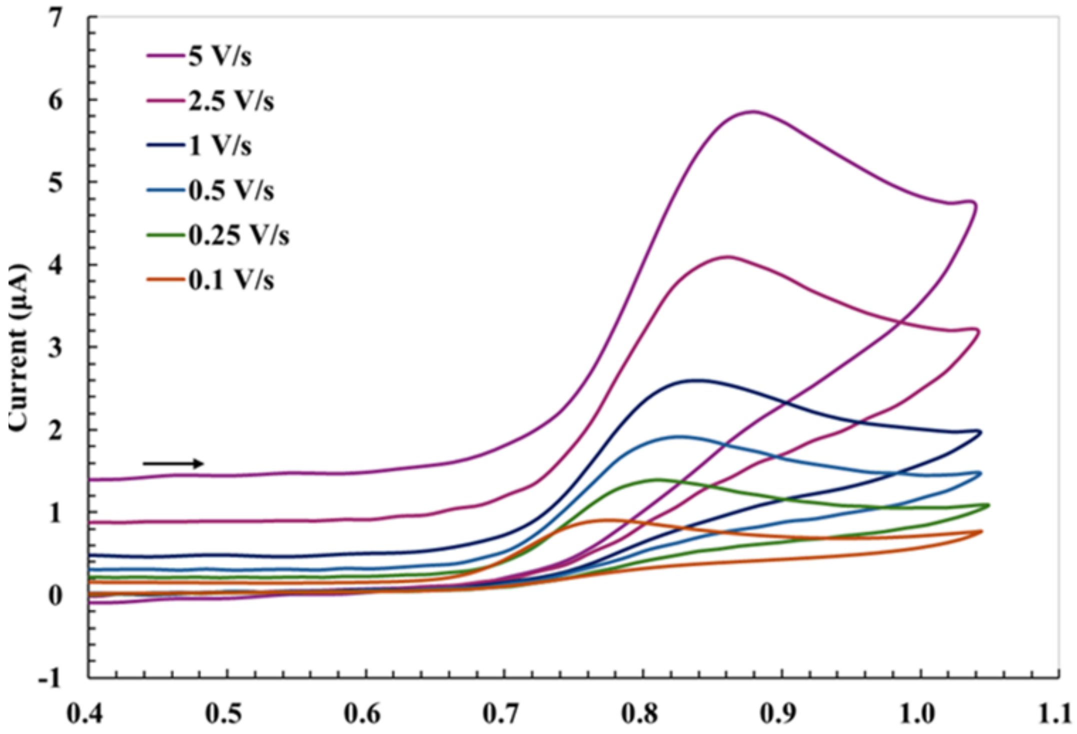

Figure S58- Cyclic voltammograms of **[PdBr]** in THF at different scan rates. Conditions: 1 mM analyte, 0.2 M [^{n}Bu$_4$N][B(C_6F_5)$_4$].

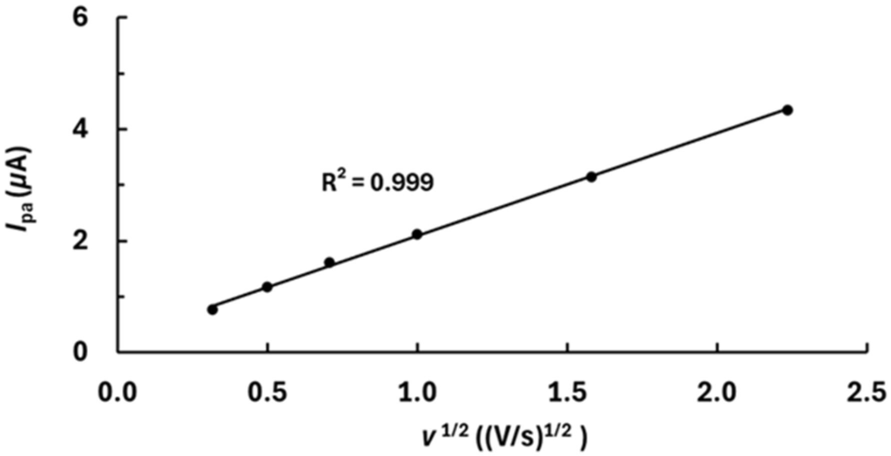

Figure S59- Anodic peak current (I_{pa}) *vs.* the square root of scan rate $v^{1/2}$ of **[PdBr]** in THF. Conditions: 1 mM analyte, 0.2 M [^{n}Bu$_4$N][B(C_6F_5)$_4$].

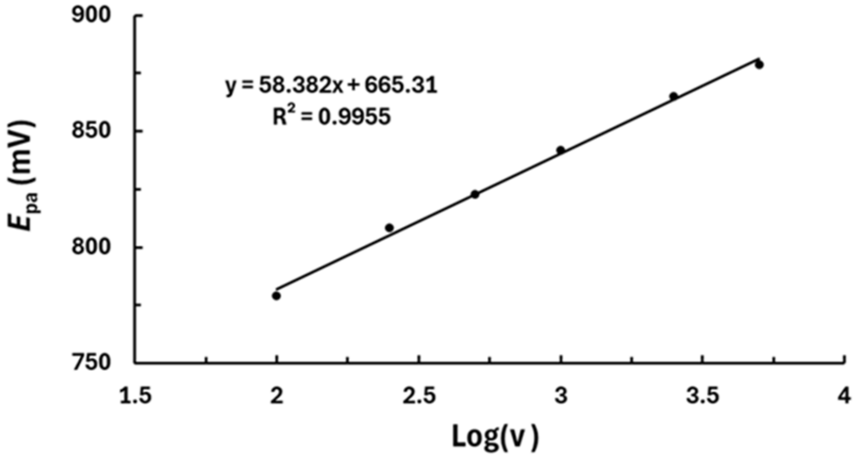

Figure S60- Anodic peak potential (E_{pa}) *vs.* the logarithm of scan rate log(v) of **[PdBr]** in THF. Conditions: 1 mM analyte, 0.2 M [$^{n}Bu_4N$][$B(C_6F_5)_4$].

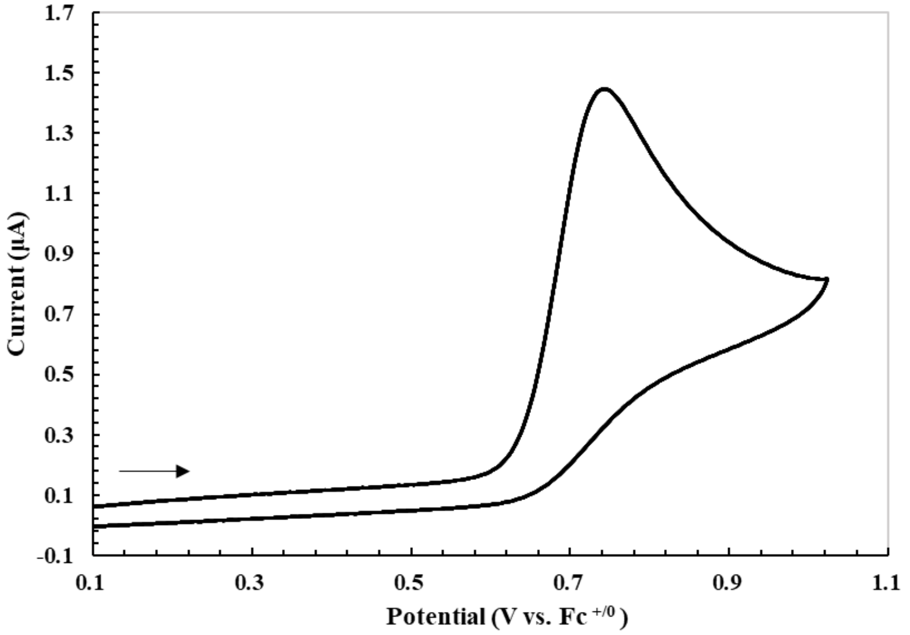

Figure S61- Cyclic voltammograms of **[PdBr]** in PhF. Conditions: 1 mM analyte, 0.2 M [$^{n}Bu_4N$][$B(C_6F_5)_4$], scan rate 100 mV/s. E_{pa} = 0.762 V vs. $Fc^{+/0}$.

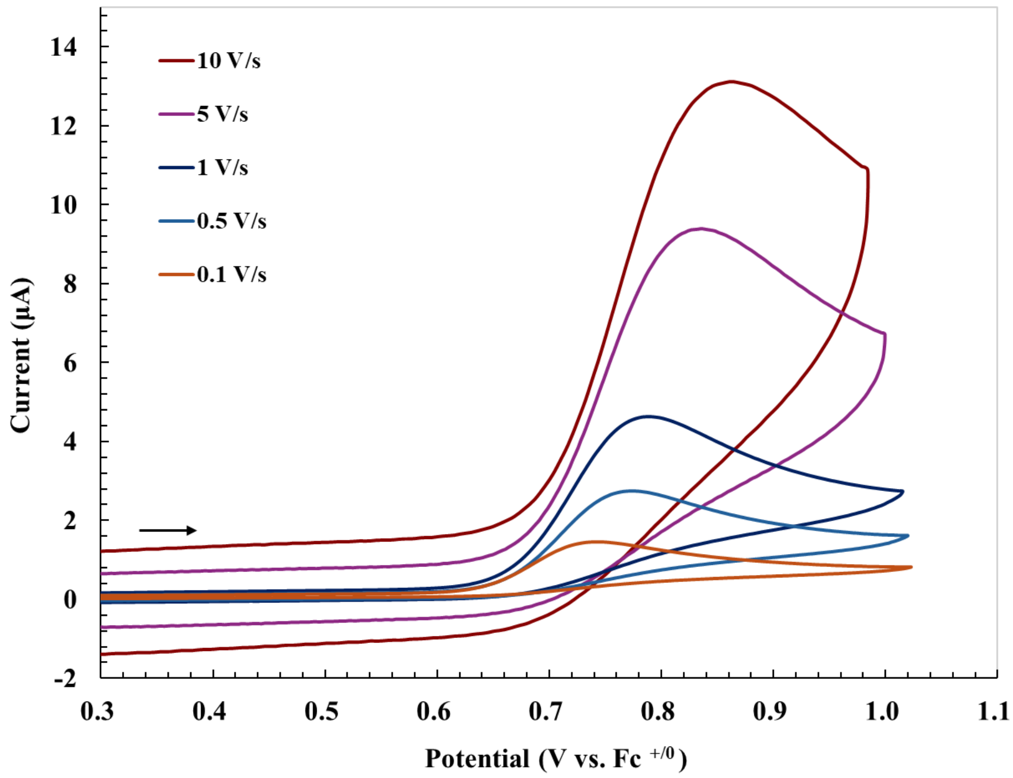

Figure S62- Cyclic voltammograms of **[PdBr]** in PhF at different scan rates. Conditions: 1 mM analyte, 0.2 M [^{n}Bu$_4$N][B(C$_6$F$_5$)$_4$].

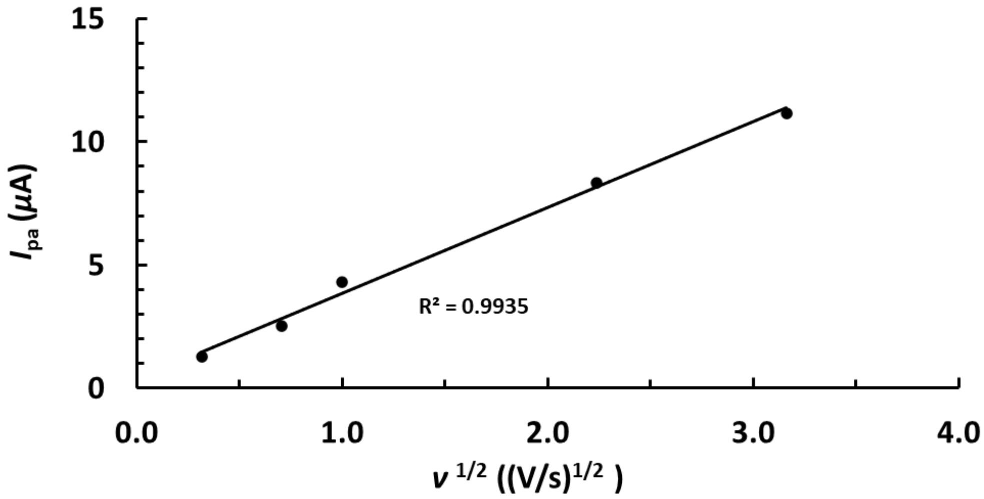

Figure S63- Anodic peak current (I_{pa}) *vs.* the square root of scan rate $v^{1/2}$ of **[PdBr]** in PhF. Conditions: 1 mM analyte, 0.2 M [^{n}Bu$_4$N][B(C$_6$F$_5$)$_4$].

X-Ray Crystallographic Structures

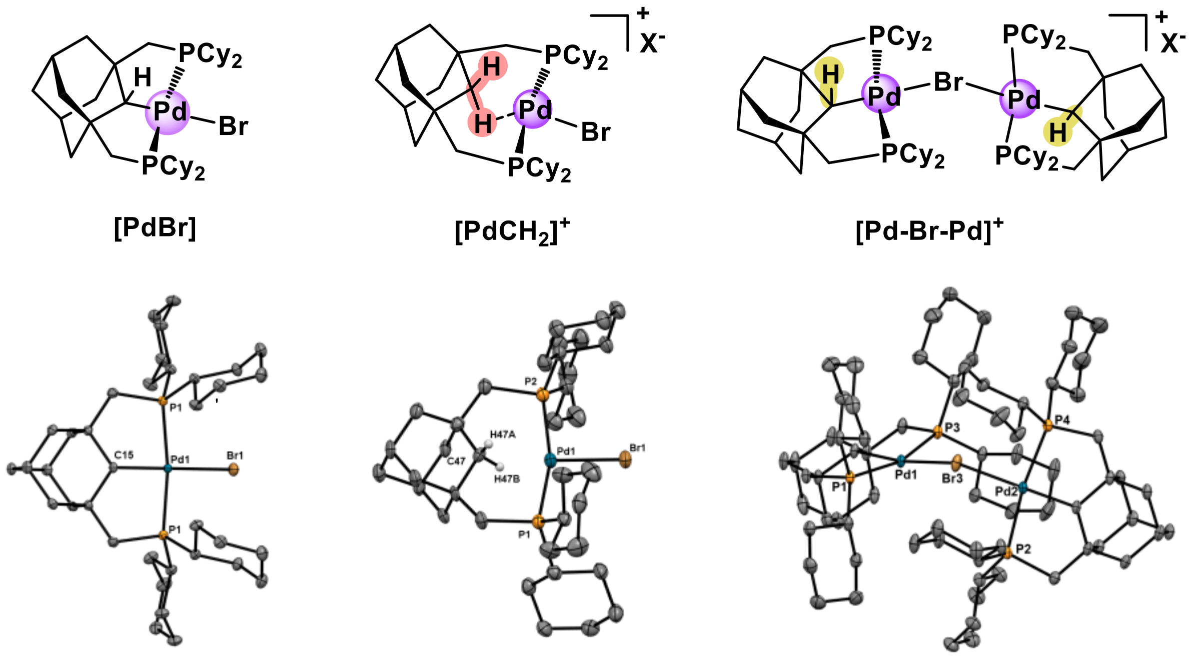

Figure S64- Molecular structures of **[PdBr]** (left), **[PdCH₂]**$^{+}$ (middle), and **[Pd-Br-Pd]**$^{+}$ (right). (50% probability ellipsoids). Most hydrogen atoms, co-crystallized solvent, and $[B(C_6F_5)_4]^-$ are omitted for clarity. $X^- = B(C_6F_5)_4^-$.

References

(1) Williams, D. B. G.; Lawton, M. Drying of Organic Solvents: Quantitative Evaluation of the Efficiency of Several Desiccants. *J. Org. Chem.* **2010**, *75* (24), 8351-8354.

(2) Averina, E. B.; Sedenkova, K. N.; Bakhtin, S. G.; Grishin, Y. K.; Kutateladze, A. G.; Roznyatovsky, V. A.; Rybakov, V. B.; Butov, G. M.; Kuznetsova, T. S.; Zefirov, N. S. symm-Tetramethylenecyclooctane: en route to polyspirocycles. *J Org Chem* **2014**, *79* (17), 8163-8170.

(3) Gerber, R.; Blacque, O.; Frech, C. M. Suzuki Cross-Coupling Reactions Catalyzed by an Aliphatic Phosphine-Based Pincer Complex of Palladium: Evidence for a Molecular Mechanism. *ChemCatChem* **2009**, *1* (3), 393-400.

(4) Heiden, Z. M.; Chen, S.; Mock, M. T.; Dougherty, W. G.; Kassel, W. S.; Rousseau, R.; Bullock, R. M. Protonation of Ferrous Dinitrogen Complexes Containing a Diphosphine Ligand with a Pendent Amine. *Inorg. Chem.* **2013**, *52* (7), 4026-4039.

(5) LeSuer, R. J.; Geiger, W. E. Improved Electrochemistry in Low-Polarity Media Using Tetrakis(pentafluorophenyl)borate Salts as Supporting Electrolytes. *Angew. Chem. Int. Ed.* **2000**, *39* (1), 248-250.

(6) Fulmer, G. R.; Miller, A. J. M.; Sherden, N. H.; Gottlieb, H. E.; Nudelman, A.; Stoltz, B. M.; Bercaw, J. E.; Goldberg, K. I. NMR Chemical Shifts of Trace Impurities: Common Laboratory Solvents, Organics, and Gases in Deuterated Solvents Relevant to the Organometallic Chemist. *Organometallics* **2010**, *29* (9), 2176-2179.

(7) Zimmer, K. D.; Shoemaker, R.; Ruminski, R. R. Synthesis and characterization of a fluxional Re(I)

carbonyl complex fac-[Re(CO)3(dpop′)Cl] with the nominally tri-dentate ligand dipyrido(2,3-a:3′,2′-j)phenazine (dpop′). *Inorganica Chimica Acta* **2006**, *359* (5), 1478-1484.
(8) Spek, A. L. Structure validation in chemical crystallography. *Acta Crystallogr., Sect. D* **2009**, *65* (2), 148-155.
(9) Tshepelevitsh, S.; Kütt, A.; Lõkov, M.; Kaljurand, I.; Saame, J.; Heering, A.; Plieger, P. G.; Vianello, R.; Leito, I. On the Basicity of Organic Bases in Different Media. *Eur. J. Org. Chem.* **2019**, *2019* (40), 6735-6748.
(10) Kaupmees, K.; Järviste, R.; Leito, I. Basicity of Very Weak Bases in 1,2-Dichloroethane. *Chem. Eur. J.* **2016**, *22* (48), 17445-17449.
(11) Neufeld, R.; John, M.; Stalke, D. The Donor-Base-Free Aggregation of Lithium Diisopropyl Amide in Hydrocarbons Revealed by a DOSY Method. *Angew. Chem. Int. Ed.* **2015**, *54* (24), 6994-6998.
(12) Neufeld, R.; Stalke, D. Accurate molecular weight determination of small molecules via DOSY-NMR by using external calibration curves with normalized diffusion coefficients. *Chem. Sci.* **2015**, *6*, 3354-3364.
(13) Bachmann, S.; Neufeld, R.; Dzemski, M.; Stalke, D. New External Calibration Curves (ECCs) for the Estimation of Molecular Weights in Various Common NMR Solvents. *Chem. Eur. J.* **2016**, *22* (25), 8462-8465.
(14) Rodima, T.; Kaljurand, I.; Pihl, A.; Mäemets, V.; Leito, I.; Koppel, I. A. Acid−Base Equilibria in Nonpolar Media. 2.1 Self-Consistent Basicity Scale in THF Solution Ranging from 2-Methoxypyridine to EtP1(pyrr) Phosphazene. *J. Org. Chem.* **2002**, *67* (6), 1873-1881.
(15) Garrido, G.; Koort, E.; Ràfols, C.; Bosch, E.; Rodima, T.; Leito, I.; Rosés, M. Acid−Base Equilibria in Nonpolar Media. Absolute pK_a Scale of Bases in Tetrahydrofuran. *J. Org. Chem.* **2006**, *71* (24), 9062-9067.
(16) Wise, C. F.; Agarwal, R. G.; Mayer, J. M. Determining Proton-Coupled Standard Potentials and X-H Bond Dissociation Free Energies in Nonaqueous Solvents Using Open-Circuit Potential Measurements. *J. Am. Chem. Soc.* **2020**, *142* (24), 10681-10691.

Part B

Computational

Contents

I. Statistical Measures S56

II. Computational Methods S57

III. Method Assessment for Obtaining Molecular Geometries S63

IV. Tackling the Conformational Challenge S64

IV.I Initial Tests for [Pd−Br−Pd]$^+$ S64

IV.II Addressing Errors in the SQM PES of [HBrH•2 OEt_2]$^+$ S66

V. Method Assessment for Energy Refinements S67

VI. Modeling Isotopic Substitution in the Dynamic K_1 Equilibrium S69

VII. Determining the Lowest-Energy Conformer of [PdBrPd]$^+$ in CH_2Cl_2 Solution S72

VIII. Additional EDA Results S73

IX. More Details on the Potential Implications of Acidification on Catalysis S79

X. Additional Computational Data S83

XI. List of Abbreviations S84

XII. References S85

I. Statistical Measures

The error of a calculated quantitity $q_{\text{calc.}}$ to the experimental reference value $q_{\text{exp.}}$ is given by

$$\Delta q = q_{\text{calc.}} - q_{\text{exp.}} \,. \tag{18}$$

For a set of N errors $\{\Delta q_i\}$, common statistical measures are then defined by the following equations:

$$\text{Mean error: ME} = \frac{1}{N}\sum_{i=1}^{N} \Delta q_i \tag{19}$$

$$\text{Mean absolute error: MAE} = \frac{1}{N}\sum_{i=1}^{N} |\Delta q_i| \tag{20}$$

$$\text{Absolute maximal error: AMAX} = \max_i \left(\{|\Delta q_i|\}\right) \tag{21}$$

$$\text{Maximal error: MAX} = \max_i \left(\{\Delta q_i\}\right) \tag{22}$$

$$\text{Minimal error: MIN} = \min_i \left(\{\Delta q_i\}\right) \tag{23}$$

$$\text{Standard deviation: SD} = \sqrt{\frac{1}{N-1}\sum_{i=1}^{N} \left(\Delta q_i - \text{ME}\right)^2} \tag{24}$$

II. Computational Methods

In general, Gibbs energies of a conformer ensemble (CE) are calculated as

$$G = \overline{G} + G_{\text{conf}} \tag{25}$$

$$= \overline{E}_{\text{gas}} + \overline{\Delta G}_{\text{mRRHO}} + \overline{\Delta G}_{\text{solv}} + G_{\text{conf}}, \tag{26}$$

where all overlined values correspond to Boltzmann-averaged properties[1], which are expensive to compute at higher levels of theory. Hence, G is approximated herein as

$$G \approx E_{\text{gas}} + \Delta G_{\text{mRRHO}} + \Delta G_{\text{solv}} + \underbrace{\overline{G}_{\text{CENSO}} - G^{\text{min}}_{\text{CENSO}}}_{\Delta\overline{G} > 0}, \tag{27}$$

with the gas phase electronic energy E_{gas}, a thermostatistical correction of the former based on the mRRHO model[2] ΔG_{mRRHO}, and the free energy of solvation ΔG_{solv}. To reduce computational cost, all three terms are only evaluated for the lowest-energy conformer. However, the Boltzmann averaged Gibbs energies are accessible at a lower level of theory ($\overline{G}_{\text{CENSO}}$), which allows for an approximate inclusion of ensemble averaging by introducing a constant energy shift $\Delta\overline{G} > 0$ with respect to the minimum free energy at lower level of theory $G^{\text{min}}_{\text{CENSO}}$, here after the conformational reranking with CENSO[1] (see details below). Thus, only conformational free energy contributions are neglected ($G_{\text{conf}} = 0$), a justified simplification given their computational complexity and resource intensity.[3]

Generation of Docked Structures

To obtain reasonable starting structures of the non-covalently bound acid-base adducts for subsequent conformational sampling, we performed automatic (static) docking using the aISS module[4] in version 6.7.0 of the XTB program[5] at the GFN2-xTB[6]/ALPB[7] level of theory (CH_2Cl_2, $\varepsilon = 8.93$ [8]). To ensure tight convergence and to avoid potential grid complications, excessively fine settings were used for the docking (`--atm`, `--pocket`, `--opt extreme`, `--stepr 2`, `--stepa 20`, `--maxgen 15`, `--maxparent 200`, `--nstack 5000`). The docked structures of $[PdCH_2]^+$ with H_2NAr^F, H_2NAr^{Cl}, and OEt_2 were not consistent with chemical intuition, assuming a loosely bound conjugate base near the CH_2 group. Hence, an attractive potential (`scaling factor=1.0`) was added between the CH and the NH_2 group to also generate such structures.

The 15 structures lowest in energy after the final geometry optimization of each docking run were energetically reranked using part one of the CENSO routines[1], employing the efficient r^2-SCAN-3c[9] composite method with version 6.0.1 of the ORCA electronic structure program package.[10,11] Solvation effects were considered via the SMD[12] continuum model for CH_2Cl_2

and thermostatistical corrections from the single-point Hessian (SPH)[13] approach were calculated at the GFN2-xTB[6]/ALPB[7](CH_2Cl_2) level of theory. This (conformational) reranking will be referred to as CENSO reranking throughout and is the basis for determining $\Delta\overline{G}$ of a full CE for approximate ensemble averaging as used in equation (27).

The above docking and reranking procedure was iteratively applied to generate all bromonium-bridged adducts (e.g., $[HBrH{\bullet}2\,OEt_2]^+$), featuring a bromide interacting with two acid units.

Conformational Analysis

The conformational space of all investigated species was explored using the GOAT algorithm[14] (`maxitermult 6.0`, `gfnuphill gfnff`, `freezeamides true`, `autowall false`) with ORCA (refer to Section IV.I for a discussion of the chosen settings). Bond constraints were incorporated in the `%geom` block of the ORCA input for select species to preserve specific internal coordinates during conformational sampling, such as critical bonds in transition state structures or the dihedral angles for molecules presented in Figure 3c of the main manuscript. Thus, if not stated otherwise, transition state structures were initially entirely optimized without addressing the conformational flexibility, followed by a comprehensive conformational analysis with essential bond constraints before reoptimizing to the final transition state structures.

All non-covalent interactions (NCI)-adducts were sampled similarly using GOAT both with and without an automatic outer wall potential (`AUTOWALL`), but also via the NCI mode with the CREST program[15–17] (version 1.2.0), again with adjusted settings (`--keepdir`, `--nci`, `--mdtemp 200`, `--wscal 0.6`, `--nmtd 6`, `--mdlen x1.5`, `--mddump 600`, `--nocross`, `--opt extreme`, `-T 24`; see Section IV.). Please note the scaled wall potential and reduced meta-dynamics (MTD) temperature, which were determined by visually checking a selection of MTD trajectories from initial test runs. A tighter wall potential is indeed crucial for any meaningful NCI sampling given that the default value only results in dissociating molecular fragments due to the applied RMSD-based bias potential. Reducing the MTD temperature also allows for more subtle NCIs to be captured as faster molecular movement at higher temperatures usually leads to immediate disruption of such low-energy NCIs.

Finally, the lowest-energy structure after full CENSO reranking of *all* obtained conformers combined from *all* conformational sampling runs was used in the subsequent geometry optimizations as detailed in the following Section. Hence, while not every specific choice of settings was rerun multiple times, a minimum combined total number of four GOAT and CREST runs effectively guarantees sufficient sampling of the NCIs (refer to Section IV. for details).

Further note that HBr dissociation was observed during the MTD runs for some of the Pd adducts with an acid, which required the introduction of bond constraints and the usage of

smaller MTD time steps (`--cinp name.inp`, `--subrmsd`, `--tstep 2.5`). This was also done for the constraints used to resolve errors in the semiempirical quantum mechanical (SQM) potential energy surface (PES) for $[HBrH{\bullet}2\,OEt_2]^+$ as described in Section IV.II.

The level of theory for the conformational benchmarking was consistently set to GFN2-xTB[6]/-ALPB[7](CH_2Cl_2) for both GOAT and CREST. However, the solvent was adjusted for the Pd(APAQ) (HFIP, $\varepsilon = 16.7$ [18,19]) and the M(OAc)(LX) (MeCN, $\varepsilon = 36.64$ [8]) systems. Since HFIP has not been parametrized for ALPB yet, benzaldehyde ($\varepsilon = 17.85$ [8]) was used instead. Hence, all CEs obtained this way were energetically reranked with CENSO as described above using the appropriate solvent for the system at hand. Additionally, test calculations also employed the GFN-FF[20] method (see Section IV.I).

Final Geometries

If not stated otherwise, all high-level DFT calculations in the following were carried out in an unrestricted fashion. The geometries obtained after conformational sampling are only of low quality and need to be reoptimized, which was done with ORCA using the efficient r^{2}SCAN-3c[9] composite method and CPCM[21,22] continuum solvation (`TIGHTOPT`, see Section III. for method justifications). Subsequent vibrational frequency analysis confirmed the nature of the stationary points on the PES (no imaginary modes: stable intermediate, exactly one imaginary mode: transition state structure) and allowed the calculation of $\Delta G_{\mathrm{mRRHO}}$. To account for isotopic substitution of hydrogen atoms with deuterium, the computed Hessian matrix was used to reevaluate the $\Delta G_{\mathrm{mRRHO}}$ contributions for every substitution pattern as detailed in Section VI.

Due to the presence of dihedral angle constraints, conventional thermostatistical corrections could not be applied to the bimetallic Pd species shown in Figure 3c, as these non-equilibrium structures violate the harmonic vibrational approximation. Instead, $\Delta G_{\mathrm{mRRHO}}$ values were obtained from SPH[13]-GFN2-xTB/ALPB calculations (`--opt extreme`), providing a more appropriate treatment of such non-stationary points on the PES.

All geometries were reoptimized in the given solvent, consequently leading to slightly altered vibrational frequency contributions for each investigated solvent. Addtionally considered solvent systems were THF ($\varepsilon = 7.52$ [8]) and DCE ($\varepsilon = 10.42$ [8]). The complete conformational workflow was not reinitiated due to the high computational cost (e.g., as for $[Pd{-}Br{-}Pd]^+$). Further, HFIP CPCM solvation was accommodated in ORCA by specifying `epsilon 16.7` in the `%cpcm` block.[18,19]

Final Electronic Energy Refinements

All electronic energies were further refined in the gas phase at the PBE0-D4[23–25]/def2-QZVPP[26,27] (def2-ECP[28] for Pd) level of theory, using tight SCF settings (`TIGHTSCF`) and large grids for numerical integration (`DEFGRID3`). The RIJCOSX[29,30] approximation was utilized with the large def2/JK[31] auxiliary basis set. For functional benchmarking, parts of the equilibrium and thermochemical data were further evaluated using the functionals shown in Table S15 with the def2-QZVPP basis set. To account for the need for diffuse functions in negatively charged species, the minimally augmented ma-def2-QZVPP[32] basis set was used for the pK_a computations from Figure 9c.

Table S15. Functionals with corresponding references used in the functional benchmark.

Functional	References
TPSSh-D4	[24,25,33]
B3LYP-D4	[24,25,34,35]
PBE0-D4	[23–25]
r^2SCAN0-D4	[24,25,36]
PW6B95-D4	[24,25,37]
ωr^2SCAN-D4	[24,25,38]
ωB97X-V	[39,40]

Solvation Free Energies

SMD and CPCM solvation energies were computed as the energy difference of a gas- and solution-phase single point calculation with the r^2SCAN-3c method on the solution phase geometries, using both `TIGHTSCF` and `DEFGRID3` in ORCA. COSMO-RS[41–43], and COSMO-RS(fine)[41–43] solvation energies were obtained with the COSMOtherm program (version C3.0, release 16.01, 2016 parameterization: BP_TZVP_C30_1601.ctd and BP_TZVPD_FINE_C30_1601.ctd, default Gsolv option). All solvation free energies are properly corrected for the change in standard state to 1 mol L^{-1}.[44]

Further Computational Details

Identical activity and diffusion coefficients for all pairs of oxidized and reduced species were assumed to approximate the experimentally measured half-potentials with herein computed redox potentials.[45] Using the direct approach[46,47], all potentials are reported against the Fc/Fc^+ redox couple *calculated* in eclipsed conformation[48] with THF as the solvent.

The free energy of $H^+_{(solv)}$ cannot be calculated by quantum chemical methods[49] and is thus prone to errors, which is why an reaction scheme[50] was constructed with 2,4,6-trimethylpyridinium ($pK_a{}^{THF}$ = 8.1 [51]) as an experimental reference point for computing the pK_a values in THF solvent. Similarly, BDFEs can be calculated using this approach[52], which was anchored to the experimental BDFE of TEMPOH (BDFE(THF) = 65.5 kcal mol^{-1} [53]). All reaction schemes used for these calculations are shown in Figure S65. However, please note that all of the above quantities reported versus the calculated Ni values do not require any specific reference choice (e.g., Figure S76).

Fc (solv)	⇌	Fc^+ (solv) + e^- (solv)	$-\Delta G_{ref} = F \cdot E^o_{abs}(Fc/Fc^+)$
**R** (solv) + e^- (solv)	⇌	$\mathbf{R}^-$ (solv)	$\Delta G_R = -F \cdot E^o_{abs}(\mathbf{R}^-/\mathbf{R})$
**R** (solv) + Fc (solv)	⇌	$\mathbf{R}^-$ (solv) + Fc^+ (solv)	$\Delta G_{DFT} = -F \cdot E^o_{vs.\ Fc/Fc+}(\mathbf{R}^-/\mathbf{R}) = -F \cdot (E^o_{abs}(\mathbf{R}^-/\mathbf{R}) - E^o_{abs}(Fc/Fc^+))$

Ref-H (solv)	⇌	Ref^- (solv) + H^+ (solv)	$\Delta G_{ref} = \ln(10) \cdot RT \cdot pK_a$ (Ref-H)
**R-H** (solv) + Ref^- (solv)	⇌	$\mathbf{R}^-$ (solv) + Ref-H (solv)	ΔG_{DFT}
**R-H** (solv)	⇌	$\mathbf{R}^-$ (solv) + H^+ (solv)	$\ln(10) \cdot RT \cdot pK_a$ (**R-H**) = $\Delta G_{ref} + \Delta G_{DFT}$

Ref'-H (solv)	⇌	Ref'• (solv) + H• (solv)	BDFE(Ref'-H)
**R-H** (solv) + Ref'• (solv)	⇌	**R**• (solv) + Ref'-H (solv)	ΔG_{DFT}
**R-H** (solv)	⇌	**R**• (solv) + H• (solv)	BDFE(**R-H**) = BDFE(Ref'-H) + ΔG_{DFT}

Figure S65. Reaction schemes used for obtaining computational redox potentials (top), pK_as (middle), and BDFEs (bottom), where ΔG_{DFT} corresponds to the computed reaction energies, which each do not depend on the Gibbs energy of the solvated electron, proton, nor hydrogen atom. Ref—H stands for the 2,4,6,-trimethylpyridinium ion, and Ref′—H corresponds to TEMPOH.

ChimeraX[54,55] (version 1.9) was used for visualization purposes.

Energy Decomposition Analysis

Geometries as obtained from optimizing with r^2SCAN-3c/CPCM(DCM) in ORCA were used as a starting point for our EDA investigation. To allow for separation of interactions of the adamantane and phophane units of the pincer ligand with the metal, both CH_2 bridges were removed and open coordination sites re-saturated with hydrogen atoms. To allow for a fair comparison to the full system, featuring the CH_2 bridge, only the hydrogen atoms were re-optimized in the sliced structures, leaving all heavy atoms at exactly identical positions compared to the full structures (see Figure S69). These geometries were then used for subsequent second-generation vertical ALMO-EDA[56–58] calculations in a restricted fashion using

Q-Chem 6.3.0.[59] The quasiclassical decomposition[60] of the frozen energy was employed and the full ALMO basis was used for constructing the polarized state.[58] Further, VFB-CT analysis provided access to unidirectional CT contributions[61] and variational (non-perturbative) COVPs[62,63] were generated for more in-depth CT characterization. If not stated otherwise, all COVPs were plotted using ChimeraX[54,55] with an isovalue of 0.065 Å^{-3}.

The B3LYP[34,35]-D3(BJ)[64–66] functional with the def2-TZVPP[27] basis set (def2-ECP[28] for Pd) was used for the energy evaluations, given its general robustness in correctly describing the intrinsics of various chemical bonds in previous EDA studies.[61,67–69] Implicit solvation[70] was consistently included for all intermediate EDA states via the conductor-like polarizable continuum model[21,22](DCM, ε=8.93 [8]), employing a high-quality Lebedev grid of 302 points per atom and universal force field[71] atomic van der Waals radii for cavity construction.

To nevertheless test the transferability of results to other levels of theories, the EDA components were re-evaluated at PBE0-D3(BJ)[64–66]/mixed (def2-TZVP for Ni and Pd, def2-ECP[28] for Pd, and de2-SVP for remaining atoms)[27] level of theory with the SMD[12] solvation model. SMD introduces a small non-electrostatic solvation contribution, which is added to $\Delta E_{\mathrm{PAULI}}$, whereas the purely electrostatic solvation contribution is combined with ΔE_{ELEC} as usual.[70]

Please note that in current versions of Q-Chem, the charge scaling of D4[24,25] is broken, which is why the D3(BJ)[64–66] dispersion correction was employed instead. All calculations in Q-Chem utilized the SG-2 standard grid[72] for numerical integration.

III. Method Assessment for Obtaining Molecular Geometries

Table S16. Comparison of key bond lengths of $[PdCH_2]^+$ (in Å) for different methods versus the experimental crystal structure (XRD). PBE0-D4, and TPSSh-D4 were employed with the def2-TZVP basis set, and CPCM(CH_2Cl_2) was used throughout. The RMSD and its heavy variant without consideration of hydrogen atoms (hRMSD) are given w.r.t the XRD structure (in Å). The XRD was used as an initial guess structure to ensure comparability.

Bond	XRD	r^2SCAN-3c	ωB97X-3c[73]	PBE0-D4	TPSSh-D4
Pd1–Br1	2.41	2.45	2.44	2.42	2.43
Pd1–P1	2.33	2.35	2.35	2.33	2.33
Pd1–P2	2.32	2.36	2.35	2.33	2.34
Pd1–C47	2.30	2.31	2.29	2.27	2.28
Pd1–H47A	2.25	2.29	2.23	2.21	2.21
Pd1–H47B	1.67	1.71	1.73	1.71	1.72
C47–H47A	0.87	1.10	1.10	1.10	1.10
C47–H47B	0.87	1.18	1.15	1.17	1.17
RMSD	-	0.50	0.53	0.57	0.57
hRMSD	-	0.33	0.37	0.40	0.40

Table S17. Comparison of key bond lengths (in Å) for the symmetric transition state structures of $[PdCH_2]^+$ optimized with different computational methods. PBE0-D4, and TPSSh-D4 were employed with the def2-TZVP basis set, and CPCM(CH_2Cl_2) was used throughout.

Bond	r^2SCAN-3c	ωB97X-3c[73]	PBE0-D4	TPSSh-D4
Pd1–Br1	2.44	2.44	2.42	2.43
Pd1–P1	2.34	2.34	2.32	2.33
Pd1–P2	2.34	2.34	2.32	2.33
Pd1–C47	2.27	2.26	2.24	2.25
Pd1–H47A	1.94	1.93	1.92	1.93
Pd1–H47B	1.95	1.95	1.92	1.92

Tables S16 and S17 contain key bond lengths of the asymmetric $[PdCH_2]^+$ and its symmetric transition state as calculated with different methods. As expected, the XRD reference structure is sufficiently reproduced by all methods, and the deviations to the experimental structure are consistent between the composite methods and the employed hybrid density functional approximations (DFAs). A similar picture emerges for the transition state structures, for which only minimal deviations between methods can be observed, suggesting that r^2SCAN-3c is suitable for computing both equilibrium geometries as well as transition state geometries for subsequent barrier height evaluations within this study (e.g., despite lacking exact exchange[74]).

Systematic discrepancies w.r.t. the experimental crystal structure, such as those in the Pd1–H47A/B bond lengths, likely stem from solid-state packing effects that cannot be captured by single-molecule solution-phase calculations, regardless of the chosen DFT method.

IV. Tackling the Conformational Challenge

IV.I Initial Tests for [Pd−Br−Pd]$^+$

Exploring the conformational space of flexible molecules becomes increasingly challenging as the molecular size increases. To address this challenge, we evaluated two state-of-the-art approaches for conformational sampling (GOAT[14] and CREST[16]) with varying settings and SQM methods for the conformationally most challenging system in our study, the bimetallic Pd complex ([PdBrPd]$^+$). Table S18 provides the relative Gibbs energies of the lowest-energy conformer after full CENSO reranking as obtained from a specific run, where the following changes to default settings may apply:

CREST:

- GFN-FF: MTD simulations were carried out with the efficient GFN-FF force field with ALPB solvation, and the geometries of the resulting CE were subsequently relaxed at GFN2-xTB/ALPB level of theory.
- LONGMTD: The MTD simulation length was increased to 250 ps or 500 ps for GFN2-xTB or GFN-FF runs, respectively. For reference, CREST otherwise uses a value of approximately 100 ps for this specific system.

GOAT:

- GFNUPHILL: Use GFN-FF for the uphill steps, reducing the computational cost of each GOAT iteration.
- MAXITERMULT: Change the multiplication factor for the number of geometry optimizations per worker from the default value of 3 to 6, which increases the length of each GOAT iteration, similar to increasing the MTD time length, albeit at a different scale.
- OPTTIGHT: Also set the optimization threshold for GOAT runs to `OPTTIGHT` in ORCA.

Table S18. Relative Gibbs energies of each lowest-energy conformer as obtained from different CREST and GOAT runs after full CENSO reranking.

Run Mode	G_{rel} (kcal/mol)
GOAT + GFNUPHILL + MAXITERMULT	0.000
GOAT + OPTTIGHT	0.236
GOAT + GFNUPHILL	0.248
GOAT (default)	0.249
CREST + GFN2-xTB + LONGMTD	0.185
CREST + GFN2-xTB (default)	0.645
CREST + GFN2-xTB (default, second run)	0.845
CREST + GFN-FF (default)	3.987
CREST + GFN-FF + LONGMTD	5.059

Please note that for an appropriate comparison of CREST with GOAT, a minimum of at least three or ideally even more runs would be needed for each specific change in settings due to the non-deterministic nature of both approaches, prohibiting a direct comparison of individual runs.

Nevertheless, the energetic ranking in Table S18 is still insightful, indicating GOAT to be more robust in finding a minimum energy structure for this molecular example. Both approaches are sensitive to the effective simulation time lengths (`LONGMTD` and `MAXITERMULT`), where larger values are beneficial. Hence, after visual inspection of multiple MTD runs in the NCI-CREST mode as used for the acid-base complexes (see Section II.), the MTD time lengths were scaled to 150 %.

Additionally, as is somewhat expected, relaxing the geometries of a GFN-FF-CE with GFN2-xTB does not necessarily redeem the quality of a full conformational exploration with GFN2-xTB. Finally, using the `GFNUPHILL` keyword approximately reduces the overall computation time by a factor of two, while seemingly retaining the robustness of a regular GOAT run.

Based on these results, it appears most promising to employ the GOAT algorithm in combination with both `GFNUPHILL` and `MAXITERMULT` for conformational analysis, where `GFNUPHILL` nicely compensates the greater computational cost of increasing `MAXITERMULT`.

IV.II Addressing Errors in the Semiempirical Potential Energy Surface of $[HBrH\bullet 2\,OEt_2]^+$

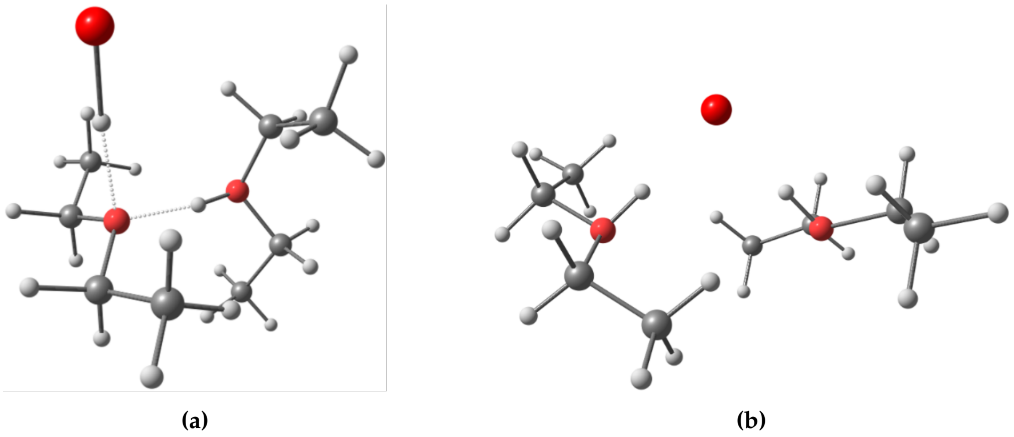

Figure S66. Lowest structure for $[HBrH\bullet 2\,OEt_2]^+$ after CENSO reranking as obtained from the best GOAT run (GFN2-xTB geometry) (a), and the overall lowest structure (r^2SCAN-3c) found as described in the text below (b).

Initially, the different NCI runs (CREST, GOAT with/without `AUTOWALL`, etc.) were compared after full CENSO reranking using the GFN2-xTB optimized geometries. However, preliminary tests with r^2SCAN-3c revealed that there is a significant error in the PES of GFN2-xTB for $[HBrH\bullet 2\,OEt_2]^+$ as exemplified by the two structures in Figure S66. The structure resulting from the best GOAT run (Figure S66a) is an adduct of HBr with the cation of Brookhart's acid instead of the bromonium bridged species as proposed in the main text (Figure S66b). At GFN2-xTB/ALPB, the latter is 5.1 $\mathrm{kcal\,mol^{-1}}$ higher in energy, whereas the qualitative ranking is completely reversed at r^2SCAN-3c/SMD level of theory, at which the former is 9.8 $\mathrm{kcal\,mol^{-1}}$ higher in energy. Hence, one can expect significant issues with the conformational sampling given that all intermediate structures are necessarily ranked at the GFN2-xTB/ALPB level of theory, motivating us to conduct a more thorough analysis to obtain the actual minimum structure of $[HBrH\bullet 2\,OEt_2]^+$.

For this, we performed multiple CREST runs, symmetrically constraining both H−Br hydrogen bonds systematically from 1.30 Å to 2.5 Å with a step size of 0.05 Å. All resulting CEs were subsequently reranked with CENSO, the lowest-energy structure optimized with r^2SCAN-3c/CPCM and finally reranked at PBE0-D4/def2-QZVPP/SMD + $\Delta G_{\mathrm{mRRHO}}$(GFN2-xTBALPB-SPH) level of theory. Hence, we effectively utilized a multitude of reasonable initial guess geometries for the r^2SCAN-3c optimizations, finally resulting in a geometry that is mostly free of the GFN2-xTB error and indeed the lowest-energy structure obtained for $[HBrH\bullet 2\,OEt_2]^+$ within this study.

Relevant in this context, g-xTB[75] is a novel SQM method currently developed by the Grimme

group that substantially enhances the accuracy of GFN2-xTB with only marginal additional computational cost. Notably, a preliminary version of g-xTB accurately predicts the energy ordering of the $[HBrH\bullet 2\,OEt_2]^+$ structures, showing remarkable concordance with the higher-level DFT methods employed herein. Consequently, once analytical nuclear gradients become available, the parallelity of the g-xTB PES relative to more sophisticated DFT methods can be leveraged in future conformational exploration, thus avoiding errors such as those described in this Section.

V. Assessing the Performance of Various Density Functional Approximations and Implicit Solvation Models

Table S19. Statistical measures for the five best-performing combinations of method and solvation model on the equilibrium data studied herein, with all energies given in units of $kcal\,mol^{-1}$.

Solvation Model	Method	MAE	ME	RMSE	MAX	MIN	AMAX	SD	N
COSMO-RS(fine)	PBE0-D4	2.18	-1.85	2.57	0.66	-3.77	3.77	2.06	4
COSMO-RS(fine)	r^2SCAN0-D4	2.23	-1.29	2.55	1.88	-3.58	3.58	2.54	4
COSMO-RS(fine)	ωr^2SCAN-D4	2.24	-2.24	2.62	-0.78	-3.84	3.84	1.58	4
COSMO-RS(fine)	TPSSh-D4	2.43	-2.43	2.73	-0.81	-3.87	3.87	1.43	4
SMD	ωr^2SCAN-D4	2.65	-2.65	3.17	-0.20	-4.86	4.86	2.01	4

Table S20. Comparison of the five best-performing method and solvation model combinations for the equilibrium data here listed with the predicted redox potentials for $[PdCH_2]^+$, with all values given in units of V. ΔE_0 corresponds to the deviation from the experimental value.

Solvation Model	Method	E_0^{calc}(Ni)	E_0^{calc}(Pd)	ΔE_0(Ni)	ΔE_0(Pd)[a]
COSMO-RS(fine)	PBE0-D4	0.342	0.848	0.187	0.038
COSMO-RS(fine)	r^2SCAN0-D4	0.300	1.057	0.145	0.247
COSMO-RS(fine)	ωr^2SCAN-D4	0.412	0.817	0.257	0.007
COSMO-RS(fine)	TPSSh-D4	0.265	0.616	0.110	-0.194
SMD	ωr^2SCAN-D4	0.137	0.591	-0.018	-0.219

[a] Irreversible oxidation; difference calculated to anodic peak potential.

Table S21. Comparison of the five best-performing method and solvation model combinations for the equilibrium data here listed with the predicted BDFEs for $[PdCH_2]^+$, with all values given in units of kcal mol^{-1}. ΔBDFE corresponds to the deviation from the experimental value.

Solvation Model	Method	BDFE^{calc}(Ni)	BDFE^{calc}(Pd)	ΔBDFE(Ni)	ΔBDFE(Pd)
COSMO-RS(fine)	PBE0-D4	67.9	74.4	-1.9	-7.6
COSMO-RS(fine)	r^{2}SCAN0-D4	64.0	72.9	-5.8	-6.1
COSMO-RS(fine)	ωr^{2}SCAN-D4	72.5	74.1	2.7	-4.9
COSMO-RS(fine)	TPSSh-D4	68.5	69.3	-1.3	-9.7
SMD	ωr^{2}SCAN-D4	76.5	78.4	6.7	-0.6

Table S22. Comparison of the five best-performing method and solvation model combinations for the equilibrium data here listed with the predicted pKa for $[PdCH_2]^+$. ΔpKa corresponds to the deviation from the experimental value and ΔΔpKa(Ni-Pd) is the computed acidification upon substituting Ni with Pd.

Solvation Model	Method	pKa^{calc}(Ni)	pKa^{calc}(Pd)	ΔpKa(Ni)	ΔpKa(Pd)	ΔΔpKa(Ni-Pd)
COSMO-RS(fine)	PBE0-D4	-4.2	-10.1	-8.4	-9.8	6.0
COSMO-RS(fine)	r^{2}SCAN0-D4	-3.4	-9.8	-7.6	-9.5	6.3
COSMO-RS(fine)	ωr^{2}SCAN-D4	-5.0	-10.6	-9.2	-10.3	5.7
COSMO-RS(fine)	TPSSh-D4	-4.4	-9.7	-8.6	-9.4	5.4
SMD	ωr^{2}SCAN-D4	2.5	-3.9	-1.7	-3.6	6.3

Table S19 provides statistical measures on the performance of the five best combinations of implicit solvation model and DFA, with PBE0-D4/COSMO-RS(fine) to yield the lowest MAE on the assessed experimental equilibrium data in DCM and DCE ($N = 3 + 1$; DCM: K_1, K_2, K_3; DCE: K_3). PBE0 is a generally robust hybrid DFA with only one empirical parameter, known for excellent performance also on electronically complicated metal species.[76,77] However, Tables S21 and S22 clearly show that the prediction of accurate absolute BDFE and pK_a data for $[PdCH_2]^+$ is challenging, regardless of the employed method combinations. It is well known that pKa values computed with COSMO-RS(fine) solvation need to be corrected via linear free energy relationships due to systematic deviations compared to experiment.[78] However, we note that the deviations reported herein are surprisingly large and not meaningful in terms of absolute numbers. Additionally, both the pK_a and BDFEs are intrinsically reference-dependent due to the use of the reaction schemes from Figure S65, which may be another issue at play. To circumvent both these potential systematic shifts in the computed thermochemical data, we opted to only report the relative change of those values going from nickel to palladium. Hence, we maximize favorable computational error cancellation while effectively employing an ideal reference compound each, given that the nickel and palladium complexes are nearly structurally identical. For instance, with this approach, the key shift in predicted acidification upon going from the nickel to the palladium species as one of the most crucial results of this

study is consistently reproduced for all employed levels of theory (within ± 1 pKa unit error), further supporting the roughly 100000-fold increase in C-H bond acidity (~ 5 pKa units) as observed experimentally. Please note that the data for all remaining method combinations not shown above can be found as `.csv` files in the appended `.zip` folder.

VI. Modeling Isotopic Substitution in the Dynamic K_1 Equilibrium

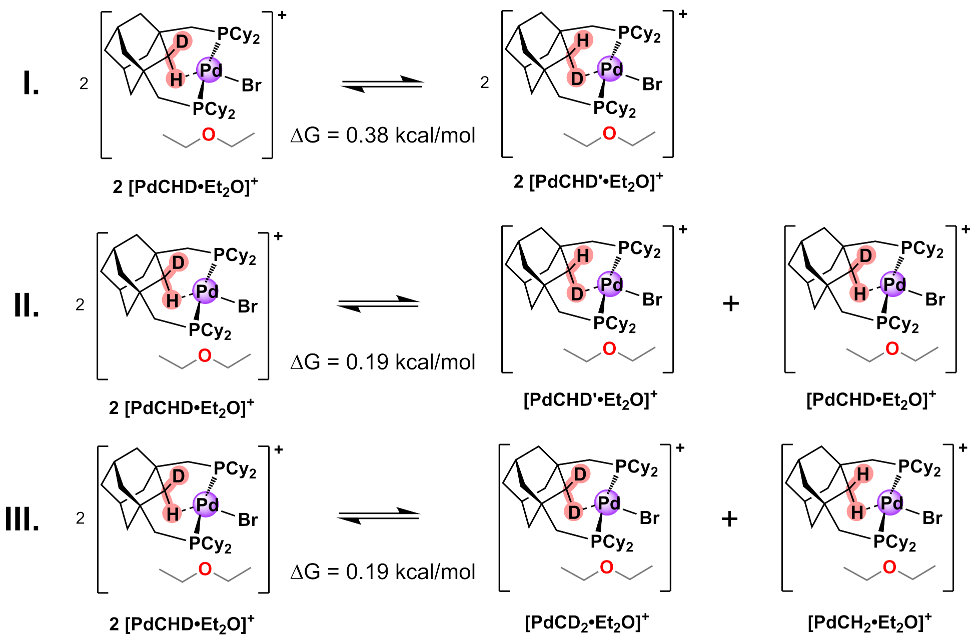

Figure S67. Gibbs free energitcs for the isotopic substitution of two equivalents of $[PdCH_2{\bullet}Et_2O]^+$.

Figure 4a of the main text shows all six protons of the K_1 equilibrium that undergo slow exchange in solution. Isotopic substitution of two of these protons, based on the stoichiometry of the reaction with deuterated acid, generates numerous possible substitution patterns and thus structure combinations, all contributing to the experimentally observed equilibrium isotope effect (EIE). To investigate this computationally, we first determined the distribution of two deuterium on the reagent side of the equilibrium (see Figure S67), revealing that substitution of H47A with longer distance to palladium (and thus higher stretching frequency) is energetically favored, which has been reported for an agostic osmium complex before.[79]

Assuming the lowest-energy structure with both deuterium residing in the H47A position to be most populated, the isotopic shift of the K_1 equilibrium can be approximated by thermostatistically averaging over all six possible reactions starting from two equivalents of $[PdCHD{\bullet}Et_2O]^+$ as shown in Figure S68. This analysis finally yields an equilibrium free energy

of 1.08 kcal mol^{-1} for the deuterated K_1 equilibrium in CH_2Cl_2, including an isotopic shift of 0.22 kcal mol^{-1}, which corresponds to a computational EIE of 1.5. Both the isotopic shift and EIE values remain consistent when calculated at 298 K and 283 K, with precision to two and one significant decimal(s), respectively.

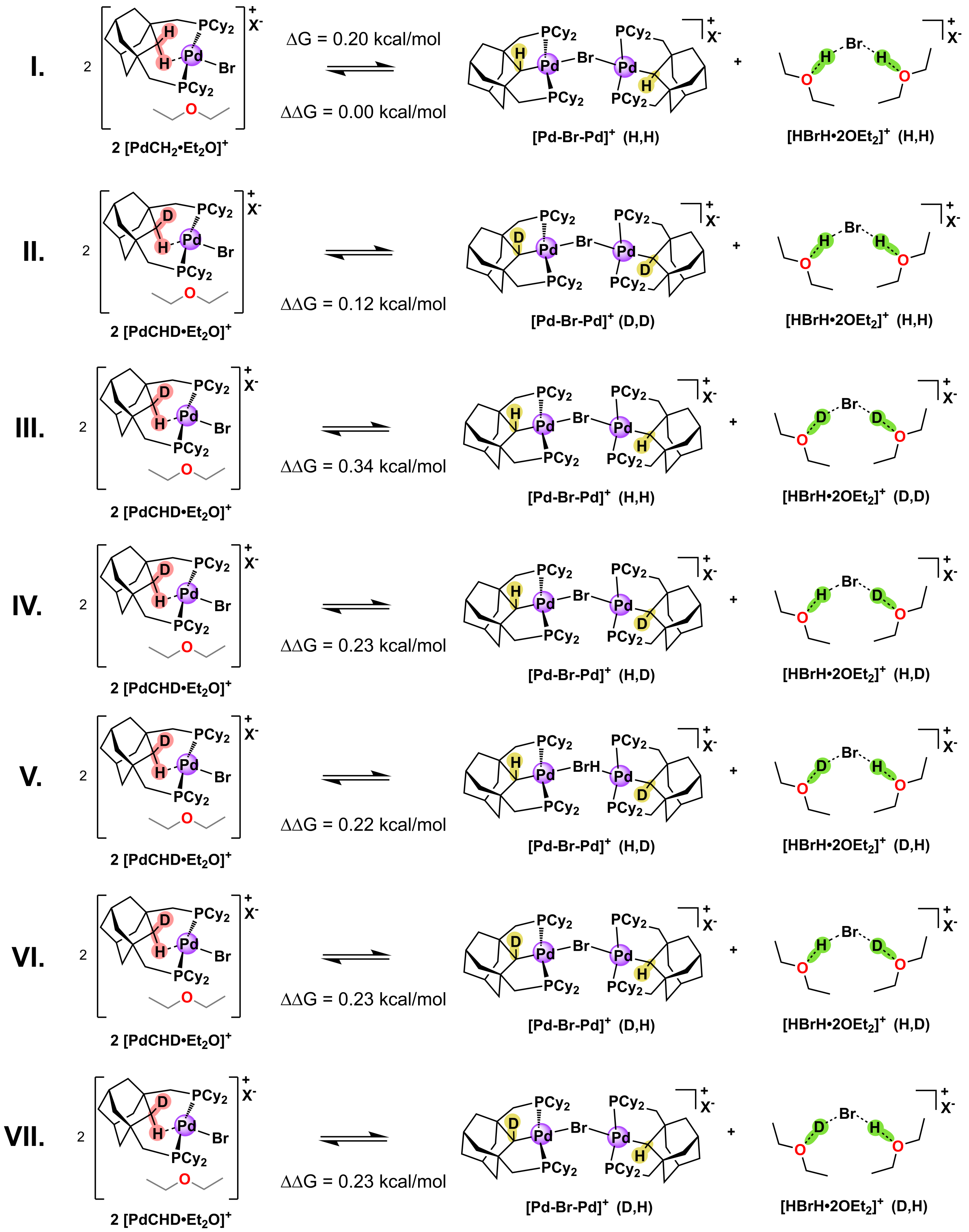

Figure S68. Shift in Gibbs free energy ($\Delta\Delta G$) upon substituting two of the exchanging protons (marked in red, green and yellow, respectively) with deuterium for all six combinations relative to the undeuterated equilibrium with experimental reaction energy of $\Delta G = 0.20\,\mathrm{kcal\,mol^{-1}}$.

VII. Determining the Lowest-Energy Conformer of $[PdBrPd]^+$ in CH_2Cl_2 Solution

Table S23. Relative free energies in kcal mol^{-1} for the lowest-energy conformer obtained after application of the full multi-level workflow in DCM as described in Section II. (blue structure from Figure 2c in the main text, CONF) compared to the DFT-optimized experimental solid-state structure (brown structure from Figure 2c, XRD). Conformational energies are provided for various solvation models and as pure gas-phase energies at PBE0-D4/def2-QZVPP, while consistently including proper thermal corrections ($\Delta G_{\mathrm{mRRHO}}$) and setting $\Delta \overline{G}$ to zero for a fair comparison.

Solvation Model	XRD	CONF
Gas	0.0	0.1
CPCM	0.0	0.2
SMD	0.9	0.0
COSMO-RS	0.0	0.4
COSMO-RS(fine)	0.0	1.7

The conformational energies shown in Table S23 reveal the complexity of accurately modeling solvation effects on conformational preferences. While CPCM and COSMO-RS yield nearly isoenergetic conformers (similar to the gas-phase calculations), only SMD and COSMO-RS(fine) predict substantial energy differences, favoring CONF and XRD, respectively.

Notably, according to our best-performing method combination from Section V., conformational sampling would have actually generated a structure higher in energy than the one initially used in the workflow. This apparent contradiction highlights critical limitations of our current computational approach. First, our benchmarking was necessarily restricted to a very small set of equilibrium data points, precluding broad claims about method accuracy and robustness across diverse chemical environments (e.g., highly accurate conformational energies in this case). Second, the computed energy differences (typically <2 kcal/mol) fall well within our expected DFT error margins, making definitive energetic rankings less reliable.

Given these limitations, we adopt a pragmatic approach that prioritizes chemical robustness over strict energy minimization. We retain conformationally explored structures unless the computed energy difference exceeds 2-3 kcal/mol at our chosen level of theory (high level DFT), or when chemical intuition suggests significant differences in binding behavior between structural candidates. This reflects the reality that our computational accuracy limits cannot reliably distinguish smaller energy differences, while the consequences of neglecting conformational flexibility entirely can be far more severe than using a slightly higher-energy, but still thermally populated conformer (see Figure 9b of the main text for one "conformational" example encountered in this study). Thus, we, for example, maintain the conformationally sampled

geometry of $[PdBrPd]^+$, recognizing that the computational error window likely exceeds the small relative energy differences observed in Table S23, which is why we do not emphasize on such small energetic differences within the main text of this study.

VIII. Additional EDA Results

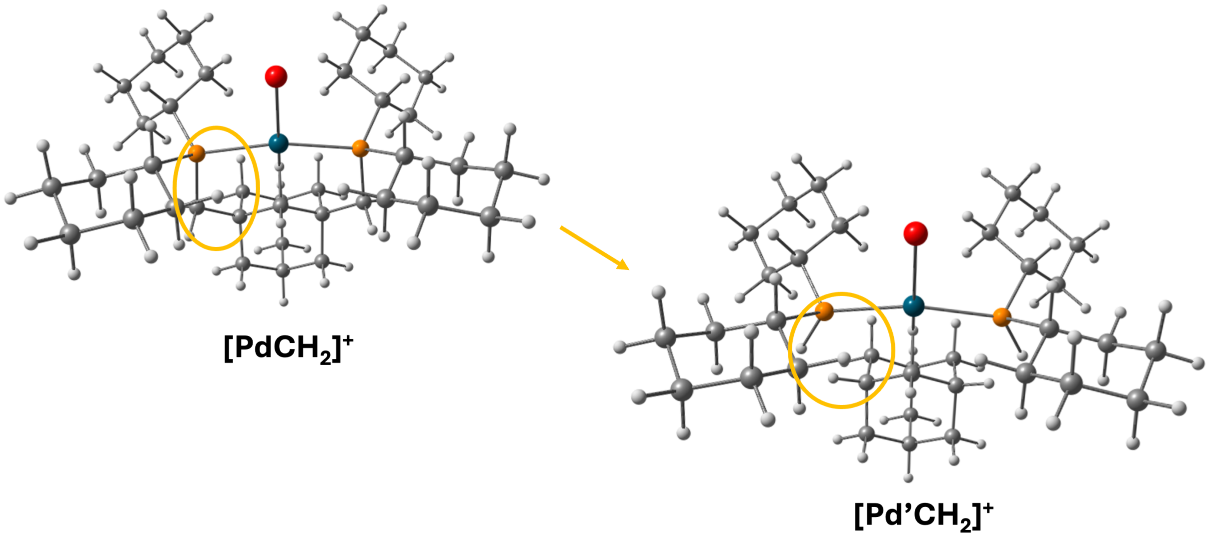

Figure S69. Exemplary slicing of the full $[PdCH_2]^+$ system into four individual fragments of the $[Pd'CH_2]^+$, allowing for separation of interactions between different parts of the pincer ligand with the metal center. Only the hydrogen atoms have been reoptimized in $[Pd'CH_2]^+$.

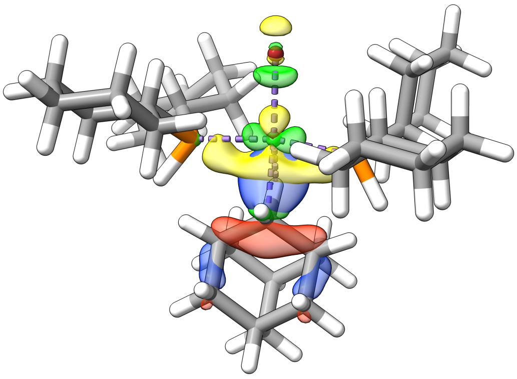

(a) $[Ni'CH_2]^+$: 64.9 % CTf, 34.7 % CT

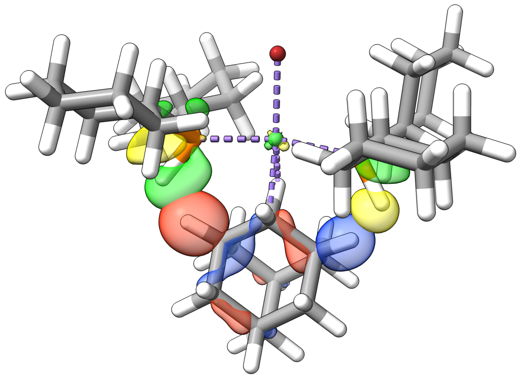

(b) $[Ni'CH_2]^+$: 12.8 % CTf, 6.9 % CT

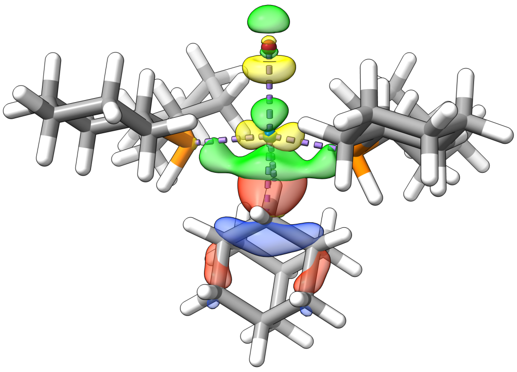

(c) $[Pd'CH_2]^+$: 83.6 % CTf, 54.8 % CT

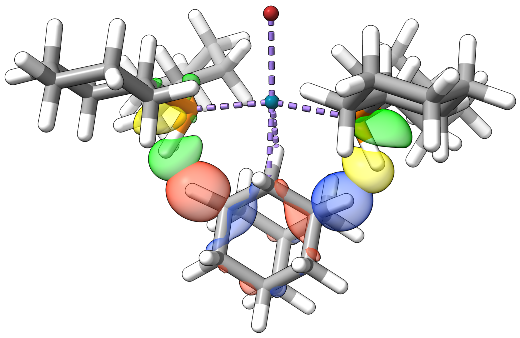

(d) $[Pd'CH_2]^+$: 7.4 % CTf, 4.9 % CT

Figure S70. Two most significantly contributing COVPs for forward CT (CTf) in the sliced $[Ni'CH_2]^+$ (a+b) and $[Pd'CH_2]^+$ (c+d) complexes where adamantane and all remaining parts of the complex (including both $HPCy_2$) were chosen as the two fragments in VFB-ALMO-EDA calculations at the B3LYP-D3(BJ)/def2-TZVPP/CPCM(DCM) level of theory. The donating orbital is shown in red and blue color, whereas the accepting orbital is displayed in yellow and green, both with an isovalue of 0.065 $Å^{-3}$. The significance of the given orbital pair with respect to the unidirectional and total CT energies is reflected in the percent values given in each subfigure.

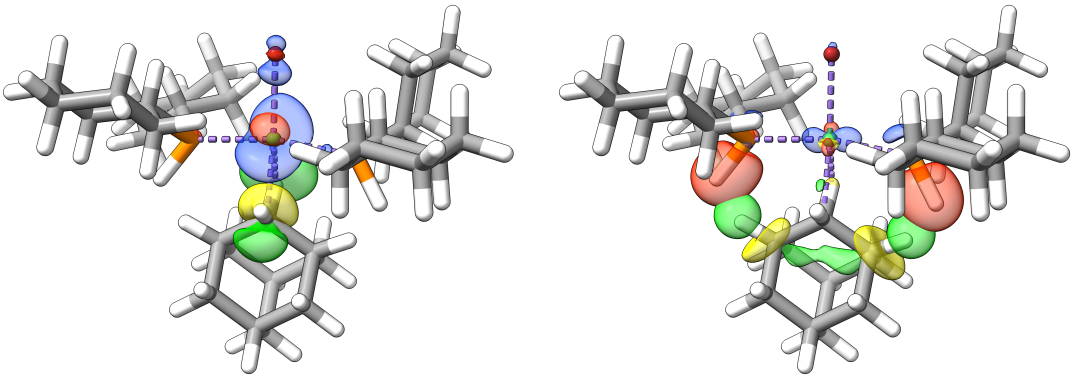

(a) $[Ni'CH_2]^+$: 23.9 % CTb, 10.5 % CT

(b) $[Ni'CH_2]^+$: 18.6 % CTb, 8.2 % CT

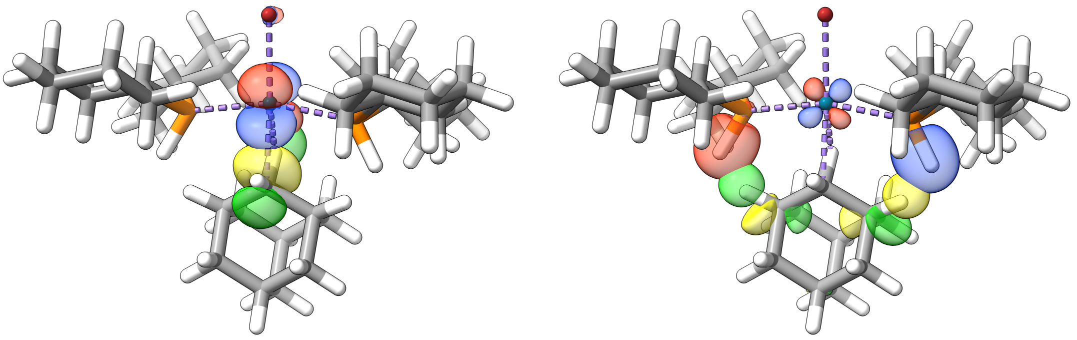

(c) $[Pd'CH_2]^+$: 41.6 % CTb, 13.1 % CT

(d) $[Pd'CH_2]^+$: 17.0 % CTb, 5.3 % CT

Figure S71. Two most significantly contributing COVPs for backward CT (CTb) in the sliced $[Ni'CH_2]^+$ (a+b) and $[Pd'CH_2]^+$ (c+d) complexes where adamantane and all remaining parts of the complex (including both $HPCy_2$) were chosen as the two fragments in VFB-ALMO-EDA calculations at the B3LYP-D3(BJ)/def2-TZVPP/CPCM(DCM) level of theory. The donating orbital is shown in red and blue color, whereas the accepting orbital is displayed in yellow and green, both with an isovalue of 0.065 Å^{-3}. The significance of the given orbital pair with respect to the unidirectional and total CT energies is reflected in the percent values given in each subfigure.

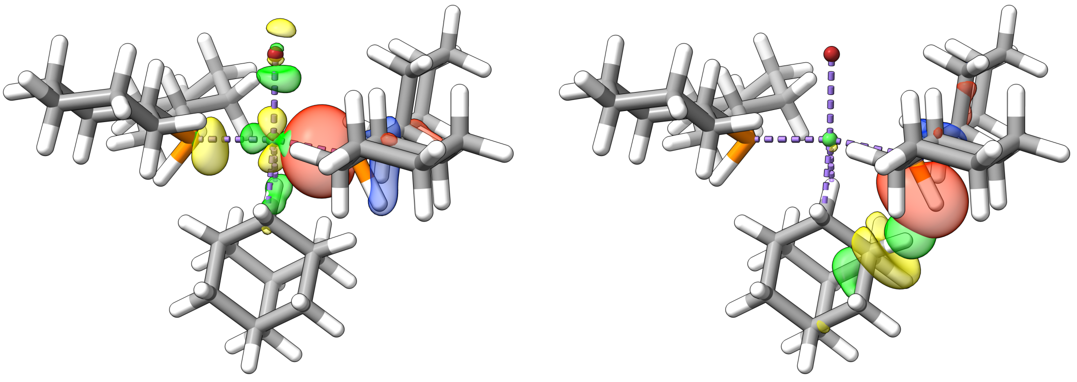

(a) $[Ni'CH_2]^+$: 90.7 % CTf, 67.3 % CT

(b) $[Ni'CH_2]^+$: 12.6 % CTf, 9.3 % CT

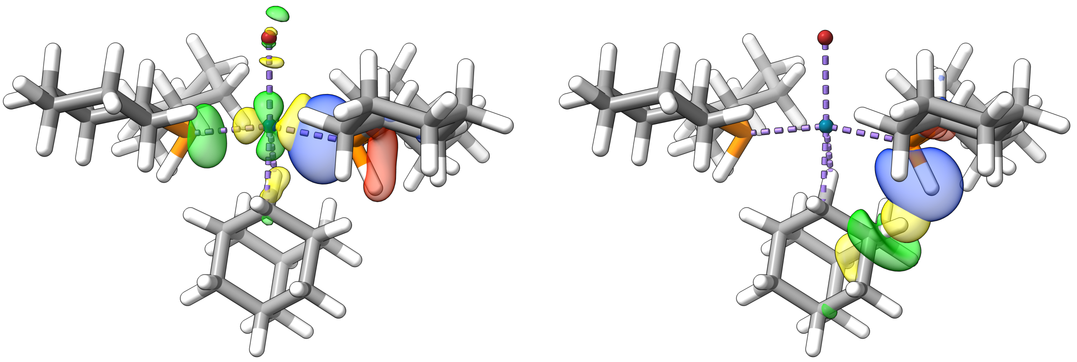

(c) $[Pd'CH_2]^+$: 95.4 % CTf, 77.4 % CT

(d) $[Pd'CH_2]^+$: 7.3 % CTf, 5.9 % CT

Figure S72. Two most significantly contributing COVPs for forward CT (CTf) in the sliced $[Ni'CH_2]^+$ (a+b) and $[Pd'CH_2]^+$ (c+d) complexes where $HPCy_2$ and all remaining parts of the complex (including adamantane and the metal) were chosen as the two fragments in VFB-ALMO-EDA calculations at the B3LYP-D3(BJ)/def2-TZVPP/CPCM(DCM) level of theory. The donating orbital is shown in red and blue color, whereas the accepting orbital is displayed in yellow and green, both with an isovalue of 0.065 Å^{-3}. The significance of the given orbital pair with respect to the unidirectional and total CT energies is reflected in the percent values given in each subfigure.

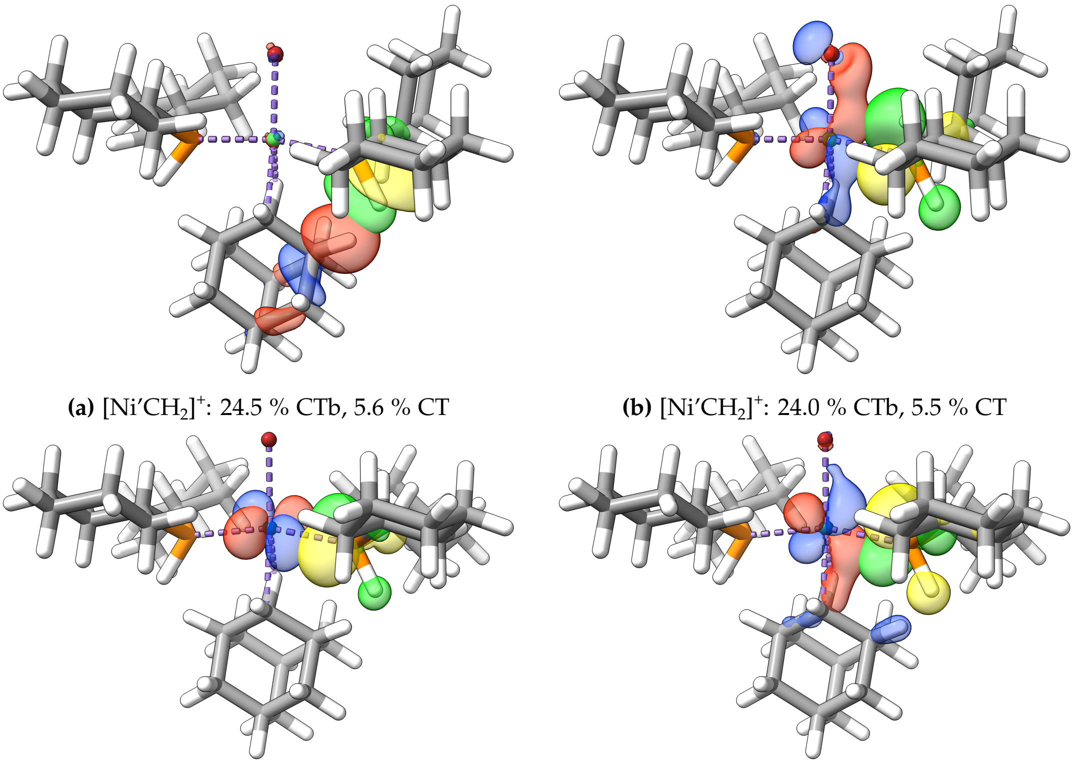

(a) $[Ni'CH_2]^+$: 24.5 % CTb, 5.6 % CT

(b) $[Ni'CH_2]^+$: 24.0 % CTb, 5.5 % CT

(c) $[Pd'CH_2]^+$: 26.9 % CTb, 4.3 % CT

(d) $[Pd'CH_2]^+$: 24.7 % CTb, 4.0 % CT

Figure S73. Two most significantly contributing COVPs for backward CT (CTb) in the sliced $[Ni'CH_2]^+$ (a+b) and $[Pd'CH_2]^+$ (c+d) complexes where $HPCy_2$ and all remaining parts of the complex (including adamantane and the metal) were chosen as the two fragments in VFB-ALMO-EDA calculations at the B3LYP-D3(BJ)/def2-TZVPP/CPCM(DCM) level of theory. The donating orbital is shown in red and blue color, whereas the accepting orbital is displayed in yellow and green, both with an isovalue of 0.065 Å^{-3}. The significance of the given orbital pair with respect to the unidirectional and total CT energies is reflected in the percent values given in each subfigure.

Table S24. ALMO-EDA based decomposition of the total interaction energy (in kcal/mol) at the PBE0-D3(BJ)/-mixed (def2-TZVP for Ni and Pd, def2-ECP for Pd, and de2-SVP for remaining atoms)/SMD(DCM) level of theory as obtained from fragmenting $[M'CH_2]+$ into adamantane ($[CH_2]$) and its corresponding remainder, including the metal center, or into one of the $HPCy_2$ ligands and resulting remainder ($HPCy_2$). The forward direction of charge flow is defined as the one from the ligand of interest to the fragment containing the metal center (e.g., $[CH_2] \rightarrow [Pd]$ for $[Pd'CH_2]+$).

Energy Component	$[Ni'CH_2]^+$ ($HPCy_2$)	$[Pd'CH_2]^+$ ($HPCy_2$)	$[Ni'CH_2]^+$ ($[CH_2]$)	$[Pd'CH_2]^+$ ($[CH_2]$)
ΔE_{INT}	−33.6	−36.2	2.1	−3.7
ΔE_{ELEC}	−119.5	−129.9	−86.5	−95.0
$\Delta E_{\mathrm{PAULI}}$	178.5	201.5	186.9	201.2
ΔE_{DISP}	−31.2	−31.6	−34.9	−35.8
ΔE_{FRZ}	27.8	40.0	65.5	70.4
ΔE_{POL}	−19.3	−15.9	−20.9	−16.0
ΔE_{CT}	−42.1	−60.2	−42.5	−58.1
ΔE_{CTf}	−32.1	−49.3	−25.8	−39.3
ΔE_{CTb}	−9.0	−9.5	−15.7	−17.3
$\Delta E_{\mathrm{CTf}}/\Delta E_{\mathrm{CTb}}$	3.6	5.2	1.6	2.3

Table S25. ALMO-EDA based decomposition of the total interaction energy (in kcal/mol) at the B3LYP-D3(BJ)/(def2-TZVPP, def2-ECP for Pd)/CPCM(DCM) level of theory as obtained from fragmenting $[M'CH_2]+$ into adamantane ($[CH_2]$) and its corresponding remainder, including the metal center, or into one of the $HPCy_2$ ligands and resulting remainder ($HPCy_2$). The forward direction of charge flow is defined as the one from the ligand of interest to the fragment containing the metal center (e.g., $[CH_2] \rightarrow [Pd]$ for $[Pd'CH_2]+$).

Energy Component	$[Ni'CH_2]^+$ ($HPCy_2$)	$[Pd'CH_2]^+$ ($HPCy_2$)	$[Ni'CH_2]^+$ ($[CH_2]$)	$[Pd'CH_2]^+$ ($[CH_2]$)
ΔE_{INT}	−25.6	−27.6	12.2	6.2
ΔE_{ELEC}	−119.3	−130.7	−81.3	−89.7
$\Delta E_{\mathrm{PAULI}}$	190.0	215.2	194.7	209.2
ΔE_{DISP}	−31.5	−32.4	−34.9	−36.5
ΔE_{FRZ}	39.2	52.1	78.5	83.1
ΔE_{POL}	−29.4	−24.8	−31.8	−25.3
ΔE_{CT}	−35.3	−54.9	−34.4	−51.6
ΔE_{CTf}	−26.2	−44.5	−18.4	−33.8
ΔE_{CTb}	−8.1	−8.9	−15.2	−16.2
$\Delta E_{\mathrm{CTf}}/\Delta E_{\mathrm{CTb}}$	3.2	5.0	1.2	2.1

IX. More Details on the Potential Implications of Acidification on Catalysis

Table S26. Relative energies at PBE0-D4/def-QZVPP/COSMO-RS(fine, HFIP) level of theory in kcal/mol between different intermediates from Figure 9 of the main text. These relative energies result from evaluating two different geometries for each metal. Ni and Pd correspond to the fully conformationally optimized structures as obtained from applying our workflow from Section II., whereas Ni@Pd and Pd@Ni were obtained from switching out the metals in their corresponding conformationally optimized structures (e.g., Pd was replaced with Ni in Ni@Pd) followed by a geometry optimization at r^2SCAN-3c/CPCM(HFIP) level of theory without allowing for major conformational changes.

Structure	Ni	Ni@Pd	Pd	Pd@Ni
[Int2-M]	2.4	0.0	0.0	3.3
[Int3-M]	6.1	0.0	0.0	5.4
[TS1-M]	0.4	0.0	0.5	0.0
[Int4-M]	0.0	0.2	0.3	0.0
[Int3′-M]$^-$ †	0.0	2.5	1.5	0.0

† ma-def2-QZVPP basis set was used to account for the need for diffuse functions (anionic system).

Application of our computational workflow as described in Section II. yielded structures with varying binding modes between the corresponding Ni and Pd species (see Figure 9b of the main text for the most extreme case). Table S26 reports on the relative energies of these structures when evaluated for both metals (see caption of Table S26 for more details).

Clearly, Table S26 indicates that our conformational workflow was not able to consistently recover the lowest-energy structures for both metals, with some of the manually generated structures, such as [Int2-Ni@Pd], being lower than the corresponding conformationally explored structures ([Int2-Ni] in the case of [Int2-Ni@Pd]). Unfortunately, this is not surprising given the fact that both the GOAT[14] procedure as well as CREST[16] calculations depend on the molecular topology of the systems, which is non-trivial to determine for borderline cases such as fluxional haptotropic ligands with a potentially changing binding motif throughout the simulations. Since both algorithms tend to remove structures exhibiting a considerably different topology, acceptance of intermediate structures with varied bonding motifs solely depends on the settings used for obtaining "covalent bonds" to determine the initial molecular topology. Hence, when no bond is detected, a new one may be formed during the conformational analysis, but not necessarily the other way around. This motivates the development of further run modes of these algorithms, allowing for more drastic local changes in the molecular topology via, e.g., user-specified input such as the first ligand shell atoms of the initial

complex geometry.

Nevertheless, despite these apparent deficiencies in detecting potentially lower-energy binding motifs, a manual evaluation is still possible, as has been carried out by substituting in both metals into the respective final structures of the corresponding other metal, with visual confirmation of the resulting optimized structures. This way, the changes in relative energy could be traced back to two structural reorganizations: 1. a change in acetate binding from κ^1 to κ^2, when going from, e.g, [Int2-Ni] to [Int2-Ni@Pd], and 2. a rearrangement of the agostic interaction from κ^2 to κ^3 for switching from, e.g., [Int3-Ni] to [Int3-Ni@Pd] (Figure 9b of the main text), which is both equally the case for the corresponding Pd species. Notably, the latter change in coordination was not reported by Houk and co-workers[19], which omitted any conformational analysis of potential dynamic binding in the original publication, thus including a significant error of at least 5 kcal mol^{-1} for [Int3-Pd] in their mechanistic analyses. Interestingly, Table S26 also suggests that genuine changes in the conformations ([Int3′-Ni@Pd] $\rightarrow$ [Int3′-Ni]) can be recovered with the conformational approach and that the topology issue described above seems specific to the metal identity (not an issue for Pd, but surely one for Ni).

Considering the minor energy penalties for [TS1-Pd] and [Int4-M], it is thus most reasonable to use Pd coordination geometries throughout to consistently compare intrinsic changes in the energetic landscapes due to changes in the metal identity. Hence, all structures from Figure 9a of the main text correspond to either Pd or Ni@Pd geometries, respectively. However, for assessing a more realistic potential acidification that could align more closely with experiments, we switch to the most populated structures, the lowest-energy ones, which are [Int3-Pd], [Int3′-Ni]$^-$ [Int3′-Pd@Ni]$^-$ and [Int3-Ni@Pd] in Figure 9c (orange) compared to the all Pd-based geometries [Int3-Pd], [Int3′-Ni@Pd]$^-$ [Int3′-Pd]$^-$ and [Int3-Ni@Pd] (Figure 9c, red).

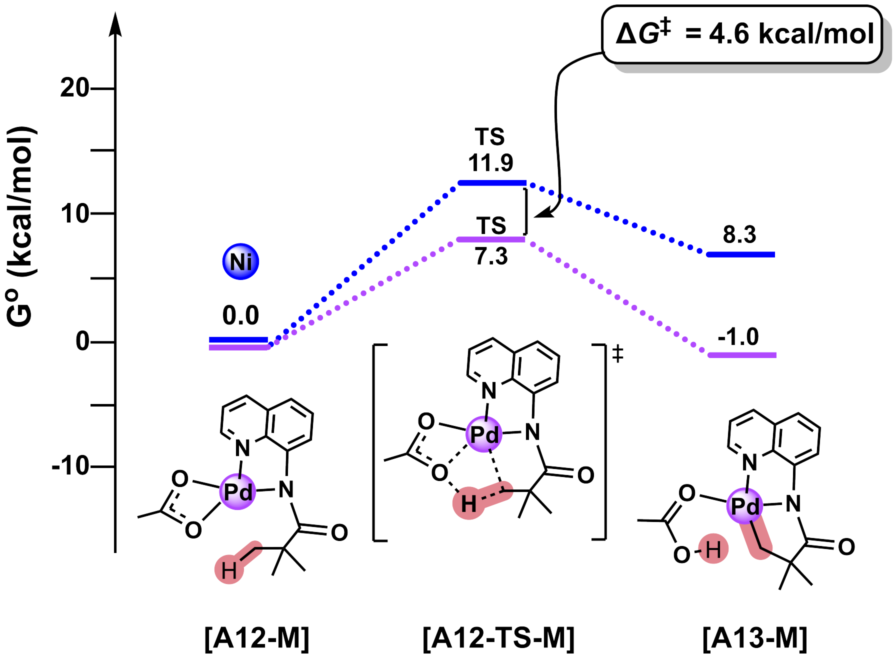

Figure S74. Free energy landscape for the deprotonation transition state of M(OAc)(LX) (M = Ni, Pd) via concerted metalation-deprotonation (CMD), showing the difference in barrier height ($\Delta G^{\dagger}$). Final energies computed at the PBE0-D4/def-QZVPP/COSMO-RS(fine, MeCN) level of theory based on "all-Ni geometries" (see Table S27). Compound labels used herein are identical to those used in the original manuscript.[80]

Interested in a potentially similar impact of a change in thermodynamics due to acidification on the resulting reaction kinetics[81,82], we probed the barrier height for C(sp^3)-H activation via CMD using Ni(LX)(OAc) and Pd(LX)(OAc) (LX = N-(quinolin-8-yl)pivalamide), a key on-cycle intermediate during catalysis[80,83,84] whose kinetics have also been studied using model Ni systems (Figure S74).[85,86] Again, we compared different binding modes for these complexes in Table S27 and thus use the ones with lower energy consistently, which are the Ni geometries for these systems. Notably, we also find a different low-energy binding mode for [A12-Pd] with acetate being κ^1-bound rather than κ^2 as reported by Chang and co-workers[80] (see Figure S75). This κ^1-binding of acetate is stablilized via an κ^3 agostic interaction between Pd and the *tert*-butyl group of the ligand framework. However, we note that the energy difference between the structures is small and within margin of our potential DFT and solvation errors (cf. Section VII.).

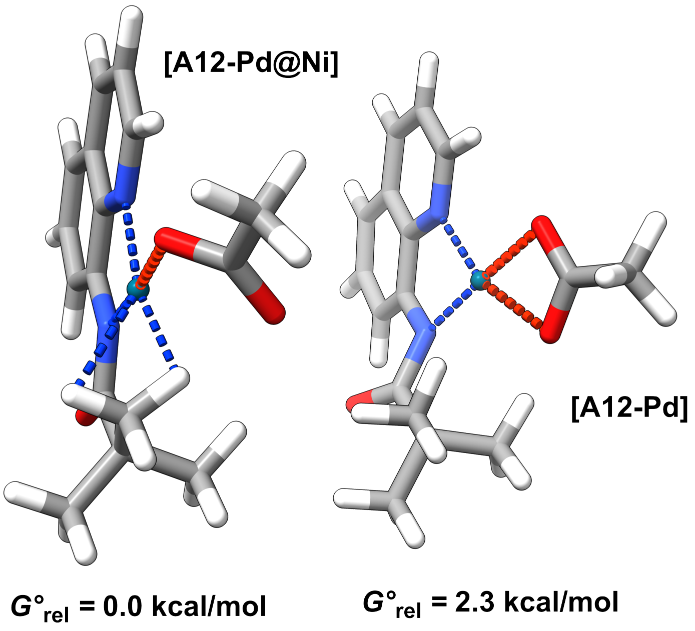

Figure S75. Comparison of the previously reported structure of [A12-Pd] (right) with the lowest energy structure as obtained in this study (left). Relative energies are given at the PBE0-D4/def-QZVPP/COSMO-RS(fine, MeCN) level of theory and the varying acetate binding mode is colored in red. Please also note that κ^1 acetate binding is only more favorable due to an κ^3 interaction with the *tert*-butyl group of the ligand.

Table S27. Relative energies at PBE0-D4/def-QZVPP/COSMO-RS(fine, MeCN) level of theory in kcal/mol between different intermediates for C(sp^3)-H activation via CMD. These relative energies result from evaluating two different geometries for each metal. Ni and Pd correspond to the fully conformationally optimized structures as obtained from applying our workflow from Section II., whereas Ni@Pd and Pd@Ni were obtained from switching out the metals in their corresponding conformationally optimized structures (e.g., Pd was replaced with Ni in Ni@Pd) followed by a geometry optimization at r^{2}SCAN-3c/CPCM(MeCN) level of theory without allowing for major conformational changes.

Structure	Ni	Ni@Pd	Pd	Pd@Ni
[A12-M]	0.0	0.1	2.3	0.0
[A12-TS-M]	0.0	0.0	0.2	0.0
[A13-M]	0.1	0.0	0.0	0.1

Nevertheless our DFT calculations predict a transition state barrier for C-H activation that is lowered by 4.6 kcal/mol upon Pd-substitution, suggesting that ground-state thermochemical modulation also has potential kinetic implications during catalytic turnover (Figure S74). This may also help explain the kinetic differences for β-H elimination in the isostructural Ni and Pd β-agostic complexes reported by Diao and co-workers ($\Delta\Delta G^\dagger \approx 6$ kcal/mol).[87] However, we again caution that for catalytic systems that undergo CMD-type pathways, internal bases will have different M-O/O-H bond strengths that might modulate their effective basicity when interacting with the acidified C-H bond. Similarly, the κ^3 agostic interaction of the *tert*-butyl

group is removed upon formation of the agostic interaction of interest in [A12-TS-M], ultimately also affecting the observed barrier heights and relative energies.

X. Additional Computational Data

Table S28. Key bond lengths of the coordinated CH_2—M unit of the full $[MCH_2]^+$ as well as the sliced $[M'CH_2]^+$ complexes as computed by DFT. H^s is the hydrogen of the CH_2-group that displays a shorter bond length to the metal, whereas H^l is the one with longer M—H distance. All bond lengths are given in Å.

Bond	$[NiCH_2]^+$	$[Ni'CH_2]^+$	$[PdCH_2]^+$	$[Pd'CH_2]^+$
d(M–H^s)	1.599	1.589	1.683	1.665
d(M–H^l)	2.072	2.075	2.306	2.313
d(C–H^s)	1.175	1.186	1.185	1.204
d(C–H^l)	1.110	1.111	1.102	1.103

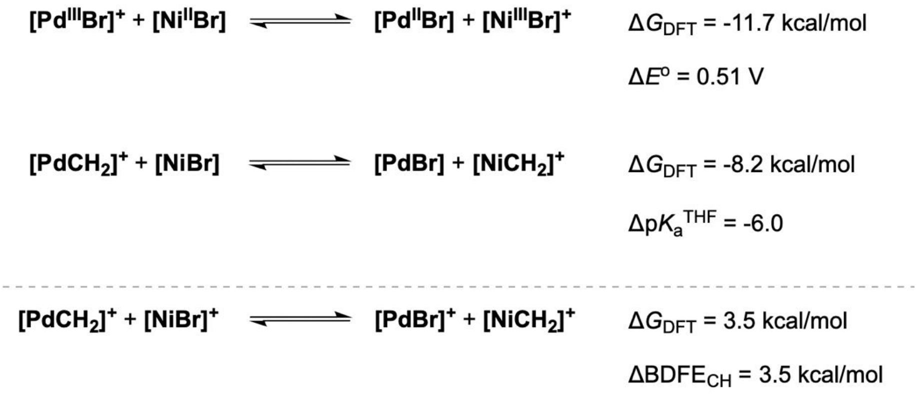

Figure S76. Computed isodesmic reaction equilibria in THF used to calculate ΔE^0, ΔpK_a(THF), and ΔBDFE(C—H). See Table 2 in the main text for results. Final energies computed at the PBE0-D4/def-QZVPP/COSMO-RS(fine) level of theory.

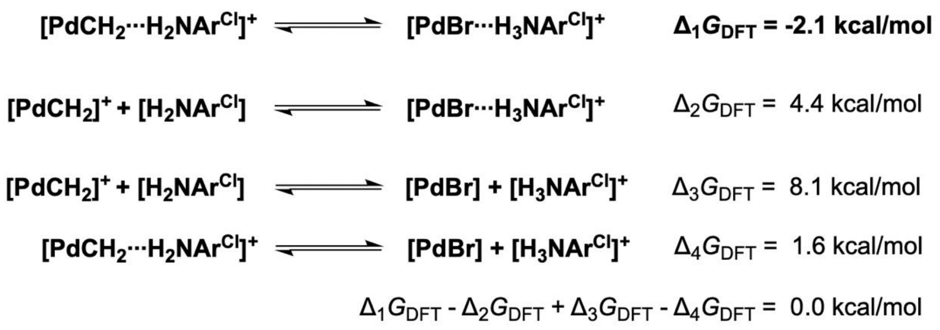

Figure S77. Computed free energies of all association and dissociation equilibria in DCE that were considered upon reacting [PdBr] with $[H_3NArCl]^+$, with $\Delta G_{exp} = -1.3 \pm 0.8$ kcal/mol. Final energies computed at the PBE0-D4/def-QZVPP/COSMO-RS(fine) level of theory.

XI. List of Abbreviations

aISS	automated interaction site screening
ALMO	absolutely localized molecular orbital
ALPB	analytical linearized Poisson-Boltzmann
AMAX	absolute maximal error
AO	atomic orbital
APAQ	acetyl-protected aminoquinoline
BDFE	bond dissociation free energy
BS	broken-symmetry
CE	conformer ensemble
CENSO	command-line energetic sorting
CMD	concerted metalation deprotonation
COSMO-RS	conductor like screening model for real solvents
COVP	complementary occupied-virtual pair
CPCM	conductor-like polarizable continuum model
CREST	conformer–rotamer ensemble sampling tool
CT	charge transfer
CTA	CT analysis
CTb	backward CT
CTf	forward CT
DCE	1,2-dichloroethane
DCM	dichloromethane
DFA	density functional approximation
DFT	density functional theory
ECP	effective core potential
EDA	energy decomposition analysis
EIE	equilibrium isotope effect
Fc^+/Fc	ferrocenium/ferrocene redox-couple
FMO	frontier MO
FRZ	frozen
GFN	geometries, vibrational frequencies and noncovalent interactions
GOAT	global optimization algorithm
HFIP	1,1,1,3,3,3–hexafluoro–2–propanol
HO	higher order
HOMO	highest occupied MO
LUMO	lowest unoccupied MO
MAE	mean absolute error
MAX	maximal error

Me	methyl
ME	mean error
MeCN	acetonitrile
MIN	minimal error
MO	molecular orbital
mRRHO	modified rigid-rotor harmonic-oscillator
MTD	meta-dynamics
NCI	non-covalent interactions
PES	potential energy surface
POL	polarized
RMSD	root-mean-square deviation
SCF	self-consistent field
SCF-MI	SCF for molecular interactions
SD	standard deviation
SMD	solvation model based on the solute electron density
SPH	single-point Hessian
SQM	semiempirical quantum mechanical
TEMPOH	1-hydroxy-2,2,6,6-tetramethylpiperidine
THF	tetrahydrofuran
TS	transition state
UFF	universal force field
VFB	variational forward-backward
XRD	X-ray diffraction
xTB	extended tight-binding

XIX. References

[1] S. Grimme, F. Bohle, A. Hansen, P. Pracht, S. Spicher, M. Stahn, "Efficient Quantum Chemical Calculation of Structure Ensembles and Free Energies for Nonrigid Molecules", *J. Phys. Chem. A* **2021**, *125*, 4039–4054.

[2] S. Grimme, "Supramolecular Binding Thermodynamics by Dispersion-Corrected Density Functional Theory", *Chem. Eur. J.* **2012**, *18*, 9955–9964.

[3] P. Pracht, S. Grimme, "Calculation of Absolute Molecular Entropies and Heat Capacities Made Simple", *Chem. Sci.* **2021**, *12*, 6551–6568.

[4] C. Plett, S. Grimme, "Automated and Efficient Generation of General Molecular Aggregate Structures", *Angew. Chem. Int. Ed.* **2023**, *62*, e202214477.

[5] C. Bannwarth, E. Caldeweyher, S. Ehlert, A. Hansen, P. Pracht, J. Seibert, S. Spicher, S. Grimme, "Review GFNn-xTB", *WIREs Comput Mol Sci.* **2021**, *11*, e1493.

[6] C. Bannwarth, S. Ehlert, S. Grimme, "GFN2-xTB—An Accurate and Broadly Parametrized Self-Consistent Tight-Binding Quantum Chemical Method with Multipole Electrostatics and Density-Dependent Dispersion Contributions", *J. Chem. Theory Comput.* **2019**, *15*, 1652–1671.

[7] S. Ehlert, M. Stahn, S. Spicher, S. Grimme, "Robust and Efficient Implicit Solvation Model for Fast Semiempirical Methods", *J. Chem. Theory Comput.* **2021**, *17*, 4250–4261.

[8] D. R. Lide, C. R. Company (Eds.), *CRC Handbook of Chemistry and Physics 90th ed.*, CRC Press, Boca Raton, FL, **2009**.

[9] S. Grimme, A. Hansen, S. Ehlert, J.-M. Mewes, "r^2SCAN-3c: A "Swiss Army Knife" Composite Electronic-Structure Method", *J. Chem. Phys.* **2021**, *154*, 064103.

[10] F. Neese, "Software Update: The ORCA Program System—Version 5.0", *WIREs Comput Mol Sci.* **2022**, *12*, e1606.

[11] F. Neese, "The SHARK Integral Generation and Digestion System", *J. Comput. Chem.* **2023**, *44*, 381–396.

[12] A. V. Marenich, C. J. Cramer, D. G. Truhlar, "Universal Solvation Model Based on Solute Electron Density and on a Continuum Model of the Solvent Defined by the Bulk Dielectric Constant and Atomic Surface Tensions", *J. Phys. Chem. B* **2009**, *113*, 6378–6396.

[13] S. Spicher, S. Grimme, "Single-Point Hessian Calculations for Improved Vibrational Frequencies and Rigid-Rotor-Harmonic-Oscillator Thermodynamics", *J. Chem. Theory Comput.* **2021**, *17*, 1701–1714.

[14] B. De Souza, "GOAT: A Global Optimization Algorithm for Molecules and Atomic Clusters", *Angew. Chem. Int. Ed.* **2025**, *64*, e202500393.

[15] S. Grimme, "Exploration of Chemical Compound, Conformer, and Reaction Space with Meta-Dynamics Simulations Based on Tight-Binding Quantum Chemical Calculations", *J. Chem. Theory Comput.* **2019**, *15*, 2847–2862.

[16] P. Pracht, F. Bohle, S. Grimme, "Automated Exploration of the Low-Energy Chemical

Space with Fast Quantum Chemical Methods", *Phys. Chem. Chem. Phys.* **2020**, *22*, 7169–7192.

[17] P. Pracht, S. Grimme, C. Bannwarth, F. Bohle, S. Ehlert, G. Feldmann, J. Gorges, M. Müller, T. Neudecker, C. Plett, S. Spicher, P. Steinbach, P. A. Wesołowski, F. Zeller, "CREST—A Program for the Exploration of Low-Energy Molecular Chemical Space", *J. Chem. Phys.* **2024**, *160*, 114110.

[18] J. M. Ramos-Villaseñor, E. Rodríguez-Cárdenas, C. E. Barrera Díaz, B. A. Frontana-Uribe, "Review—Use of 1,1,1,3,3,3–Hexafluoro–2–Propanol (HFIP) Co-Solvent Mixtures in Organic Electrosynthesis", *J. Electrochem. Soc.* **2020**, *167*, 155509.

[19] Y.-F. Yang, G. Chen, X. Hong, J.-Q. Yu, K. N. Houk, "The Origins of Dramatic Differences in Five-Membered vs Six-Membered Chelation of Pd(II) on Efficiency of C(Sp^3)–H Bond Activation", *J. Am. Chem. Soc.* **2017**, *139*, 8514–8521.

[20] S. Spicher, S. Grimme, "Robust Atomistic Modeling of Materials, Organometallic, and Biochemical Systems", *Angew. Chem. Int. Ed.* **2020**, *59*, 15665.

[21] V. Barone, M. Cossi, "Quantum Calculation of Molecular Energies and Energy Gradients in Solution by a Conductor Solvent Model", *J. Phys. Chem. A* **1998**, *102*, 1995–2001.

[22] M. Cossi, N. Rega, G. Scalmani, V. Barone, "Energies, Structures, and Electronic Properties of Molecules in Solution with the C-PCM Solvation Model", *J. Comput. Chem.* **2003**, *24*, 669.

[23] C. Adamo, V. Barone, "Toward Reliable Density Functional Methods without Adjustable Parameters: The PBE0 Model", *J. Chem. Phys.* **1999**, *110*, 6158–6170.

[24] E. Caldeweyher, C. Bannwarth, S. Grimme, "Extension of the D3 Dispersion Coefficient Model", *J. Chem. Phys.* **2017**, *147*, 034112.

[25] E. Caldeweyher, S. Ehlert, A. Hansen, H. Neugebauer, S. Spicher, C. Bannwarth, S. Grimme, "A Generally Applicable Atomic-Charge Dependent London Dispersion Correction", *J. Chem. Phys.* **2019**, *150*, 154122.

[26] F. Weigend, F. Furche, R. Ahlrichs, "Gaussian Basis Sets of Quadruple Zeta Valence Quality for Atoms H–Kr", *J. Chem. Phys.* **2003**, *119*, 12753–12762.

[27] F. Weigend, R. Ahlrichs, "Balanced Basis Sets of Split Valence, Triple Zeta Valence and Quadruple Zeta Valence Quality for H to Rn: Design and Assessment of Accuracy", *Phys. Chem. Chem. Phys.* **2005**, *7*, 3297–3305.

[28] D. Andrae, U. Häußermann, M. Dolg, H. Stoll, H. Preuß, “Energy-Adjustedab Initio Pseudopotentials for the Second and Third Row Transition Elements”, *Theoret. Chim. Acta* **1990**, *77*, 123–141.

[29] F. Neese, F. Wennmohs, A. Hansen, U. Becker, “Efficient, Approximate and Parallel Hartree–Fock and Hybrid DFT Calculations. A ‘Chain-of-Spheres’ Algorithm for the Hartree–Fock Exchange”, *Chem. Phys.* **2009**, *356*, 98–109.

[30] B. Helmich-Paris, B. De Souza, F. Neese, R. Izsák, “An Improved Chain of Spheres for Exchange Algorithm”, *J. Chem. Phys.* **2021**, *155*, 104109.

[31] F. Weigend, “Hartree–Fock Exchange Fitting Basis Sets for H to Rn”, *J. Comput. Chem.* **2008**, *29*, 167–175.

[32] J. Zheng, X. Xu, D. G. Truhlar, “Minimally Augmented Karlsruhe Basis Sets”, *Theor Chem Acc* **2011**, *128*, 295–305.

[33] V. N. Staroverov, G. E. Scuseria, J. Tao, J. P. Perdew, “Comparative Assessment of a New Nonempirical Density Functional: Molecules and Hydrogen-Bonded Complexes”, *J. Chem. Phys.* **2003**, *119*, 12129–12137.

[34] C. Lee, W. Yang, R. G. Parr, “Development of the Colle-Salvetti Correlation-Energy Formula into a Functional of the Electron Density”, *Phys. Rev. B* **1988**, *37*, 785.

[35] A. D. Becke, “Density-Functional Exchange-Energy Approximation with Correct Asymptotic Behavior”, *Phys. Rev. A* **1988**, *38*, 3098.

[36] M. Bursch, H. Neugebauer, S. Ehlert, S. Grimme, “Dispersion Corrected r^2SCAN Based Global Hybrid Functionals: r^2SCANh, r^2SCAN0, and r^2SCAN50”, *J. Chem. Phys.* **2022**, *156*, 134105.

[37] Y. Zhao, D. G. Truhlar, “Design of Density Functionals That Are Broadly Accurate for Thermochemistry, Thermochemical Kinetics, and Nonbonded Interactions”, *J. Phys. Chem. A* **2005**, *109*, 5656–5667.

[38] L. Wittmann, H. Neugebauer, S. Grimme, M. Bursch, “Dispersion-Corrected r^2SCAN Based Double-Hybrid Functionals”, *J. Chem. Phys.* **2023**, *159*, 224103.

[39] N. Mardirossian, M. Head-Gordon, “ωB97X-V: A 10-Parameter, Range-Separated Hybrid, Generalized Gradient Approximation Density Functional with Nonlocal Correlation, Designed by a Survival-of-the-Fittest Strategy”, *Phys. Chem. Chem. Phys.* **2014**, *16*, 9904–9924.

[40] O. A. Vydrov, T. Van Voorhis, "Nonlocal van Der Waals Density Functional: The Simpler the Better", *J. Chem. Phys.* **2010**, *133*, 244103.

[41] A. Klamt, "Conductor-like Screening Model for Real Solvents: A New Approach to the Quantitative Calculation of Solvation Phenomena", *J. Phys. Chem.* **1995**, *99*, 2224–2235.

[42] A. Klamt, V. Jonas, T. Bürger, J. C. W. Lohrenz, "Refinement and Parametrization of COSMO-RS", *J. Phys. Chem. A* **1998**, *102*, 5074–5085.

[43] A. Klamt, "The COSMO and COSMO-RS Solvation Models", *WIREs Comput Mol Sci.* **2011**, *1*, 699–709.

[44] C. J. Cramer, *Essentials of Computational Chemistry: Theories and Models 2nd ed.*, Wiley, Chichester, West Sussex, England; Hoboken, NJ, **2004**.

[45] O. Hammerich, B. Speiser (Eds.), *Organic Electrochemistry: Revised and Expanded 5th ed.*, CRC Press, Boca Raton, FL, **2015**.

[46] J. Ho, "Are Thermodynamic Cycles Necessary for Continuum Solvent Calculation of pKas and Reduction Potentials?", *Phys. Chem. Chem. Phys.* **2015**, *17*, 2859–2868.

[47] J. Ho, M. Z. Ertem, "Calculating Free Energy Changes in Continuum Solvation Models", *J. Phys. Chem. B* **2016**, *120*, 1319–1329.

[48] F. Wang, N. Mohammadi, S. P. Best, D. Appadoo, C. T. Chantler, "Dominance of Eclipsed Ferrocene Conformer in Solutions Revealed by the IR Spectra between 400 and 500 Cm-1", *Radiat. Phys. Chem.* **2021**, *188*, 109590.

[49] K. S. Alongi, G. C. Shields in *Annual Reports in Computational Chemistry*, *Vol. 6*, Elsevier, **2010**, pp. 113–138.

[50] S. Sastre, R. Casasnovas, F. Muñoz, J. Frau, "Isodesmic Reaction for Accurate Theoretical pK_a Calculations of Amino Acids and Peptides", *Phys. Chem. Chem. Phys.* **2016**, *18*, 11202–11212.

[51] T. Rodima, I. Kaljurand, A. Pihl, V. Mäemets, I. Leito, I. A. Koppel, "Acid-Base Equilibria in Nonpolar Media. 2. Self-Consistent Basicity Scale in THF Solution Ranging from 2-Methoxypyridine to EtP1(Pyrr) Phosphazene", *J. Org. Chem.* **2002**, *67*, 1873–1881.

[52] W. Treyde, K. Riedmiller, F. Gräter, "Bond Dissociation Energies of X–H Bonds in Proteins", *RSC Adv.* **2022**, *12*, 34557–34564.

[53] C. F. Wise, R. G. Agarwal, J. M. Mayer, "Determining Proton-Coupled Standard Potentials

and X–H Bond Dissociation Free Energies in Nonaqueous Solvents Using Open-Circuit Potential Measurements", *J. Am. Chem. Soc.* **2020**, *142*, 10681–10691.

[54] E. F. Pettersen, T. D. Goddard, C. C. Huang, G. S. Couch, D. M. Greenblatt, E. C. Meng, T. E. Ferrin, "UCSF Chimera—A Visualization System for Exploratory Research and Analysis", *J. Comput. Chem.* **2004**, *25*, 1605–1612.

[55] E. F. Pettersen, T. D. Goddard, C. C. Huang, E. C. Meng, G. S. Couch, T. I. Croll, J. H. Morris, T. E. Ferrin, "UCSF ChimeraX : Structure Visualization for Researchers, Educators, and Developers", *Protein Sci.* **2021**, *30*, 70–82.

[56] Y. Mao, M. Loipersberger, P. R. Horn, A. Das, O. Demerdash, D. S. Levine, S. Prasad Vecham, T. Head-Gordon, M. Head-Gordon, "From Intermolecular Interaction Energies and Observable Shifts to Component Contributions and Back Again: A Tale of Variational Energy Decomposition Analysis", *Annu. Rev. Phys. Chem.* **2021**, *72*, 641.

[57] P. R. Horn, Y. Mao, M. Head-Gordon, "Probing Non-Covalent Interactions with a Second Generation Energy Decomposition Analysis Using Absolutely Localized Molecular Orbitals", *Phys. Chem. Chem. Phys.* **2016**, *18*, 23067.

[58] R. Z. Khaliullin, E. A. Cobar, R. C. Lochan, A. T. Bell, M. Head-Gordon, "Unravelling the Origin of Intermolecular Interactions Using Absolutely Localized Molecular Orbitals", *J. Phys. Chem. A* **2007**, *111*, 8753.

[59] E. Epifanovsky, A. T. B. Gilbert, X. Feng, J. Lee, Y. Mao, N. Mardirossian, P. Pokhilko, A. F. White, M. P. Coons, A. L. Dempwolff, Z. Gan, D. Hait, P. R. Horn, L. D. Jacobson, I. Kaliman, J. Kussmann, A. W. Lange, K. U. Lao, D. S. Levine, J. Liu, S. C. McKenzie, A. F. Morrison, K. D. Nanda, F. Plasser, D. R. Rehn, M. L. Vidal, Z.-Q. You, Y. Zhu, B. Alam, B. J. Albrecht, A. Aldossary, E. Alguire, J. H. Andersen, V. Athavale, D. Barton, K. Begam, A. Behn, N. Bellonzi, Y. A. Bernard, E. J. Berquist, H. G. A. Burton, A. Carreras, K. Carter-Fenk, R. Chakraborty, A. D. Chien, K. D. Closser, V. Cofer-Shabica, S. Dasgupta, M. de Wergifosse, J. Deng, M. Diedenhofen, H. Do, S. Ehlert, P.-T. Fang, S. Fatehi, Q. Feng, T. Friedhoff, J. Gayvert, Q. Ge, G. Gidofalvi, M. Goldey, J. Gomes, C. E. González-Espinoza, S. Gulania, A. O. Gunina, M. W. D. Hanson-Heine, P. H. P. Harbach, A. Hauser, M. F. Herbst, M. Hernández Vera, M. Hodecker, Z. C. Holden, S. Houck, X. Huang, K. Hui, B. C. Huynh, M. Ivanov, Á. Jász, H. Ji, H. Jiang, B. Kaduk, S. Kähler, K. Khistyaev, J. Kim, G. Kis, P. Klunzinger, Z. Koczor-Benda, J. H. Koh, D. Kosenkov, L. Koulias, T. Kowalczyk, C. M. Krauter, K. Kue, A. Kunitsa, T. Kus, I. Ladjánszki, A. Landau, K. V. Lawler, D. Lefrancois, S. Lehtola, R. R. Li, Y.-P. Li, J. Liang, M. Liebenthal, H.-H. Lin, Y.-S. Lin, F. Liu, K.-Y. Liu, M. Loipersberger, A. Luenser, A. Manjanath, P. Manohar, E. Mansoor, S. F. Manzer, S.-P. Mao, A. V. Marenich, T. Markovich, S. Mason, S. A. Mau-

rer, P. F. McLaughlin, M. F. S. J. Menger, J.-M. Mewes, S. A. Mewes, P. Morgante, J. W. Mullinax, K. J. Oosterbaan, G. Paran, A. C. Paul, S. K. Paul, F. Pavošević, Z. Pei, S. Prager, E. I. Proynov, Á. Rák, E. Ramos-Cordoba, B. Rana, A. E. Rask, A. Rettig, R. M. Richard, F. Rob, E. Rossomme, T. Scheele, M. Scheurer, M. Schneider, N. Sergueev, S. M. Sharada, W. Skomorowski, D. W. Small, C. J. Stein, Y.-C. Su, E. J. Sundstrom, Z. Tao, J. Thirman, G. J. Tornai, T. Tsuchimochi, N. M. Tubman, S. P. Veccham, O. Vydrov, J. Wenzel, J. Witte, A. Yamada, K. Yao, S. Yeganeh, S. R. Yost, A. Zech, I. Y. Zhang, X. Zhang, Y. Zhang, D. Zuev, A. Aspuru-Guzik, A. T. Bell, N. A. Besley, K. B. Bravaya, B. R. Brooks, D. Casanova, J.-D. Chai, S. Coriani, C. J. Cramer, G. Cserey, A. E. DePrince, R. A. DiStasio, A. Dreuw, B. D. Dunietz, T. R. Furlani, W. A. Goddard, S. Hammes-Schiffer, T. Head-Gordon, W. J. Hehre, C.-P. Hsu, T.-C. Jagau, Y. Jung, A. Klamt, J. Kong, D. S. Lambrecht, W. Liang, N. J. Mayhall, C. W. McCurdy, J. B. Neaton, C. Ochsenfeld, J. A. Parkhill, R. Peverati, V. A. Rassolov, Y. Shao, L. V. Slipchenko, T. Stauch, R. P. Steele, J. E. Subotnik, A. J. W. Thom, A. Tkatchenko, D. G. Truhlar, T. Van Voorhis, T. A. Wesolowski, K. B. Whaley, H. L. Woodcock, P. M. Zimmerman, S. Faraji, P. M. W. Gill, M. Head-Gordon, J. M. Herbert, A. I. Krylov, "Software for the Frontiers of Quantum Chemistry: An Overview of Developments in the Q-Chem 5 Package", *J. Chem. Phys.* **2021**, *155*, 084801.

[60] P. R. Horn, Y. Mao, M. Head-Gordon, "Defining the Contributions of Permanent Electrostatics, Pauli Repulsion, and Dispersion in Density Functional Theory Calculations of Intermolecular Interaction Energies", *J. Chem. Phys.* **2016**, *144*, 114107.

[61] M. Loipersberger, Y. Mao, M. Head-Gordon, "Variational Forward–Backward Charge Transfer Analysis Based on Absolutely Localized Molecular Orbitals: Energetics and Molecular Properties", *J. Chem. Theory Comput.* **2020**, *16*, 1073.

[62] S. P. Veccham, J. Lee, Y. Mao, P. R. Horn, M. Head-Gordon, "A Non-Perturbative Pairwise-Additive Analysis of Charge Transfer Contributions to Intermolecular Interaction Energies", *Phys. Chem. Chem. Phys.* **2021**, *23*, 928.

[63] R. Z. Khaliullin, A. T. Bell, M. Head-Gordon, "Analysis of Charge Transfer Effects in Molecular Complexes Based on Absolutely Localized Molecular Orbitals", *The Journal of Chemical Physics* **2008**, *128*, 184112.

[64] S. Grimme, S. Ehrlich, L. Goerigk, "Effect of the Damping Function in Dispersion Corrected Density Functional Theory", *J. Comput. Chem.* **2011**, *32*, 1456.

[65] S. Grimme, "Semiempirical GGA-type Density Functional Constructed with a Long-Range Dispersion Correction", *J. Comput. Chem.* **2006**, *27*, 1787–1799.

[66] S. Grimme, J. Antony, S. Ehrlich, H. Krieg, "A Consistent and Accurate *Ab Initio*

Parametrization of Density Functional Dispersion Correction (DFT-D) for the 94 Elements H-Pu", *J. Chem. Phys.* **2010**, *132*, 154104.

[67] P. R. Horn, M. Head-Gordon, "Polarization Contributions to Intermolecular Interactions Revisited with Fragment Electric-Field Response Functions", *J. Chem. Phys.* **2015**, *143*.

[68] K. J. Kron, S. J. Gomez, Y. Mao, R. J. Cave, S. Mallikarjun Sharada, "Computational Analysis of Electron Transfer Kinetics for CO_2 Reduction with Organic Photoredox Catalysts", *J. Phys. Chem. A* **2020**, *124*, 5359–5368.

[69] Z. R. Wong, T. K. Schramm, M. Loipersberger, M. Head-Gordon, F. D. Toste, "Revisiting the Bonding Model for Gold(I) Species: The Importance of Pauli Repulsion Revealed in a Gold(I)-Cyclobutadiene Complex", *Angew Chem Int Ed* **2022**, *61*.

[70] Y. Mao, M. Loipersberger, K. J. Kron, J. S. Derrick, C. J. Chang, S. M. Sharada, M. Head-Gordon, "Consistent Inclusion of Continuum Solvation in Energy Decomposition Analysis: Theory and Application to Molecular CO $_2$ Reduction Catalysts", *Chem. Sci.* **2021**, *12*, 1398.

[71] A. K. Rappe, C. J. Casewit, K. S. Colwell, W. A. Goddard, W. M. Skiff, "UFF, a Full Periodic Table Force Field for Molecular Mechanics and Molecular Dynamics Simulations", *J. Am. Chem. Soc.* **1992**, *114*, 10024.

[72] S. Dasgupta, J. M. Herbert, "Standard Grids for High-precision Integration of Modern Density Functionals: SG-2 and SG-3", *J. Comput. Chem.* **2017**, *38*, 869–882.

[73] M. Müller, A. Hansen, S. Grimme, "ωB97X-3c: A Composite Range-Separated Hybrid DFT Method with a Molecule-Optimized Polarized Valence Double-ζ Basis Set", *J. Chem. Phys.* **2023**, *158*, 014103.

[74] K. R. Bryenton, A. A. Adeleke, S. G. Dale, E. R. Johnson, "Delocalization Error: The Greatest Outstanding Challenge in Density-functional Theory", *WIREs Comput Mol Sci.* **2023**, *13*.

[75] T. Froitzheim, M. Müller, A. Hansen, S. Grimme, *G-xTB: A General-Purpose Extended Tight-Binding Electronic Structure Method For the Elements H to Lr (Z=1–103)*, **2025**.

[76] S. Dohm, A. Hansen, M. Steinmetz, S. Grimme, M. P. Checinski, "Comprehensive Thermochemical Benchmark Set of Realistic Closed-Shell Metal Organic Reactions", *J. Chem. Theory Comput.* **2018**, *14*, 2596–2608.

[77] L. R. Maurer, M. Bursch, S. Grimme, A. Hansen, "Assessing Density Functional Theory

for Chemically Relevant Open-Shell Transition Metal Reactions", *J. Chem. Theory Comput.* **2021**, *17*, 6134–6151.

[78] A. Klamt, F. Eckert, M. Diedenhofen, M. E. Beck, "First Principles Calculations of Aqueous pKa Values for Organic and Inorganic Acids Using COSMO-RS Reveal an Inconsistency in the Slope of the pKa Scale", *J. Phys. Chem. A* **2003**, *107*, 9380–9386.

[79] R. B. Calvert, J. R. Shapley, "Decacarbonyl(Methyl)Hydrotriosmium: NMR Evidence for a Carbon.Cntdot..Cntdot.Hydrogen.Cntdot..Cntdot.Osmium Interaction", *J. Am. Chem. Soc.* **1978**, *100*, 7726–7727.

[80] Y. B. Kim, J. Won, J. Lee, J. Kim, B. Zhou, J.-W. Park, M.-H. Baik, S. Chang, "Ni-Catalyzed Intermolecular C(Sp^3)–H Amidation Tuned by Bidentate Directing Groups", *ACS Catal.* **2021**, *11*, 3067–3072.

[81] J. M. Anglada, E. Besalu, J. M. Bofill, R. Crehuet, "Prediction of Approximate Transition States by Bell-Evans-Polanyi Principle: I", *J. Comput. Chem.* **1999**, *20*, 1112–1129.

[82] J. Cheng, P. Hu, P. Ellis, S. French, G. Kelly, C. M. Lok, "Brønsted-Evans-Polanyi Relation of Multistep Reactions and Volcano Curve in Heterogeneous Catalysis", *J. Phys. Chem. C* **2008**, *112*, 1308–1311.

[83] V. G. Zaitsev, D. Shabashov, O. Daugulis, "Highly Regioselective Arylation of Sp^3 C-H Bonds Catalyzed by Palladium Acetate", *J. Am. Chem. Soc.* **2005**, *127*, 13154–13155.

[84] D. Shabashov, O. Daugulis, "Auxiliary-Assisted Palladium-Catalyzed Arylation and Alkylation of Sp^2 and Sp^3 Carbon-Hydrogen Bonds", *J. Am. Chem. Soc.* **2010**, *132*, 3965–3972.

[85] D. D. Beattie, A. C. Grunwald, T. Perse, L. L. Schafer, J. A. Love, "Understanding Ni(II)-Mediated C(Sp^3)–H Activation: Tertiary Ureas as Model Substrates", *J. Am. Chem. Soc.* **2018**, *140*, 12602–12610.

[86] B. E. Nadeau, D. D. Beattie, E. K. J. Lui, M. Tewkesbury, J. A. Love, L. L. Schafer, "Electronic Directing Group Modification for Improved Ni(II)-Mediated C(Sp^3)–H Activation: A Hammett Investigation of 8-Aminoquinoline", *Organometallics* **2023**, *42*, 2326–2334.

[87] H. Xu, C. T. Hu, X. Wang, T. Diao, "Structural Characterization of β-Agostic Bonds in Pd-Catalyzed Polymerization", *Organometallics* **2017**, *36*, 4099–4102.
